# Varicose veins of lower extremities: Insights from the first large-scale genetic study

**DOI:** 10.1371/journal.pgen.1008110

**Published:** 2019-04-18

**Authors:** Alexandra S. Shadrina, Sodbo Z. Sharapov, Tatiana I. Shashkova, Yakov A. Tsepilov

**Affiliations:** 1 Laboratory of Theoretical and Applied Functional Genomics, Novosibirsk State University, Novosibirsk, Russia; 2 Laboratory of Recombination and Segregation Analysis, Institute of Cytology and Genetics, Novosibirsk, Russia; 3 Laboratory of Pharmacogenomics, Institute of Chemical Biology and Fundamental Medicine, Novosibirsk, Russia; 4 Knomics LLC, Skolkovo Innovation Center, Moscow, Russia; 5 Department of Biological and Medical Physics, Moscow Institute of Physics and Technology, Moscow, Russia; 6 Research and Training Center on Bioinformatics, A.A. Kharkevich Institute for Information Transmission Problems, Moscow, Russia; Newcastle University, UNITED KINGDOM

## Abstract

Varicose veins of lower extremities (VVs) are a common multifactorial vascular disease. Genetic factors underlying VVs development remain largely unknown. Here we report the first large-scale study of VVs performed on a freely available genetic data of 408,455 European-ancestry individuals. We identified the 12 reliably associated loci that explain 13% of the SNP-based heritability, and prioritized the most likely causal genes *CASZ1*, *PIEZO1*, *PPP3R1*, *EBF1*, *STIM2*, *HFE*, *GATA2*, *NFATC2*, and *SOX9*. VVs-associated variants within these loci exhibited pleiotropic effects on several phenotypes including blood pressure/hypertension and blood cell traits. Gene set enrichment analysis revealed gene categories related to abnormal vasculogenesis. Genetic correlation analysis confirmed known epidemiological associations between VVs and deep venous thrombosis, weight, rough labor, and standing job, and found a genetic overlap with multiple traits that have not been previously suspected to share common genetic background with VVs. These traits included educational attainment, fluid intelligence and prospective memory scores, walking pace (negative correlation with VVs), smoking, height, number of operations, pain, and gonarthrosis (positive correlation with VVs). Finally, Mendelian randomization analysis provided evidence for causal effects of plasma levels of MICB and CD209 proteins, and anthropometric traits such as waist and hip circumference, height, weight, and both fat and fat-free mass. Our results provide novel insight into both VVs genetics and etiology. The revealed genes and proteins can be considered as good candidates for follow-up functional studies and might be of interest as potential drug targets.

## Author summary

Varicose veins of lower extremities (VVs) affect about 30% of adults in developed countries and cause both cosmetic and health problems. A strong body of evidence indicates that heredity plays an important role in the etiology of this condition. However, genetic basis of VVs remains poorly understood. Here, we present the results of the first large-scale genetic study for VVs. We identified genes which are the most likely involved in VVs pathogenesis. We show that VVs are correlated at a genetic level with numerous traits and phenotypes, including those already known from prior epidemiological studies (deep venous thrombosis, body mass index, standing job, etc.) as well as with those that have not been suspected to share common genetic background with VVs (fluid intelligence and prospective memory scores, smoking, walking pace, pain all over the body, and other traits). Finally, using genetic variants as instruments, we demonstrate direct causal effects of the traits related to anthropometry, such as height and weight, and plasma levels of immune-related proteins MICB and CD209. Our study provides novel insight into both VVs genetics and etiology. The revealed genes (*CASZ1*, *PIEZO1*, *PPP3R1*, *EBF1*, *STIM2*, *HFE*, *GATA2*, *NFATC2*, and *SOX9*) and proteins (MICB and CD209) can be considered as good candidates for follow-up functional studies and might be of interest as potential drug targets.

## Introduction

Varicose veins (VVs) are one of the clinical manifestations of chronic venous disease posing both a cosmetic and medical problem. VVs can be found in different parts of the body, but most commonly occur in the lower extremities. Prevalence estimates of this condition vary across ethnic groups ranging from 2–4% in the Northern group of the Cook Islands to 50–60% in some countries of the Western world [1]. Increased age, female sex, number of pregnancies, obesity, history of deep venous thrombosis, and standing occupation are among other risk factors [2]. VVs not related to the post-thrombotic syndrome or venous malformations are defined as primary VVs.

Pathogenesis of VVs is still not fully clarified. According to current understanding, key factors implicated in VVs development include changes in hemodynamic forces (decrease in laminar shear stress and increase in venous filling pressure), endothelial activation, inflammation, hypoxia, and dysregulation of matrix metalloproteinases and their tissue inhibitors [3–5]. These alterations underlie pathological remodeling of the vascular wall and loss of its tone. Questions remain about the order of events and the primary stimulus triggering the set of disease-related changes.

The cumulative evidence from epidemiological, family, and genetic association studies strongly indicates that there is a hereditary component in VVs etiology [6–8]. However, despite progress in this field [9–12], current knowledge of the genetic basis of this pathology is far from being complete. Elucidating genes involved in susceptibility to VVs would help to identify key molecular players in the disease initiation, provide deeper insights into its pathogenesis, and eventually contribute to development of improved targeted therapy aimed at VVs treating and preventing.

Large-scale biobanks linked to electronic health records open up unparalleled opportunities to investigate the genetics of complex traits. Today, UK Biobank is the largest repository that contains information on genotypes and phenotypes for half a million participating individuals [13]. This resource is open to all bona fide researchers, and access to data is provided upon approval of their application and payment of necessary costs. However, the need to incur high costs related to data access and computation can be an insurmountable obstacle for those who cannot afford these expenses. The Neale Lab (http://www.nealelab.is/) and the Gene ATLAS [14] (http://geneatlas.roslin.ed.ac.uk/) are two independent projects, which intend to remove this burden by generating genetic association data and sharing them with broader scientific community. These resources provide free open access to “quick-and-dirty” genome-wide association study (GWAS) summary statistics for a wide range of phenotypes measured in the UK Biobank. In our study, we aimed to employ state-of-the-art bioinformatics approaches to extract maximum possible information from these open resources with regard to the genetics of VVs of lower extremities. Our objectives were to (1) identify genetic loci reliably associated with VVs risk and prioritize the genes that account for the revealed associations, (2) elucidate pleiotropic effects of identified loci, (3) investigate genetic overlap between VVs and other complex traits, (4) gain etiological insights and explore cause-and-effect relationships by means of Mendelian randomization analysis.

## Results

### Study design, assumptions, and limitations

**Fig 1:**
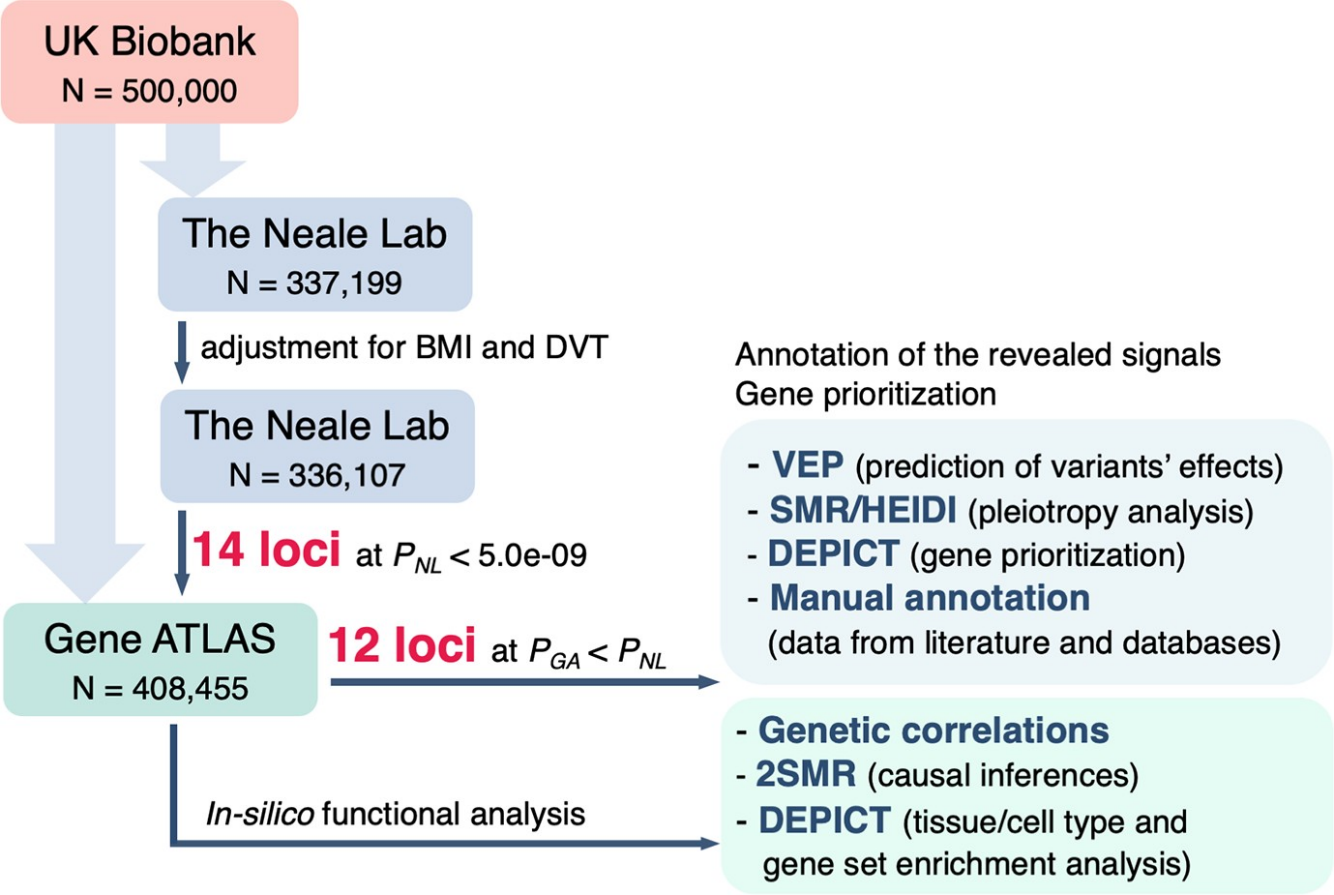
Scheme depicting the overall workflow of our study. GWAS summary statistics for VVs was obtained from the Gene ATLAS (larger sample size, less stringent filtering criteria) and the Neale Lab database (smaller sample size, more stringent filtering criteria). Search for associated loci was performed using the Neale Lab data adjusted for two potential confounders–body mass index (BMI) and deep venous thrombosis (DVT). GWAS summary statistics for these two traits were also obtained from the Neale Lab database. Since our study design did not imply a replication stage, genome-wide significance threshold was set at 5.0e-09. To be more rigorous, we filtered out those loci for which *P*-values in the Gene ATLAS dataset were higher than the corresponding *P*-values in the Neale Lab dataset (after all corrections). For the resulting 12 loci, we performed a functional annotation analysis and prioritized the most likely causal genes. Except for VEP, all *in-silico* analyses were conducted using the Gene ATLAS dataset since it enables to achieve the highest statistical power and is genetically equivalent to other studied VVs datasets (genetic correlation coefficient = 0.99–1.00, S1 Table).

Our study was designed as a one-stage GWAS followed by downstream bioinformatics analysis. The overall workflow of the study is depicted in Fig 1. All calculations were entirely based on the UK Biobank data for white British individuals available in open access databases: the Neale Lab database and the Gene ATLAS database. These projects used different software, methods of analysis, and quality control approaches. The Gene ATLAS project applied less stringent filtering criteria and therefore had larger sample size. We calculated genetic correlations between VVs in both public databases and found out that these traits are almost genetically equivalent (genetic correlation coefficient = 0.99, S1 Table).

The identification of VVs-associated loci and the search for functional variants was carried out using GWAS summary statistics provided by the Neale Lab. Since no replication was performed, significance threshold was raised from 5.0e-08 to a more conservative level of 5.0e-09. Moreover, we filtered out loci associated with VVs in the Gene ATLAS dataset at *P*-values ≥ *P*-values in the Neale Lab dataset (assuming that large sample size provides higher level of statistical significance). Thus, only signals with more convincing association data have been left for further analysis and interpretation.

Functional bioinformatics analysis was conducted using the Gene ATLAS data.

In carrying out this study, we had to face a number of challenges and limitations that must be acknowledged. The first limitation was intrinsic to the general approach to phenotype definition based on the electronic medical records system, which was employed in the UK Biobank study. Phenotype “VVs of lower extremities” was defined based on International Classification of Disease (ICD-10) billing code “I83” present in the electronic patient record. The Neale Lab reported the phenotype prevalence of 2.1%. It is much lower than VVs prevalence estimated by European epidemiological studies. Despite the evidence of a “healthy volunteer” selection bias in the UK Biobank study [15], such a low rate indicates that a proportion of individuals remained undiagnosed. This is in line with a recent primary healthcare register-based study reporting VVs prevalence rate of 3% in German general practice [16]. This phenomenon could be explained by a non-life-threatening nature of varicose veins, which might discourage patients from communication to the doctor. Given that individuals not diagnosed with I83 served as controls in our study, we could therefore expect an overall decrease in the statistical power to detect gene-disease associations. Another potential source of missing associations was immensely strict criteria used by the Neale Lab for single nucleotide polymorphisms (SNPs) quality control removing around 75% of SNPs initially provided by UK Biobank.

The next important limitation arose from the lack of access to individual-level data resulting in the inability to control a possible selection or sampling bias. We suggested that traits related to VVs risk factors could potentially cause unequal representation of patients with different characteristics in the case and the control groups, and thereby induce spurious associations or effect modification. The Neale Lab analyses were adjusted for sex, but other factors were beyond our control. In particular, we did not know how body mass index (BMI) was distributed in the case and the control groups and what proportion of patients in each group had deep venous thrombosis (DVT). In order to address this challenge, we performed an adjustment for these potential confounders by implementation of the method based on GWAS summary statistics [17, 18] (Supplemental Methods, Section 4).

Summarizing the above, we can state that the limitations of our study were mainly related to the loss of statistical power and a “quick-and-dirty” approach to summary statistics generation. Nevertheless, given a large sample size and a large number of associations tested, we assume that this obstacle could be at least partially compensated by a huge scale of the UK Biobank study itself.

### Genome-wide association study for VVs of lower extremities

#### Locus definition

We defined associated loci as regions within 250 kb from the lead SNP and reported only the most significant SNP hits per locus.

Fourteen loci were identified that met a genome-wide level of statistical significance of *P* < 5.0e-09 as provided by the Neale Lab (Table 1, expanded data can be found in S2 Table). Adjustment for DVT and BMI only slightly changed the observed effects of SNPs, and correction for the LD Score regression intercept (1.0403) did not eliminate or yield additional genome-wide significant loci. However, associations of loci tagged by rs7856039 and rs192647746 had higher *P*-values in the Gene ATLAS database than in the Neale Lab database. These loci were omitted from further analysis.

**Table 1:** Top SNPs associated with varicose veins of lower extremities.

SNP	Chr: position*	EffA/RefA†	EAF	GWAS for VVs, the Neale Lab data adjusted for BMI and DVT, N = 336,107				GWAS for VVs, the Gene ATLAS data, N = 408,455			h^2^ (%)^§^
				*β*	SE	*P*	*P*^‡^_*(GC)*_	*β*	SE	*P*	
rs11121615	1: 10825577	T/C	0.69	-0.0056	0.0004	7.6e-50	6.0e-48	-0.0070	0.0004	3.0e-76	3.7
rs2911463	16: 88835545	A/G	0.69	-0.0041	0.0004	9.1e-28	9.7e-27	-0.0047	0.0004	2.1e-34	2.0
rs2861819	2: 68489221	C/G	0.66	0.0030	0.0004	7.4e-17	3.0e-16	0.0040	0.0004	9.4e-27	1.2
rs3101725	5: 127524018	C/T	0.76	-0.0030	0.0004	2.9e-13	8.4e-13	-0.0037	0.0004	1.2e-19	0.9
rs11135046	5: 158230013	T/G	0.54	-0.0025	0.0003	3.7e-13	1.1e-12	-0.0032	0.0004	3.1e-19	0.9
rs28558138	4: 26818080	C/G	0.42	-0.0024	0.0004	1.3e-11	3.2e-11	-0.0035	0.0004	3.1e-23	0.8
rs7773004	6: 26267755	G/A	0.49	-0.0023	0.0003	6.4e-11	1.5e-10	-0.0027	0.0004	1.0e-14	0.7
*rs7856039*	*9*: *118385117*	*C/T*	*0*.*67*	*0*.*0024*	*0*.*0004*	*8*.*1e-11*	*1*.*9e-10*	*0*.*0022*	*0*.*0004*	*3*.*3e-09*	*0*.*7*
rs9880192	3: 128297569	C/G	0.41	-0.0023	0.0004	8.7e-11	2.0e-10	-0.0025	0.0004	1.8e-12	0.7
*rs192647746*	*4*: *182760366*	*G/A*	*0*.*001*	*0*.*0333*	*0*.*0052*	*1*.*2e-10*	*2*.*9e-10*	*0*.*0267*	*0*.*0054*	*8*.*8e-07*	*0*.*7*
rs12625547	20: 50154647	G/T	0.17	-0.0029	0.0005	1.3e-10	2.9e-10	-0.0033	0.0005	1.3e-12	0.7
rs236530	17: 68217471	T/C	0.75	-0.0026	0.0004	1.4e-10	3.2e-10	-0.0030	0.0004	9.9e-14	0.7
rs2241173	17: 70028445	G/A	0.58	-0.0022	0.0004	4.4e-10	9.7e-10	-0.0024	0.0004	2.1e-11	0.7
rs73107980	12: 48187601	T/C	0.24	0.0025	0.0004	1.4e-09	2.8e-09	0.0026	0.0004	1.6e-10	0.6

Loci for which *P*-values in the Gene ATLAS dataset were higher than the corresponding *P*-values in the Neale Lab dataset are shown in italics. These loci were not considered in further analysis.

BMI, body mass index; DVT, deep venous thrombosis; EAF, effect allele frequency; SE, standard error; SNP, single nucleotide polymorphism

*Chromosome: position on chromosome according to GRCh37.p13 assembly

^†^Effective allele/reference allele

^‡^*P*-value corrected for the LD Score regression intercept

^§^Proportion of SNP-based heritability explained by the variant

**Fig 2:**
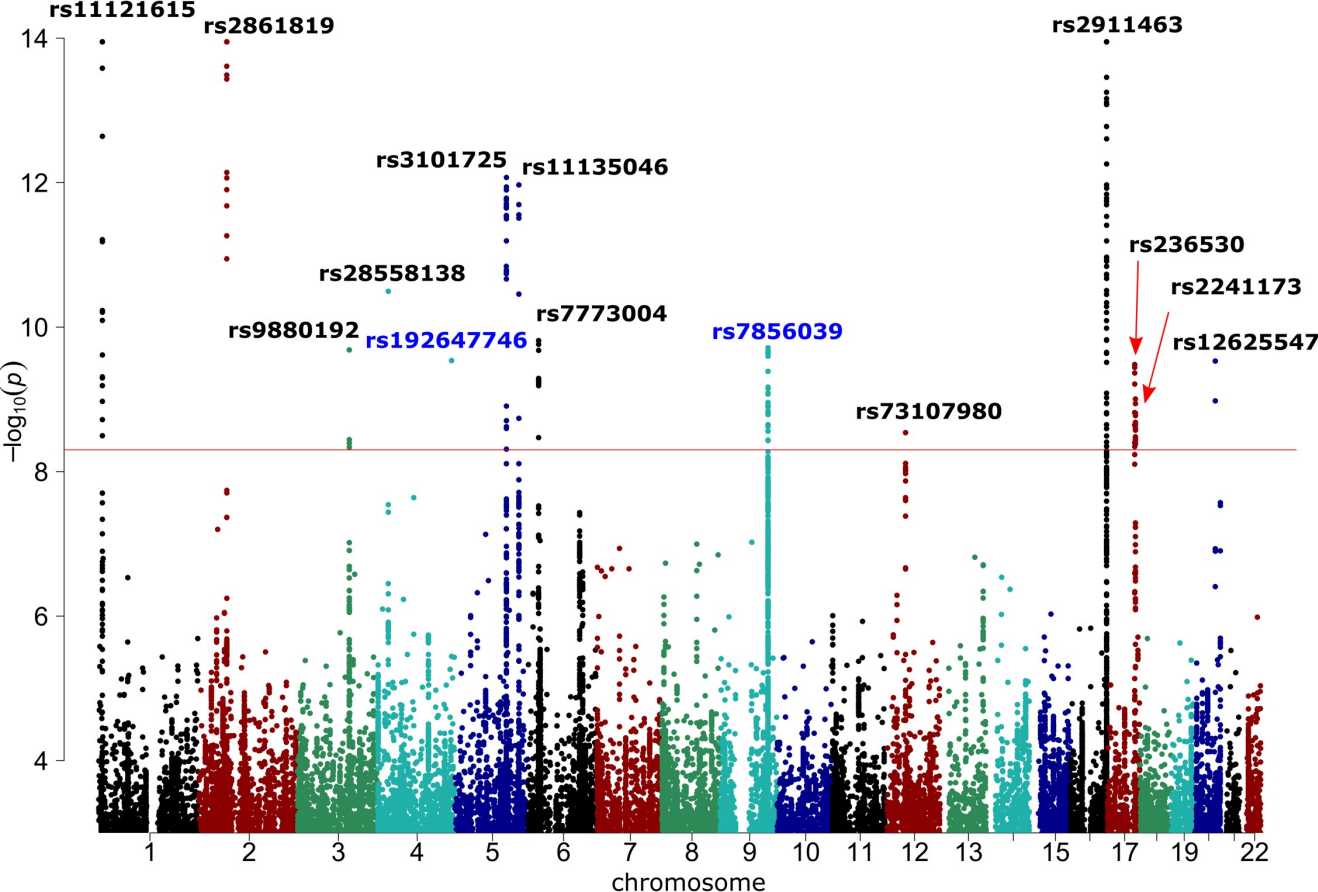
Graphical summary of GWAS results after the adjustment of the Neale Lab data for DVT and BMI and the genomic control correction using LD Score regression intercept. Red line corresponds to the genome-wide significance threshold of *P* = 5.0e-09. Only associations with *P* < 1.0e-03 are presented. Points with -log_10_(*P*) >14 are depicted as points with -log_10_(*P*) = 14. Loci for which *P*-values in the Gene ATLAS dataset were higher than the corresponding *P*-values in the Neale Lab dataset are annotated in blue. These loci were not included in further analyses.

Manhattan plot of–log_10_(*P*) is presented in Fig 2. A quantile-quantile plot for observed vs. expected distribution of *P*-values is shown in S1 Fig. Regional association plots are given in S2 Fig. Genes located in these regions are listed in S2 Table. We performed conditional and joint (COJO) analysis and detected two independent signals in the locus on chromosome 16 at a distance of 39 kb from each other (S3 Table). Lead SNPs rs9972645 and rs2911463 were in low linkage disequilibrium (r^2^ = 0.15).

### Functional annotation of the revealed signals

#### Literature-based annotation

We explored whether genes located in close proximity to identified hits could have biologically plausible roles in VVs based on their function or previously revealed associations with other complex traits. The results of literature-based candidate gene prioritization are provided in S4 Table. Furthermore, we compiled a list of traits associated at a genome-wide significance level with either lead SNPs, or variants in LD with these polymorphisms using the PhenoScanner (S5 Table) and the Pubmed databases. Summary results are given in S4 Table.

Our analysis showed that among the genes nearest to the top GWAS loci (±250 kb), 4 genes were related to vascular development and remodeling (*CASZ1*, *PIEZO1*; putative role in blood vessel remodeling: *STIM2* and *NFATC2*), 4 genes were implicated in blood pressure and hypertension (*CASZ1*, *PIEZO1*, *SLC12A2*, and *EBF1*), 5 genes were linked to immune response/inflammation (*PPP3R1*, *EBF1*, *GATA2*, *NFATC2*, and *RAPGEF3*), and 2 genes were ion channels involved in regulation of both cell volume and vascular tone (*PIEZO1* and *SLC12A2*). One gene (*FBN2*) encoded a protein regulating elastogenesis. In the locus tagged by rs236530, two potassium voltage-gated channels *KCNJ16* and *KCNJ2* were identified, although current evidence does not suggest any mechanistic link of these genes to VVs formation. Interestingly, the only characterized gene in the locus tagged by rs2241173 was *SOX9* –the gene encoding transcription factor required for male sexual development, chondrogenesis/skeletal development and implicated in fibrosis and cancer [19]. Biological functions of this protein are related to extracellular matrix organization, cell proliferation, and epithelial-to-mesenchymal transition. The direct target of SOX9 is the collagen type II alpha 1 chain (*COL2A1*) gene, which is located in another locus revealed in the present study (tagged by rs73107980, 214 kb upstream of the *RAPGEF3* gene). Ectopic *SOX9* expression was demonstrated to cause aberrant expression of extracellular matrix components [20]. It is possible that SOX9 and its targets play currently unknown roles in venous pathophysiology.

Three signals overlapped with findings from a recent GWAS for VVs conducted by “23andMe” company [12]: rs11121615 in the *CASZ1* gene, which was among the top SNPs identified in that study; rs2861819 near the *PPP3R1*, which is in complete LD (D’ = 1.00, r^2^ = 0.98) with their top SNP rs6712038; and rs9972645 in the *PIEZO1* gene, which is in LD (D’ = 0.96, r^2^ = 0.60) with their another hit rs4516218. For 3 loci (tagged by rs2911463, rs3101725, and rs7773004), a genome-wide significant association with red blood cell traits has previously been demonstrated. One of these signals was near the *HFE* gene implicated in iron absorption. Two loci (tagged by rs9880192 and rs12625547) showed genome-wide significant associations with white blood cell count/percentage. Other traits associated with the revealed SNPs were platelet count/crit, allergic disease, hypertension, and anthropometric traits (S4 Table).

Changes in blood pressure, abnormal vascular wall remodeling, altered vascular tone, deterioration of vein wall elastic properties as well as inflammation are known factors related to VVs. Association with red blood cell traits is intriguing. Hypothetically, both erythrocyte homeostasis and VVs could be linked to iron overload [21, 22]. It is noteworthy that association between hemochromatosis-related functional variant in the *HFE* gene and primary VVs was revealed in our previous candidate-gene study in ethnic Russian individuals [21].

#### Searching for functional variants

Ensembl Variant Effect Predictor (VEP) [23] analysis identified a moderate-impact missense variant rs2044693 in the *PNO1* gene (in the locus tagged by rs2861819, r^2^ = 0.90, D’ = 0.97 in Europeans). SIFT and PolyPhen tools predicted no damaging effect of this SNP on the protein function. Furthermore, missense variant rs8043637 was found in the *CTU2* gene (in the locus tagged by rs2911463, r^2^ = 0.12, D’ = 0.66 in Europeans). SIFT tool indicated this SNP as deleterious, and PolyPhen tool–as possibly/probably damaging. Full data are available in S6 Table.

#### Testing for pleiotropy: VVs and gene expression

We used Summary data-based Mendelian Randomization (SMR) analysis followed by the Heterogeneity in Dependent Instruments (HEIDI) test [24] to identify genes whose expression level is associated with the same causal SNPs that affect the risk of VVs. The results are given in S7 Table. SMR/HEIDI analysis provided evidence for pleiotropic effects of variants within rs3101725, rs2861819, and rs2241173 regions. In the first region, association was revealed with the expression of long intergenic non-protein coding RNA 1184 (*LINC01184*) located in close proximity to the *SLC12A2* on the reverse strand. Changes in the *SLC12A2* expression were not associated with VVs in our tests. Interestingly, the previous study showed that disruption of a bi-directional promoter between these genes altered the expression of both *SLC12A2* and *LINC01184*, while deletion of the third exon of *LINC01184* affected only *LINC01184* expression [25].

In loci tagged by rs2861819 and rs2241173, our analysis identified *PPP3R1* gene and the gene encoding long intergenic non-protein coding RNA *AC005152*.*3* with uncharacterized function.

It is important to note that SMR/HEIDI analysis does not distinguish pleiotropy from causality. Therefore, we can hypothesize causal relationships between the expression level of identified genes and the risk of VVs development.

Besides this, we found associations with expression levels of three genes within rs2911463 region (*APRT*, *ZFPM1*, and *RNF166*), one gene within rs2861819 region (*WDR92*) and the *TRIM38* gene located ~280 kb from rs7773004 (S7B Table). However, statistically significant heterogeneity revealed in the HEIDI test indicated that these associations were mediated by polymorphisms different from those that alter VVs risk.

#### DEPICT analysis

Data-driven Expression Prioritized Integration for Complex Traits (DEPICT) framework [26] was used to conduct a gene set and tissue/cell type enrichment analysis as well as to provide additional evidence for gene prioritization. Results of DEPICT analysis for SNP sets associated with VVs at *P* < 5.0e-08 and *P* < 1.0e-05 are given in S8 and S9 Tables, respectively. For the first set, we identified a significant enrichment of the Mammalian Phenotype (MP) Ontology terms “abnormal vasculogenesis” and “abnormal vascular development” (S8A Table). When we relaxed significance threshold of input SNPs to *P* < 1.0e-05, 22 MP terms were shown to be enriched with additional 9 categories related to abnormal morphology of heart and vessels (“abnormal blood vessel morphology”, “abnormal vascular smooth muscle morphology”, “failure of heart looping”, etc., S9A Table). The remaining MP terms were associated with aberrant morphology of other tissues and organs and disruption of embryonic development. Besides this, we observed enrichment for several subnetworks of protein-protein interactions, including that for ENG (major glycoprotein of the vascular endothelium, mutations in its gene cause multisystemic vascular dysplasia), SMAD 2, 4, 6, 7 (transduce signals from TGF-beta family members), and Notch4 (regulates arterial specification [27]).

Tissue/cell type enrichment analysis revealed no statistically significant categories (S8B and S9B Tables).

DEPICT gene prioritization tool provided 2 and 47 prioritized genes depending on the level of statistical significance of the input SNPs (*P* < 5.0e-08 and *P* < 1.0e-05, respectively; S8C and S9C Tables).

#### Summary of gene prioritization

Basing on cumulative evidence from different analyses, we drew up a list of genes which most likely account for the revealed associations and seem to be functionally relevant to VVs development (Table 2). In several cases, the results were ambiguous. For the locus on chromosome 2, we advocate for the *PPP3R1* gene since it displayed a great effect size at a high significance level in the SMR test (*P =* 1.3e-22 and *β* = 120.0, S7 Table). Moreover, biological functions of PPP3R1 suggest its involvement in VVs etiology (Table 2). In particular, it induces MCP-1 production, which was shown to be enhanced in VVs [28, 29]. Finally, a recent study demonstrated that expression of *PPP3R1* is increased in the varicose veins as compared to normal veins (fold change = 1.5) [30]. For the locus on chromosome 16, literature data along with DEPICT analysis strongly suggest the involvement of the *PIEZO1* gene. In the locus by rs2241173, two genes can be prioritized, but only one gene (*SOX9*) has a characterized function. For loci on chromosomes 5 (tagged by rs3101725), 12 (tagged by rs73107980) and 17 (tagged by rs236530), we were unable to prioritize any gene although proposed several candidates.

**Table 2:** Summary of gene prioritization.

Lead SNP	Locus*	Number of genes per locus^†^	Prioritized gene	Nearest gene, *yes/no*	Evidence for prioritization
rs11121615	1: 11075577	4	*CASZ1*	*yes*	L, D
rs2911463	16: 89085545^‡^	20	***PIEZO1***	*yes*	L, D
			*CTU2*	*no*	V
rs2861819	2: 68739221	8	***PPP3R1***	*yes*	L, E, S
			*PNO1*	*no*	V
rs3101725	5: 127774018	3	*SLC12A2*	*yes*	L
			*FBN2*	*no*	L
			*LINC01184*	*no*	S
rs11135046	5: 158480013	1	*EBF1*	*yes*	L
rs28558138	4: 27068080	2	*STIM2*	*no*	L, D
rs7773004	6: 26517755	41	*HFE*	*no*	L
rs9880192	3: 128297569	8	*GATA2*	*no*	L
rs12625547	20: 50154647	4	*NFATC2*	*yes*	L
rs236530	17: 68217471	5	*KCNJ16*	*no*	-
			*KCNJ2*	*yes*	-
rs2241173	17: 70028445	6	***SOX9***	*no*	L
			*AC005152*.*3 (LOC102723505)*	*no*	S
rs73107980	12: 48187601	9	*COL2A1*	*no*	L
			*RAPGEF3 (EPAC1)*	*no*	L

Bold font indicates the genes which, in our opinion, have stronger evidence for prioritization than other candidates in the locus.

D, DEPICT analysis; L, literature-based prioritization; E, expression change in VVs

S, SMR/HEIDI analysis; V, Variant Effect Predictor analysis

*Chromosome: position on chromosome according to GRCh37.p13 assembly

^†^Calculated based on regional association plots generated with LocusZoom tool (http://locuszoom.org/, S2 Fig)

^‡^This locus contains two independent association signals. SNP rs9972645 that tags the second signal is also located in the *PIEZO1* gene

### Testing for pleiotropy: VVs and other complex traits

**Fig 3:**
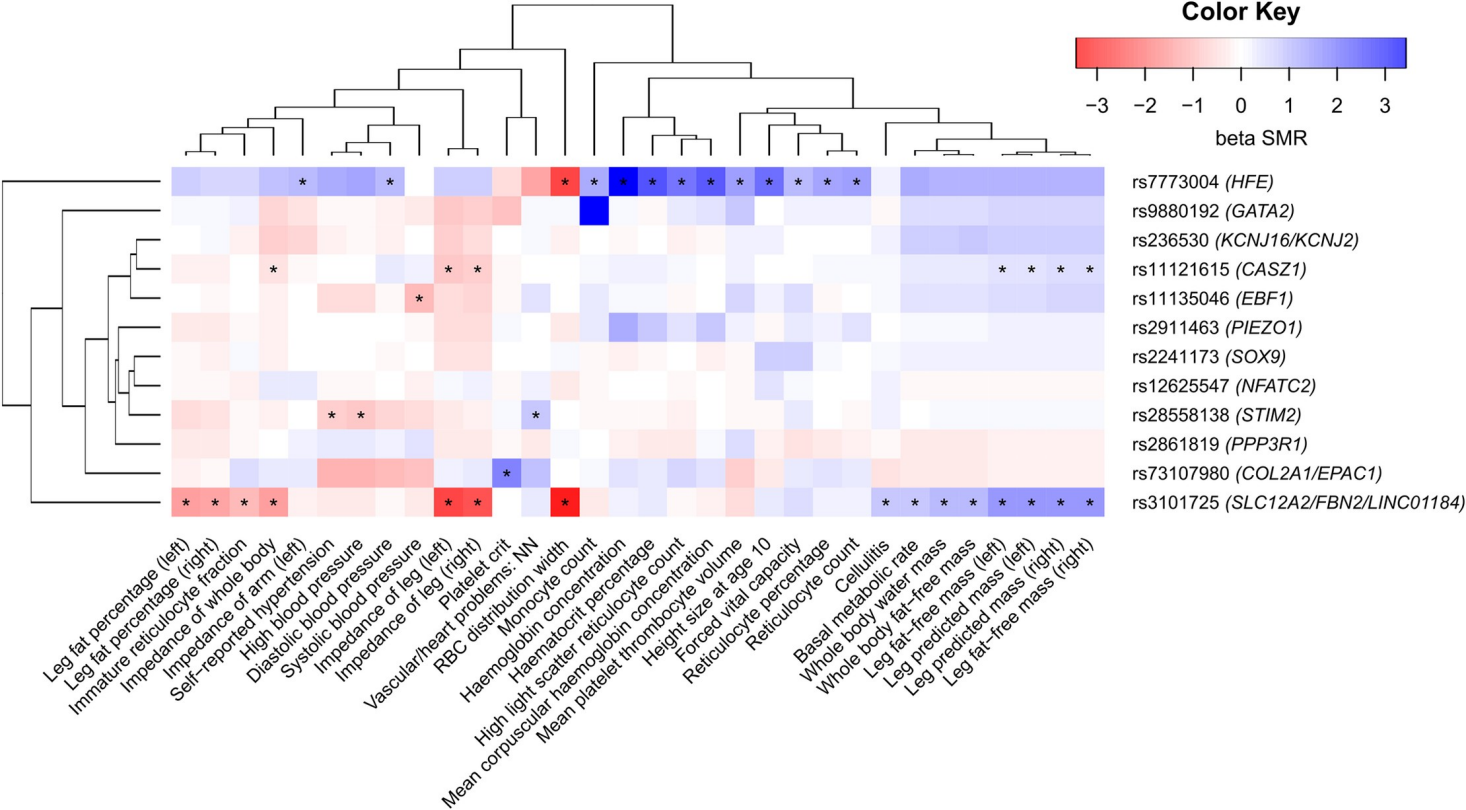
Pleiotropic effects of identified loci on other complex traits. Color depicts the sign and the magnitude of SMR beta coefficient. Negative sign (red) means opposed effects, and positive sign (blue) means the same direction of effect. Traits that passed both SMR and HEIDI test are marked with an asterisk. Prioritized genes in loci are shown in parentheses. Full names of traits as indicated in the Neale lab and the Gene ATLAS databases are given in S11 Table. RBC, red blood cell.

SMR/HEIDI analysis revealed 32 traits that shared the same casual variants with VVs (traits for this and further analyses were obtained from the GWAS-MAP database, see Materials and Methods section and S10 Table for details). Pleiotropic effects were found for 6 out of 12 studied loci (Fig 3, S11 Table). Variants within loci tagged by rs11121615 and rs3101725 showed positive SMR beta coefficients (the same direction of effect) with predicted and fat-free mass of both legs and a whole body as well as with basal metabolic rate, and negative coefficients (opposed effects)–with leg fat percentage as well as impedance of both legs and a whole body (parameter negatively correlated with fat-free mass). Loci tagged by rs3101725 and rs7773004 comprised SNPs with pleiotropic effects on red blood cell erythrocyte distribution width (negative beta). Locus tagged by rs7773004 was also related to numerous blood traits, such as mean corpuscular haemoglobin concentration, mean platelet thrombocyte volume, and monocyte and reticulocyte count. Locus tagged by rs73107980 was associated with platelet crit. Three loci (rs11135046, rs28558138, and rs28558138) were linked to blood pressure and hypertension with different SMR beta signs. Another interesting finding was identification of positive SMR beta for associations with vascular/heart problems (rs28558138 locus), cellulitis (rs3101725 locus), forced viral capacity, and height size at age 10 (rs7773004 locus). Overall, our analysis revealed three main groups of traits: one cluster related to mass, basal metabolic rate, and cellulitis, one cluster of blood-related traits linked only to rs7773004 locus, and one cluster containing the remaining traits (Fig 3).

### Genetic correlations

**Fig 4:**
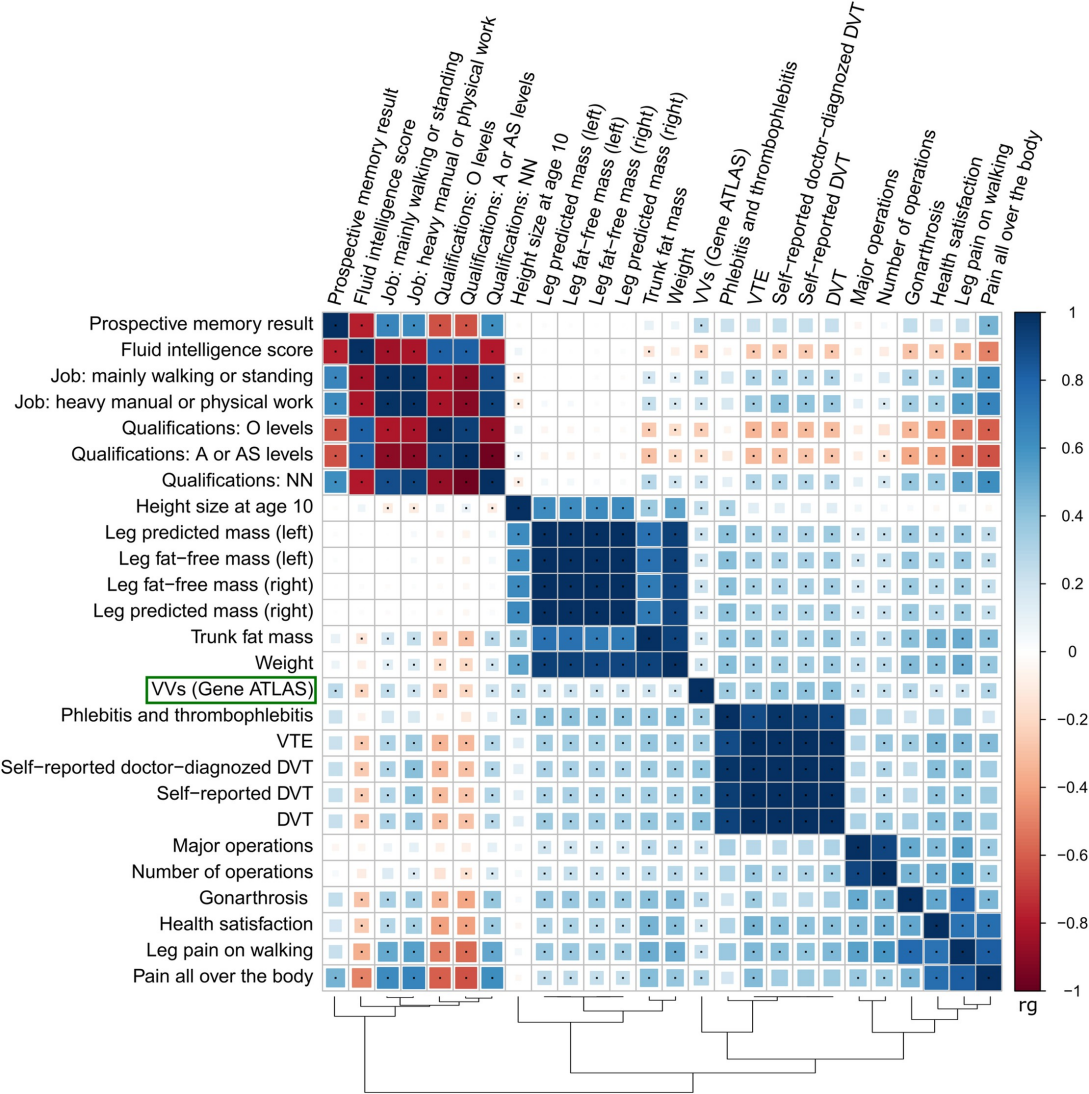
Matrix of genetic correlations between VVs and other complex traits. Color depicts the sign and the absolute value of genetic correlation coefficient (rg). Only the traits having |rg| ≥ 0.2 with VVs are shown. Combinations with statistically significant rg are marked with points (*P* < 3.1e-05). O levels of qualification correspond to the General Certificate of Education Ordinary Level/the General Certificate of Secondary Education; A or AS level correspond to Advanced or Advanced Subsidiary levels. “Qualifications: NN” means no qualification. Full names of traits as indicated in the Neale lab and the Gene ATLAS databases are given in S15 Table. For “Prospective memory result” and “Health satisfaction”, high scores correspond to poor performance. DVT, deep venous thrombosis; VTE, venous thromboembolism.

A list of genetic correlation estimates (rg) between VVs and 861 complex traits is presented in S12 Table. Twenty five traits showed statistically significant correlation with VVs with absolute values of rg ≥ 0.2. Correlation matrix for this subset is displayed as a heatmap in Fig 4. We observed 5 main clusters: traits related to the type of job, intelligence, and qualification; traits related to height and mass (including predicted and fat-free mass of both legs); thrombosis-related traits; traits related to operations; and traits related to pain (including leg pain and gonarthrosis) and health satisfaction. VVs trait was closest to the thrombosis-related cluster (positive correlation). It was also positively correlated with mass, operations, and pain-related traits as well as with lower levels of qualification and heavy manual/walking/standing job. For traits with less prominent correlation with VVs, we observed the same trend: pain and anthropometric traits (sitting and standing height, BMI, mass, etc.) showed positive correlations, whilst higher levels of education–negative ones. Interestingly, negative correlation was observed with usual walking pace, and positive–with current smoking.

We calculated partial genetic correlations for the subset of 7 non-collinear traits with |rg | ≥ 0.2. Two traits–DVT and height size at age 10 –were shown to share common genetic background with VVs after the adjustment for the remaining traits in the analyzed subset (S3 Fig). In other words, their shared genetic components were demonstrated to be at least partially independent of other complex traits. Additionally, we estimated partial genetic correlations between VVs, standing height, and weight. Both standing height and weight had independent genetic components shared with VVs (S4 Fig).

### Hypothesis-free search for causal relationships

**Fig 5:**
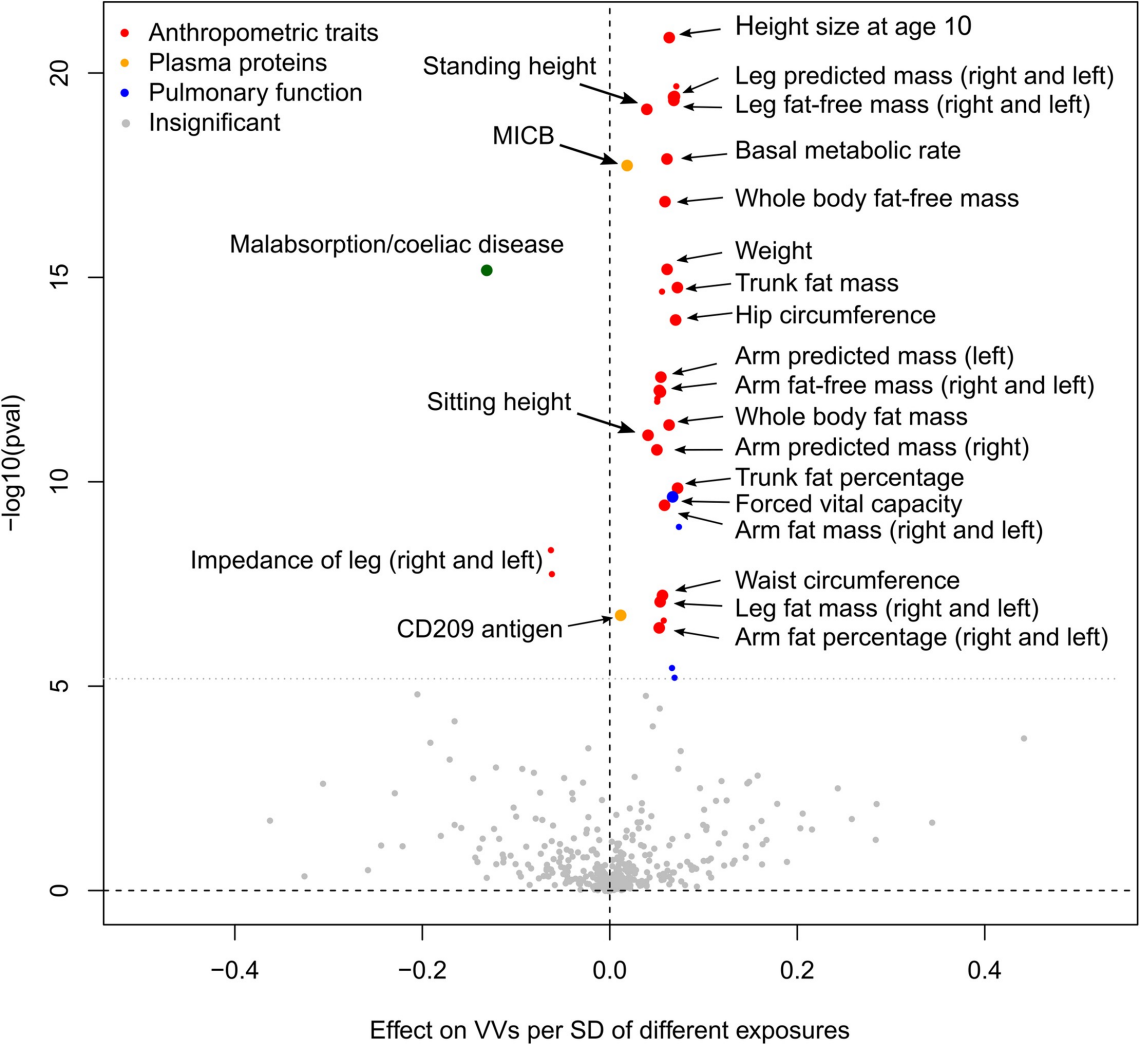
Volcano plot depicting the results of hypothesis-free 2SMR analysis (IVW approach). The x-axis shows 2SMR beta, and the y-axis denotes the level of statistical significance on a logarithmic scale. Grey color indicates the traits that did not pass either 2SMR, or the Steiger test, or additional robustness analysis. Small colored circles depict the traits that passed these tests, but either exhibited heterogeneity in causal effects amongst instruments after removing potential outlier SNPs or did not passed 2SMR after this procedure. Large colored circles represent the traits that both passed 2SMR and showed no heterogeneity after the outliers removal (“the most reliable traits”). 2SMR coefficients are shown as calculated before the correction for pleiotropy. “Pulmonary function” traits include different estimates of forced vital capacity and forced expiratory volume in 1-second. Further details are provided in S13 Table. MICB, MHC class I polypeptide-related sequence B.

We applied a 2-sample Mendelian randomization (2SMR) [31] strategy to infer causal relationships between a broad range of “exposure” phenotypes and VVs as an outcome. In total, 39 complex traits were shown to be potential causative factors. Although only genome-wide associated SNPs from exposure GWAS were selected as instrumental variables, we did not require these loci to be replicated. Nevertheless, we checked the stability of our tests with regard to instruments selection by performing the robustness analysis. This test along with the Steiger test [32] for the correct direction of effect underpinned the exclusion of 2 out of 39 traits (S13 Table). Further, we assessed violations of MR assumption of absence of horizontal pleiotropy (influence of genetic instruments on the outcome only through the exposure, also known as “exclusion-restriction criterion”) by means of sensitivity analyses [31]. We did not observe a statistically significant intercept in MR-Egger regression for any trait. However, only a small proportion of traits showed symmetry in Funnel plots and had no heterogeneity in causal effects amongst instruments. This provides evidence that, for the majority of traits, at least some of the selected instruments exhibit horizontal pleiotropic effects. Such traits mainly involved several hundred genome-wide SNPs that made leave-one-out analysis also uninformative. In order to correct for horizontal pleiotropy, we applied a straightforward approach having excluded all instrumental variables associated with VVs at the level of statistical significance higher than 0.01. Our correction led to symmetry in 26 out of 33 asymmetrical Funnel plots and eliminated heterogeneity in causal effects for 28 out of 34 traits, although 6 traits lost the statistical significance of 2SMR coefficients (S5 Fig, S13 Table). Removing potential sources of heterogeneity also reduced absolute values of 2SMR beta for all the corrected phenotypes. A graphical representation of our results, including 25 causal inferences that we consider the most reliable, is shown in Fig 5. Twenty one traits were related to anthropometry and included standing and sitting height, weight, hip and waist circumference, fat-free, predicted, and fat mass of legs and arms, etc. One trait was a spirometry measurement associated with pulmonary function. Nonetheless, since it is positively correlated with height, we suppose that it has no independent effect on VVs development. Similarly, the reverse association between malabsorption/coeliac disease and VVs could actually be induced by a weight loss as one of the complications of these conditions. Moreover, although this trait has passed all the necessary tests, we avoid making strong claims about its causality since it was self-reported and involved only ~1,500 cases.

All the above mentioned traits were derived from the Neale Lab repository and therefore had about 80% sample overlap with VVs dataset obtained from the Gene ATLAS. Shared participants between the “exposure” and the “outcome” GWAS can cause bias in the Mendelian randomization analysis when weak instruments are selected [33]. In our study, this limitation was mitigated by using only strong instruments associated with exposure traits at a high level of statistical significance (*P* < 1.0e-08). We estimated the relative bias and type 1 error rate inflation using analytical formulae provided by Burgess et al. [33] and a web application (https://sb452.shinyapps.io/overlap/). The relative bias was shown to be small, and the type 1 error was close to nominal: for 80% sample overlap, F-statistics of at least 33.3 (strong instruments selection), sample size of 337,000 and 408,000 for exposure and outcome traits, ordinary least squares estimate of 0.1 and a number of instrumental variables = 600, the relative bias was 2.3%, and the type 1 error– 6.2%. For 300 instruments used for most UK Biobank traits, the relative bias was 2.4%, and the type 1 error– 5.6%. (Supplemental Methods, Section 8). Thus, we considered the application of 2SMR approach suitable for our settings.

Nevertheless, in order to fully overcome the sample overlap problem and confirm our results, we searched for independent GWAS and performed a replication analysis. Data were obtained for height, weight, waist circumference, and body fat percentage. Only height reached Bonferroni-corrected level of statistical significance and remained significant after the correction for pleiotropy (2SMR *P* < 1.0e-07, S14 Table). However, null results for other traits could be explained by a limited power of the analysis. For example, sample size for weight was 4.5 times smaller than in the Neale Lab study.

Finally, the remaining two traits from the “reliable” set were plasma levels of MHC class I polypeptide-related sequence B protein and CD209 antigen. These phenotypes were not UK Biobank traits, therefore our results could not be confounded by a sample overlap.

## Discussion

In the present study, we utilized freely available GWAS summary data to unravel the genetic underpinnings of VVs of lower extremities. We used “quick-and-dirty” statistics provided by the Neale Lab and the Gene ATLAS projects, which aimed to help the scientific community query UK Biobank results for hundreds of human traits avoiding the need to incur high computational costs [34]. These data have been generated without deep insight into each phenotype. Lack of individual-level data made us be as rigorous as possible to avoid false positive discoveries, so we skimmed off the most apparent evidence. Nevertheless, as far as we are aware, our study is the largest and the most comprehensive study of VVs genetics to date, and huge size of the UK Biobank dataset is expected to overcome potential issues related to phenotypic noise. Beyond that, the availability of GWAS results makes our research fully reproducible.

Overall, SNP-heritability on the liability scale was estimated at ~28% assuming the disease prevalence of 20–30%. Using a one-stage GWAS approach, we identified 12 susceptibility loci that explain 13.4% of the SNP-based heritability (Table 1, Fig 2, S2 Table). For each revealed locus, we prioritized the most likely causal genes (Table 2, literature data are given in S4 Table). Nearly 27% of the variance explained by our top 12 SNPs was attributable to the polymorphism in the *CASZ1* gene involved in blood vessel development. This strong signal has previously been revealed by “23andMe” [12] and subsequently replicated in our own sample of ethnic Russian individuals [10]. An especially interesting finding, in our opinion, is an association of VVs with SNPs in a recently discovered *PIEZO1* gene. *PIEZO1* encodes a pressure-activated ion channel which senses shear stress and controls vascular architecture [35]. Mice embryos lacking functional Piezo1 exhibit defects in vascular remodeling and die at midgestation [36]. Other prioritized genes were *PPP3R1*, *EBF1*, *STIM2*, *HFE*, *GATA2*, *NFATC2*, and *SOX9*. The *HFE* gene has been linked to the risk of VVs in our recent candidate-gene study [21]. Meanwhile, we could not prioritize any gene in the loci tagged by rs3101725, rs236530, and rs73107980 (Table 2). On the one hand, region near rs3101725 contains two genes (*SLC12A2* and *FBN2*) that could play a role in varicose transformation, and on the other hand, the causal polymorphism was shown to be eQTL for a nearby non-protein coding RNA *LINC01184* with unknown function. *KCNJ16* and *KCNJ2* genes in the locus tagged by rs236530 encode potassium voltage-gated channels, and their role in vascular biology is unknown. *RAPGEF3* gene in the rs73107980 locus regulates vascular permeability and promotes vascular smooth muscle cell migration, and the transcription of the *COL2A1* gene (encoding an extracellular matrix component) is under direct control of the *SOX9* gene product (prioritized for the rs2241173 locus), so both these genes could potentially be causal.

The revealed genes can be considered as good candidates for future follow-up functional studies. It is noteworthy that *SLC12A2*, *FBN2*, *STIM2*, *HFE*, *KCNJ16*, *KCNJ2*, and *COL2A1* were included in the druggable gene set by Finan et al. [37], and the *PIEZO1* and the *KCNJ2* gene products belong to a “potential drug target” class according to the Human Protein Atlas (https://www.proteinatlas.org/).

Gene set enrichment analysis involving both genome-wide and less strongly associated signals detected gene categories related to abnormal vascular development and morphology (S8A and S9A Tables). This observation is consistent with the hypothesis that pathological changes in the vein wall are the primary event preceding VVs formation [38]. Furthermore, our genetic correlations analysis confirmed known epidemiological associations between VVs and DVT [1, 16] as well as standing job [1, 39, 40] and rough labour [40, 41] (Fig 4, S12 Table). Shared familial susceptibility with venous thromboembolism has already been shown by Zöller et al. [42]. Here we demonstrate that DVT and VVs share specific genetic components which are independent from other factors such as obesity or number of operations (S3 Fig). Since none of the top GWAS hits were related to thrombosis, we can conclude that SNPs with less prominent associations are responsible for this genetic overlap. On the contrary, albeit several identified loci were also associated with blood pressure/hypertension and red and white blood cell traits, including mean corpuscular hemoglobin concentration (Fig 3, S4 and S11 Tables), we found no evidence for genetic correlation between VVs and these traits. We can therefore attribute these effects only to pleiotropy. Intriguingly, we observed small, but significant genetic overlap with smoking (rg = 0.16). Smoking is considered only as suggestive risk factor for VVs since epidemiological studies have mainly shown no association with this habit [1, 39, 41, 43]. Other novel interesting findings include genetic links with prospective memory and fluid intelligence, level of education (negative correlation), pain (knee pain, pain all over the body, neck or shoulder pain, and leg pain on walking), usual walking pace, and gonarthrosis.

Further, we obtained strong evidence for association between VVs and anthropometric traits such as weight, height, waist and hip circumference. We not only observed genetic correlations (Figs 3 and 4, S11 and S12 Tables), but also demonstrated these traits to be causative factors for VVs development (Fig 5, S13 Table). It is important to note that genetic overlap and causal relationships were inferred for both fat and fat-free mass. Thus, we can speculate that increased weight is a risk factor for VVs regardless of whether it was caused by excess body fat or a large mass of other tissues. According to some theories, association between overweight and VVs can be explained by greater concentrations of circulating estrogens or even by a confounding effect of parity [1]. Although this can be true, our results suggest that the cause may also be an increase in mass *per se*. Moreover, we found that height has common genetic component with VVs independent from weight and other traits (S4 Fig) and is also causally related to VVs (Fig 5). Causal inference was additionally confirmed using an independent dataset (S14 Table). This is in agreement with the results of the Edinburgh Vein Study that has shown a significant relationship between increasing height and VVs [39]. However, the majority of epidemiological studies to date have been focused only on BMI and did not consider height or other body characteristics. Notably, the formula for BMI contains the square of height in the denominator. It is possible that the impact of height underlies the inconsistency in the results, when some studies show a positive association between BMI and VVs, while others reveal no effect [1]. We therefore recommend that future epidemiological studies collect and analyze data on height and weight along with data on BMI.

Last but not least, we detected the causal effect of plasma levels of two proteins–MHC class I polypeptide-related sequence B protein (MICB) and CD209 antigen. Both molecules are involved in innate and adaptive immunity. MICB is a ligand for the activating receptor NKG2D present on the surface of natural killer and some other immune-related cells. CD209, also known as DC-SIGN, is a C-type lectin receptor expressed on dendritic cells and macrophages. In our study, Mendelian randomization analysis for CD209 was performed using two genetic instruments–rs505922 and rs8106657 identified by Suhre et al. [44]. The first SNP is located in the *ABO* gene responsible for ABO blood group determination. Allele rs505922 C tags non-O group and is in linkage disequilibrium with the allele A of the neighboring SNP rs507666 (D’ = 1.00, r^2^ = 0.39), which was found to be the top variant associated with VVs in the “23andMe” GWAS [12]. This association was validated in our previous study using data from UK Biobank [10]. At the same time, rs505922 explains nearly 40% of the variability of the plasma CD209 concentration (our estimate based on the published data [44]) with the allele C being linked to higher levels of CD209. It is tempting to speculate that association between the *ABO* gene polymorphisms and VVs as well as between VVs and blood group A [45] is mediated by this protein. We conducted SMR/HEIDI analysis for the locus containing these SNPs and demonstrated that both associations with VVs and CD209 were related to the same causal polymorphism (*P*_SMR_ = 3.3e-06, *P*_HEIDI_ = 0.75), that supports our hypothesis. Nevertheless, the role of CD209 as well as MICB in VVs etiology needs further experimental confirmation. Besides this, it should be taken into account that the level of CD209 in plasma does not necessarily correlates with its level on the surface of the cells, and circulating CD209 may possess its own specific function.

## Materials and methods

### Ethics statement

This study was based on genetic data provided by UK Biobank. All study participants provided written informed consent, and the study was approved by the North West Multi-centre Research Ethics Committee.

### Study sample, genotyping, and quality control

All cases and controls analyzed in our study were derived from UK Biobank [13]. Sociodemographic, physical, lifestyle, and health-related characteristics of UK Biobank participants have been reported elsewhere [15]. In brief, enrolled subjects were aged 40–69 years, were less likely to be obese, to smoke, to drink alcohol, and had fewer self-reported health conditions compared with the general population. Genotyping was performed using the Affymetrix UK BiLEVE and the Affymetrix UK Biobank Axiom arrays. Details on DNA extraction, genotyping, imputation, and quality control (QC) procedures have been reported previously [46].

#### The Neale Lab data

We used genetic association data provided by the Neale Lab (http://www.nealelab.is/) for 337,199 QC positive UK Biobank individuals (6,958 patients diagnosed with “I83: VVs of lower extremities” and 330,241 control individuals without this diagnosis). Data were downloaded on December 15, 2017. Information on data processing is given on the Neale Lab website (http://www.nealelab.is/blog/2017/9/11/details-and-considerations-of-the-uk-biobank-gwas). In short, phenotypes were analyzed automatically by the PHEnome Scan ANalysis Tool. SNPs QC criteria included minor allele frequency (MAF) > 0.1%, the Hardy-Weinberg equilibrium *P* > 1.0e-10, and INFO score > 0.8. After filtration, 10,879,183 autosomal SNPs were left for the analysis. Per-individual QC procedure included removing non-white British and closely related individuals, patients with sex chromosome aneuploidies, and individuals who had withdrawn consent from the UK Biobank study. Associations were adjusted for sex and the first 10 principal components from the UK Biobank sample QC file. We also adjusted the data for BMI and DVT using GWAS summary statistics for these traits downloaded from the same resource (as described previously [17, 18]) and corrected the results for residual inflation using the LD Score regression intercept [47]. Details of adjusting for covariates are given in Supplemental Methods (Section 4).

#### The Gene ATLAS data

We derived summary statistics for 408,455 UK Biobank participants (10,861 patients diagnosed with “I83: VVs of lower extremities” and 397,594 control individuals without this diagnosis) from the Gene ATLAS database (http://geneatlas.roslin.ed.ac.uk/). Data were downloaded on December 8, 2017. Details of this study including QC criteria have been reported previously by Canela-Xandri et al. [14]. In brief, data were obtained for a cohort of both related and unrelated individuals of white British descent; the associations were calculated using Mixed Linear Models with adjustment for age, age^2^, sex, array batch, UK Biobank Assessment Center, and the leading 20 genomic principal components as computed by UK Biobank; individuals with missing or conflicting data, non-biallelic variants, and SNPs with MAF < 0.01% and/or the Hardy-Weinberg equilibrium *P* < 1.0e-50 were excluded. The Gene ATLAS contains information for over 30 million variants. In our analysis, we focused on 10,829,469 SNPs that overlap the Neale Lab SNP set.

### Conditional analysis

Regional association plots were generated with LocusZoom tool (http://locuszoom.org/) for regions within 250 kb from the lead SNP. Conditional and joint (COJO) analysis was carried out by a summary statistics-based method described by Yang et al. [48]. Calculations were performed using the GCTA software [49]. LD (linkage disequilibrium) matrix was computed with PLINK 1.9 software (https://www.cog-genomics.org/plink2) using 1000 Genomes data for 503 European individuals (http://www.internationalgenome.org/data/). We claimed the presence of one independent signal per locus if none of the polymorphisms except the lead SNP passed the significance threshold of *P* = 5.0e-08.

### Literature-based functional annotation

We used regional association plots to identify genes located within associated loci. These genes were further queried for potential involvement in the processes relevant to VVs pathogenesis. For each gene, we scanned an Online Mendelian Inheritance in Man database (OMIM, https://www.omim.org/), the NCBI Gene (https://www.ncbi.nlm.nih.gov/gene), and the Pubmed database to inquire into their biological functions. Furthermore, we interrogated whether other hypothetical varicose-related traits were associated with these genes according to previously published GWAS. The Pubmed search was performed using the gene name as a keyword as well as combinations of the gene name and “GWAS”, or “genome-wide association study”, or “varicose”, or “venous”, or “vascular”. The information obtained was used for the literature-based gene prioritization.

Additionally, we used a PhenoScanner tool [50] to make a list of complex traits associated at *P* < 5.0e-08 with lead SNPs or other polymorphisms in high LD (r^2^ ≥ 0.8) with lead variants (http://www.phenoscanner.medschl.cam.ac.uk/phenoscanner). 1000 Genomes-derived polymorphisms served as proxies for the analysis.

### VEP analysis

For the VEP [23] analysis, we used a set of SNPs in high LD (r^2^ > 0.8) with 13 SNPs identified by the COJO analysis (in a 250kb window). LD proxies were selected from the 1000 Genomes phase 3 version 5 panel using a "proxysnps" R package for European population. Besides this, we selected all SNPs associated with VVs at *P* ≤ T, where log_10_(T) = log_10_(*P*_min_) + 1, and *P*_min_ is a *P*-value for the strongest association per locus (±250 kb from the independent hit). This additional criterion was applied since genotype data for the UK Biobank samples have been imputed using the Haplotype Reference Consortium (HRC) panel, and some HRC SNPs could possibly be missed in the 1000 Genomes panel.

Analysis was performed using software available online (https://www.ensembl.org/info/docs/tools/vep/index.html).

### SMR/HEIDI analysis

SMR/HEIDI approach [24] was used to test for potential pleiotropic effects of identified loci on VVs and other complex traits including gene expression levels in certain tissues. SMR analysis provides evidence for pleiotropy but is unable to define whether both traits are affected by the same underlying causal polymorphism. The latter is specified by a HEIDI test that distinguishes pleiotropy from linkage disequilibrium. Summary statistics for gene expression levels was obtained from Westra Blood eQTL [51] (peripheral blood, http://cnsgenomics.com/software/smr/#eQTLsummarydata) and the GTEx [52] database (44 tissues, https://gtexportal.org). VVs summary statistics was obtained from the Gene ATLAS database. Summary statistics for other complex traits was derived from the GWAS-MAP database (see below).

Nominal *P* for SMR test was set at 3.54e-06 (0.05/14,117, where 14,117 is the total of number of tests corresponding to all analyzed SNPs, probes, and tissues) and 1.88e-06 (0.05/12*2,219, where 12 is the number of loci and 2,219 is the number of non-VVs traits in the GWAS-MAP database excluding binary traits with the number of cases or controls less than 1000). For HEIDI analysis in a gene expression study, we used a conservative threshold of *P* = 0.01 (*P* < 0.01 corresponds to the rejection of pleiotropy hypothesis). For HEIDI in a complex traits analysis, we implemented a less conservative threshold of *P* = 0.001 since the number of independent test was much higher. Details of data processing are given in Supplemental Methods (Section 5).

### DEPICT analysis

DEPICT analysis was performed using DEPICT [26] software version 1.1, release 194 (https://data.broadinstitute.org/mpg/depict/) with default parameters. GWAS summary statistics was obtained from the Gene ATLAS database. We employed DEPICT for both genome-wide significant SNPs (*P* < 5.0e-08) as well as for loci associated with VVs at *P* < 1.0e-05. As in previous analyzes, we defined locus as ±250 kb from the lead SNP. The major histocompatibility complex (MHC) region was eliminated. Significance threshold was set at FDR < 0.05.

### The GWAS-MAP platform

The GWAS-MAP platform integrates a database which was created to study cardiovascular diseases and contains summary-level GWAS results for 123 metabolomics traits, 1,206 circulating proteins, 2,419 complex traits from the UK Biobank, and 8 traits related to coronary artery disease, myocardial infarction and their risk factors. We additionally added 5 VVs-related traits analyzed in the present study (the Gene ATLAS data for VVs; the Neale Lab data for VVs, BMI, and DVT; the Neale Lab data for VVs adjusted for BMI and DVT) as well as 33 traits from the Gene ATLAS database that we supposed to be biologically relevant to VVs. Description of all 3,794 traits is provided in S10 Table. The GWAS-MAP platform contains embedded software for LD Score regression [47], 2-sample Mendelian randomization analysis (MR-Base package [31]), and our implementation of SMR/HEIDI analysis [24]. Further details are given in Supplemental methods (Section 6).

### Genetic correlations

Genetic correlations (rg) between VVs and other complex traits were calculated using LDSC software (https://github.com/bulik/ldsc/). We applied a cross-trait LD Score regression technique as previously described by Bulik-Sullivan et al. [53]. This method requires only GWAS summary statistics and is not biased by a sample overlap. We analyzed 861 heritable non-VVs traits from the GWAS-MAP database. Only traits with a total sample size of ≥ 10,000 and ≥ 1 million SNPs tested were included in the analysis. GWAS summary statistics for VVs was obtained from the Gene ATLAS database. Statistical significance threshold was set at 1.16e-05 (0.01/861). For 25 traits that passed this threshold with |rg| ≥ 0.2, we calculated a matrix of genetic correlations. Partial genetic correlations were estimated using the inverse of the genetic correlation matrix. Significance threshold was set at 3.1e-05 (0.01/325, where 325 is the number of pairwise combinations). For partial genetic correlations between VVs, standing height, and weight, nominal *P* was set at 3.3e-03 (0.01/3). Clustering and visualization were performed by the “corrplot” package for the R language (basic “hclust” function). Further details are provided in Supplemental Methods (Section 3).

### 2SMR analysis

Casual relationships between 2,221 non-VVs phenotypes (“exposures”) from the GWAS-MAP database and VVs (“outcome”) were assessed by 2-sample Mendelian randomization (2SMR) as previously described by the MR-Base collaboration [31] (http://www.mrbase.org/). All the details of our protocol are given in Supplemental Methods (Section 7). Binary traits with the number of cases or controls less than 1000 were not included in the study. GWAS summary statistics for VVs was obtained from the Gene ATLAS database. Analysis was performed on the GWAS-MAP platform. Two 2SMR approaches were used: an inverse variance weighted meta-analysis of Wald ratios (IVW) and MR-Egger regression. The nominal *P* was set at 1.1e-05 (0.05/2*2,221, where 2 is the number of approaches). For traits that passed either IVW or MR-Egger test, we performed the Steiger test [32] for identifying the correct direction of effect, and conducted the robustness analysis (our own approach). Traits that passed all the analyses were then subjected to sensitivity tests [31], that included assessing heterogeneity in causal effects amongst instruments, horizontal pleiotropy test (based on the intercept in MR Egger regression), leave-one-out analysis, and Funnel plots inspection. The nominal *P* for the Steiger test was set at 2.3e-05 (0.05/2,221), and for the robustness analysis, horizontal pleiotropy test, and heterogeneity analysis–at 1.3e-03 (0.05/38, where 38 is the number of traits that passed both 2SMR analysis and the Steiger test). Leave-one-out and Funnel plots were examined manually. Sensitivity analyses revealed the presence of horizontal pleiotropy for the majority of traits. To correct for this confounder, we omitted all instrumental variables associated with VVs with *P* < 0.01, and then repeated IVW 2SMR and sensitivity analyses. Additionally, for the resulting set of traits, we searched for independent GWAS in the MR-Base database [31] and conducted confirmatory IVW 2SMR analysis (where appropriate) with the MR-Base default parameters. The nominal *P* was set at 0.013 (0.05/4, where 4 is the number of traits).

## Supporting information

S1 Text Supplemental methods. (PDF)

S1 Fig A quantile-quantile plot for observed vs. expected distribution of *P*-values for χ^2^ statistics. (PDF)

S2 Fig Regional association plot of–log_10_ (*P*) for SNPs located at the distance of ≤ 250 kb from the index SNP. Color of circles indicates the strength of linkage disequilibrium with the lead SNP based on the squared correlation coefficient (r^2^). Blue line indicates recombination rate (cM/Mb). Genes are indicated as blue bars under the plot.
(PDF)

S3 Fig Matrix of partial genetic correlations between VVs and a subset of non-collinear complex traits. Color depicts the sign and the absolute value of genetic correlation coefficient (rg). Combinations with statistically significant correlations are marked with points. Full names of traits as indicated in the Neale lab and the Gene ATLAS databases are given in S15 Table.
(PDF)

S4 Fig Matrix of partial genetic correlations between VVs, standing height, and weight. Color depicts the sign and the absolute value of genetic correlation coefficient (rg). Combinations with statistically significant correlations are marked with points.
(PDF)

S5 Fig Leave-one-out and Funnel plots before and after removal of instrumental variables associated with VVs with *P* < 0.01. (PDF)

S1 Table Genetic correlations between VVs in different datasets included in the analysis. (DOCX)

S2 Table Top loci associated with VVs of lower extremities. (XLS)

S3 Table Results of conditional and joint analysis. (XLS)

S4 Table Gene prioritization based on a literature review. Protein names are indicated in italics. (DOC)

S5 Table Complex traits associated with the revealed hits (found in a PhenoScanner catalogue). (XLS)

S6 Table Results of the VEP analysis. (XLS)

S7 Table Results of SMR/HEIDI analysis. Searching for pleiotropic effects on VVs and gene expression.
(XLS)

S8 Table Results of DEPICT analysis for SNPs with *P* < 5e-08. (XLS)

S9 Table Results of DEPICT analysis for SNPs with *P* < 1e-05. (XLS)

S10 Table Description of traits included in the GWAS-MAP database. (XLS)

S11 Table Results of SMR/HEIDI analysis. Searching for pleiotropic effects on VVs and other complex traits. Associations that passed both SMR and HEIDI analyses.
(XLS)

S12 Table Genetic correlations between VVs and other complex traits. (XLS)

S13 Table Results of hypothesis-free 2SMR analysis. (XLS)

S14 Table Results of 2SMR analysis performed on independent datasets. (XLS)

S15 Table Trait abbreviations, full names of traits, and their sources (genetic correlations). (XLS)
