# Supplementary material for: Varicose veins of lower extremities: Insights from the first large-scale genetic study
Source: PLoS Genet. 2019 Apr 18;15(4):e1008110. doi: 10.1371/journal.pgen.1008110 (PMC6490943; doi:10.1371/journal.pgen.1008110)
Supplement: S1 Text — (PDF) [file pgen.1008110.s001.pdf]

Supplemental Methods

Contents

Section 1. Summary data used in the analysis 2

Section 2. Variances, covariances, and phenotypic correlations 2

Section 3. Heritability, intercept, and genetic correlations 4

Section 4. Adjustment for covariates 5

Section 5. Testing for pleiotropy using SMR/HEIDI approach 6

Section 6. GWAS-MAP platform 7

Section 7. 2SMR analysis 10

Section 8. Estimation of the relative bias and type 1 error for overlapping samples in 2SMR analysis 13

Section 1. Summary data used in the analysis

Trait name as indicated in the resource	Abbreviation	Resource	Date of data download	Sample size	Number of cases	Trait prevalence	Number of SNPs analyzed	AF reported?
Diagnoses - main ICD10: I83 Varicose veins of lower extremities	I83_NL	The Neale Lab*	December 15, 2017	337,199	6,958	0.021	10,879,183	Yes
I83 Varicose veins of lower extremities	I83_GA	Gene ATLAS†	December 8, 2017	408,455	10,861	0.027	30,795,334	No
Non-cancer illness code_ self-reported: deep venous thrombosis (dvt)	DVT	The Neale Lab*	March 7, 2018	337,159	6,767	0.020	10,879,183	Yes
Body mass index (BMI)	BMI	The Neale Lab*	February 3, 2018	336,107	*N/A*	*N/A*	10,879,183	Yes

AF, allele frequency

*http://www.nealelab.is/blog/2017/7/19/rapid-gwas-of-thousands-of-phenotypes-for-337000-samples-in-the-uk-biobank

†http://geneatlas.roslin.ed.ac.uk/

Both the Neale Lab and the Gene ATLAS projects employed linear (not logistic) regression for binary traits analysis. Data provided by the Neale Lab were adjusted for sex and the first 10 principal components from the UK Biobank sample QC file. Data provided by the Gene ATLAS were adjusted for age, age^2, sex, array batch, UK Biobank assessment center, and the leading 20 genomic principal components as computed by UK Biobank.

Section 2. Variances, covariances, and phenotypic correlations

Variances of quantitative traits were estimated as follows:

1) The list of "pruned" SNPs was generated which contained 535,775 polymorphisms in low linkage disequilibrium (LD) with each other. To create this list, we used 1000 Genomes Project reference panel (1000G) of 268 European individuals. Data were processed using PLINK 1.9 software (https://www.cog-genomics.org/plink2) with options: "--maf 0.1" and "--indep-pairwise 500kb 500 0.5". Then, we searched for overlap between this list and SNPs from the GWAS for the trait of interest. SNPs presented in both lists were additionally filtered by imputation quality > 0.7, minor allele frequency (MAF) > 0.05, and *P*-value > 0.1. The final set contained around 200,000 SNPs.
2) Variance of each genotype g was approximated under the assumption of the Hardy-Weinberg equilibrium (HWE) as $var(g) = 2p(1-p)$, where p is the allele frequency.

3) Variance of the trait y was estimated as $var(y) = median(vy)$, where vy is a vector $vy = SE^2 \times N_{total} \times var(g)$, SE is a standard error of β, and N_{total} is a sample size.

Variances of binary (case/control) traits y was estimated as $var(y) = prev \times (1 - prev)$, where $prev$ is the prevalence of the trait. Besides this, we used the method described above to calculate the variance and observed high concordance between the results (the difference was in the fourth decimal place).

Phenotypic correlations (hereinafter referred to as **correlations**) and **covariances** between the traits were estimated by the method reported by Stephens et al.[1]

The following procedure was implemented:

1) Searching for overlap between SNPs from GWAS for both traits and the list of "pruned" SNPs.
2) Filtering of these SNPs by imputation quality > 0.7, MAF > 0.05, and *P*-value > 0.1.
3) Matching the alleles. If the effect allele in the first GWAS was the reference allele in the second GWAS, we "flipped" allele in the second GWAS changing the beta sign.
4) Correlations between the traits were estimated as correlations between vectors of Z-statistics obtained from GWAS for each trait ($Z = \beta / SE$).

Covariances between the traits were estimated as follows:

$cov(y_1, y_2) = \sqrt{var(y_1) \times var(y_2)} \times cor(Z_1, Z_2)$, where $cor(Z_1, Z_2)$ is the correlation between traits y_1 and y_2.

REFERENCES:

1. Stephens, M. A unified framework for association analysis with multiple related phenotypes. *PLoS One* **8**, e65245 (2013). doi:10.1371/journal.pone.0065245

<u>Section 3.</u> Heritability, intercept, and genetic correlations

SNP-captured **heritability (h^2)** was estimated using LDSC (LD SCore) software (https://github.com/bulik/ldsc/). Summary statistics was filtered using LDSC default criteria of $r^2 > 0.9$, MAF > 0.01, and the overlap with the list of 1,215,001 common HapMap3 SNPs with high imputation quality. The MHC region was excluded. We further used this SNP set to analyze **genetic correlations** as well as to calculate the **intercept** by methods described by Bulik-Sullivan et al.[1,2] The intercept was further used to correct GWAS results for residual inflation.

Genetic correlations between VVs and other complex traits were assessed based on I83_GA. First, we performed a hypothesis-free scan of correlations between VVs and 861 non-VVs traits from the GWAS-MAP database (see below) that met the following criteria: (1) total sample size ≥ 10,000, (2) number of SNPs ≥ 1 million, (3) $h^2/SE \geq 2$, (4) $0 < h^2 < 1$. Statistical significance threshold was set at 1.16e-05 (Bonferroni-corrected P = 0.01/861). Twenty five traits that passed this threshold and had absolute values of genetic correlation coefficients ≥ 0.2 were selected for further analysis. For this final set of traits, we calculated a matrix of genetic correlations and carried out clustering and visualization by the "corrplot" package for the R language (basic "hclust" function). Ward's clustering method was used, and squared Euclidean distances were estimated by subtracting absolute values of genetic correlations from 1. Significance threshold was set at 3.1e-05 (0.01/325, where 325 is the number of pairwise combinations).

Partial genetic correlations ρ_{ij} were evaluated for the subset of non-collinear traits using the inverse of the genetic correlation matrix. The following equation was used:

$$\rho_{ij} = \frac{-p_{ij}}{\sqrt{p_{ii} \times p_{jj}}}$$

where p is the element of the inverted genetic correlation matrix. Traits for partial generic correlations analysis were selected based on the iterative procedure: if two traits had absolute values of genetic correlation coefficients > 0.7, only that trait was selected which had the highest absolute value of genetic correlation with VVs. Statistical significance threshold was set at 3.1e-05. Additionally, we calculated partial genetic correlations between VVs, standing height, and weight. Nominal P was set at 3.3e-03 (0.01/3).

REFERENCES:

1. Bulik-Sullivan, B. K. et al. An atlas of genetic correlations across human diseases and traits. *Nat. Genet.* **47**, 1236–1241 (2015). doi:10.1038/ng.3406
2. Bulik-Sullivan, B. K. et al. LD Score regression distinguishes confounding from polygenicity in genome-wide association studies. *Nat. Genet.* **47**, 291–5 (2015). doi:10.1038/ng.3211

Section 4. Adjustment for covariates

We performed a summary-level adjustment of the studied trait (I83_NL) for BMI and DVT using summary statistics derived from the Neale Lab database. This method of adjustment is robust if there is a full sample overlap between GWAS of interest, and UK Biobank is exactly the case. Our approach was first tested and described in our previous study[1].

In short, this method implements the following idea. Consider a regression model:

$$y = Xb + e \quad (1)$$

where *y* is *n* × 1 vector of phenotypes; *n* is a sample size; *X* is the design *n* x (*k*+1) matrix, which can be partitioned into the column vector of genotype *g* and the submatrix of *k* covariates *C*, *X*=[*g*|*C*]; *b* is a vector of joint effects; and *e* is a residual noise.

This problem has a well-known least squares solution:

$$b = (X'X)^{-1}X'y \text{ and } var(b) = \sigma_j^2(X'X)^{-1} \text{ (2)}$$

where σ_j^2 is the residual variance of *y*; $X'X = M \times (n - k - 2)$; *M* is the covariance matrix of *X*; $X'y = cov(X, y) \times (n - 1)$; $cov(X, y)$is the vector of covariances between the trait *y* and all predictors *X*.

Thus, the solution (2) can be rewritten in terms of the covariance matrix as follows:

$$b = M^{-1} \times cov(X, y) \times \frac{n-1}{n-k-2};\ \sigma_j^2 = var(y) - b \times cov(X, y) \text{ and } var(b) = diag\left(\frac{\sigma_j^2 * M^{-1}}{n-k-2}\right) \quad (3)$$

X and *y* are not necessarily centered.

Thus, if we know $cov(X, y)$ and the covariance matrix M, we have a solution to the problem in Eq. (1). Since we have access to the results of univariate GWAS for all traits, we can estimate and/or approximate M and $cov(X, y)$ through the estimation of covariances between the genotype and each trait, the covariance between the traits, the variance of the genotype, and variances of traits.

The covariance between the genotype g and the trait of interest C (or y) can be estimated using the solution:

$$cov(g, C_i) = \beta_{g,C} \times var(g) \quad (4)$$

where $\beta_{g,C}$ is the effect of the genotype g on the trait C from a univariate GWAS. The covariance matrix M, the variance of the genotype, and variances of traits can be estimated as described above. This gives us the solution to the problem (3).

REFERENCES:

1. Tsepilov, Y. A. et al. A network-based conditional genetic association analysis of the human metabolome. Gigascience **7**, 1-11 (2018). doi: 10.1093/gigascience/giy137

<u>Section 5.</u> Testing for pleiotropy using SMR/HEIDI approach

SMR/HEIDI analysis was conducted as described by Zhu et al.[1] HEIDI statistics was calculated as $T_{HEIDI} = \sum_i^m z_{d(i)}^2$, where m is the number of SNPs selected for analysis, $z_{d(i)} = {d_i}/{SE_{(d_i)}}$ and $d_i = \beta_{SMR_i} - \beta_{SMR\ (lead\ SNP)}$.

SNP selection was performed as follows:

1) We defined a set of eligible markers within ±250 kb from the lead SNP in the primary GWAS, which had $\chi^2 > 10$ in the primary GWAS, and for which the results were reported in the secondary GWAS;
2) Made empty "target" and "rejected" SNP sets;
3) Selected SNP from the primary GWAS with the lowest *P*;
4) If this SNP had $r^2 > 0.9$ with any SNP in the target SNP set, we added it to the "rejected" set. LD matrix (r^2) was computed with PLINK 1.9 (https://www.cog-genomics.org/plink2) using 1000 Genomes data for 503 European individuals (http://www.internationalgenome.org/data/);
5) Otherwise, it was added to the "target" set;

6) Procedure was repeated from the step 3) until either eligible SNP set was exhausted, or the "target" set had 20 SNPs. If we could not select 3 or more SNPs, no test was performed.

GWAS summary statistics for VVs was obtained from the Gene ATLAS database (I83_GA). When testing for pleiotropy with complex traits, we standardized all SNP effects (β) and standard errors (SE): $\breve{\beta}_{Y_i} = \beta_{Y_i}/SD_{Y_i}$ and $\breve{SE}_{Y_i} = SE_{Y_i}/SD_{Y_i}$, where $\breve{\beta}_{Y_i}$ and $\breve{SE}_{Y_i}$ are standardized betas and standard errors for the trait Y_i; β_{Y_i} and SE_{Y_i} are original betas and standard errors for the trait Y_i; $SD_{Y_i}$is a square root of estimated variance of the trait Y_i.

Analysis was conducted using Python 3.5 as the main programming language.

REFERENCES:

1. Zhu, Z. et al. Integration of summary data from GWAS and eQTL studies predicts complex trait gene targets. *Nat. Genet.* **48**, 481–7 (2016). doi:10.1038/ng.3538

Section 6. GWAS-MAP platform

Our platform integrates a database of summary-level GWAS results which was originally created to study cardiovascular disease (CVD). We have collected GWAS summary statistics for different complex traits (including those related to coronary artery disease) as well as for levels of circulating proteins and metabolites. We additionally added 5 traits analyzed in the present study and 33 traits from the Gene ATLAS database that we supposed to be biologically relevant to VVs. A brief description of data is given in the Table below. All 3,794 traits are listed in Table S10.

SNPs from each GWAS were matched with polymorphisms from 1000 Genomes Project Phase 3 v5 reference panel. SNPs with conflicting data on rs id, position, and alleles were excluded. For SNPs that have passed this filtering, alleles were harmonized across all GWAS and sorted in a lexicographic order. In total, our database comprises ~28 billion SNP-trait associations.

GWAS summary statistics is stored using the ClickHouse database management system (https://clickhouse.yandex/). For each trait, we created an annotation file that contains information about the study design and key characteristics of association analysis (name of the cohort, sample size, model of inheritance, trait transformations, reference population, etc.). Metadata is organized with the PostgreSQL database system (https://www.postgresql.org/).

Besides a GWAS database, our platform contains embedded software for LD Score regression[12], MR-Base[13,14], and our implementation of SMR/HEIDI analysis[15] (see above).

Data included in the GWAS-MAP database.

Dataset/source	Number of traits	Description		Reference
The Neale Lab	2,419	Complex traits from the UK Biobank		http://www.nealelab.is/
"Metabolomics_NMR"	123	Circulating metabolites quantified with the NMR metabolomics platform (University Hospitals of Strasbourg, France)		[1]
"Protein biomarkers_Olink"	82	Plasma proteins considered relevant to cardiovascular disease measured with the ProSeek CVD array I (Olink Biosciences, Sweden)		[2]
"Proteomics_SOMAscan"	1,124	Blood circulating proteins measured with the SOMAscan platform (SomaLogic Inc., USA)		[3]
"CAD_traits"	8	Coronary artery disease-related traits	Coronary artery disease	[4]
			Coronary artery disease	[5]
			Myocardial infarction	[6]
			Fasting glucose	[7]
			Cigarettes smoked per day	[8]
			Body mass index	[9]
			Waist-hip ratio	[10]
			Educational attainment	[11]
Additionally added				
"Varicose-related traits_our study"	5	The Neale Lab data for VVs (I83_NL), BMI, and DVT;<br>The Gene ATLAS data for VVs (I83_GA);<br>The Neale Lab data for VVs adjusted for BMI and DVT		*N/A*
"Varicose-related traits_Gene ATLAS"	33	The Gene ATLAS data for traits that we hypothesized to be relevant to VVs		http://geneatlas.roslin.ed.ac.uk/

REFERENCES:

1. Kettunen, J. *et al.* Genome-wide study for circulating metabolites identifies 62 loci and reveals novel systemic effects of LPA. *Nat. Commun.* **7,** 11122 (2016). doi:10.1038/ncomms11122
2. Folkersen, L. et al. Mapping of 79 loci for 83 plasma protein biomarkers in cardiovascular disease. *PLOS Genet.* **13**, e1006706 (2017). doi:10.1371/journal.pgen.1006706
3. Suhre, K. et al. Connecting genetic risk to disease end points through the human blood plasma proteome. *Nat. Commun.* **8**, 14357 (2017). doi:10.1038/ncomms14357

4. Schunkert, H. et al. Large-scale association analysis identifies 13 new susceptibility loci for coronary artery disease. *Nat. Genet.* **43**, 333–338 (2011). doi:10.1038/ng.784
5. Howson, J. M. M. *et al.* Fifteen new risk loci for coronary artery disease highlight arterial-wall-specific mechanisms. *Nat. Genet.* **49,** 1113–1119 (2017). doi:10.1038/ng.3874
6. Nikpay, M. *et al.* A comprehensive 1000 Genomes–based genome-wide association meta-analysis of coronary artery disease. *Nat. Genet.* **47,** 1121–1130 (2015). doi:10.1038/ng.3396
7. Dupuis, J. *et al.* New genetic loci implicated in fasting glucose homeostasis and their impact on type 2 diabetes risk. *Nat. Genet.* **42,** 105–116 (2010). doi:10.1038/ng.520
8. The Tobacco and Genetics Consortium. Genome-wide meta-analyses identify multiple loci associated with smoking behavior. *Nat. Genet.* **42,** 441–447 (2010). doi:10.1038/ng.571
9. Locke, A. E. *et al.* Genetic studies of body mass index yield new insights for obesity biology. *Nature* **518,** 197–206 (2015). doi:10.1038/nature1417
10. Shungin, D. *et al.* New genetic loci link adipose and insulin biology to body fat distribution. *Nature* **518,** 187–196 (2015). doi:10.1038/nature14132
11. Okbay, A. *et al.* Genome-wide association study identifies 74 loci associated with educational attainment. *Nature* **533,** 539–542 (2016). doi:10.1038/nature17671
12. Bulik-Sullivan, B. K. *et al.* LD Score regression distinguishes confounding from polygenicity in genome-wide association studies. *Nat. Genet.* **47,** 291–5 (2015). doi:10.1038/ng.3211
13. Hemani, G. *et al.* MR-Base: a platform for systematic causal inference across the phenome using billions of genetic associations. *bioRxiv* **078972** (2016). doi:10.1101/078972
14. Hemani, G. *et al.* The MR-Base platform supports systematic causal inference across the human phenome. *Elife* **7,** e34408 (2018). doi:10.7554/eLife.34408
15. Zhu, Z. et al. Integration of summary data from GWAS and eQTL studies predicts complex trait gene targets. *Nat. Genet.* **48**, 481–7 (2016). doi:10.1038/ng.3538

Section 7. 2SMR analysis

Two-sample Mendelian randomization (2SMR) was performed as previously described by the MR-Base collaboration[1,2] (http://www.mrbase.org/). We implemented two approaches: an inverse variance weighted (IVW) meta-analysis of Wald ratios and MR-Egger regression. "Exposure" phenotypes were obtained from the GWAS-MAP database, VVs ("outcome") phenotype was derived from the Gene ATLAS database (I83_GA), and the overall analysis was conducted on the GWAS-MAP platform. Binary traits with the number of cases or controls less than 1000 were excluded. In our opinion, 2SMR results for such traits would not be reliable, and their interpretation could be made only with caution.

Instrumental variables (SNPs) from "exposure" GWAS were selected using the following criteria: (1) MAF > 0.1, (2) *P* < 1.0e-08 for traits obtained from the Neale Lab and the Gene ATLAS, and *P* < 5.0e-08 for the remaining phenotypes. We applied more rigorous threshold for the UK Biobank traits in order to mitigate the limitation arising from 80% (the Neale Lab) or 100% (Gene ATLAS) sample overlap with I83_GA. Overlapping samples can provide bias in Mendelian randomization analyses in the case of weak instruments selection, and this bias is directed towards the observational association between the exposure and the outcome[3]. Our instruments were therefore restricted to more strongly associated loci. Estimation of magnitude of the relative bias and type 1 error rate inflation for different sample overlap proportions, number of instrumental variables, and 2SMR beta coefficients assuming strong instruments is provided in Section 8.

We extracted only one lead (the most significant) SNP per locus. Locus was defined as ± 1000 kb from lead SNP.

- If less than two instruments could be extracted, the trait was omitted from the analysis.
- If the number of SNPs was equal to two, we used only IVW approach.
- If we could extract more than two SNPs, we used both IVW and MR-Egger approaches.

SNP effects (β) and standard errors were standardized as follows: $\breve{\beta}_{Y_i} = \beta_{Y_i}/SD_{Y_i}$ and $\breve{SE}_{Y_i} = SE_{Y_i}/SD_{Y_i}$, where $\breve{\beta}_{Y_i}$ and $\breve{SE}_{Y_i}$ are standardized betas and standard errors for the trait Y_i; β_{Y_i} and SE_{Y_i} are original betas and standard errors for the trait Y_i; SD_{Y_i} is a square root of estimated variance of the trait Y_i.

The nominal *P* for 2SMR analysis was set at 1.1e-05 (0.05/2*2,221, where 2 is the number of approaches, and 2,221 is the number of non-VVs traits in the GWAS-MAP database that met our inclusion criteria). For traits that passed either IVW or MR-Egger test, we performed **the Steiger test**[4]. Significance threshold was set at 2.3e-05 (0.05/2,221). Traits that passed the Steiger test were further subjected to **the robustness analysis**. It was conducted as follows:

1) We relaxed significance threshold for instrumental variables to 1.0e-05;
2) Selected up to 6 sets of up to 10, 20, 40, 60, 80, and 100 (where appropriate) independent top SNPs with MAF > 0.1 from "exposure" GWAS (for example, if we had only 35 hits, we made 3 sets of 10, 20, and 35 SNPs);
3) Performed 2SMR for each set using both IVW and MR-Egger approaches;
4) Estimated heterogeneity in 2SMR coefficients (calculated in all abovementioned 2SMR tests including the initial test with the threshold of $P < 5.0\text{e-}08$ for SNP selection) by Cochran's Q test.
5) Calculated χ^2 for each analysis in step 3 as $\chi 2 = ({}^{\beta_{SMR}}/_{SE_{\beta_{SMR}}})^2$
6) Calculated P of the robustness analysis as the sum of χ^2: $P = \sum_{i=1}^{6} \chi 2$
   This variable was distributed as χ^2 with six or less degrees of freedom depending on the maximal number of top SNP sets.

Results of 2SMR analyses were considered robust if:

- P in the robustness analysis (either for IVW or for the MR-Egger approach) was less than 1.3e-03 (0.05/38, where 38 is the number of traits that passed both 2SMR analysis and the Steiger test);
- Heterogeneity estimated in step 4 (for both IVW and the MR-Egger approaches) was statistically insignificant ($P \geq 1.3\text{e-}03$).

For traits that showed robust 2SMR results, we conducted **sensitivity analyses**[1,2] (examination of potential violations of MR assumptions):

- Assessing heterogeneity in causal effects amongst instruments by Cochran's Q test (significance threshold was set at 1.3e-03);
- Horizontal pleiotropy test (estimation of the intercept in MR Egger regression; significance threshold was set 1.3e-03);
- Leave-one-out analysis (does leaving a single SNP lead to a dramatic change in 2SMR beta? YES/NO/Impossible to estimate);
- Funnel plots assessment (are plots symmetrical? YES/NO/Impossible to estimate).

Leave-one-out and funnel plots were inspected manually.

The majority of revealed traits showed statistically significant heterogeneity in causal effects amongst instruments as well as Funnel plots asymmetry, that indicates the presence of horizontal pleiotropy. This problem can potentially be overcome by identification and selective removal of outliers

exhibiting pleiotropic effects – for example, via calculation of Cook's distances or performing the MR-PRESSO outlier test[5]. In our study, we applied more straightforward approach to correct for horizontal pleiotropy: we omitted all instrumental variables associated with the outcome with $P < 0.01$, and then repeated IVW 2SMR and sensitivity analyses.

Finally, for the resulting set of traits, we used the MR-Base platform[1,2] to search for independent GWAS performed on samples other than UK Biobank, and conducted additional 2SMR analysis with the MR-Base default parameters (summary statistics for VVs was also derived from I83_GA). Our aim was to completely avoid sample overlap and confirm the beta sign and the statistical significance of the results. We selected genome-wide SNPs that overlap with SNPs from I83_GA and have MAF > 0.05. The nominal *P* for 2SMR, heterogeneity in causal effects amongst instruments analysis as well as for the Steiger test was set at 0.013 (0.05/4, where 4 is the number of traits). Eventually, since heterogeneity was also significant, we applied the abovementioned approach to remove instruments with potential pleiotropic effects, and recalculated the data.

REFERENCES:

1. Hemani, G. *et al.* MR-Base: a platform for systematic causal inference across the phenome using billions of genetic associations. *bioRxiv* **078972** (2016). doi:10.1101/078972
2. Hemani, G. *et al.* The MR-Base platform supports systematic causal inference across the human phenome. *Elife* **7,** e34408 (2018). doi:10.7554/eLife.34408
3. Burgess, S., Davies, N. M. & Thompson, S. G. Bias due to participant overlap in two-sample Mendelian randomization. *Genet. Epidemiol.* **40,** 597–608 (2016). doi:10.1002/gepi.21998
4. Hemani, G., Tilling, K. & Davey Smith, G. Orienting the causal relationship between imprecisely measured traits using GWAS summary data. *PLOS Genet.* **13,** e1007081 (2017). doi:10.1371/journal.pgen.1007081
5. Verbanck, M., Chen, C.-Y., Neale, B. & Do, R. Detection of widespread horizontal pleiotropy in causal relationships inferred from Mendelian randomization between complex traits and diseases. *Nat. Genet.* **50,** 693–698 (2018). doi:10.1038/s41588-018-0099-7

Section 8. Estimation of the relative bias and type 1 error for overlapping samples in 2SMR analysis

Effects of different parameters on bias in instrumental variables (IV)-based studies have been assessed in a variety of previous works[1-8].

For 2SMR, it has been proven that if the main IV assumptions* are satisfied, the relative bias depends on (1) the proportion of overlap between the samples, (2) the strength of association between IV and exposure, (3) the strength of association of confounders with the exposure and the outcome.

*main MR assumptions:
(IV1) the instrument is associated with the exposure;
(IV2) the instrument does not influence the outcome through some pathway other than the exposure;
(IV3) the instrument does not associate with confounders[9].

We estimated the relative bias and type 1 error rate in 2SMR analysis assuming settings used in the present study for the UK Biobank traits:

- strong instruments: expected F-statistics ≥ 33.3 (corresponds to $P < 1.0e\text{-}08$ of the association between IV and the exposure trait, see Section 7)
- N = 337,000 or 408,000 for exposure traits and 408,000 for and the outcome trait
- Coefficient of determination of risk factor on genetic variants: R^2 = (F-statistics/N)*k, where N is the sample size of the exposure trait, k – number of instrumental variables

Calculation were performed using web application (https://sb452.shinyapps.io/overlap/):

OLS *	N instruments	Overlap proportion, %	Bias	Relative bias	Type 1 error rate
**0.025**	10	80	0.0006085	2.43%	5.00%
		100	0.0007606	3.04%	5.00%
	50	80	0.0006066	2.43%	5.01%
		100	0.0007583	3.03%	5.01%
	100	80	0.0006042	2.42%	5.01%
		100	0.0007553	3.02%	5.02%
	200	80	0.0005995	2.40%	5.03%
		100	0.0007494	3.00%	5.04%
	300	80	0.0005947	2.38%	5.04%
		100	0.0007434	2.97%	5.06%
	400	80	0.00059	2.36%	5.05%
		100	0.0007375	2.95%	5.08%
	500	80	0.0005852	2.34%	5.06%
		100	0.0007315	2.93%	5.10%

	600	80	0.0005804	2.32%	5.08%
		100	0.0007255	2.90%	5.12%
**0.05**	10	80	0.001217	2.43%	5.01%
		100	0.001521	3.04%	5.01%
	50	80	0.001213	2.43%	5.03%
		100	0.001517	3.03%	5.04%
	100	80	0.001208	2.42%	5.05%
		100	0.001511	3.02%	5.09%
	200	80	0.001199	2.40%	5.11%
		100	0.001499	3.00%	5.17%
	300	80	0.001189	2.38%	5.16%
		100	0.001487	2.97%	5.25%
	400	80	0.00118	2.36%	5.21%
		100	0.001475	2.95%	5.33%
	500	80	0.00117	2.34%	5.26%
		100	0.001463	2.93%	5.40%
	600	80	0.001161	2.32%	5.30%
		100	0.001451	2.90%	5.48%
**0.1**	10	80	0.002434	2.43%	5.02%
		100	0.003043	3.04%	5.03%
	50	80	0.002426	2.43%	5.11%
		100	0.003033	3.03%	5.17%
	100	80	0.002417	2.42%	5.22%
		100	0.003021	3.02%	5.34%
	200	80	0.002398	2.40%	5.43%
		100	0.002997	3.00%	5.68%
	300	80	0.002379	2.38%	5.64%
		100	0.002974	2.97%	6.00%
	400	80	0.00236	2.36%	5.84%
		100	0.00295	2.95%	6.32%
	500	80	0.002341	2.34%	6.04%
		100	0.002926	2.93%	6.63%
	600	80	0.002322	2.32%	6.23%
		100	0.002902	2.90%	6.92%
**0.2**	10	80	0.004868	2.43%	5.09%
		100	0.006085	3.04%	5.14%

	50	80	0.004853	2.43%	5.44%
		100	0.006066	3.03%	5.69%
	100	80	0.004834	2.42%	5.88%
		100	0.006042	3.02%	6.38%
	200	80	0.004796	2.40%	6.75%
		100	0.005995	3.00%	7.74%
	300	80	0.004758	2.38%	7.59%
		100	0.005947	2.97%	9.08%
	400	80	0.00472	2.36%	8.41%
		100	0.0059	2.95%	10.38%
	500	80	0.004682	2.34%	9.21%
		100	0.005852	2.93%	11.65%
	600	80	0.004643	2.32%	9.99%
		100	0.005804	2.90%	12.88%
**0.4**	10	80	0.009736	2.43%	5.36%
		100	0.01217	3.04%	5.56%
	50	80	0.009706	2.43%	6.79%
		100	0.01213	3.03%	7.81%
	100	80	0.009668	2.42%	8.58%
		100	0.01208	3.02%	10.65%
	200	80	0.009592	2.40%	12.16%
		100	0.01199	3.00%	16.32%
	300	80	0.009516	2.38%	15.68%
		100	0.01189	2.97%	21.87%
	400	80	0.009439	2.36%	19.11%
		100	0.0118	2.95%	27.22%
	500	80	0.009363	2.34%	22.44%
		100	0.0117	2.93%	32.30%
	600	80	0.009287	2.32%	25.63%
		100	0.01161	2.90%	37.07%

*ordinary least squares estimate

For 23 UK Biobank traits that passed all thresholds in 2SMR analysis:

- sample overlap was 80% (traits derived from the Neale Lab dataset),
- number of instruments ranged from 8 to 633 (nearly 300 for the majority of traits),
- OLS were considered 0.1 (SMR beta coefficients were approximately 0.05 or less, and we assumed that potential confound could inflate the OLS value).

Thus, the observed relative bias was 2.3-2.4%, and type 1 error rate was 5-6%.

REFERENCES:

1. Stock, J. & Yogo, M. Testing for weak instruments in linear IV regression. *SSRN eLibrary* **11,** T0284 (2002).
2. Nelson, C. & Startz, R. The distribution of the instrumental variables estimator and its t-ratio when the instrument is a poor one. *J. Bus.* **63,** 125–140 (1990).
3. Bound, J., Jaeger, D. A. & Baker, R. M. Problems with instrumental variables estimation when the correlation between the instruments and the endogeneous explanatory variable is weak. *J. Am. Stat. Assoc.* **90,** 443-450 (1995).
4. Staiger, D. & Stock, J. H. Instrumental variables regression with weak instruments. *Econometrica* **65,** 557–586 (1997).
5. Pierce, B. L. & Burgess, S. Efficient design for Mendelian randomization studies: Subsample and 2-sample instrumental variable estimators. *Am. J. Epidemiol.* **178,** 1177–1184 (2013). doi: 10.1093/aje/kwt084
6. Burgess, S., Dudbridge, F. & Thompson, S. G. Combining information on multiple instrumental variables in Mendelian randomization: Comparison of allele score and summarized data methods. *Stat. Med.* **35,** 1880–1906 (2016). doi: 10.1002/sim.6835
7. Burgess, S. & Thompson, S. G. Bias in causal estimates from Mendelian randomization studies with weak instruments. *Stat. Med.* **30,** 1312–1323 (2011). doi: 10.1002/sim.4197
8. Burgess, S., Davies, N. M. & Thompson, S. G. Bias due to participant overlap in two-sample Mendelian randomization. *Genet. Epidemiol.* **40,** 597–608 (2016). doi: 10.1002/gepi.21998
