# Supplementary material for: Varicose veins of lower extremities: Insights from the first large-scale genetic study
Source: PLoS Genet. 2019 Apr 18;15(4):e1008110. doi: 10.1371/journal.pgen.1008110 (PMC6490943; doi:10.1371/journal.pgen.1008110)

Figure S1. A quantile-quantile plot for observed vs. expected distribution of P-values for χ2 statistics.

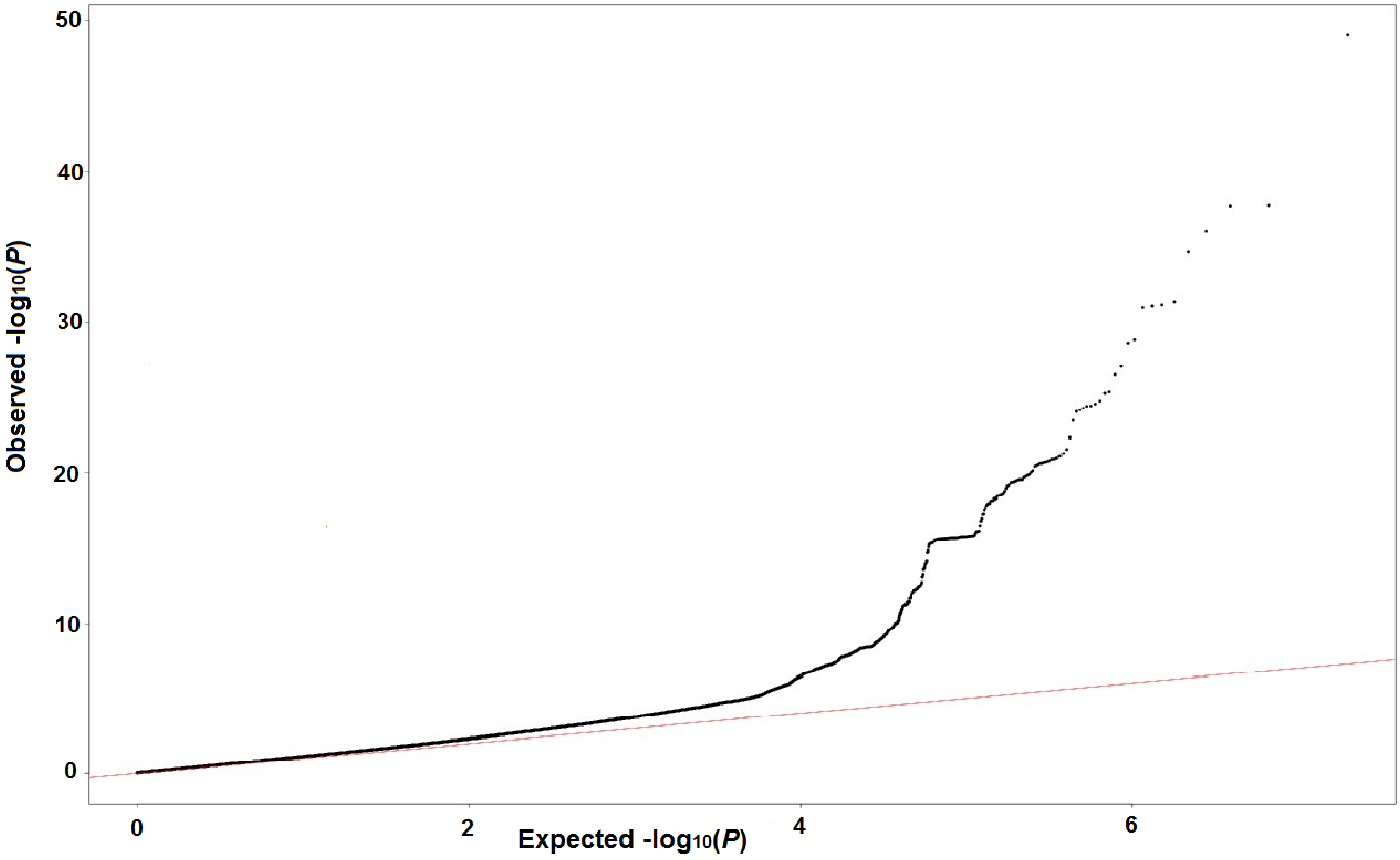

Supplement: S1 Fig — (PDF) [file pgen.1008110.s002.pdf]
