# Supplementary material for: Varicose veins of lower extremities: Insights from the first large-scale genetic study
Source: PLoS Genet. 2019 Apr 18;15(4):e1008110. doi: 10.1371/journal.pgen.1008110 (PMC6490943; doi:10.1371/journal.pgen.1008110)
Supplement: S2 Fig — Color of circles indicates the strength of linkage disequilibrium with the lead SNP based on the squared correlation coefficient (r2). Blue line indicates recombination rate (cM/Mb). Genes are indicated as blue bars under the plot. (PDF) [file pgen.1008110.s003.pdf]

Figure S2. Regional association plots of –log10 (P) for SNPs located at the distance of ≤ 250 kb from the index SNPs. Color of circles indicates the strength of linkage disequilibrium with the lead SNP based on the squared correlation coefficient (r2). Blue line indicates recombination rate (cM/Mb). Genes are indicated as blue bars under the plot.

rs11121615 2

rs2911463 3

rs2861819 4

rs3101725 5

rs11135046 6

rs28558138 7

rs7773004 8

rs9880192 9

rs12625547 10

rs236530 11

rs2241173 12

rs73107980 13

rs11121615

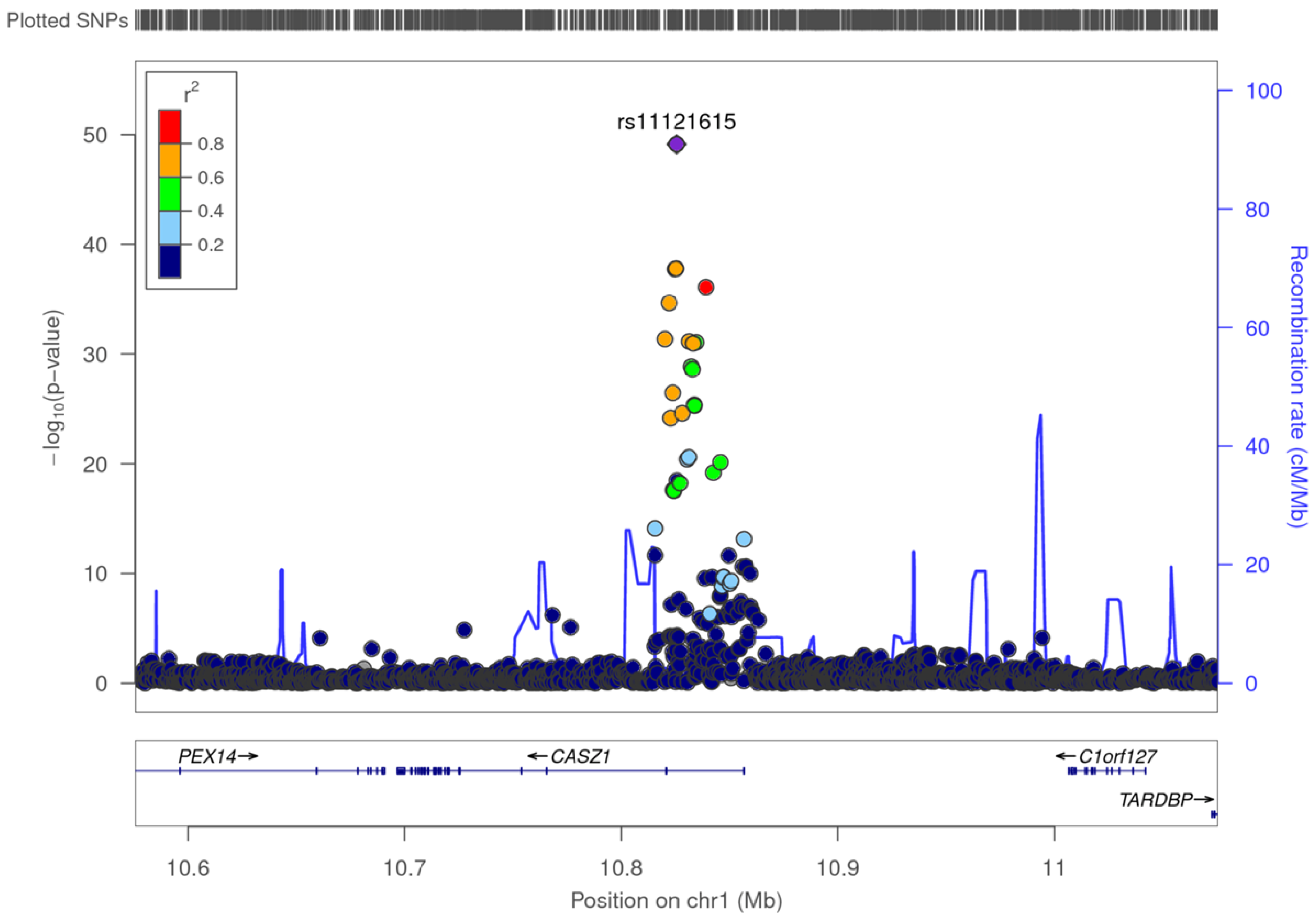
Plotted SNPs
r2
0.8
0.6
0.4
0.2
rs11121615
50
40
30
20
10
0
−log10(p−value)
100
80
60
40
20
0
Recombination rate (cM/Mb)
PEX14
CASZ1
C1orf127
TARDBP
10.6
10.7
10.8
10.9
11
Position on chr1 (Mb)

rs2911463

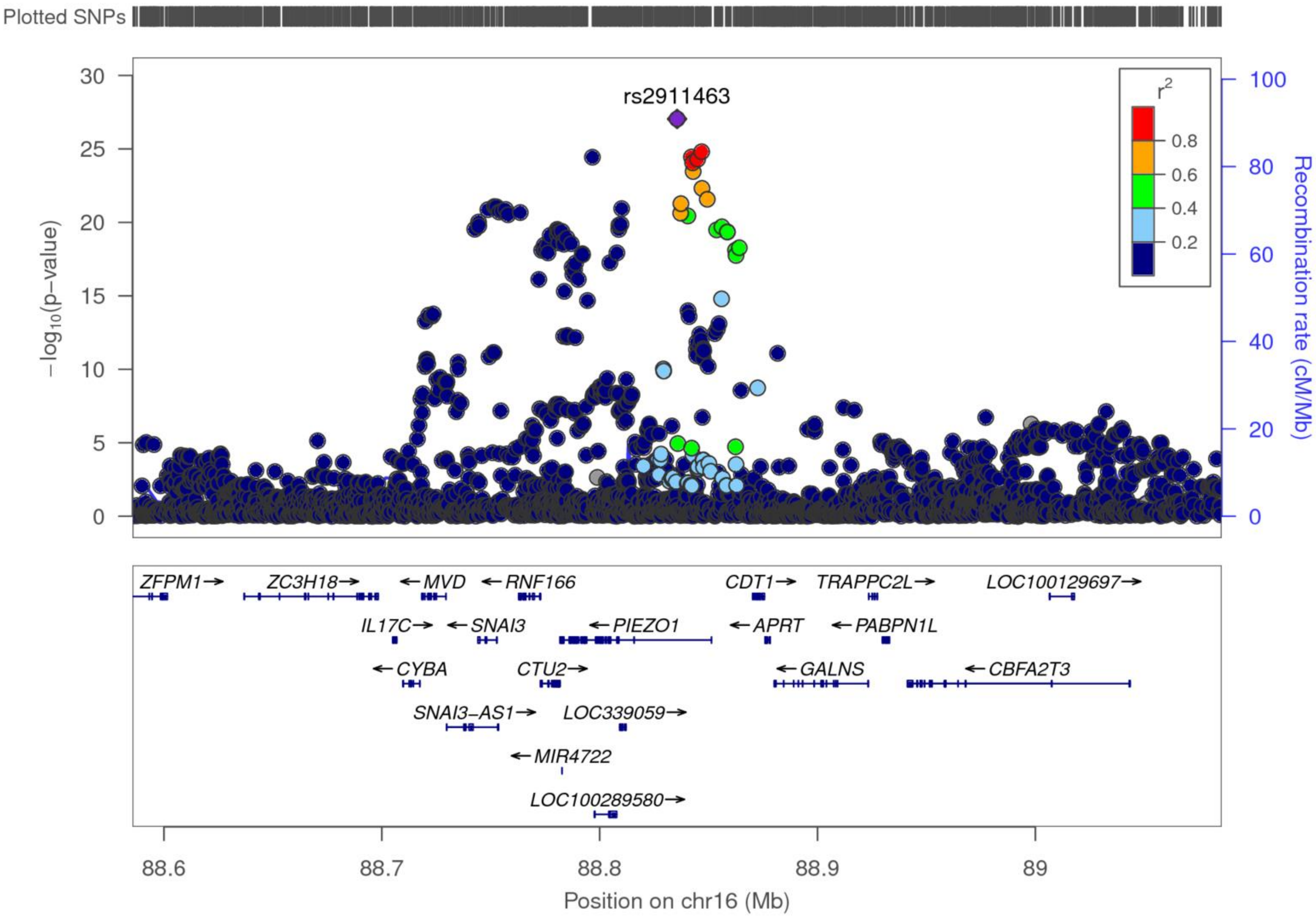

Plotted SNPs
rs2911463
−log10(p−value)
Recombination rate (cM/Mb)
r2
0.8
0.6
0.4
0.2
ZFPM1→
ZC3H18→
←MVD
←RNF166
CDT1→
TRAPPC2L→
LOC100129697→
IL17C→
←SNAI3
←PIEZO1
←APRT
←PABPN1L
←CYBA
CTU2→
←GALNS
←CBFA2T3
SNAI3−AS1→
LOC339059→
←MIR4722
LOC100289580→
88.6
88.7
88.8
88.9
89
Position on chr16 (Mb)

rs2861819

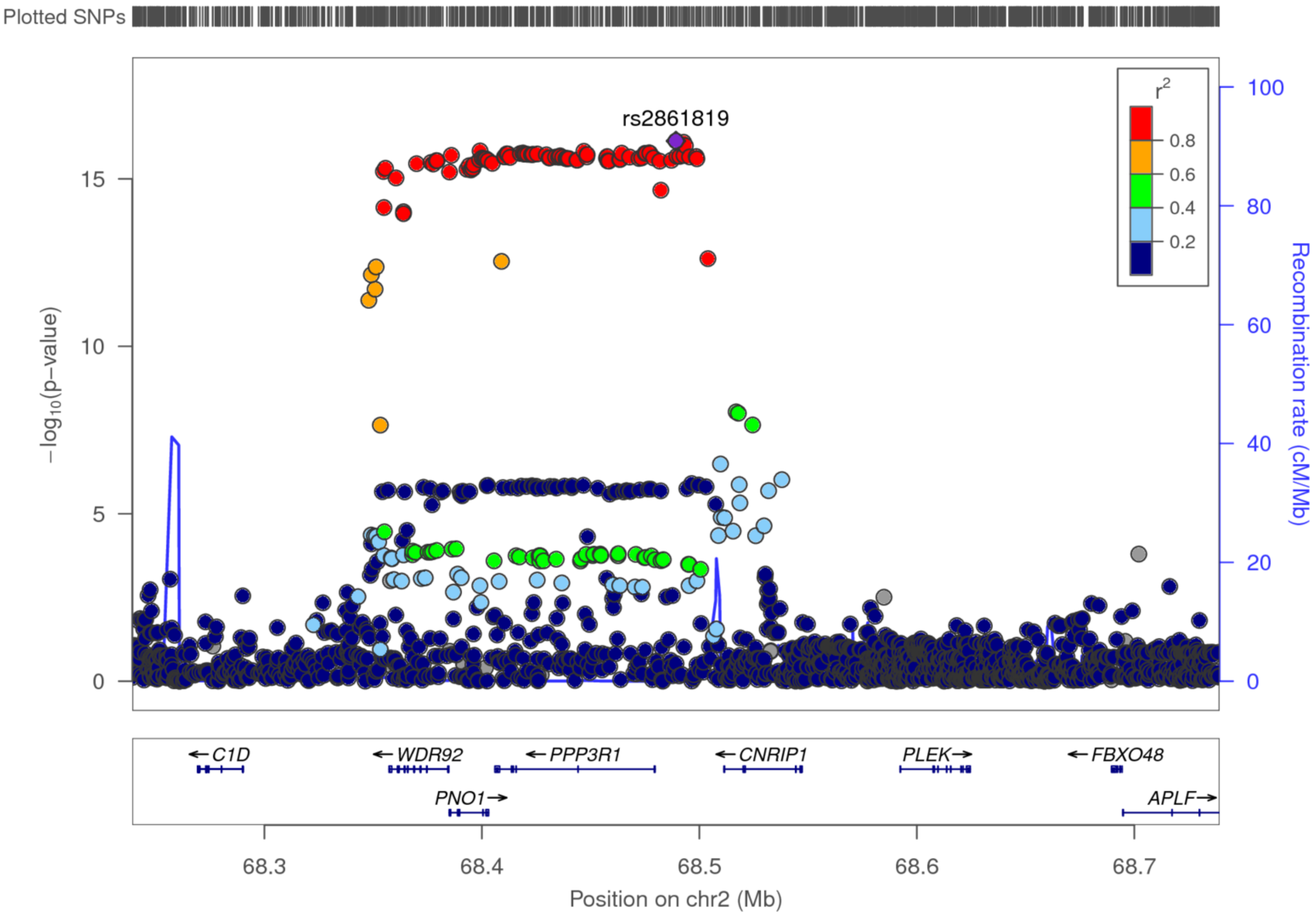
Plotted SNPs
rs2861819
r2
0.8
0.6
0.4
0.2
100
80
60
40
20
0
Recombination rate (cM/Mb)
15
10
5
0
−log10(p−value)
←C1D
←WDR92
←PPP3R1
←CNRIP1
PLEK→
←FBXO48
PNO1→
APLF→
68.3
68.4
68.5
68.6
68.7
Position on chr2 (Mb)

rs3101725

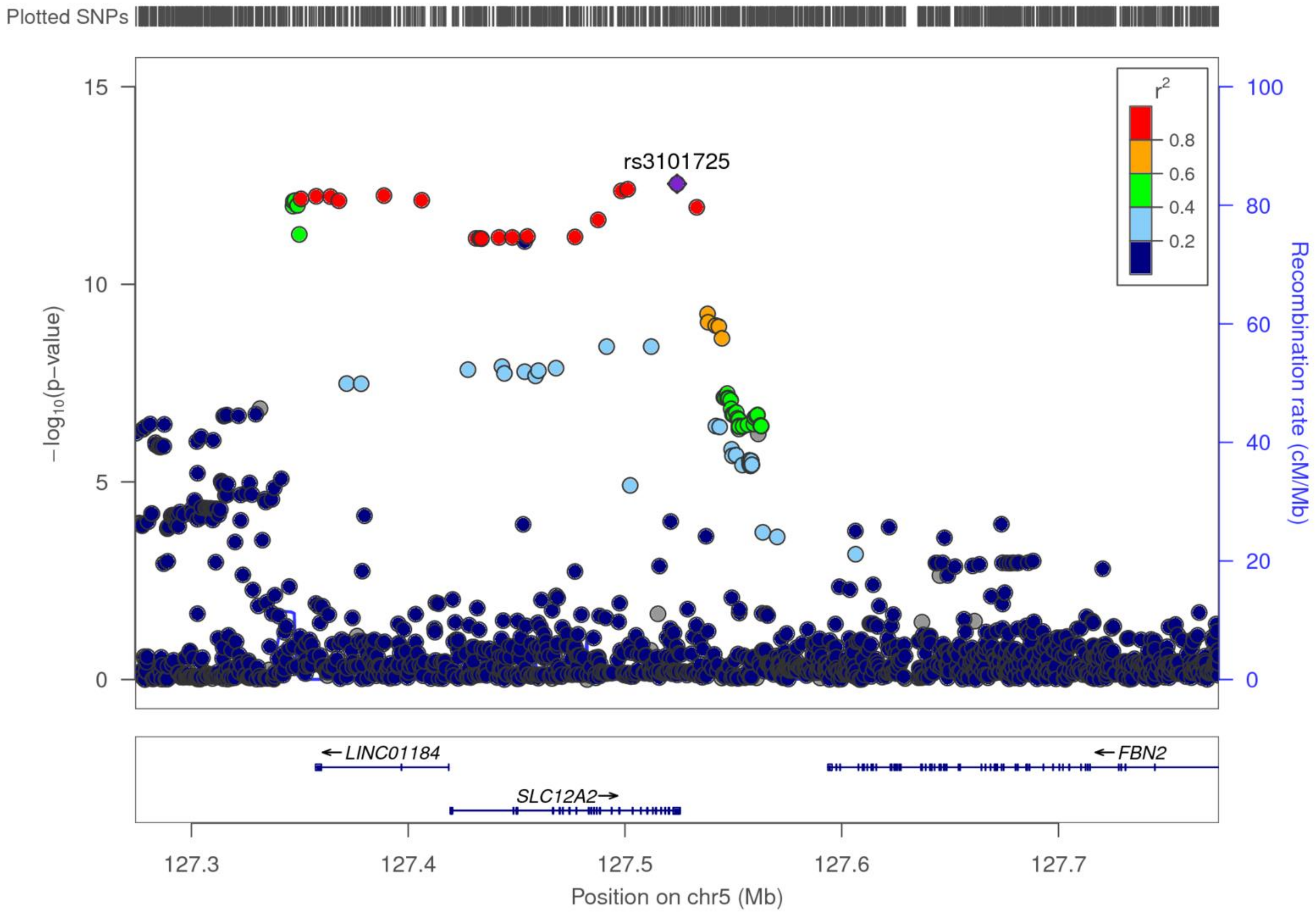

Plotted SNPs
rs3101725
r²
0.8
0.6
0.4
0.2
15
10
5
0
−log10(p−value)
100
80
60
40
20
0
Recombination rate (cM/Mb)
← LINC01184
SLC12A2 →
← FBN2
127.3
127.4
127.5
127.6
127.7
Position on chr5 (Mb)

rs11135046

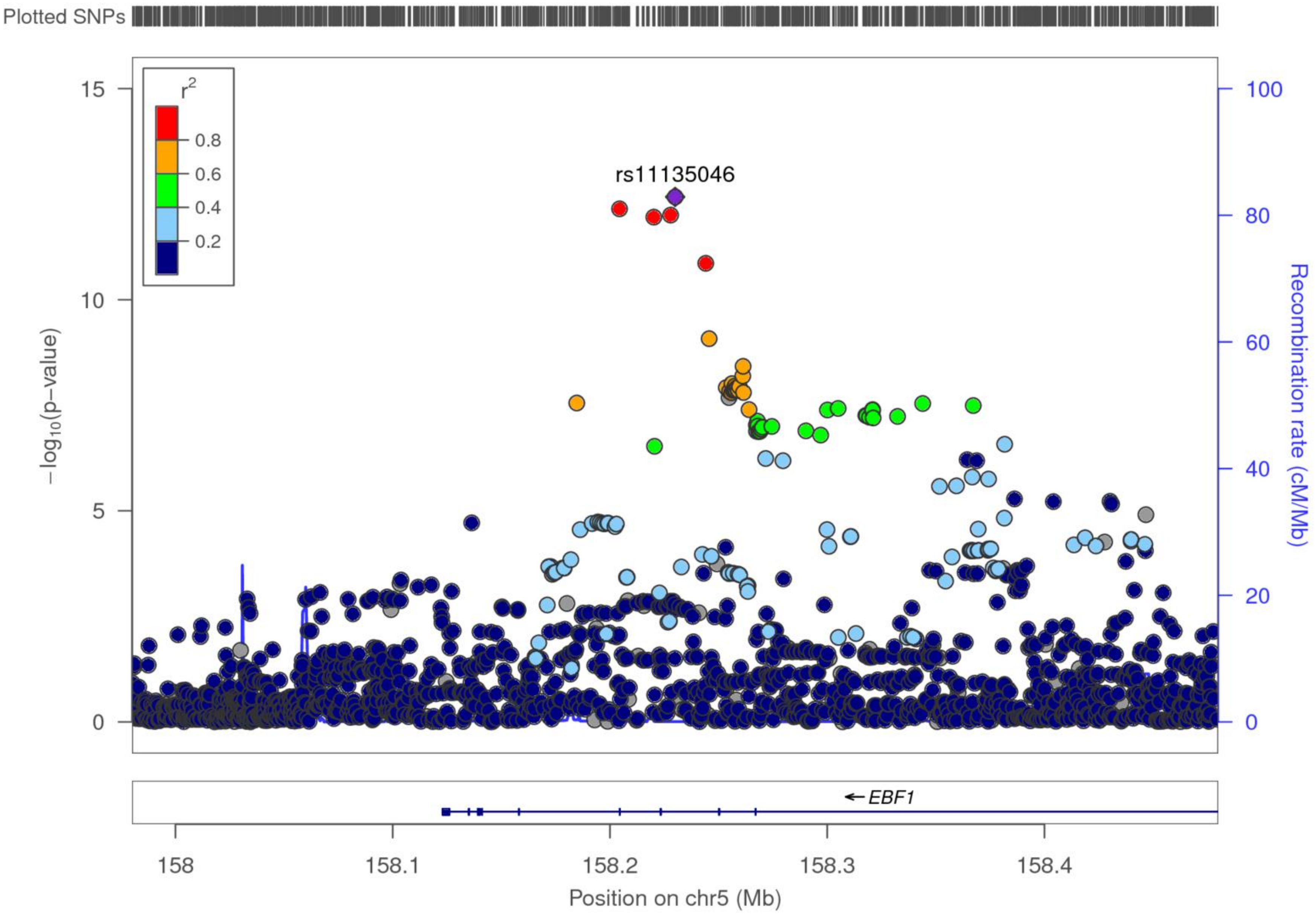
Plotted SNPs
r2
0.8
0.6
0.4
0.2
rs11135046
15
10
5
0
−log10(p−value)
100
80
60
40
20
0
Recombination rate (cM/Mb)
← EBF1
158
158.1
158.2
158.3
158.4
Position on chr5 (Mb)

rs28558138

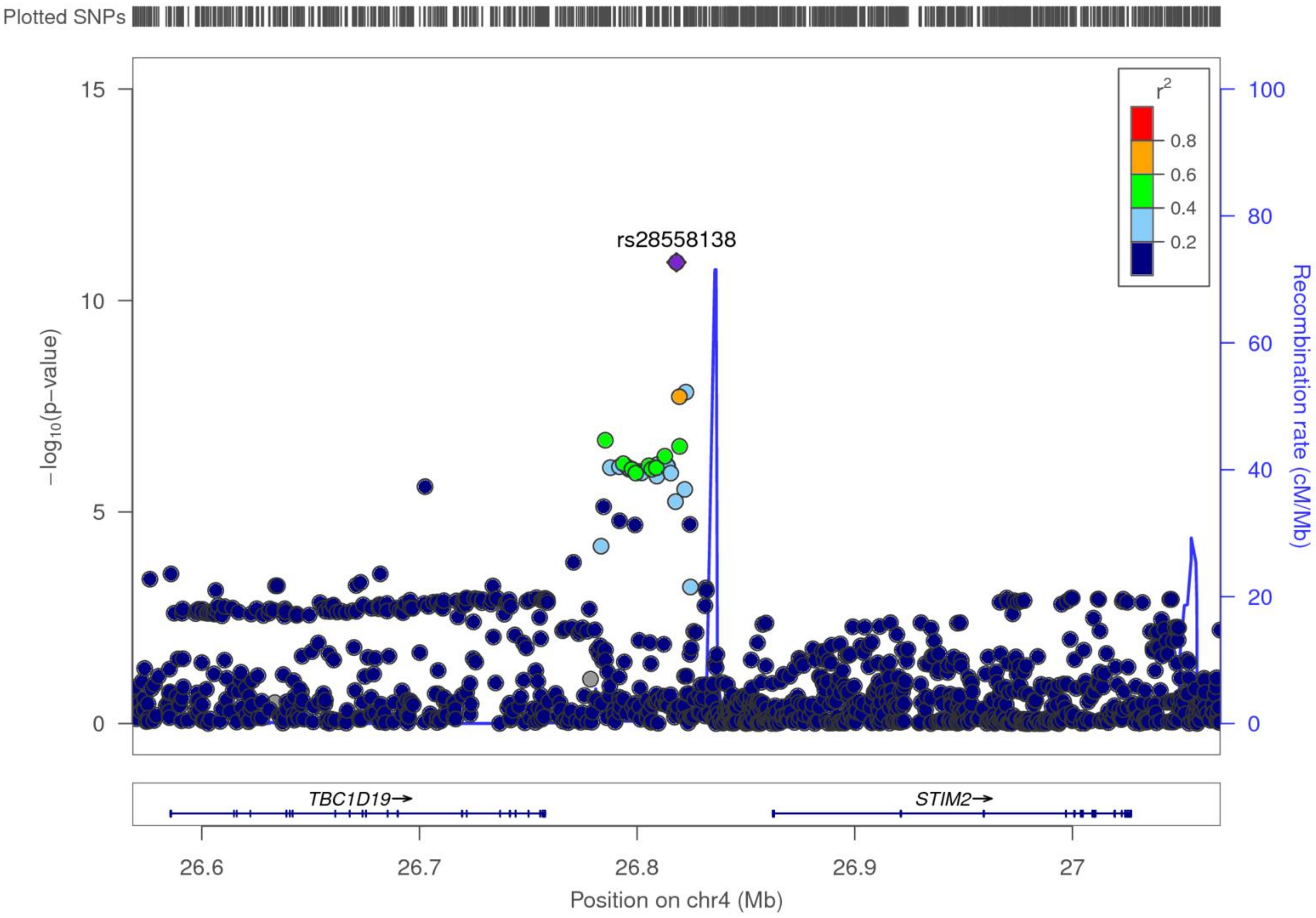
Plotted SNPs
15
10
5
0
−log10(p−value)
rs28558138
r2
0.8
0.6
0.4
0.2
100
80
60
40
20
0
Recombination rate (cM/Mb)
TBC1D19→
STIM2→
26.6
26.7
26.8
26.9
27
Position on chr4 (Mb)

rs7773004

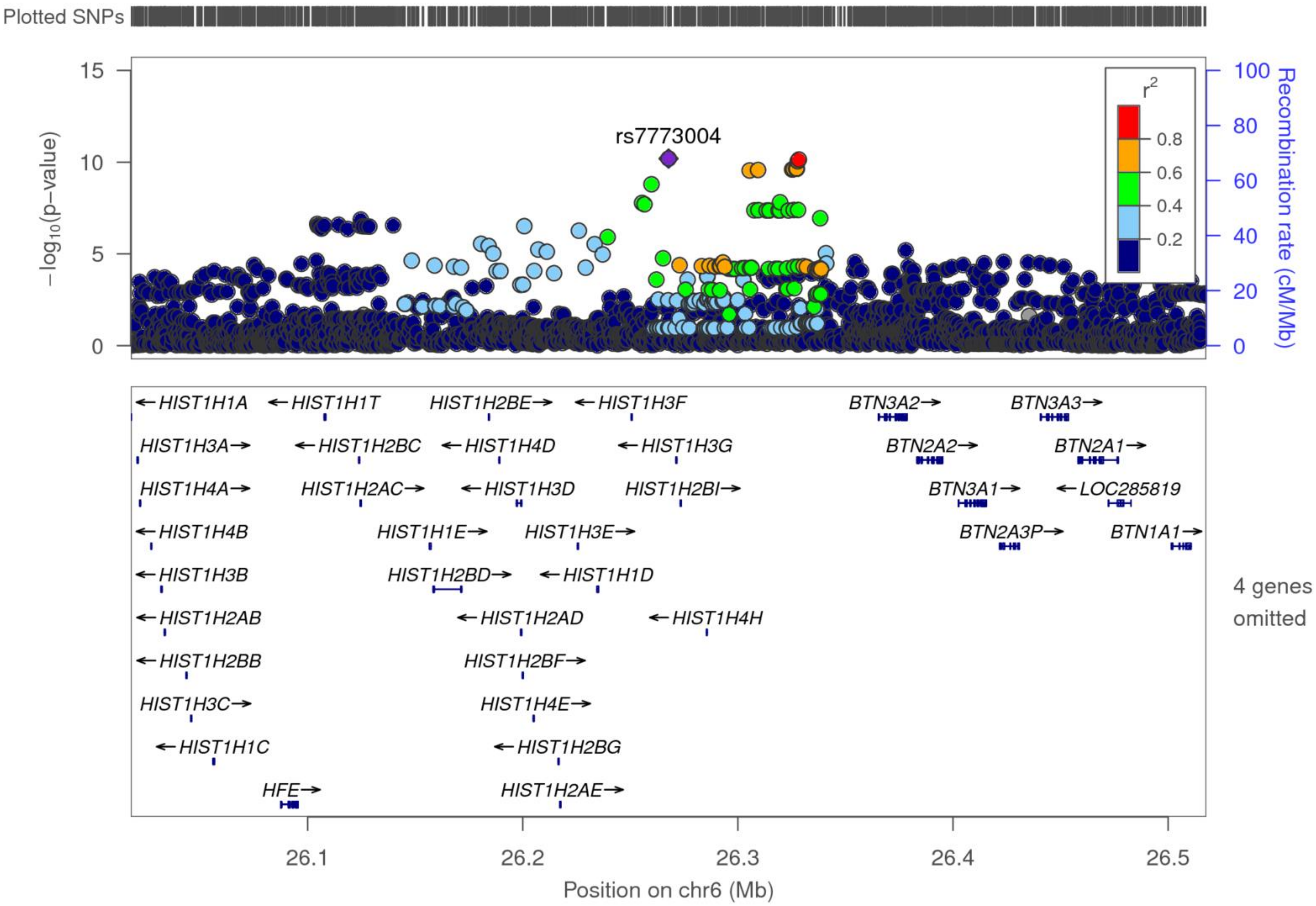

Plotted SNPs
rs7773004
−log10(p−value)
15
10
5
0
100
80
60
40
20
0
Recombination rate (cM/Mb)
r2
0.8
0.6
0.4
0.2
HIST1H1A
HIST1H1T
HIST1H2BE
HIST1H3F
BTN3A2
BTN3A3
HIST1H3A
HIST1H2BC
HIST1H4D
HIST1H3G
BTN2A2
BTN2A1
HIST1H4A
HIST1H2AC
HIST1H3D
HIST1H2BI
BTN3A1
LOC285819
HIST1H4B
HIST1H1E
HIST1H3E
BTN2A3P
BTN1A1
HIST1H3B
HIST1H2BD
HIST1H1D
HIST1H2AB
HIST1H2AD
HIST1H4H
HIST1H2BB
HIST1H2BF
HIST1H3C
HIST1H4E
HIST1H1C
HIST1H2BG
HFE
HIST1H2AE
4 genes omitted
26.1
26.2
26.3
26.4
26.5
Position on chr6 (Mb)

rs9880192

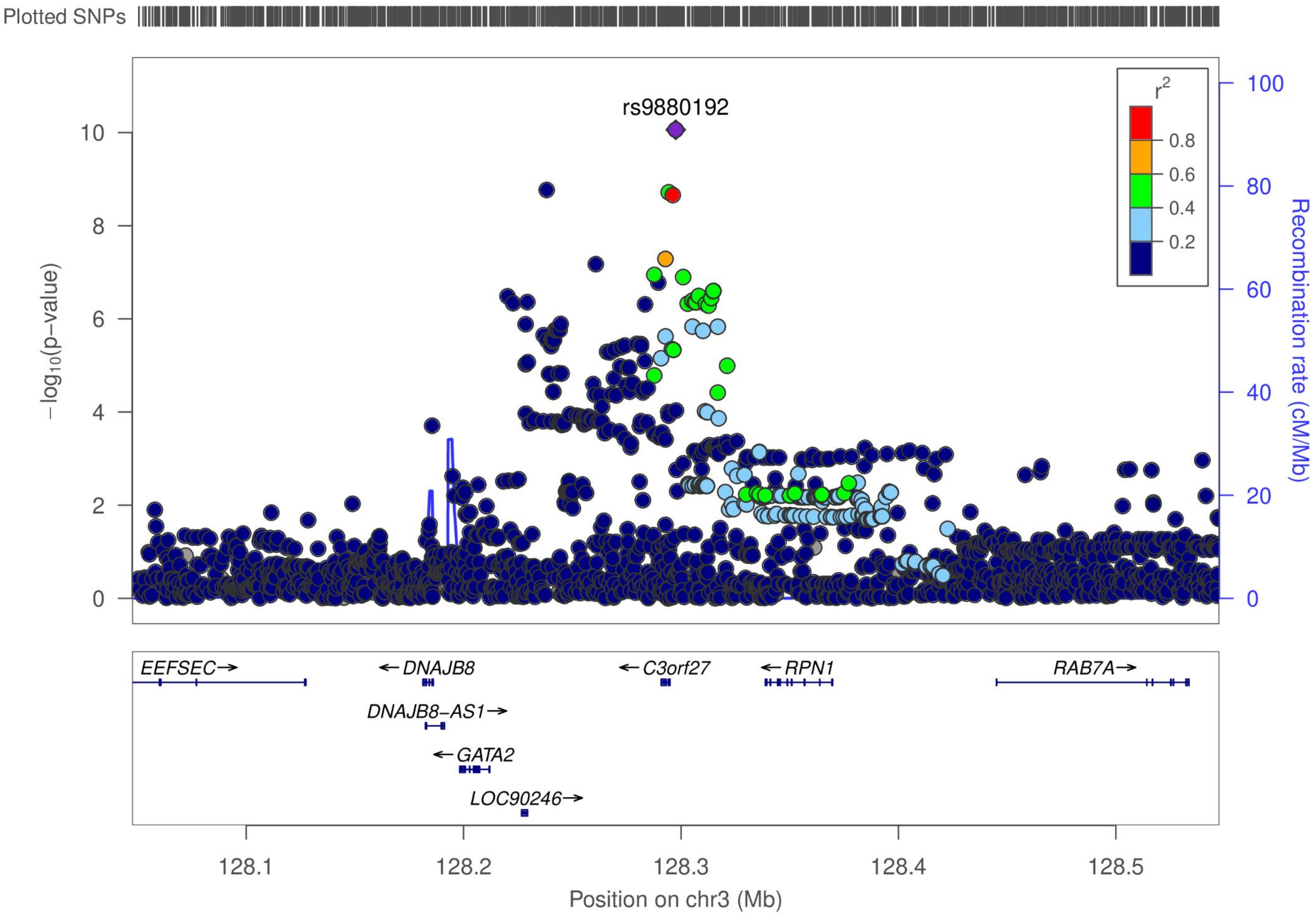

Plotted SNPs
rs9880192
r2
0.8
0.6
0.4
0.2
−log10(p−value)
Recombination rate (cM/Mb)
EEFSEC→
←DNAJB8
←C3orf27
←RPN1
RAB7A→
DNAJB8−AS1→
←GATA2
LOC90246→
128.1
128.2
128.3
128.4
128.5
Position on chr3 (Mb)

rs12625547

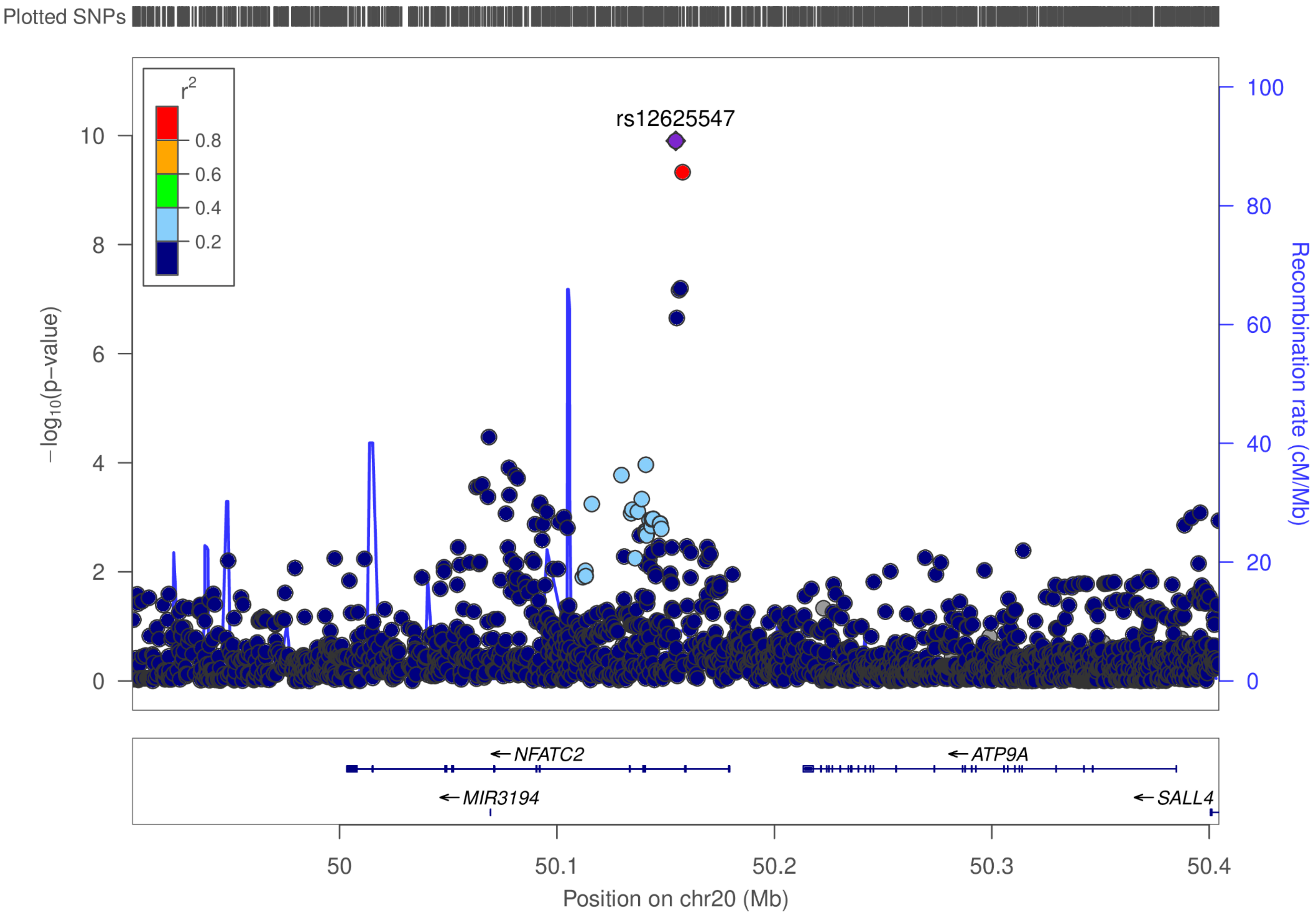

Plotted SNPs
r2
0.8
0.6
0.4
0.2
rs12625547
10
8
6
4
2
0
−log10(p−value)
100
80
60
40
20
0
Recombination rate (cM/Mb)
NFATC2
ATP9A
MIR3194
SALL4
50
50.1
50.2
50.3
50.4
Position on chr20 (Mb)

rs236530

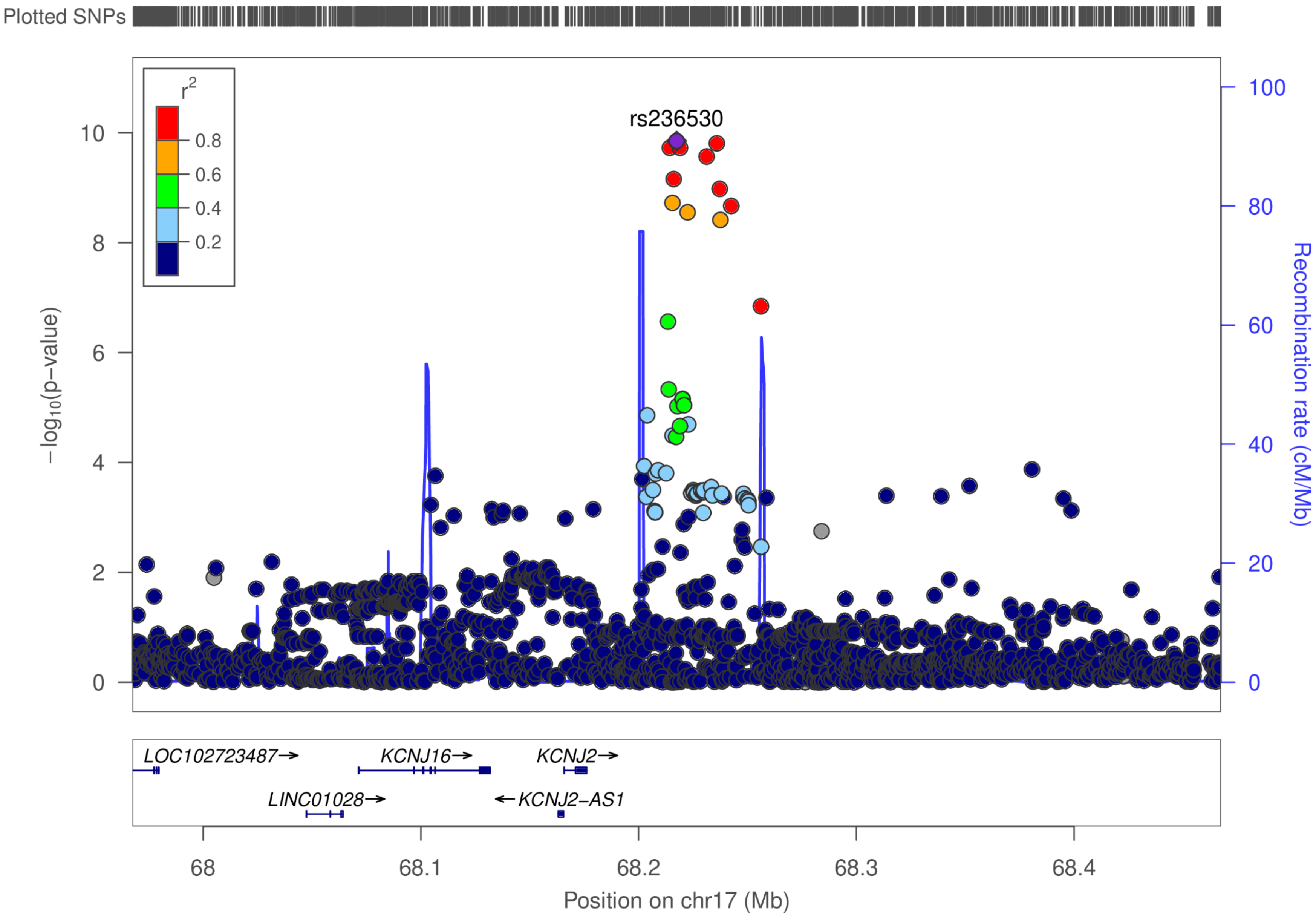

Plotted SNPs
r2
0.8
0.6
0.4
0.2
rs236530
10
8
6
4
2
0
−log10(p−value)
100
80
60
40
20
0
Recombination rate (cM/Mb)
LOC102723487→
KCNJ16→
KCNJ2→
LINC01028→
←KCNJ2−AS1
68
68.1
68.2
68.3
68.4
Position on chr17 (Mb)

rs2241173

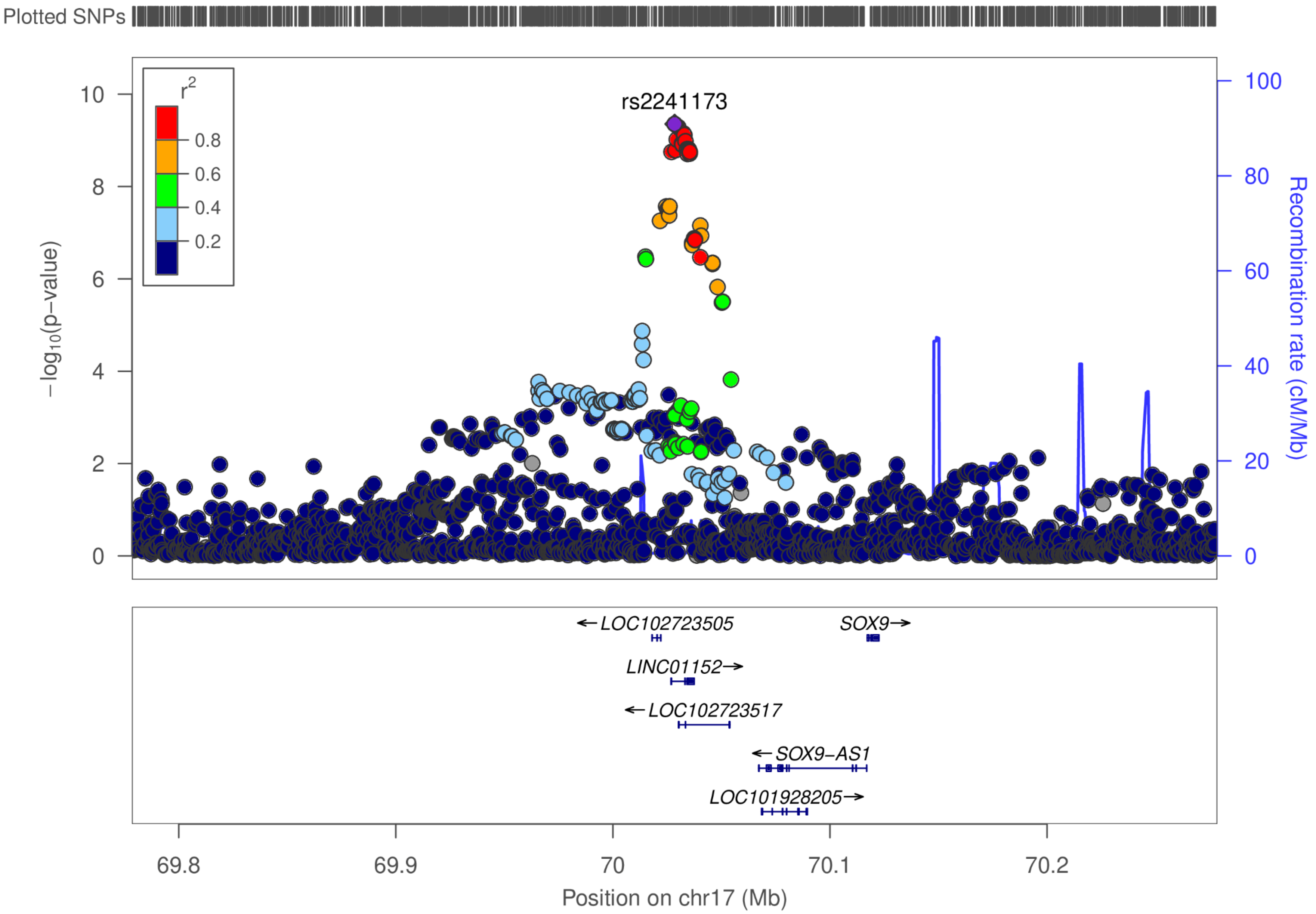

Plotted SNPs
rs2241173
r2
0.8
0.6
0.4
0.2
10
8
6
4
2
0
−log10(p−value)
100
80
60
40
20
0
Recombination rate (cM/Mb)
←LOC102723505
SOX9→
LINC01152→
←LOC102723517
←SOX9−AS1
LOC101928205→
69.8
69.9
70
70.1
70.2
Position on chr17 (Mb)

rs73107980

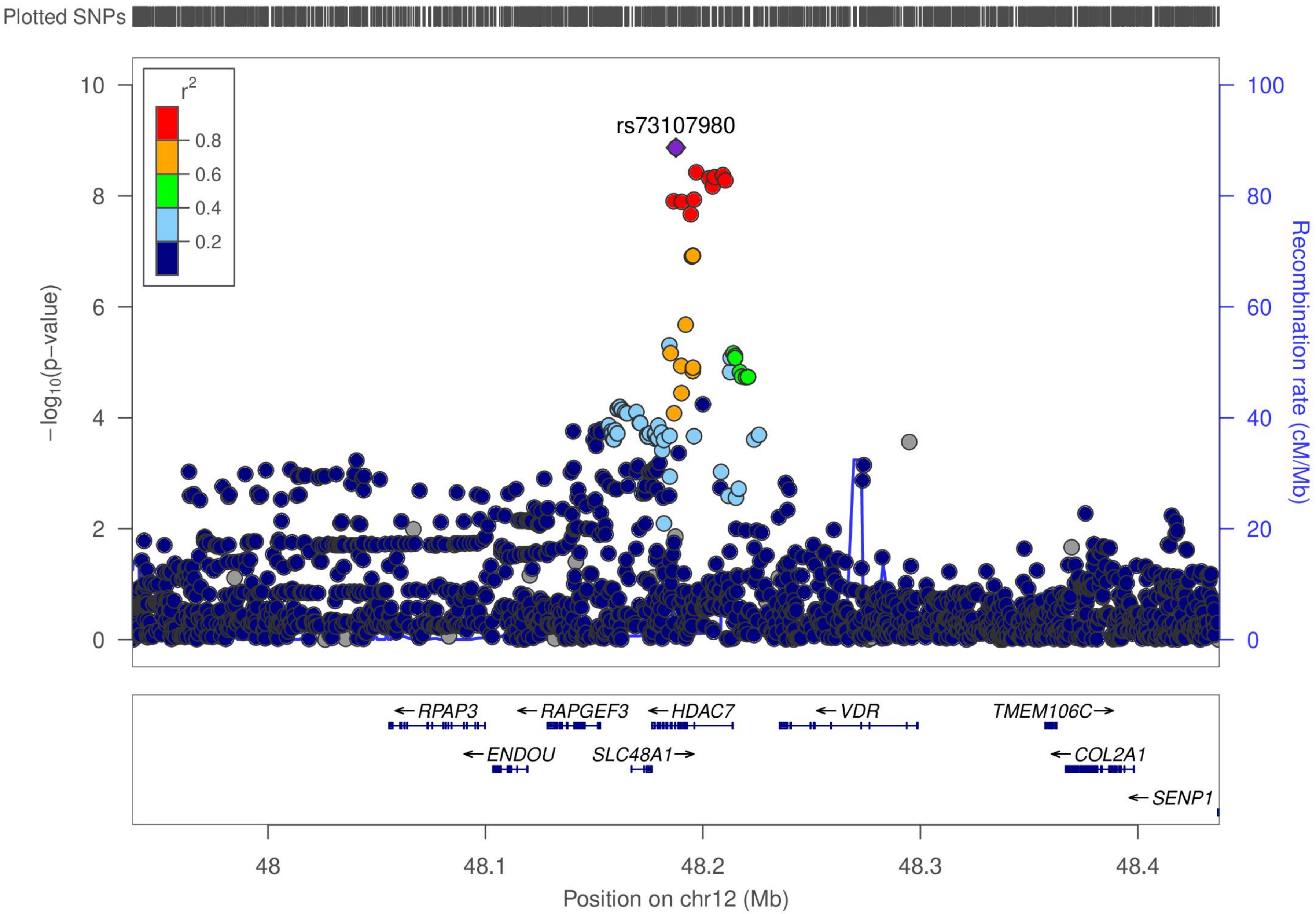

Plotted SNPs
rs73107980
r²
0.8
0.6
0.4
0.2
−log10(p−value)
Recombination rate (cM/Mb)
RPAP3
RAPGEF3
HDAC7
VDR
TMEM106C
ENDOU
SLC48A1
COL2A1
SENP1
48
48.1
48.2
48.3
48.4
Position on chr12 (Mb)
