# Supplementary material for: Varicose veins of lower extremities: Insights from the first large-scale genetic study
Source: PLoS Genet. 2019 Apr 18;15(4):e1008110. doi: 10.1371/journal.pgen.1008110 (PMC6490943; doi:10.1371/journal.pgen.1008110)

Figure S3. Matrix of partial genetic correlations between VVs and a subset of non-collinear complex traits. Color depicts the sign and the absolute value of genetic correlation coefficient (rg). Combinations with statistically significant correlations are marked with points. Full names of traits as indicated in the Neale lab and the Gene ATLAS databases are given in Table S15.

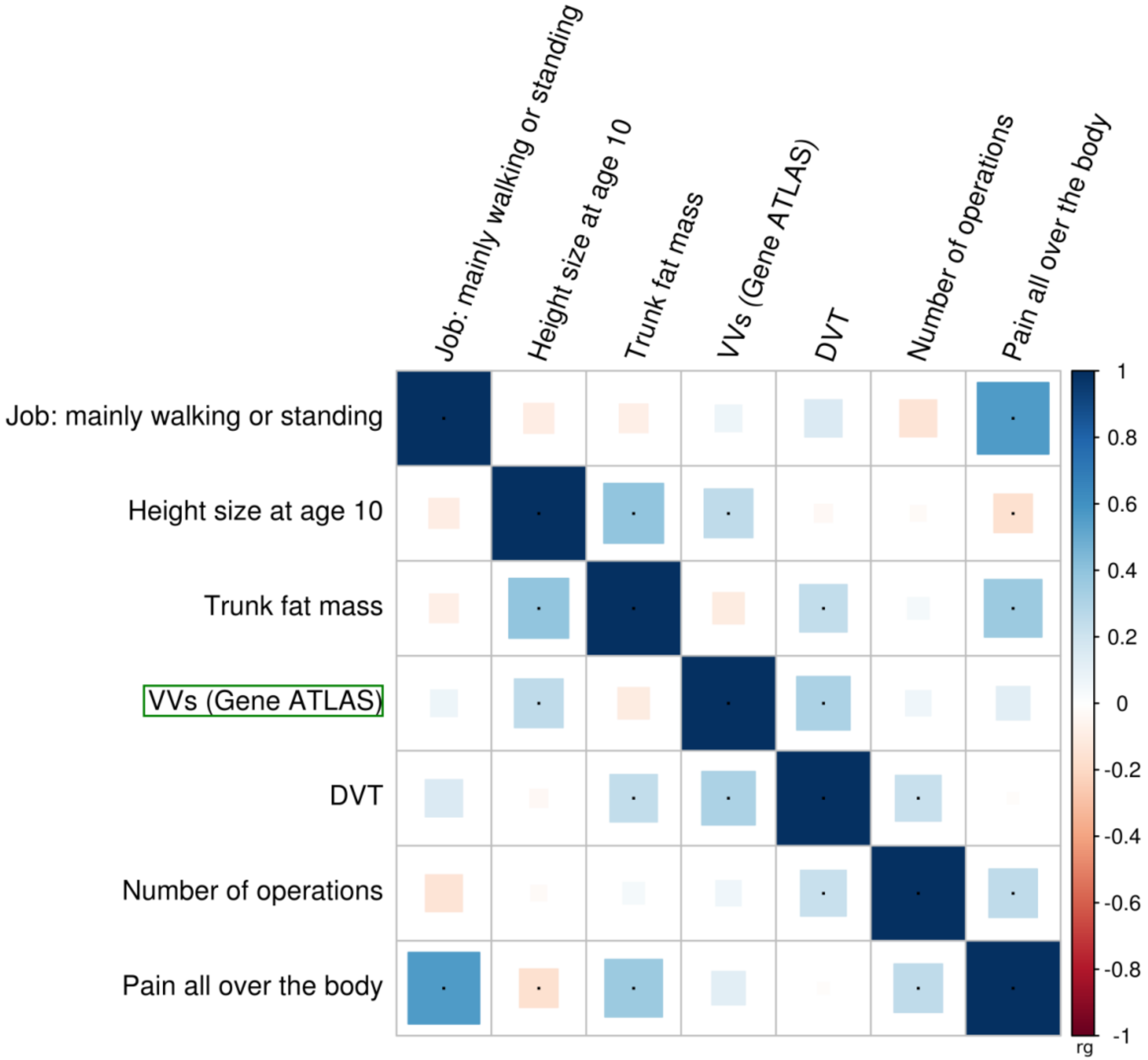

Supplement: S3 Fig — Color depicts the sign and the absolute value of genetic correlation coefficient (rg). Combinations with statistically significant correlations are marked with points. Full names of traits as indicated in the Neale lab and the Gene ATLAS databases are given in S15 Table. (PDF) [file pgen.1008110.s004.pdf]
