# Supplementary material for: Varicose veins of lower extremities: Insights from the first large-scale genetic study
Source: PLoS Genet. 2019 Apr 18;15(4):e1008110. doi: 10.1371/journal.pgen.1008110 (PMC6490943; doi:10.1371/journal.pgen.1008110)

Figure S4. Matrix of partial genetic correlations between VVs, standing height, and weight. Color depicts the sign and the absolute value of genetic correlation coefficient (rg). Combinations with statistically significant correlations are marked with points.

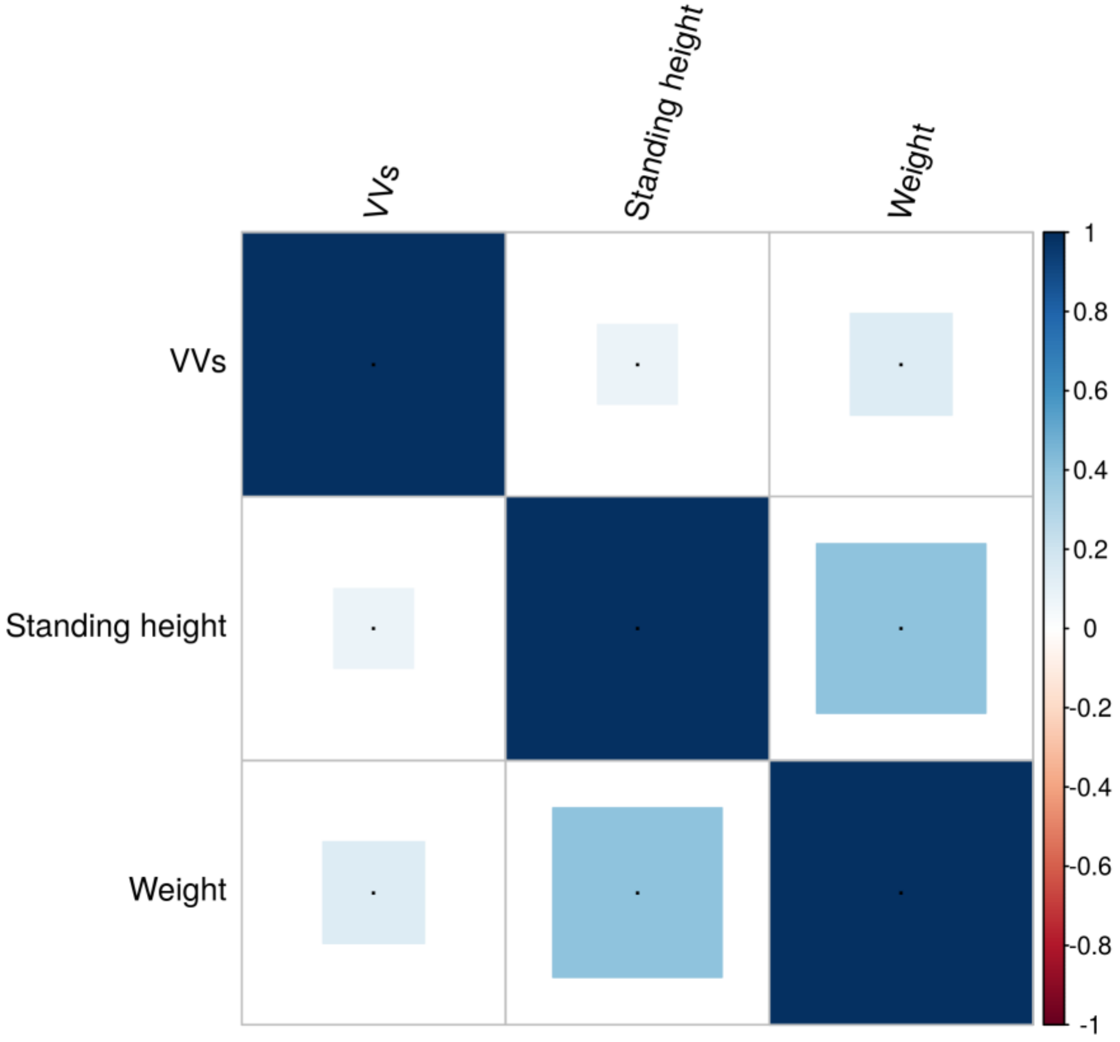

Supplement: S4 Fig — Color depicts the sign and the absolute value of genetic correlation coefficient (rg). Combinations with statistically significant correlations are marked with points. (PDF) [file pgen.1008110.s005.pdf]
