# Supplementary material for: Varicose veins of lower extremities: Insights from the first large-scale genetic study
Source: PLoS Genet. 2019 Apr 18;15(4):e1008110. doi: 10.1371/journal.pgen.1008110 (PMC6490943; doi:10.1371/journal.pgen.1008110)
Supplement: S5 Fig — (PDF) [file pgen.1008110.s006.pdf]

Figure S5. Leave-one-out and Funnel plots before and after removal of instrumental variables associated with VVs with P < 0.01.

Standing height 3

Comparative height size at age 10 5

Leg fat-free mass (left) 7

Whole body fat-free mass 9

Leg predicted mass (left) 11

Basal metabolic rate 13

Trunk fat mass 15

Trunk fat-free mass 17

Weight 19

Whole body water mass 21

Leg predicted mass (right) 23

Leg fat-free mass (right) 25

Sitting height 27

Trunk predicted mass 29

Arm fat-free mass (right) 31

Arm predicted mass (left) 33

Whole body fat mass 35

Arm fat-free mass (left) 37

Hip circumference 39

Arm predicted mass (right) 41
Arm fat mass (left) 43
Arm fat mass (right) 45
Leg fat mass (left) 47
Leg fat mass (right) 49
Forced vital capacity (FVC), Best measure 51
Trunk fat percentage 53
Waist circumference 55
Arm fat percentage (right) 57
MHC class I polypeptide-related sequence B 59
Forced vital capacity (FVC) 61
Arm fat percentage (left) 63
Impedance of leg (left) 65
Impedance of leg (right) 67
Forced expiratory volume in 1-second (FEV1), Best measure 69
Forced expiratory volume in 1-second (FEV1) 71
Non-cancer illness code, self-reported: malabsorption/coeliac disease 73
CD209 antigen 75
Tissue factor 75

Standing height

<table>
<tr><td>Before removing instruments</td><td>After removing instruments</td></tr>
<tr><td>Leave-one-out plot
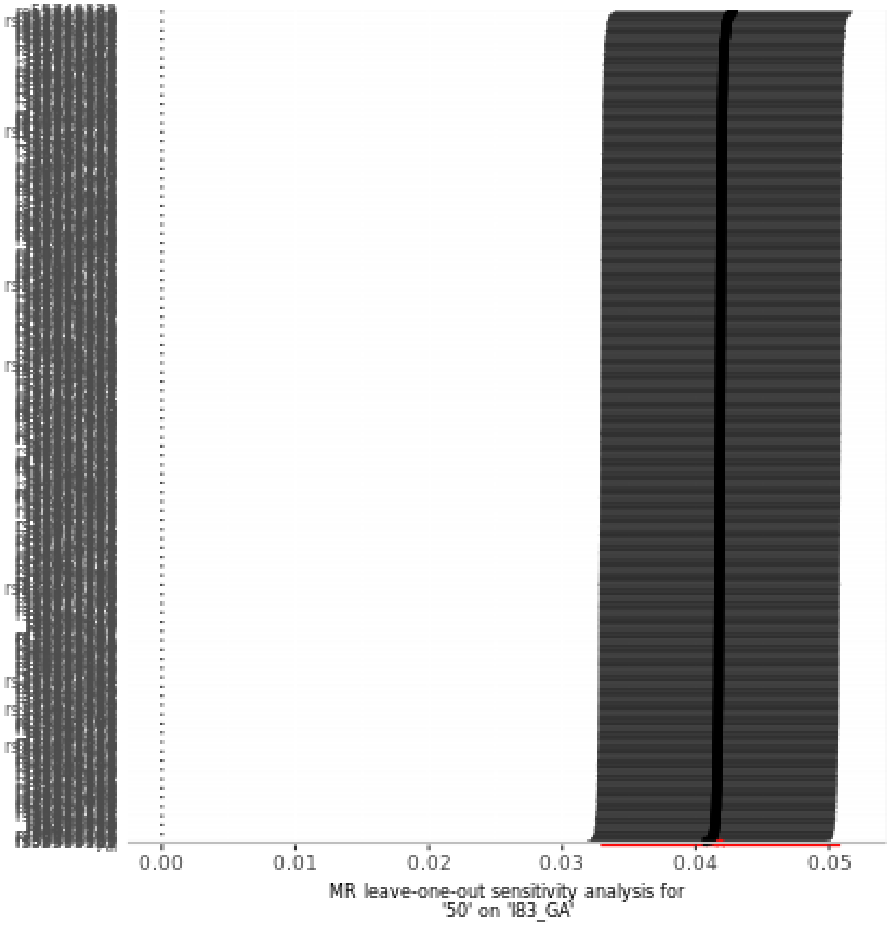
</td><td>Leave-one-out plot
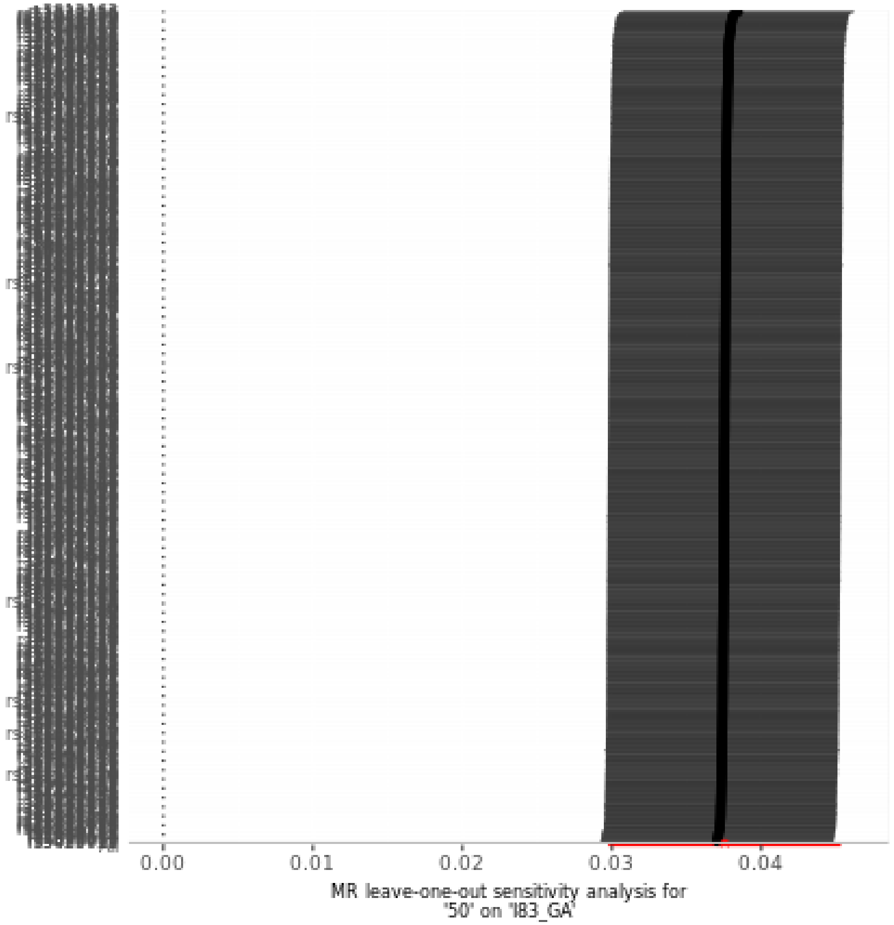
</td></tr>
</table>

Funnel plot

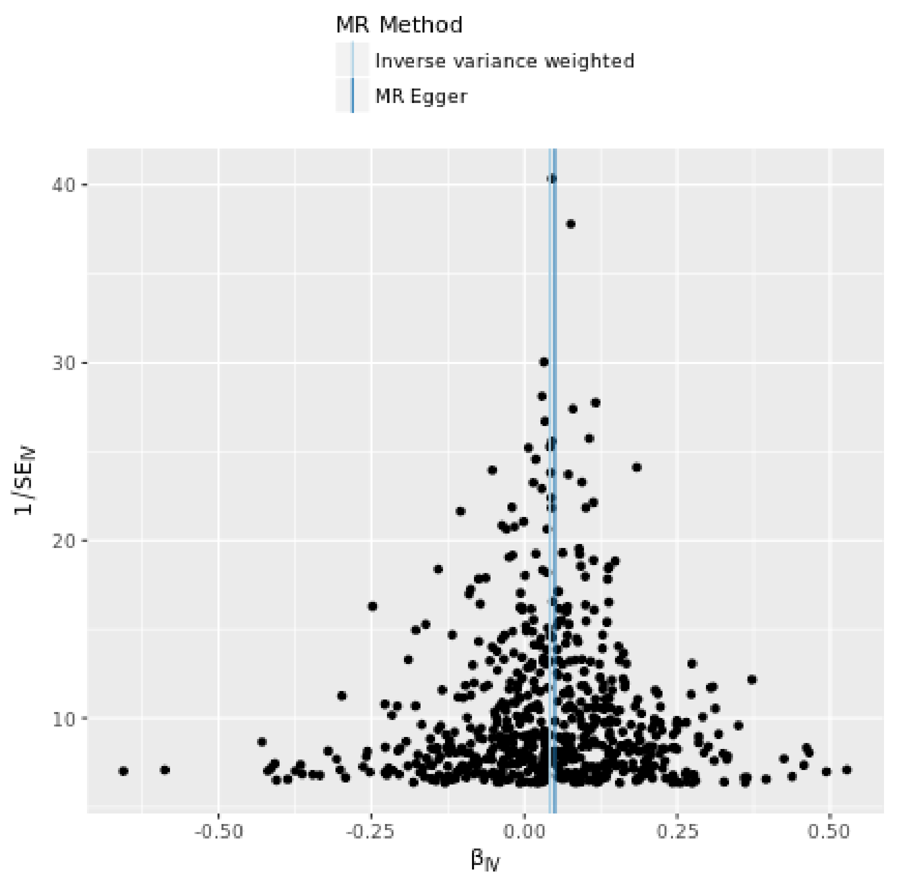

Funnel plot

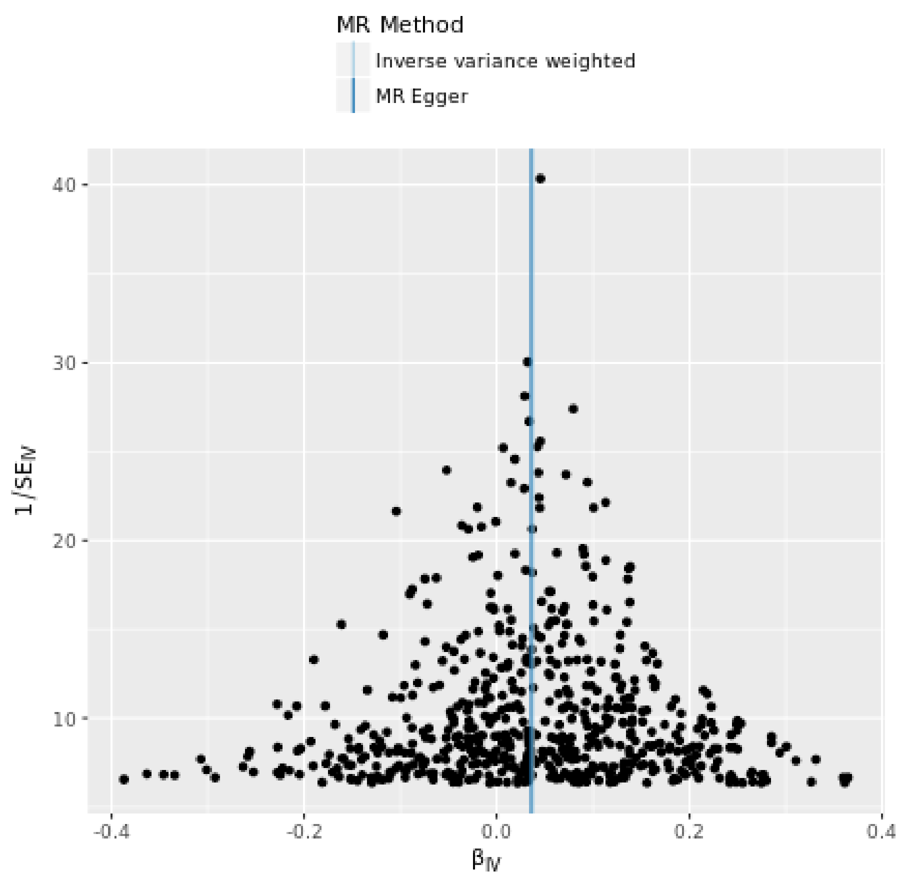

Comparative height size at age 10

Before removing instruments	After removing instruments
Leave-one-out plot<br>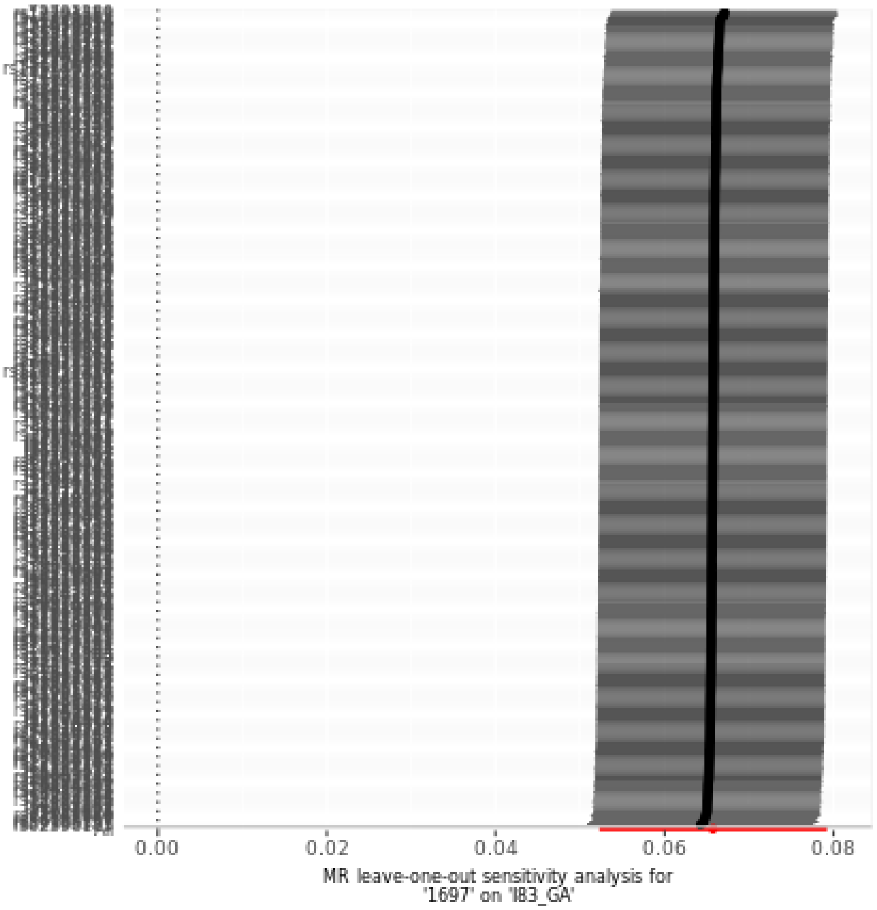 	Leave-one-out plot<br>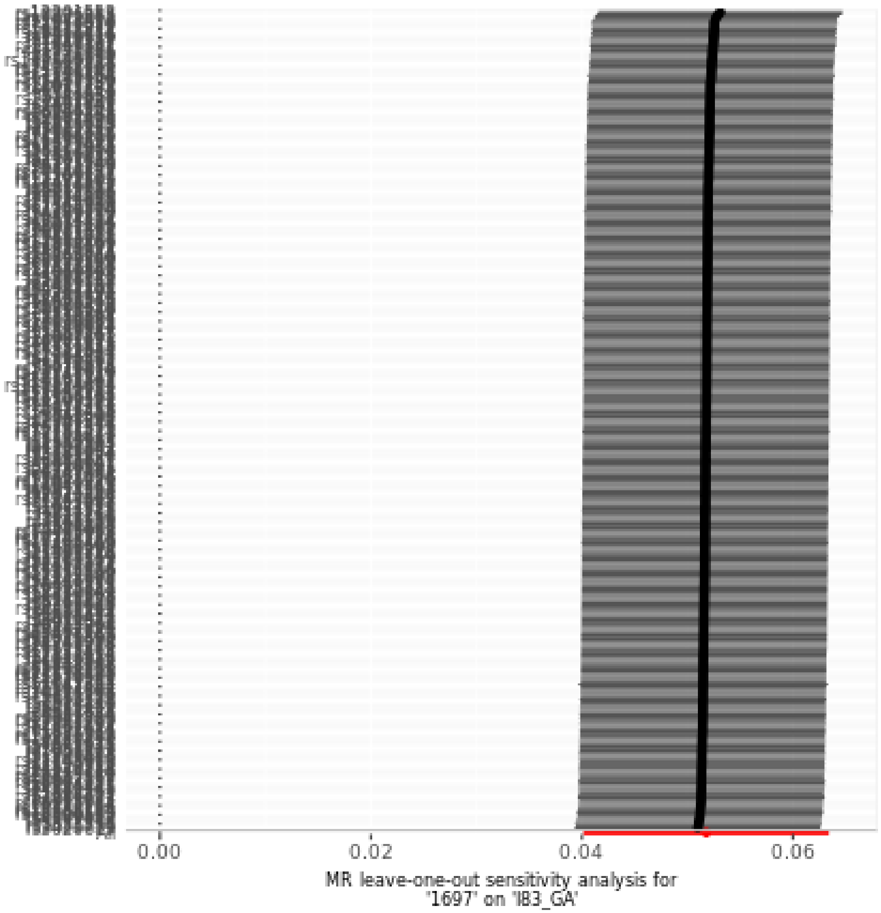

Funnel plot 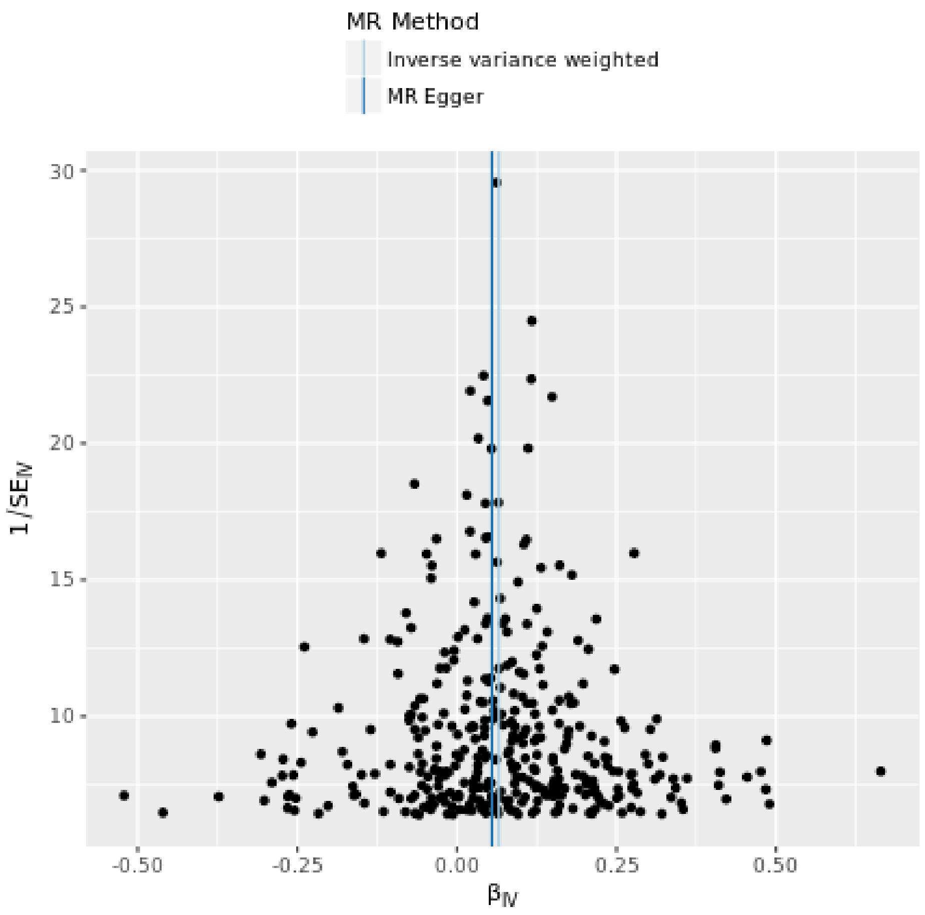	Funnel plot 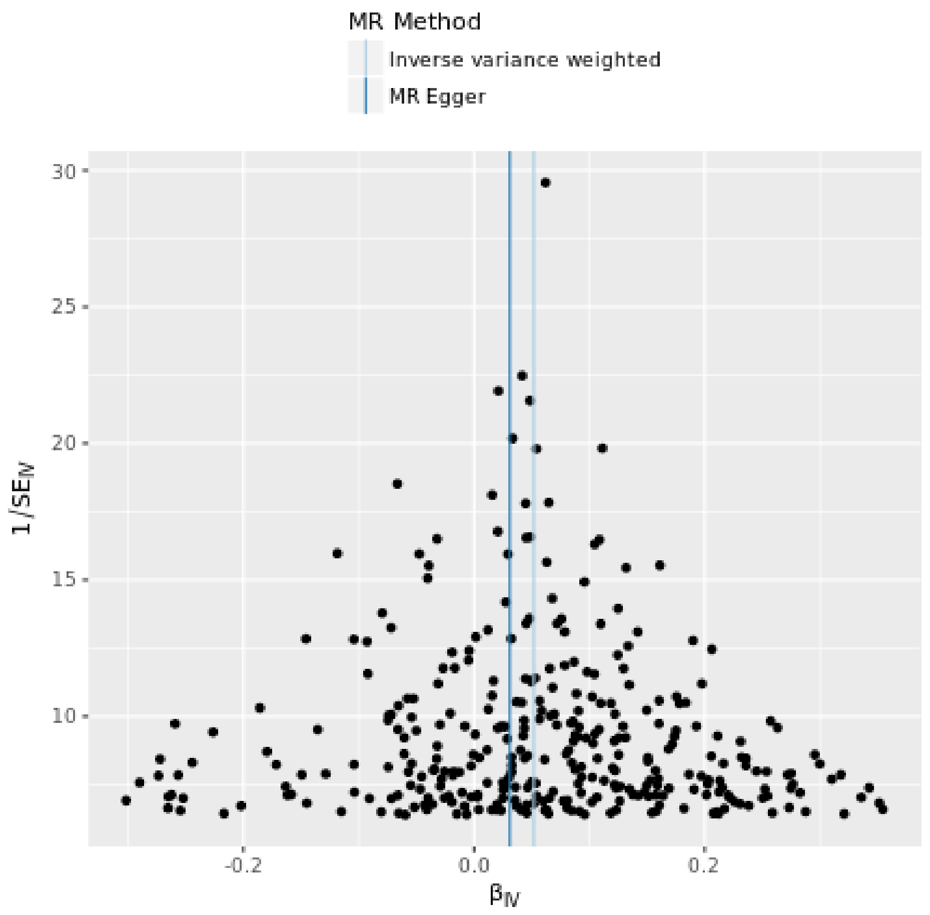

Leg fat-free mass (left)

Before removing instruments	After removing instruments
Leave-one-out plot 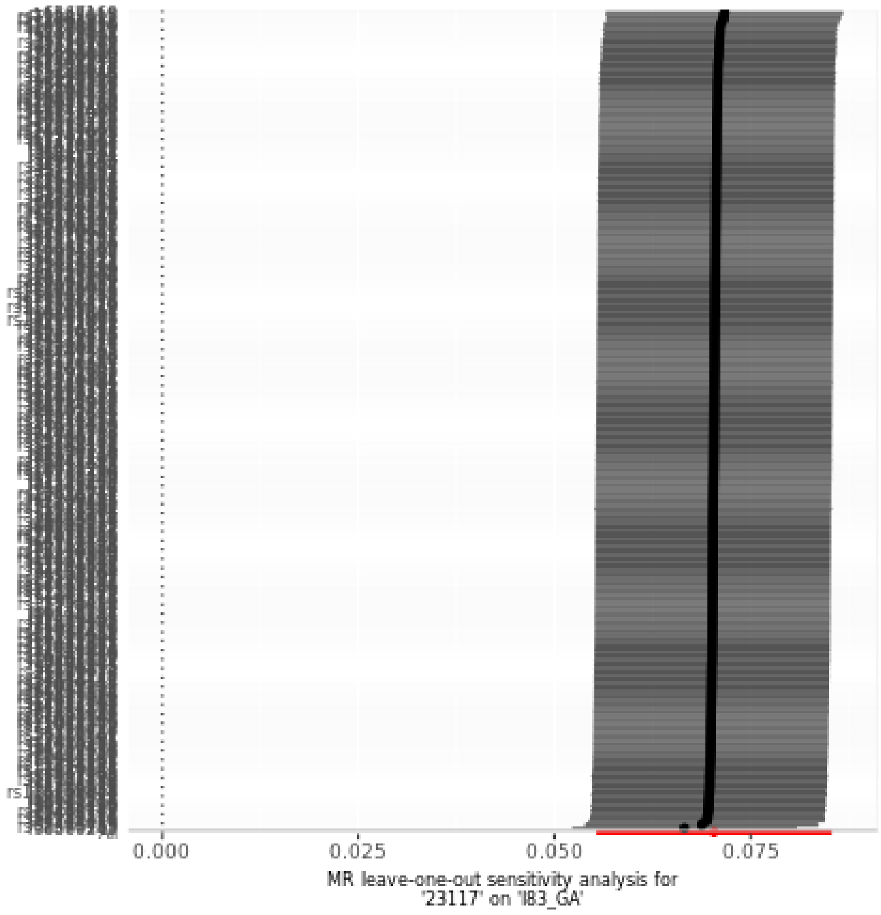	Leave-one-out plot 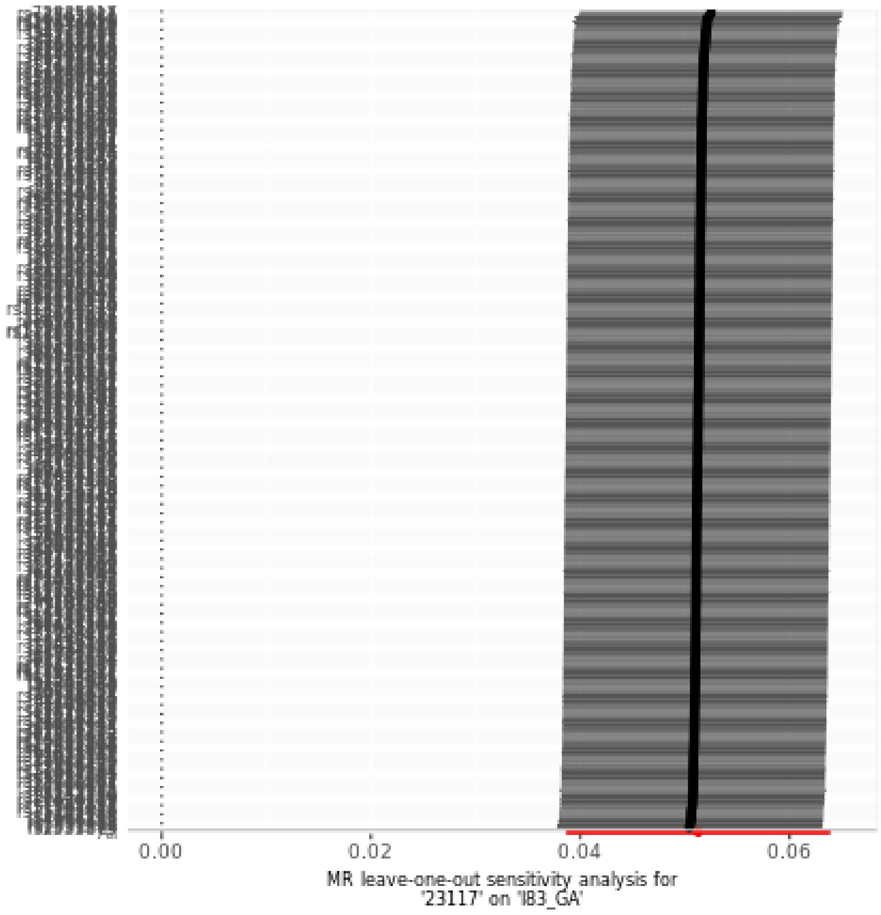

Funnel plot

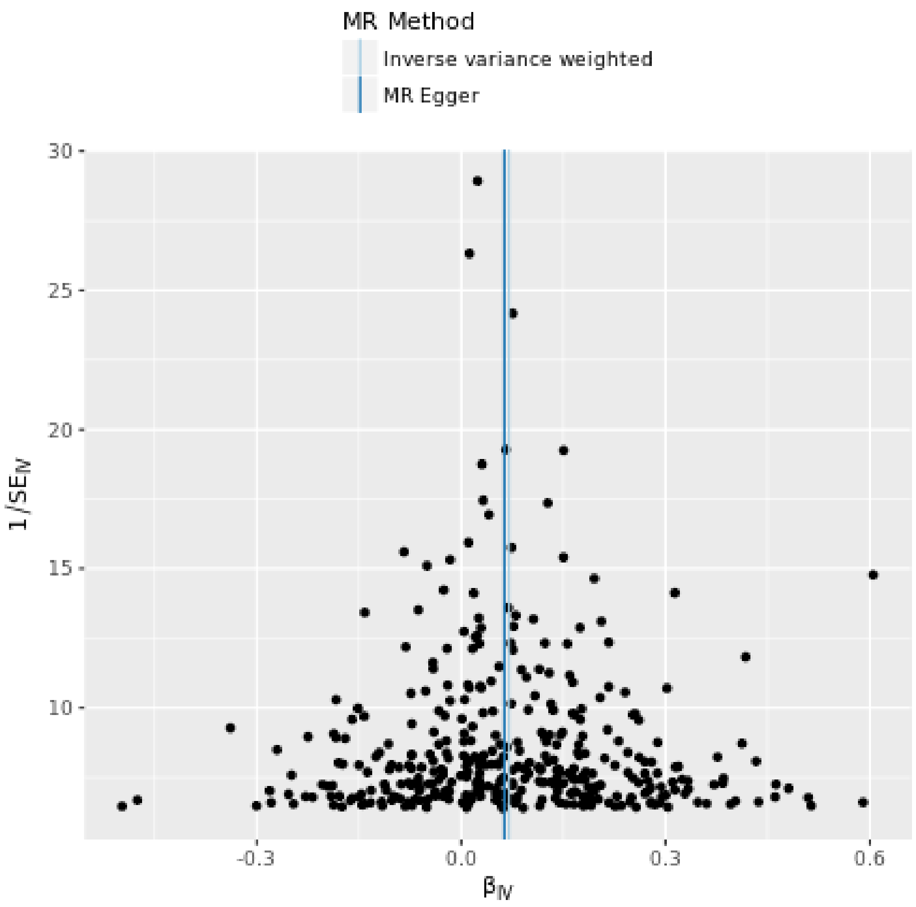

Funnel plot

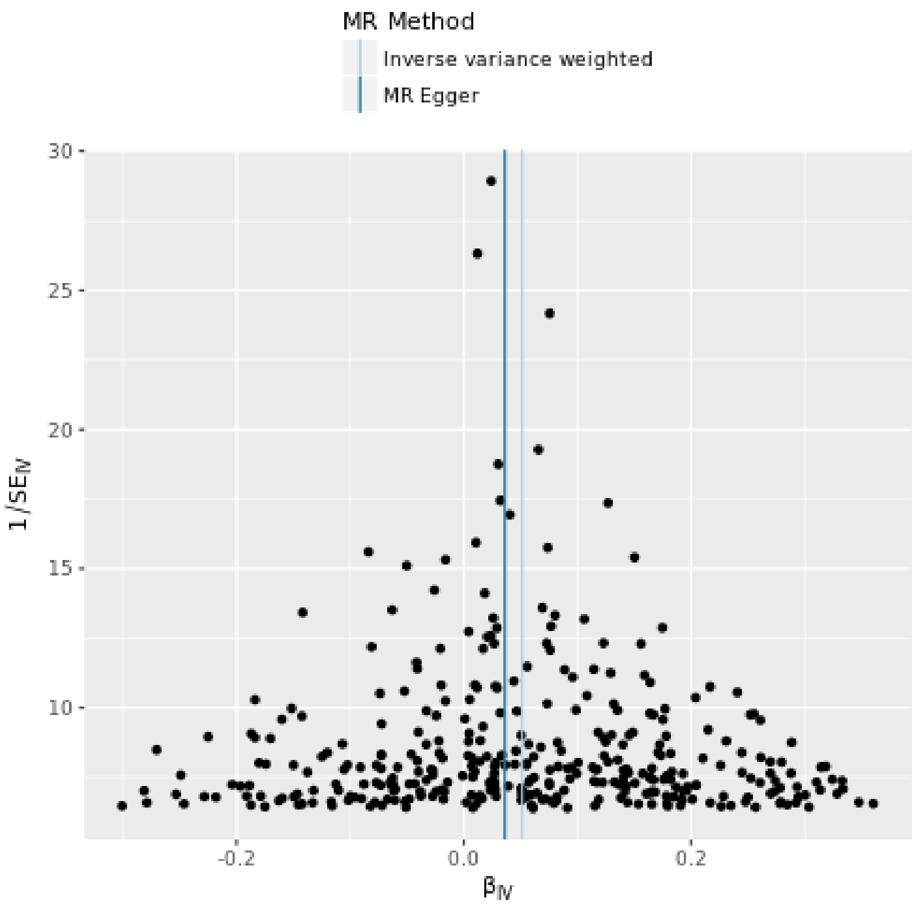

Whole body fat-free mass

<table>
<tr><td>Before removing instruments</td><td>After removing instruments</td></tr>
<tr><td>Leave-one-out plot

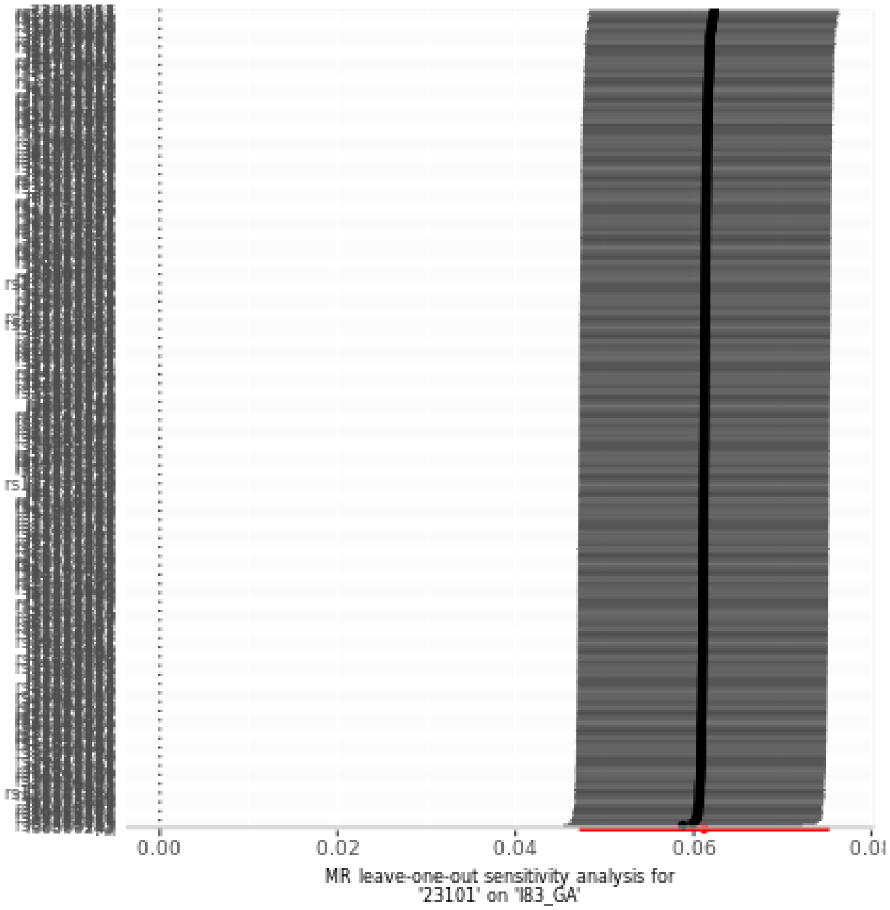

</td><td>Leave-one-out plot

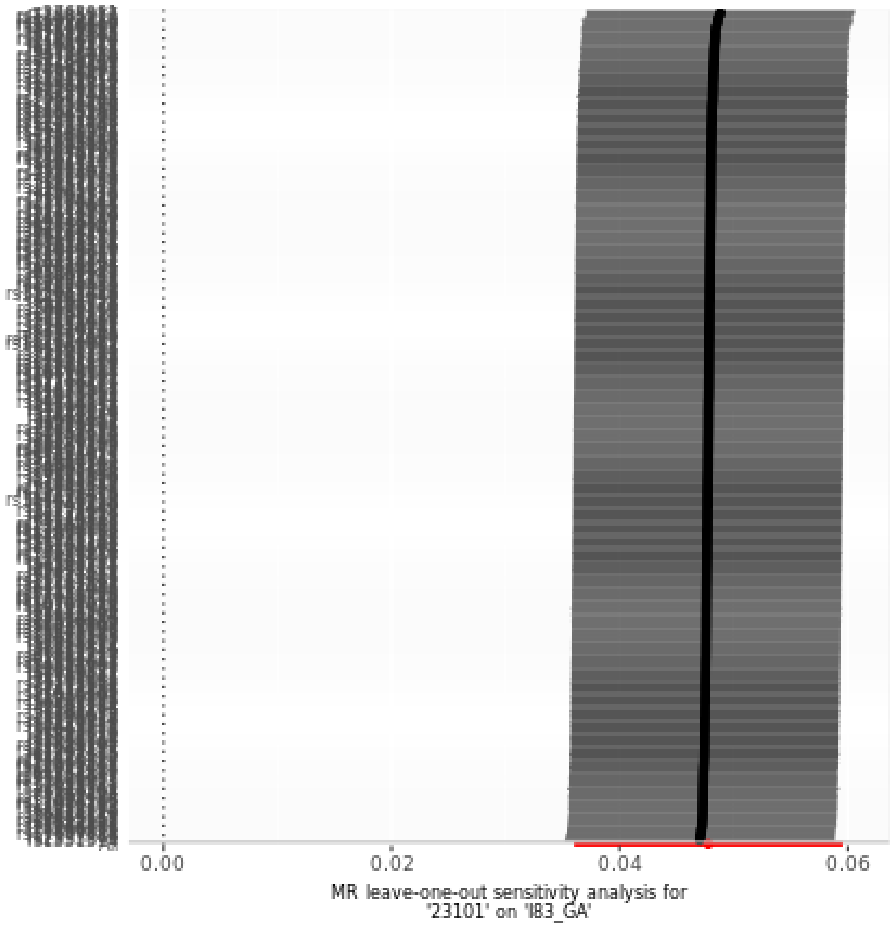

</td></tr>
</table>

Funnel plot

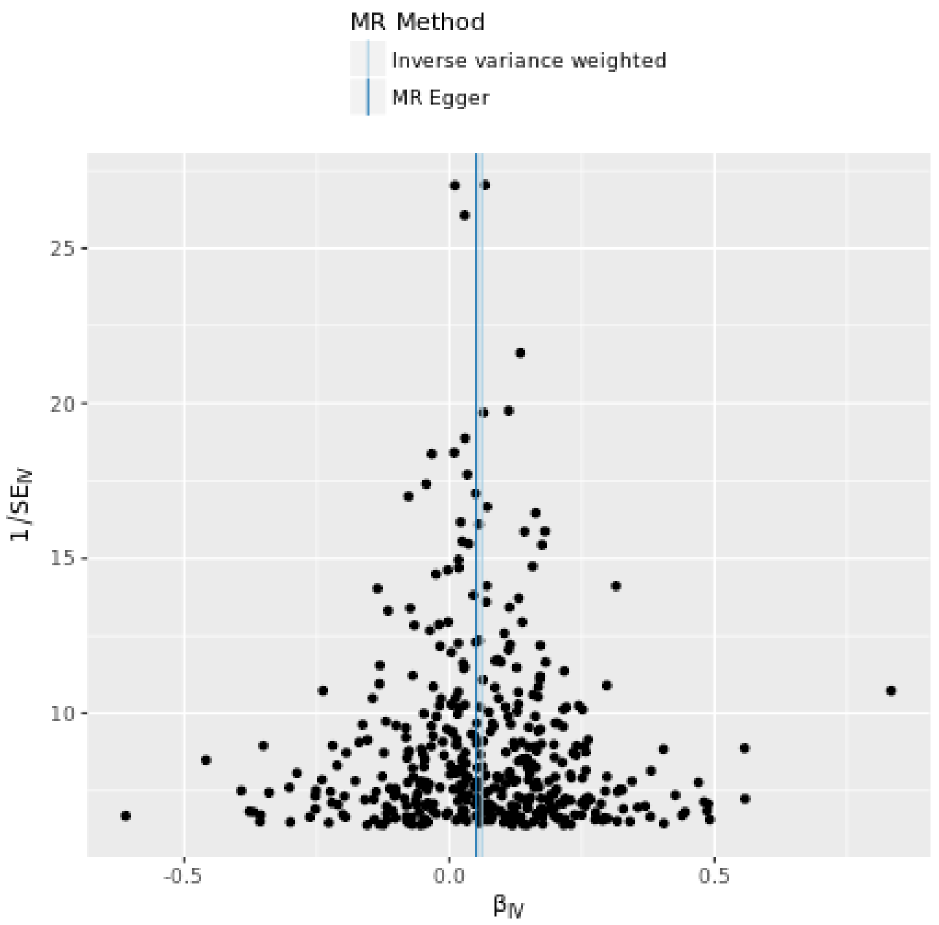

Funnel plot

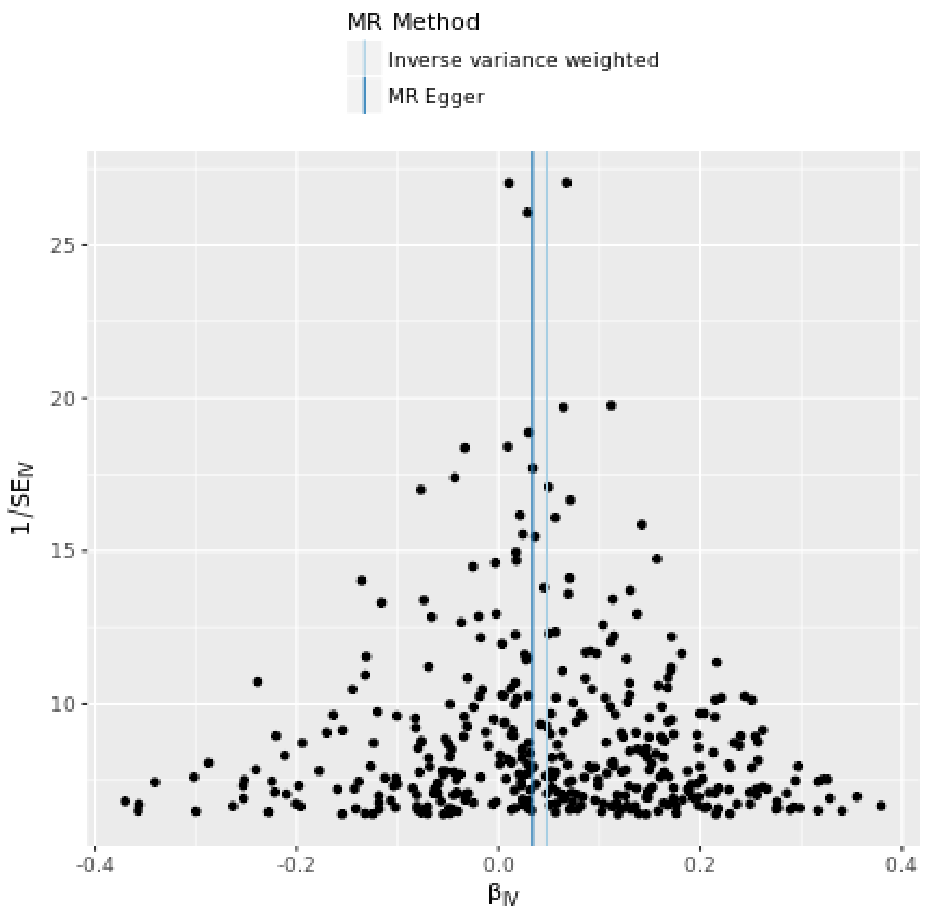

Leg predicted mass (left)

Before removing instruments	After removing instruments
Leave-one-out plot<br>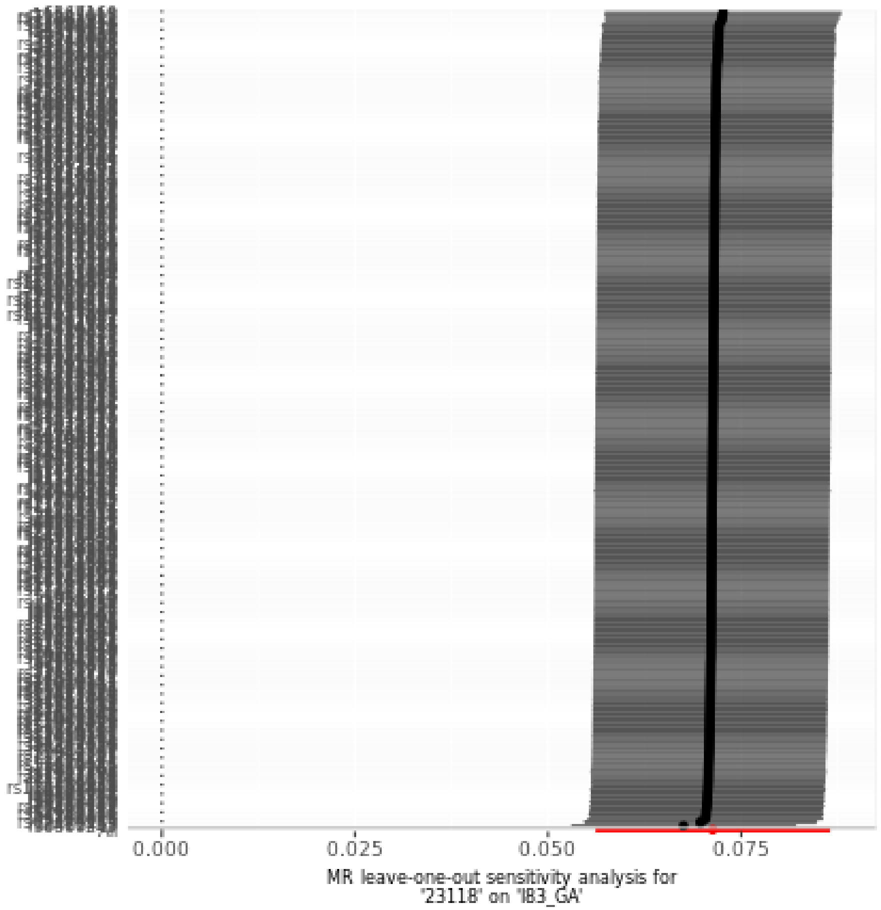 	Leave-one-out plot<br>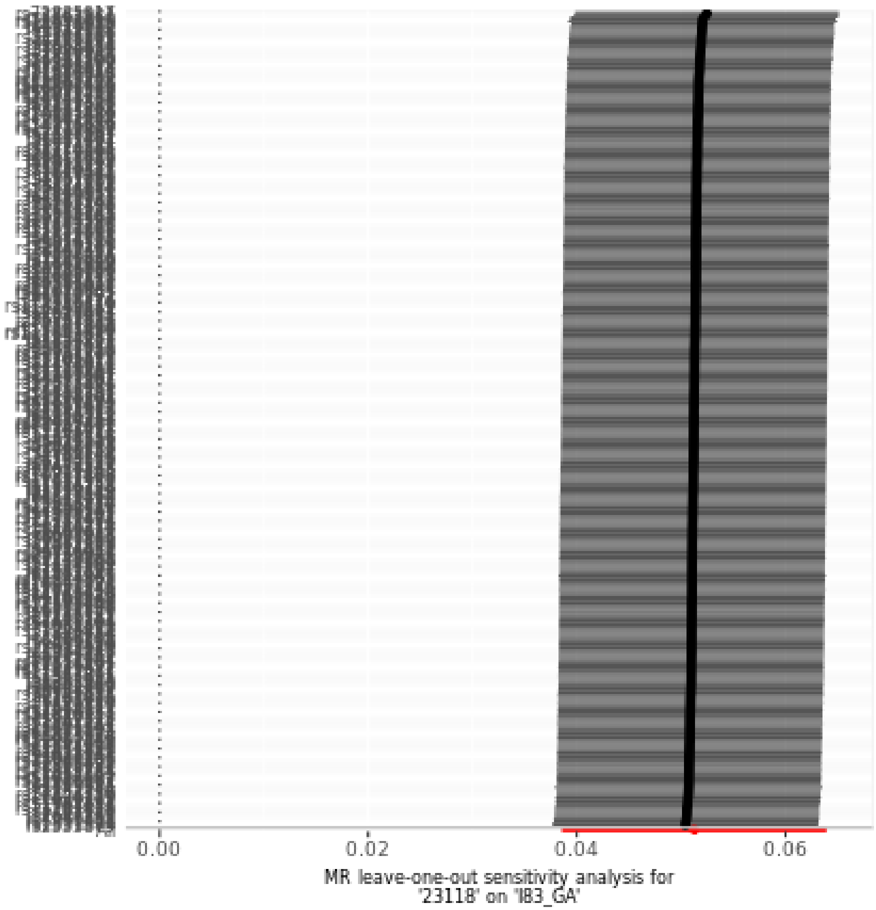

Funnel plot

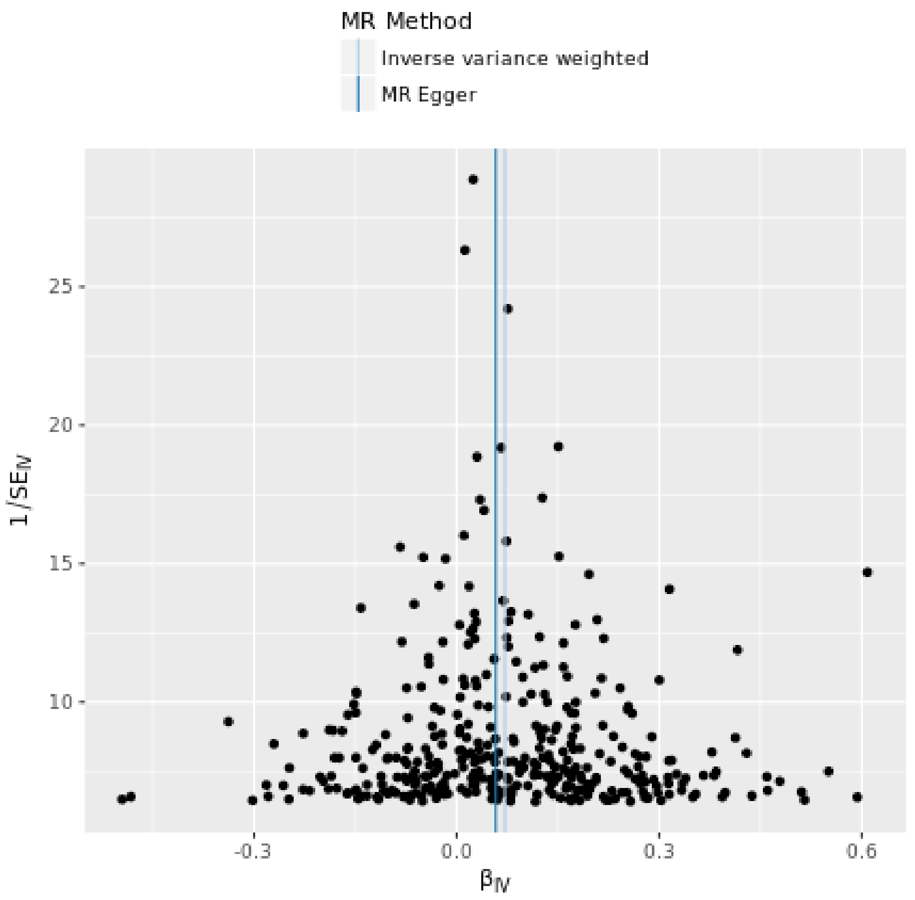

Funnel plot

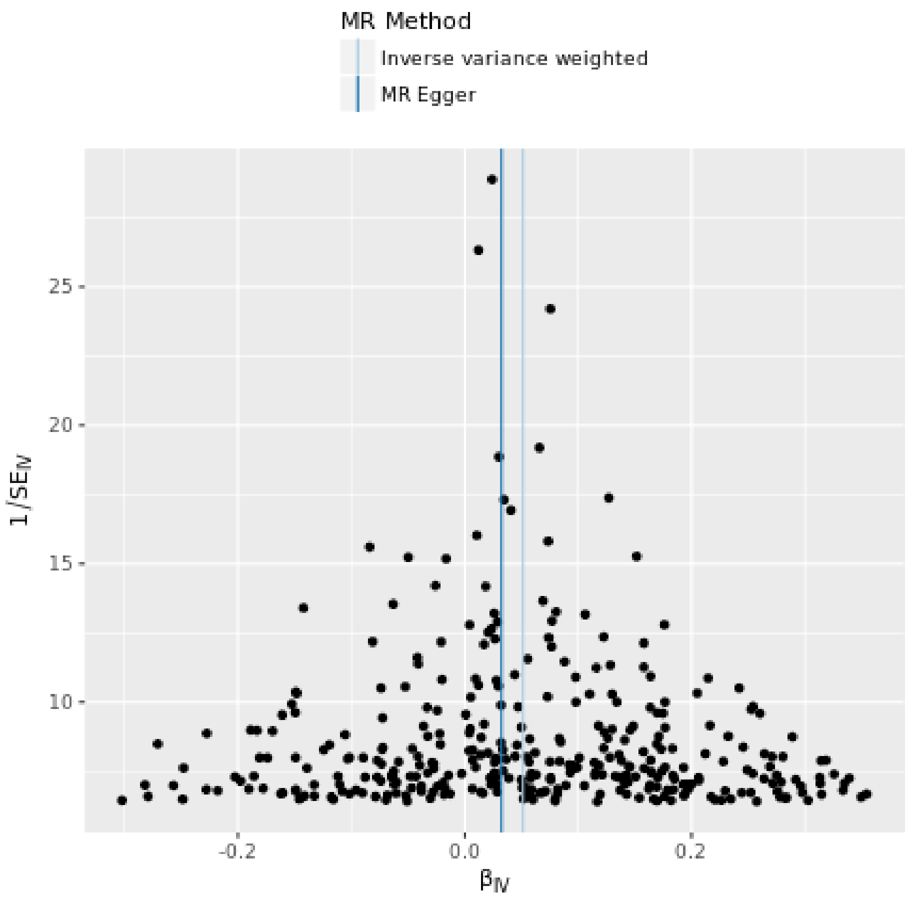

Basal metabolic rate

<table>
<tr><td>Before removing instruments</td><td>After removing instruments</td></tr>
<tr><td>Leave-one-out plot
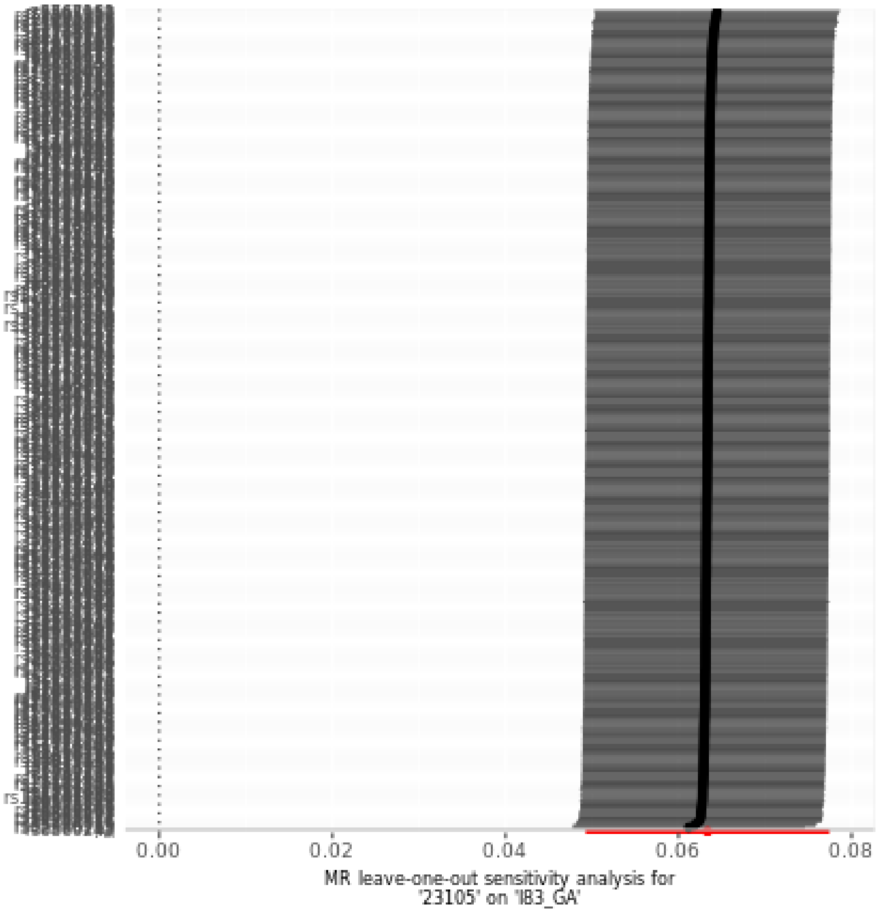
</td><td>Leave-one-out plot
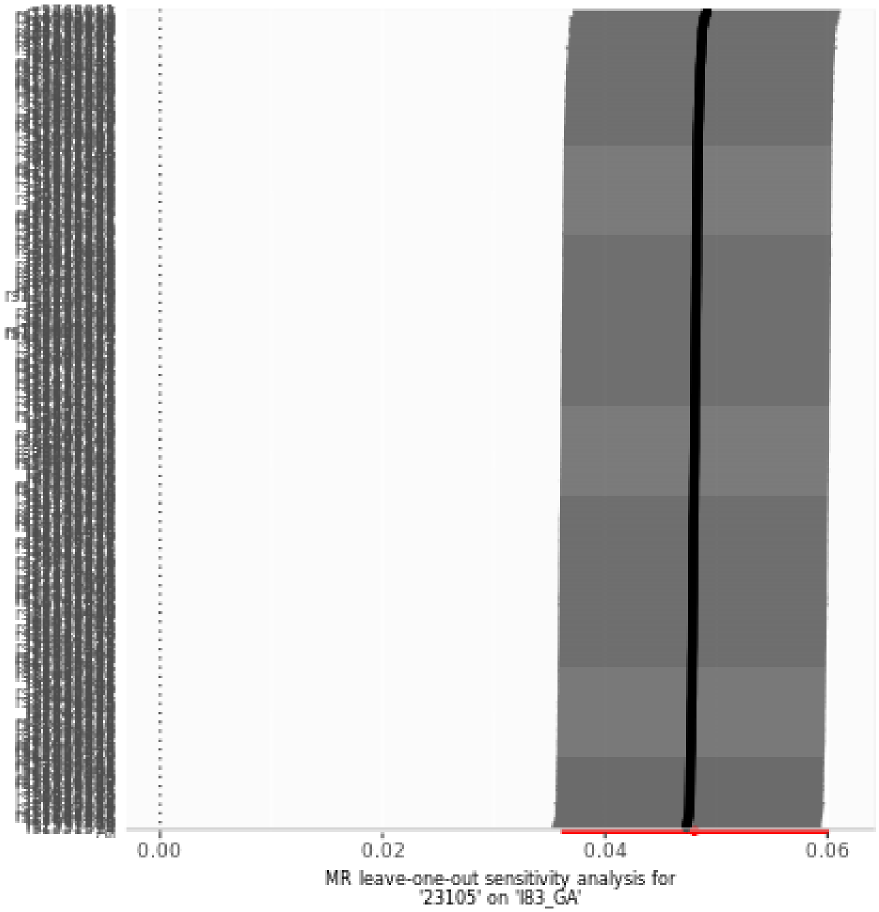
</td></tr>
</table>

Funnel plot

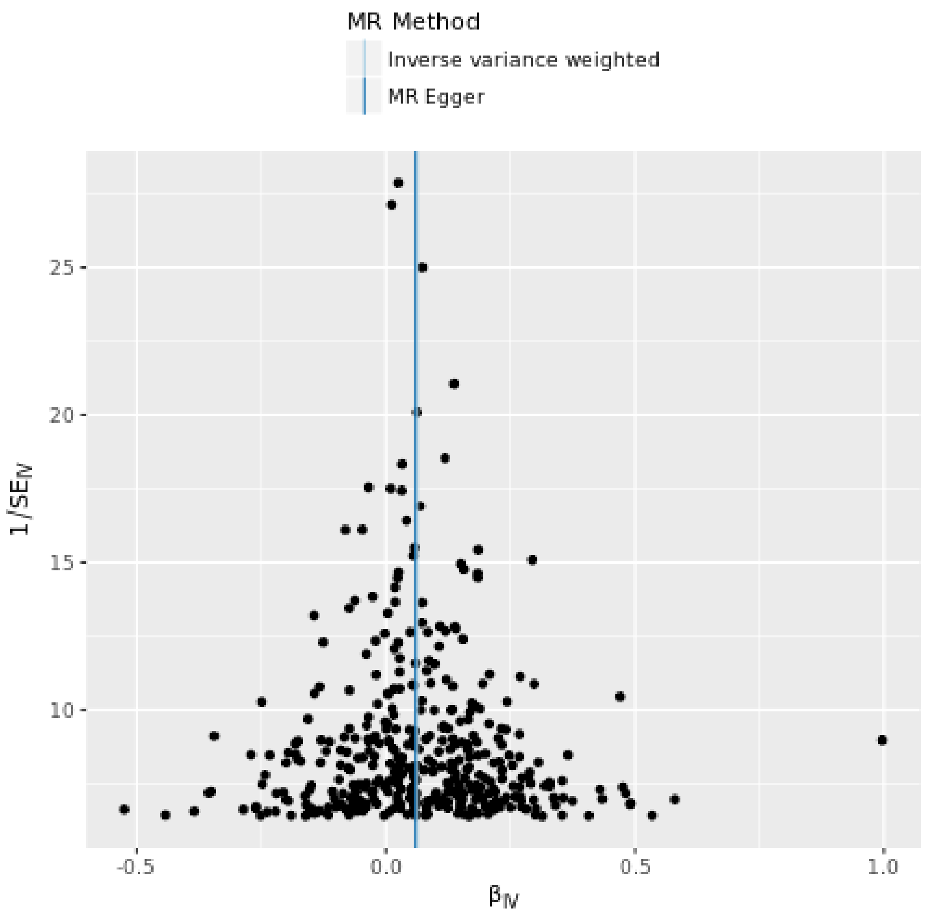

Funnel plot

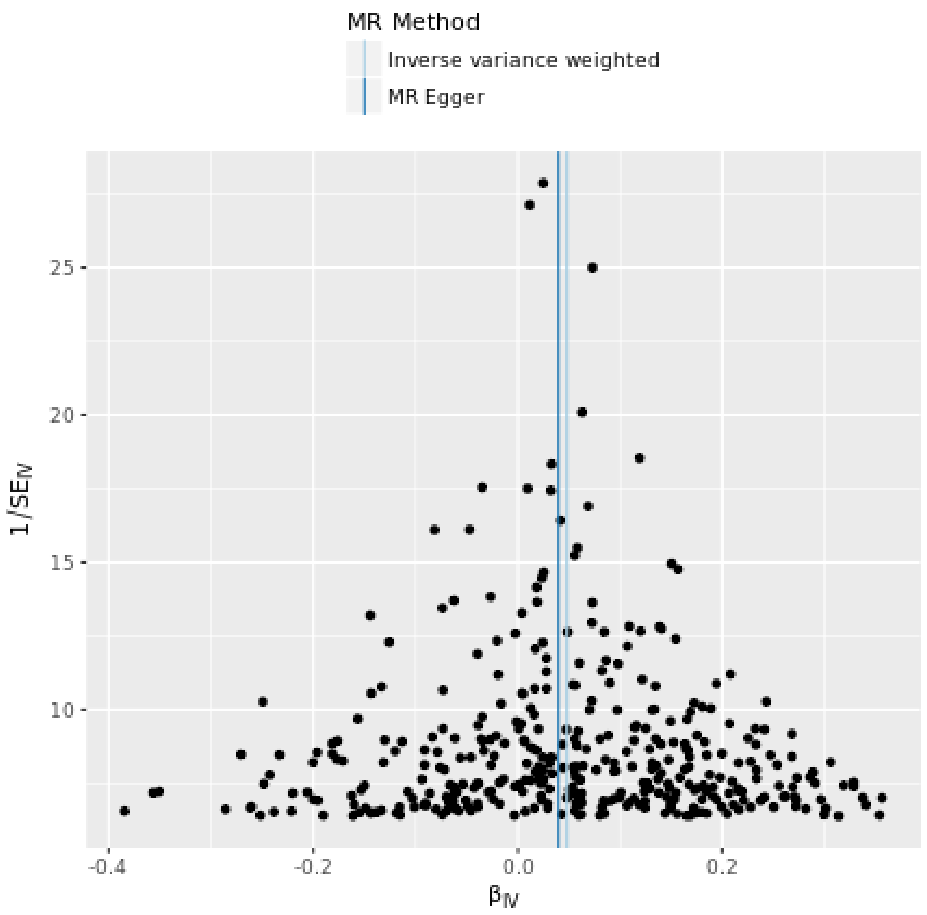

Trunk fat mass

Before removing instruments	After removing instruments
Leave-one-out plot 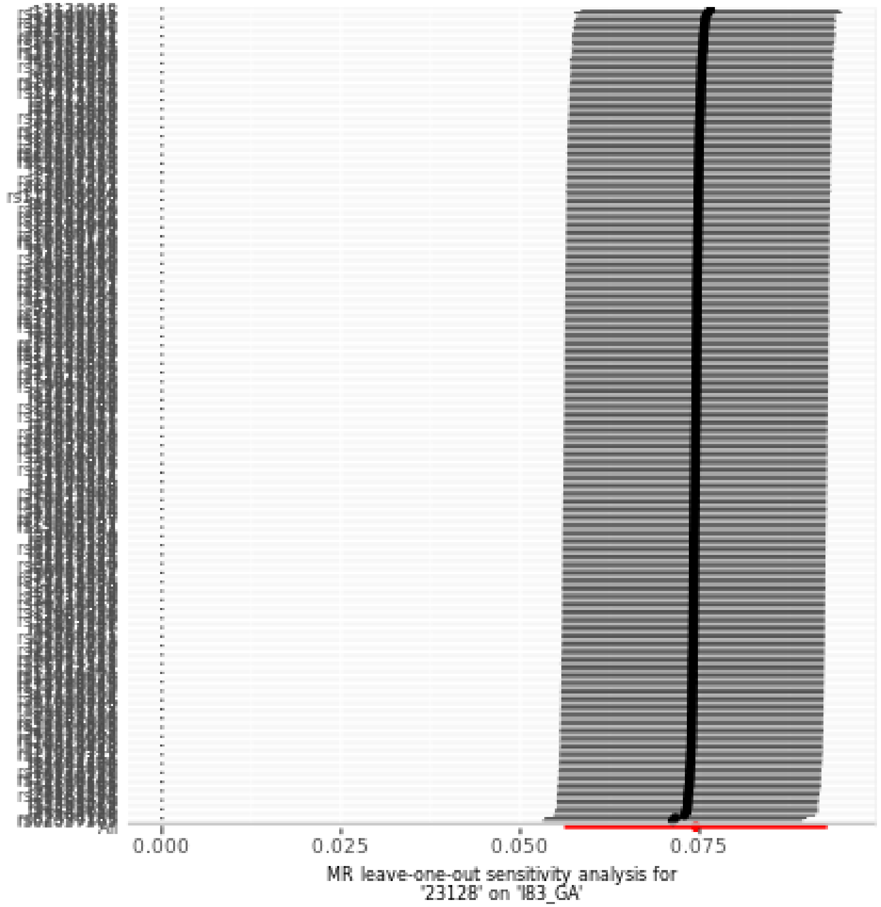	Leave-one-out plot 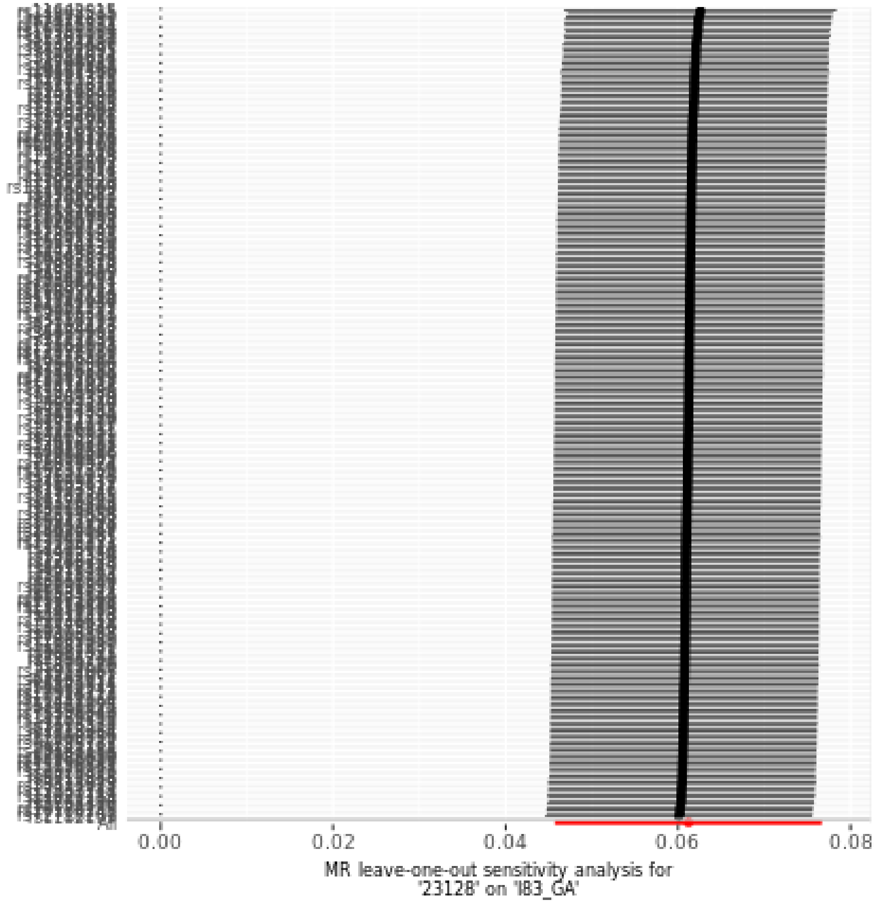

Funnel plot

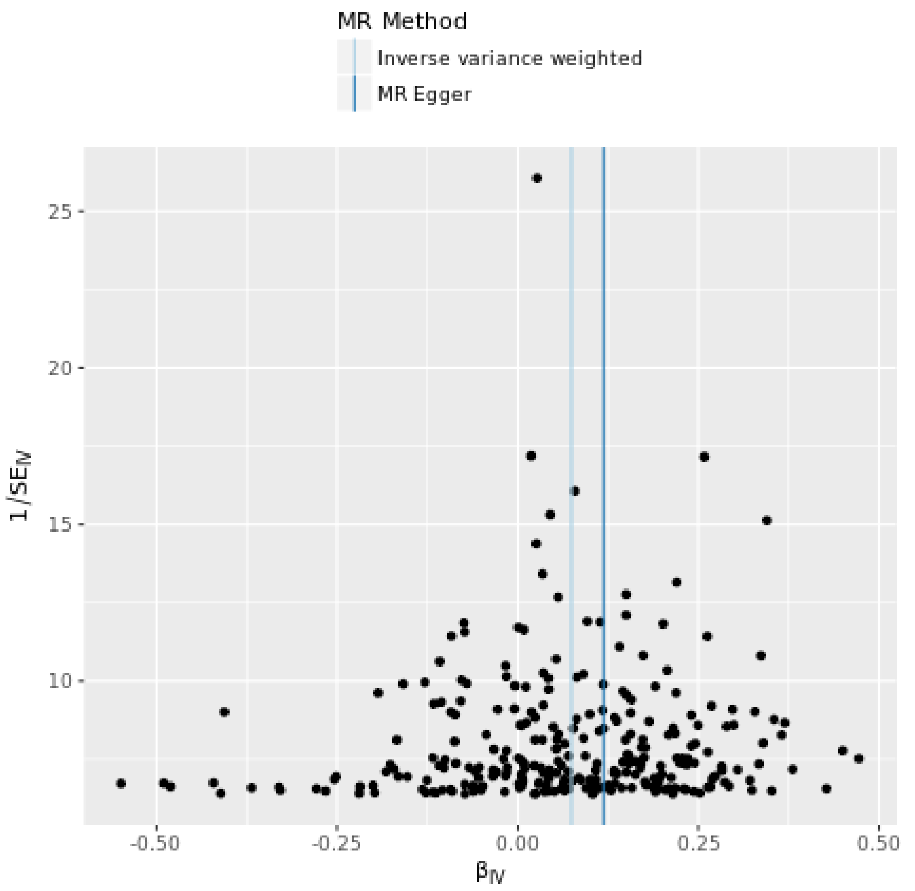

Funnel plot

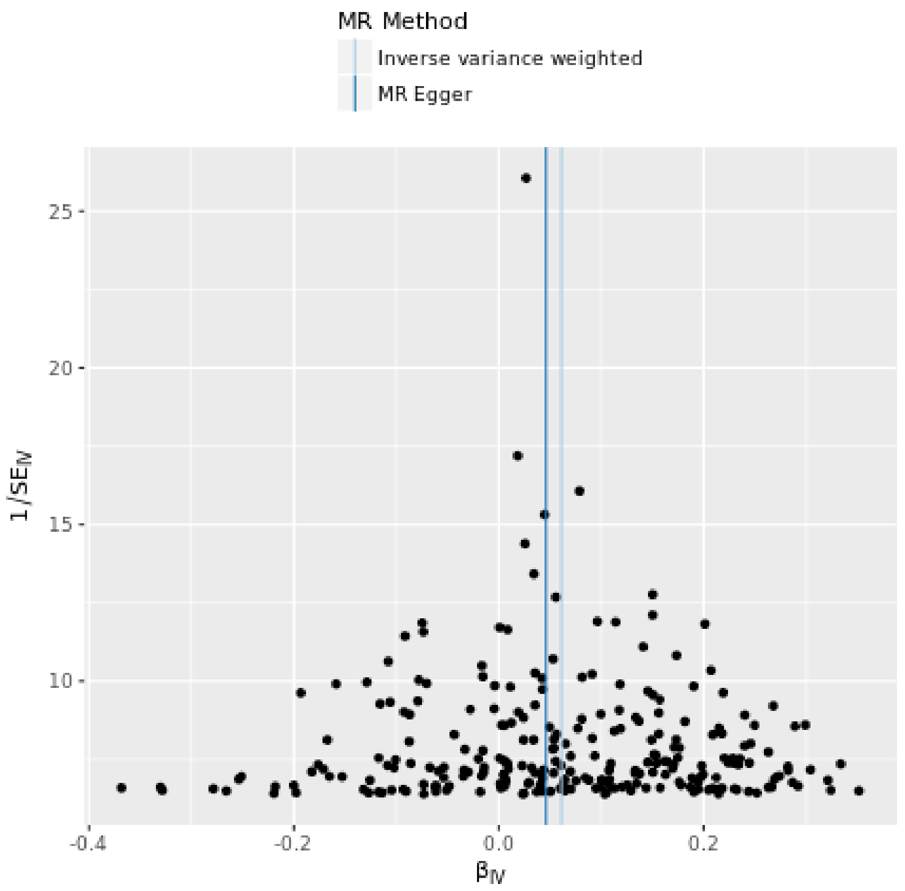

Trunk fat-free mass

<table>
<tr><td>Before removing instruments</td><td>After removing instruments</td></tr>
<tr><td>Leave-one-out plot
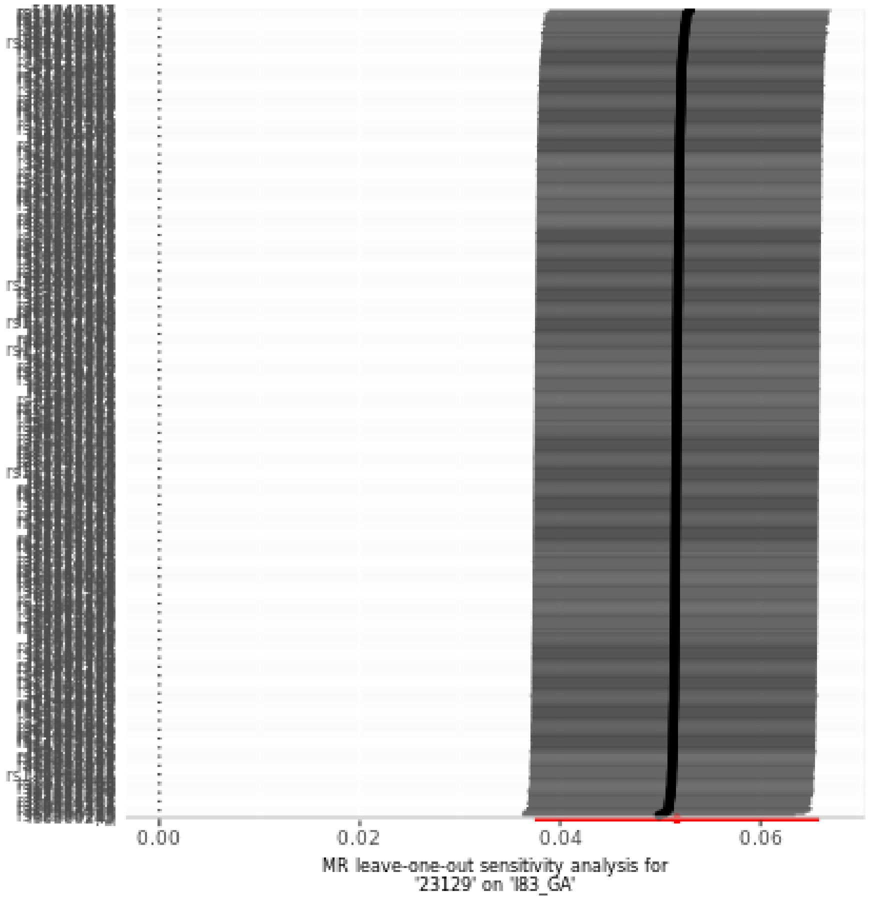
</td><td>Leave-one-out plot
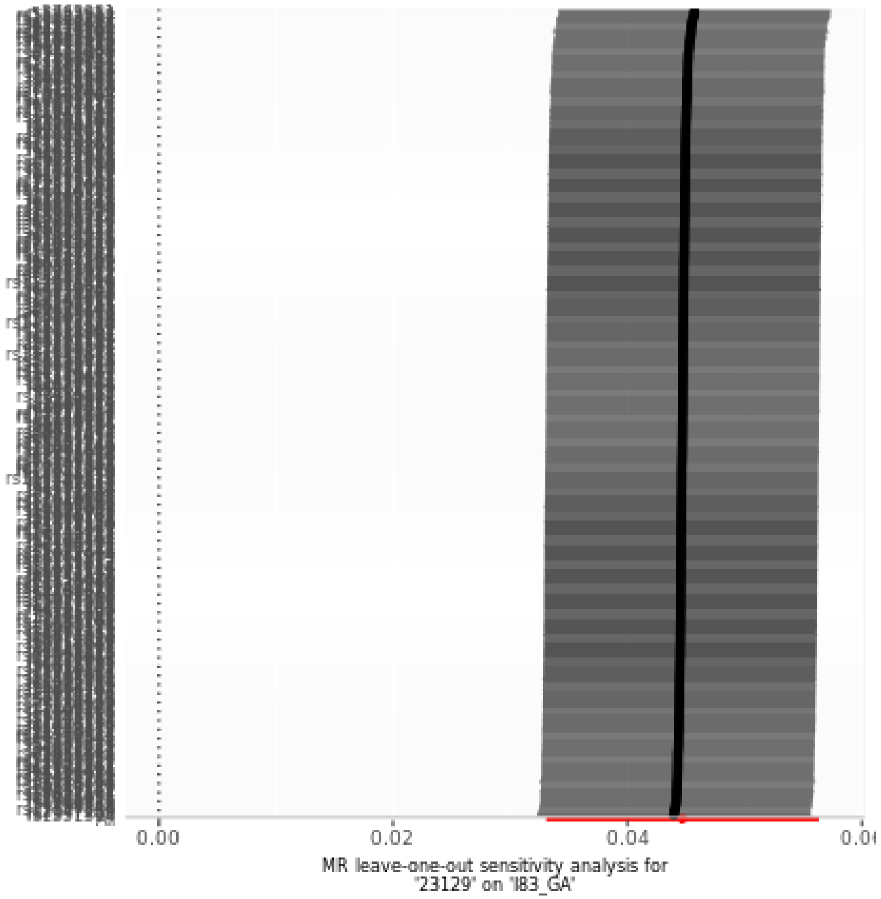
</td></tr>
</table>

Funnel plot

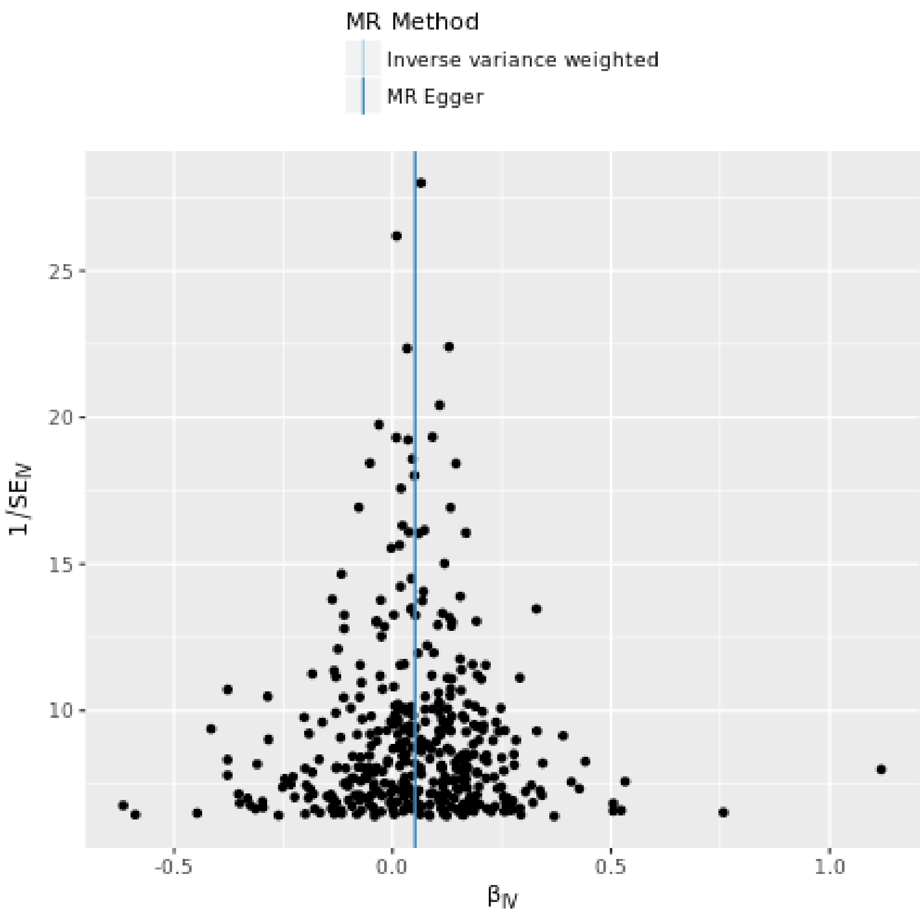

Funnel plot

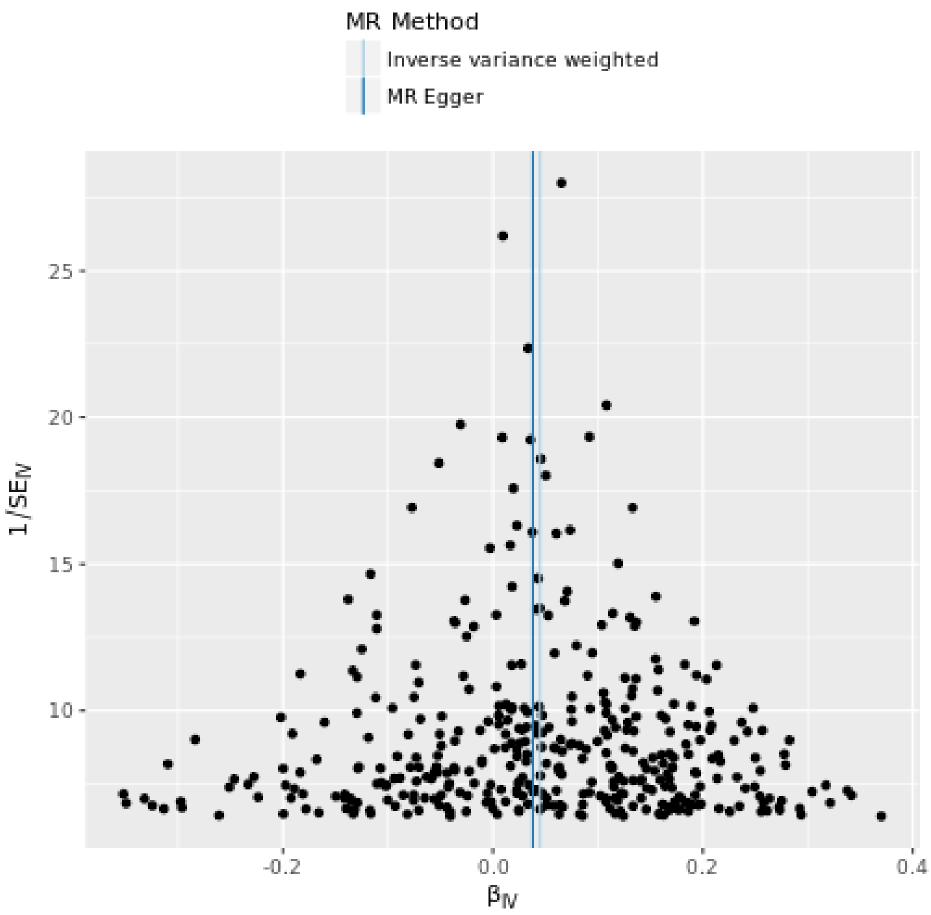

Weight

Before removing instruments	After removing instruments
Leave-one-out plot 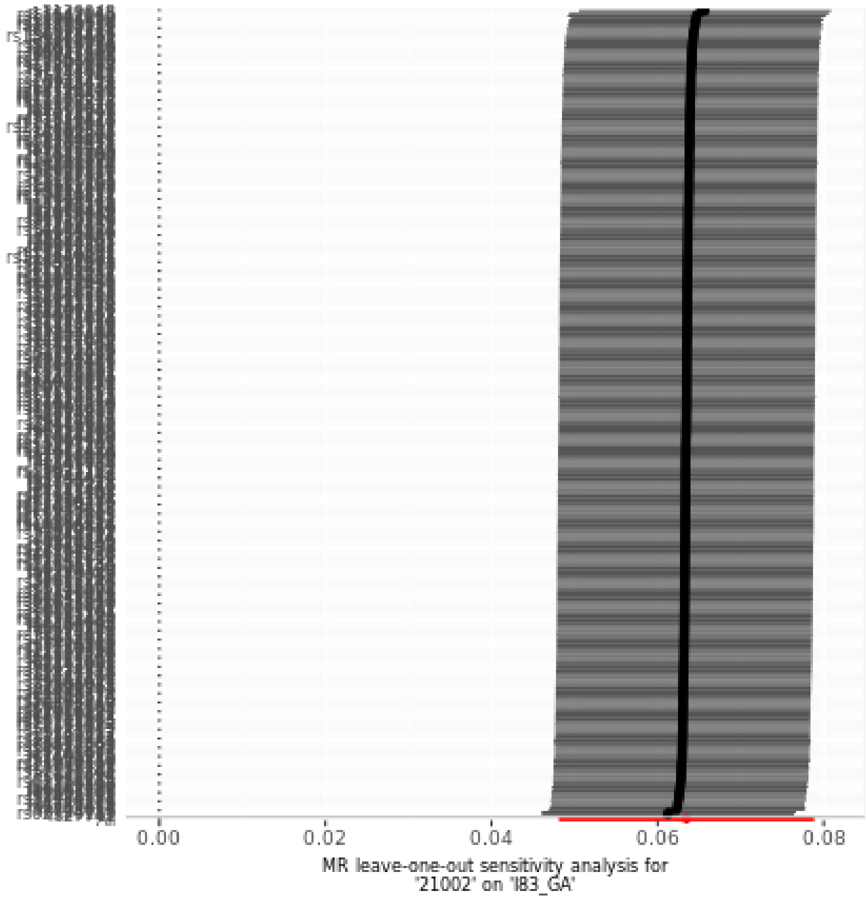	Leave-one-out plot 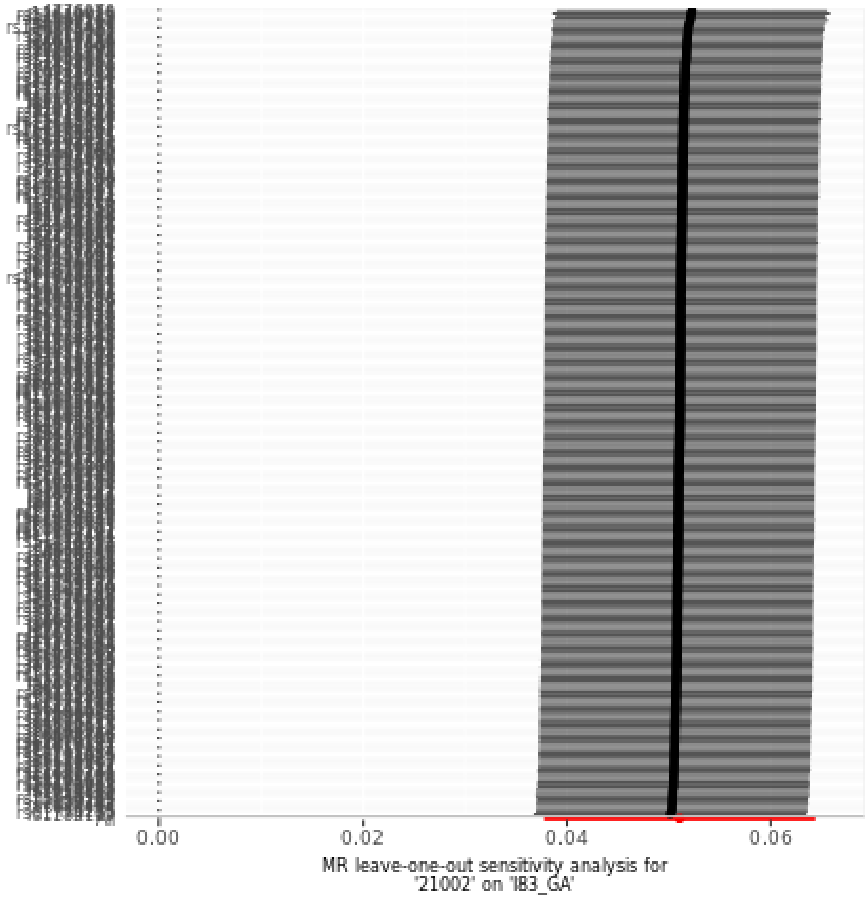

Funnel plot	Funnel plot
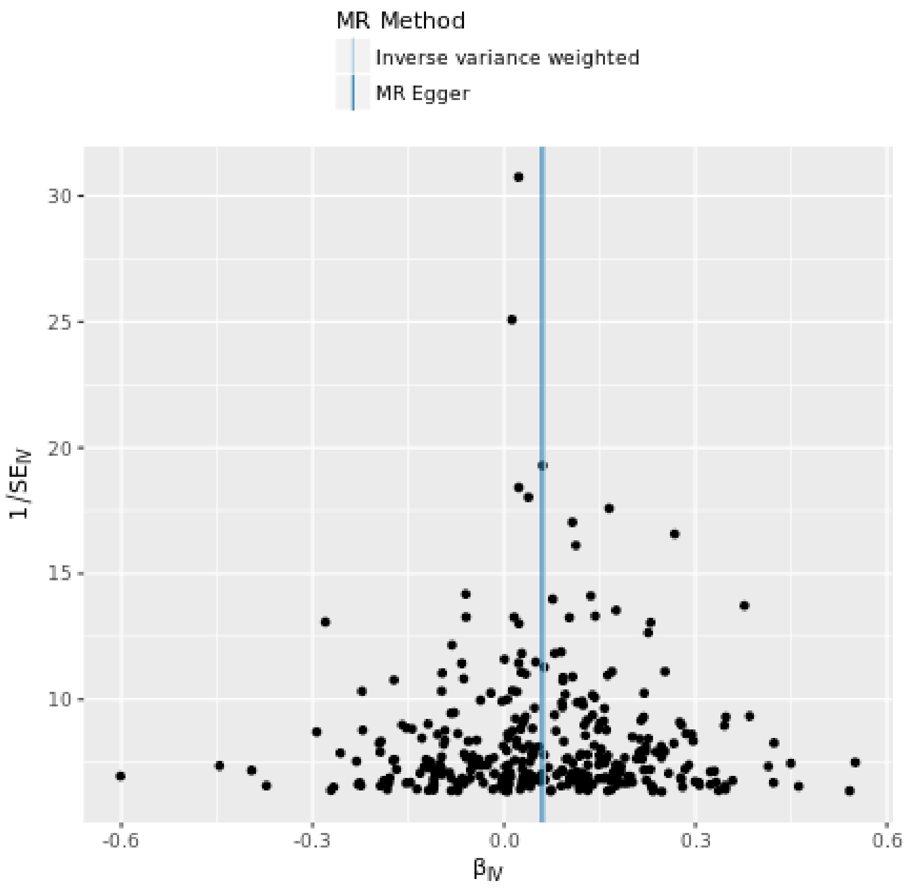	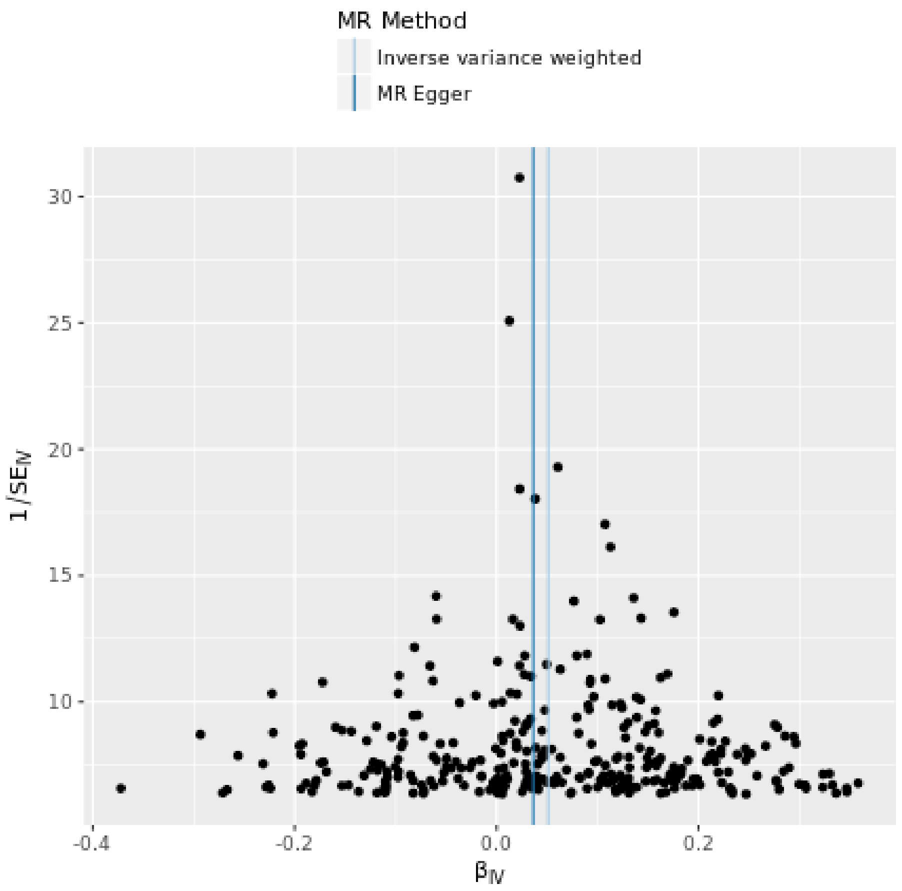

Whole body water mass

<table>
<tr><td>Before removing instruments</td><td>After removing instruments</td></tr>
<tr><td>Leave-one-out plot
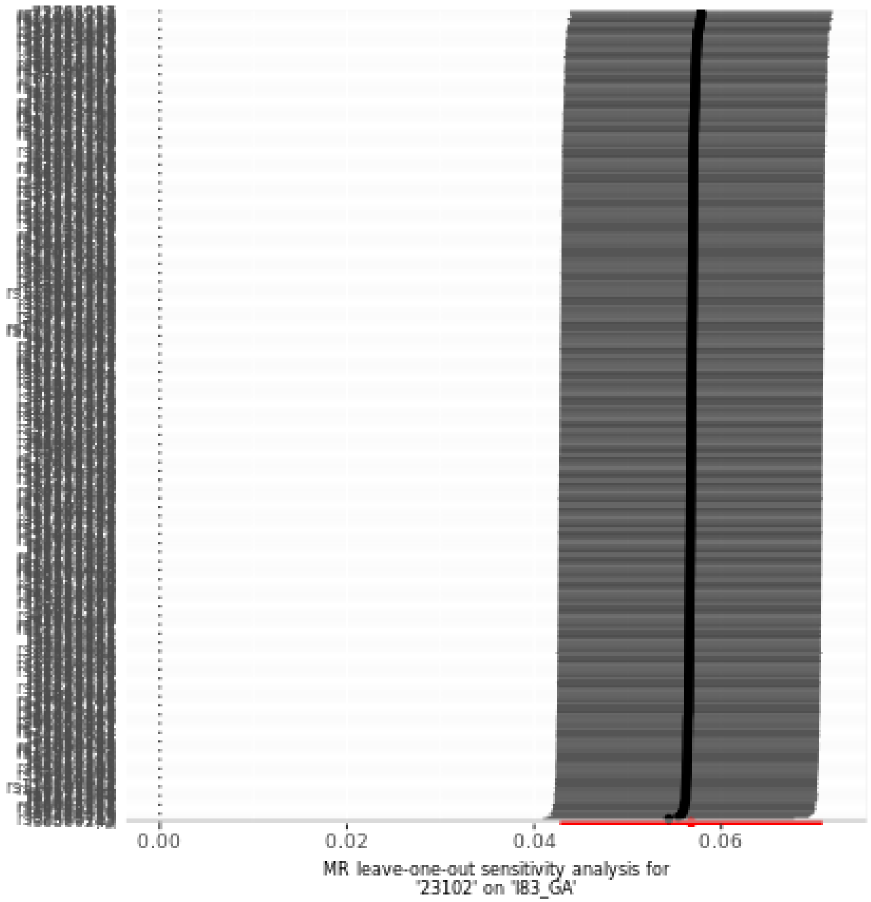
</td><td>Leave-one-out plot
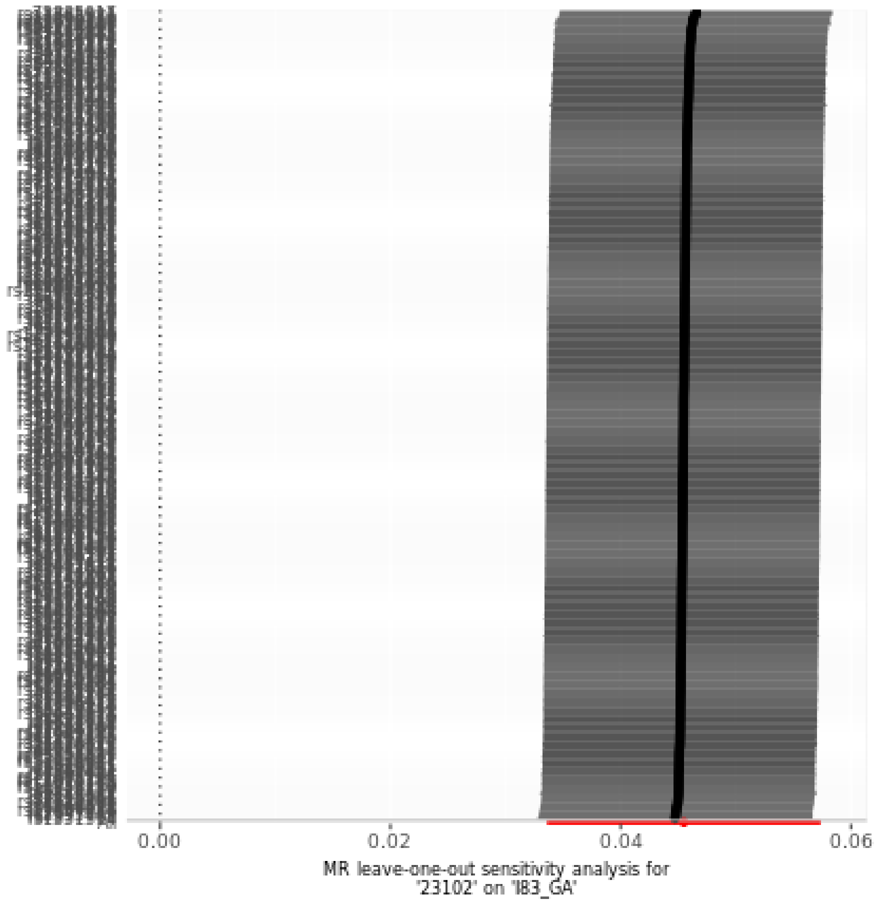
</td></tr>
</table>

Funnel plot	Funnel plot
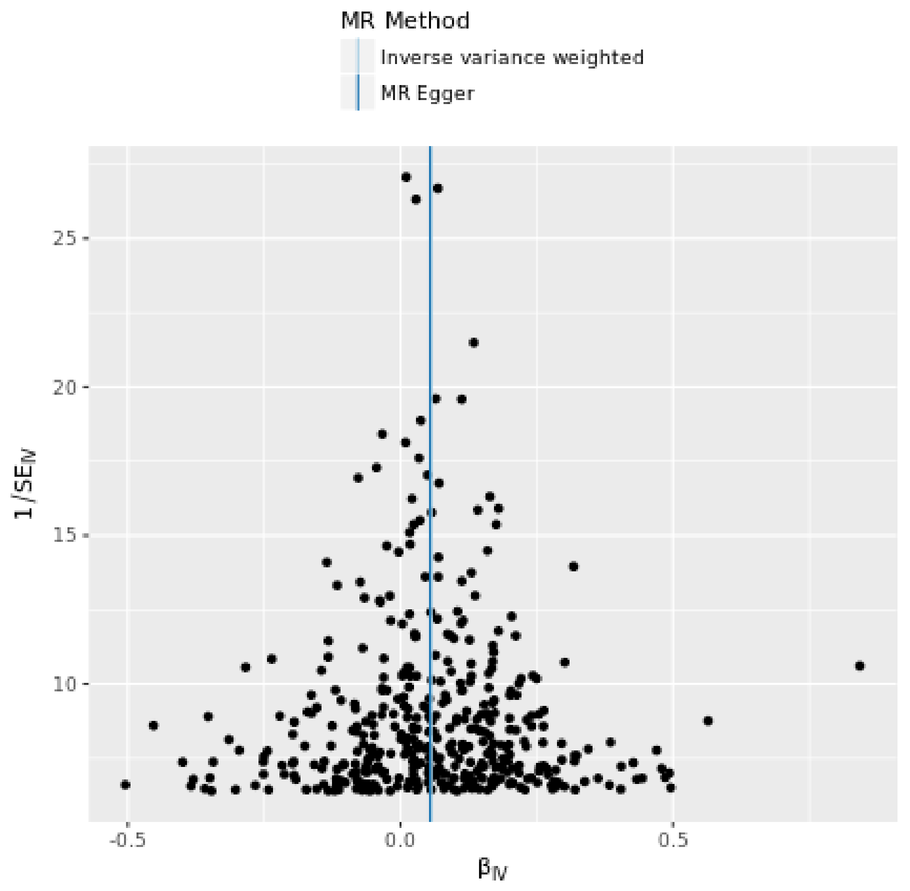	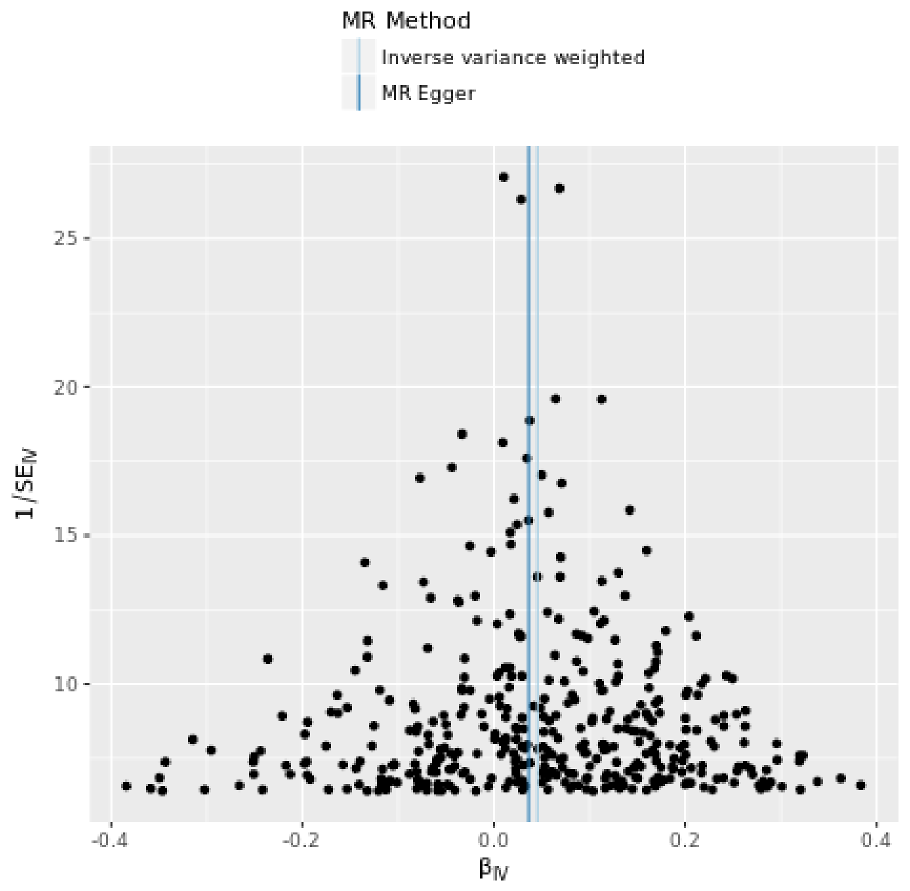

Leg predicted mass (right)

Before removing instruments	After removing instruments
Leave-one-out plot<br>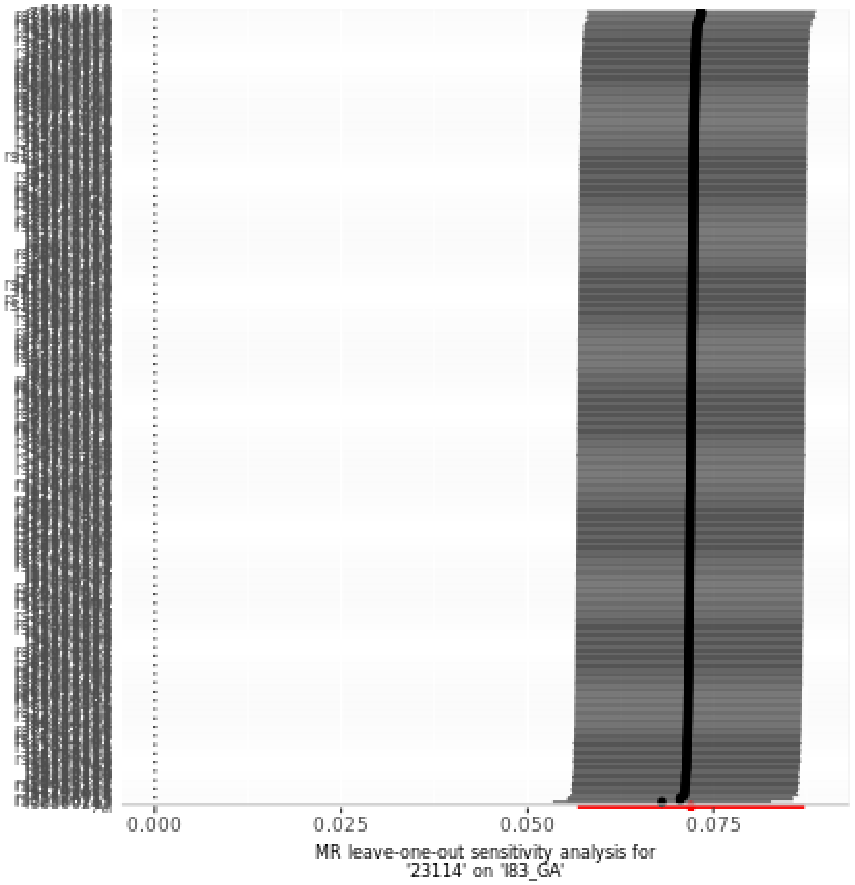 	Leave-one-out plot<br>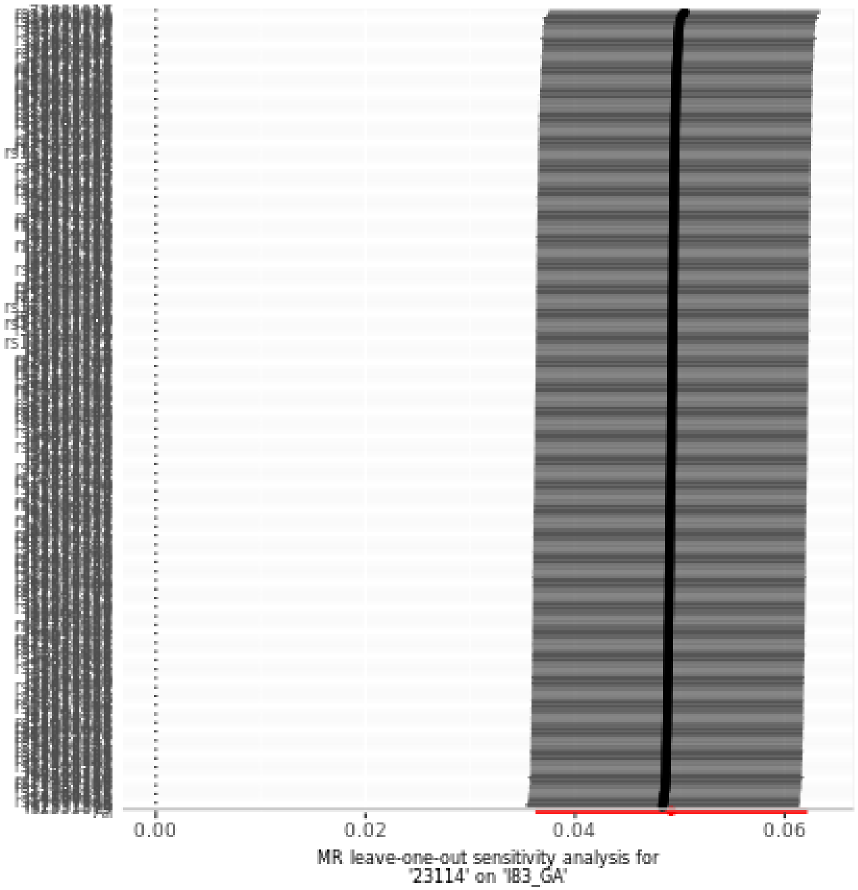

Funnel plot

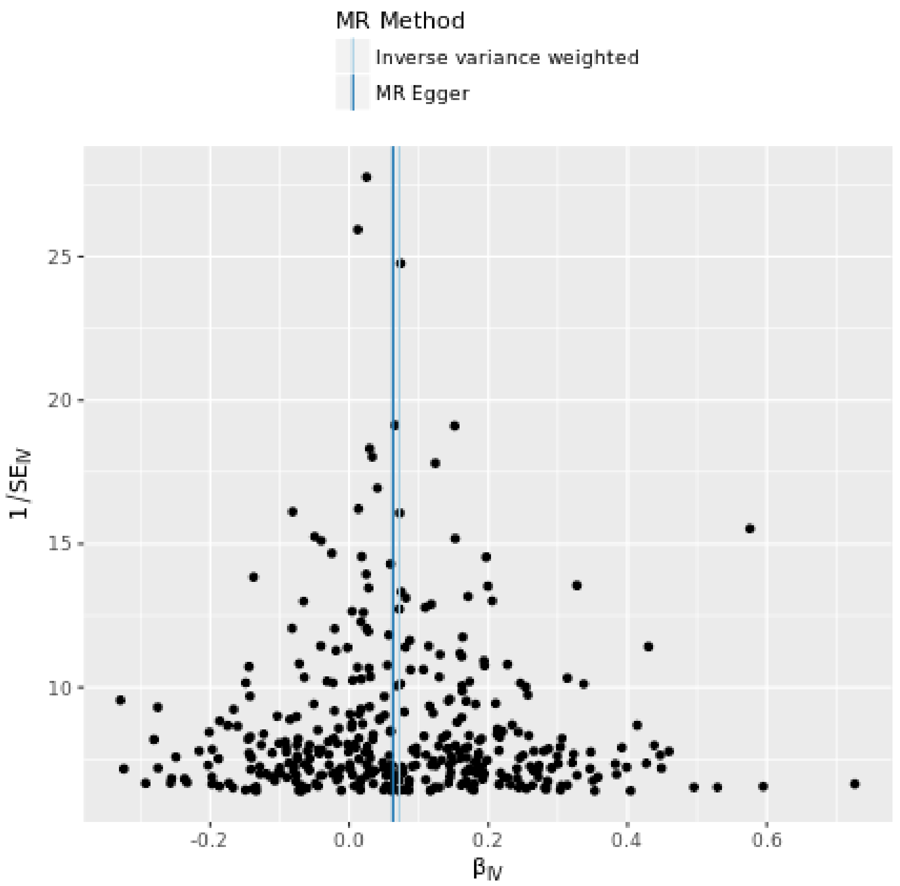

Funnel plot

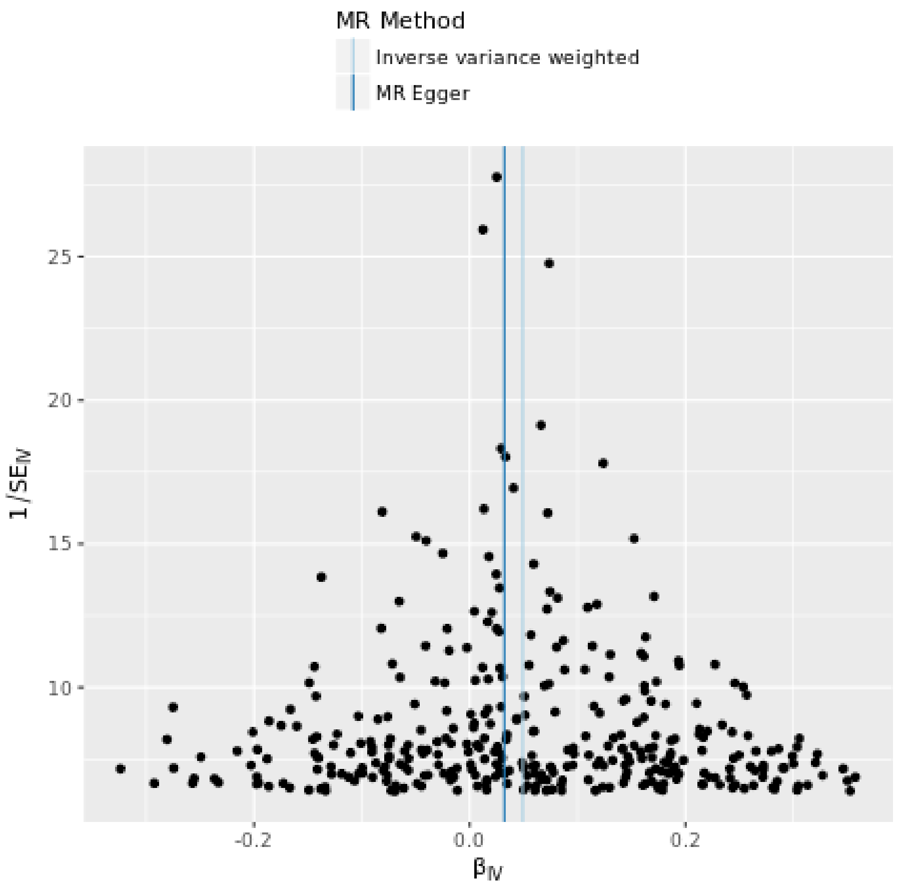

Leg fat-free mass (right)

<table>
<tr><td>Before removing instruments</td><td>After removing instruments</td></tr>
<tr><td>Leave-one-out plot
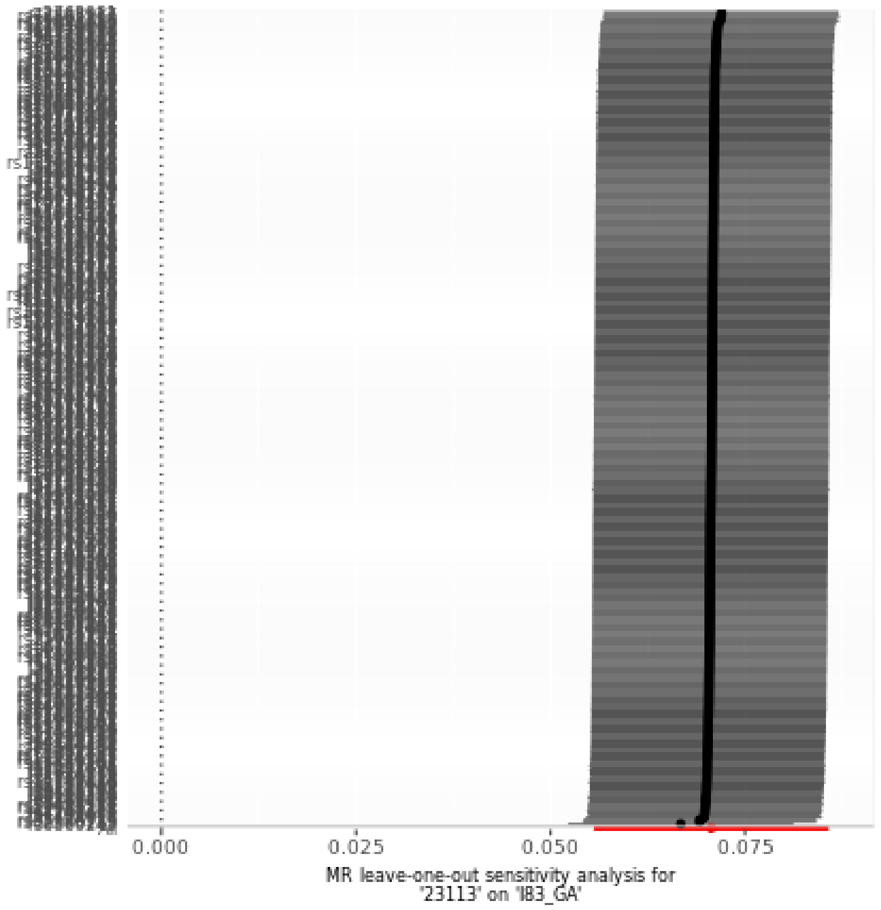
</td><td>Leave-one-out plot
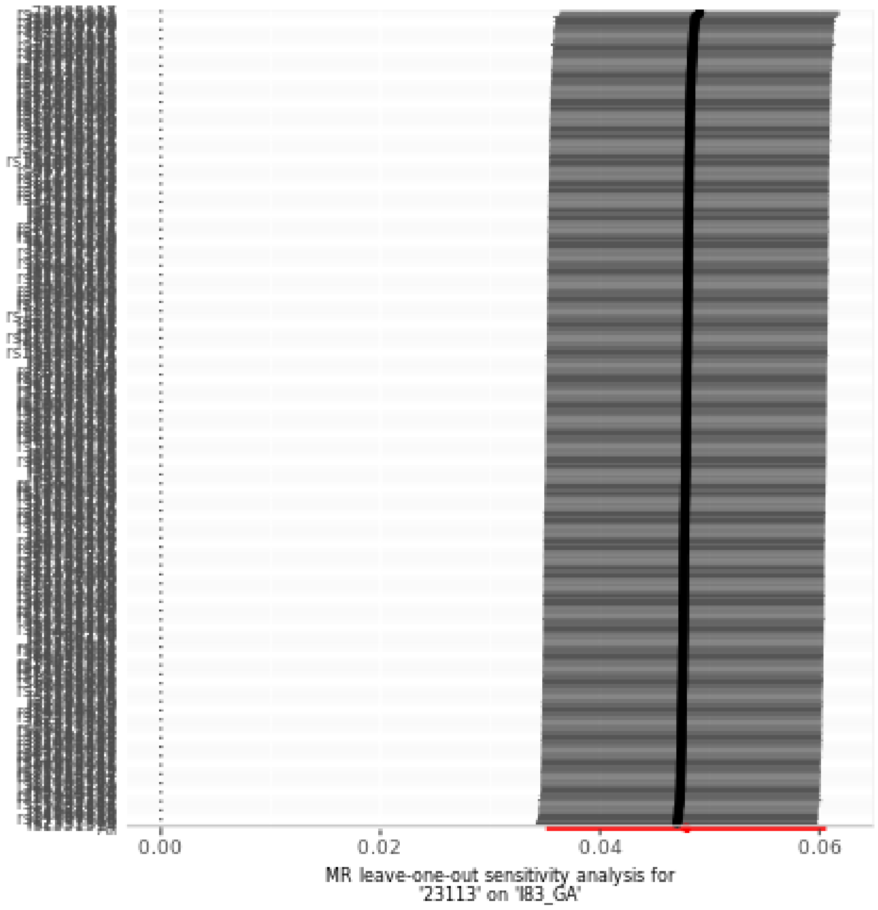
</td></tr>
</table>

Funnel plot

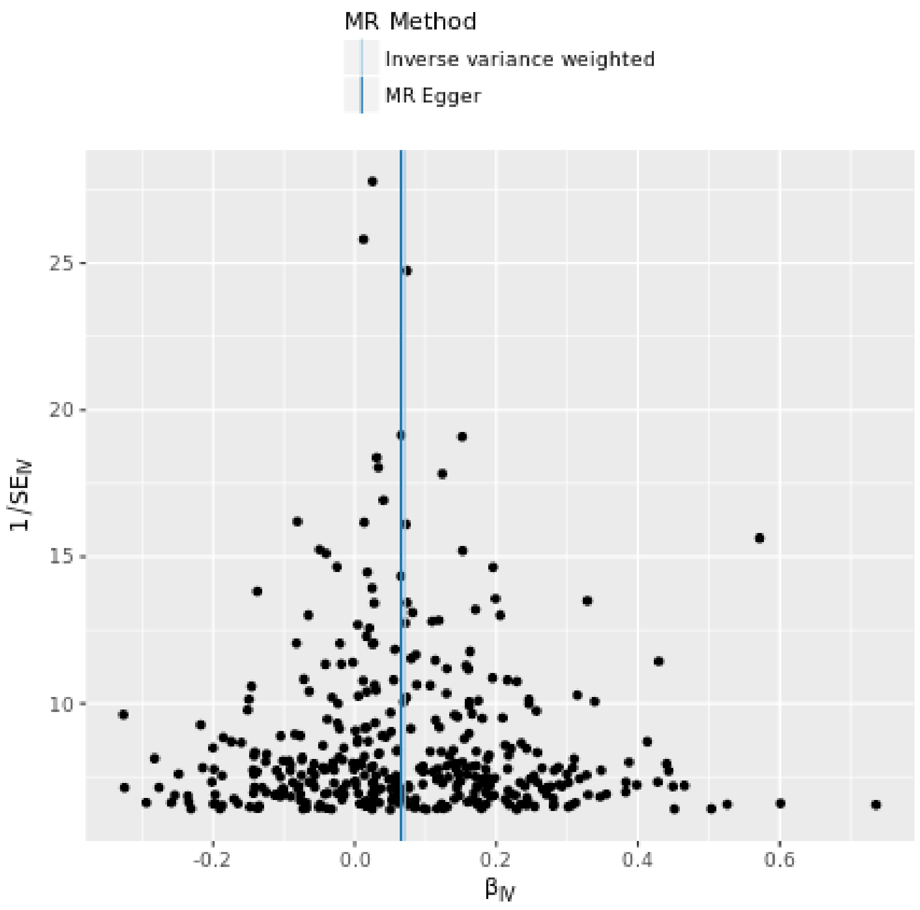

Funnel plot

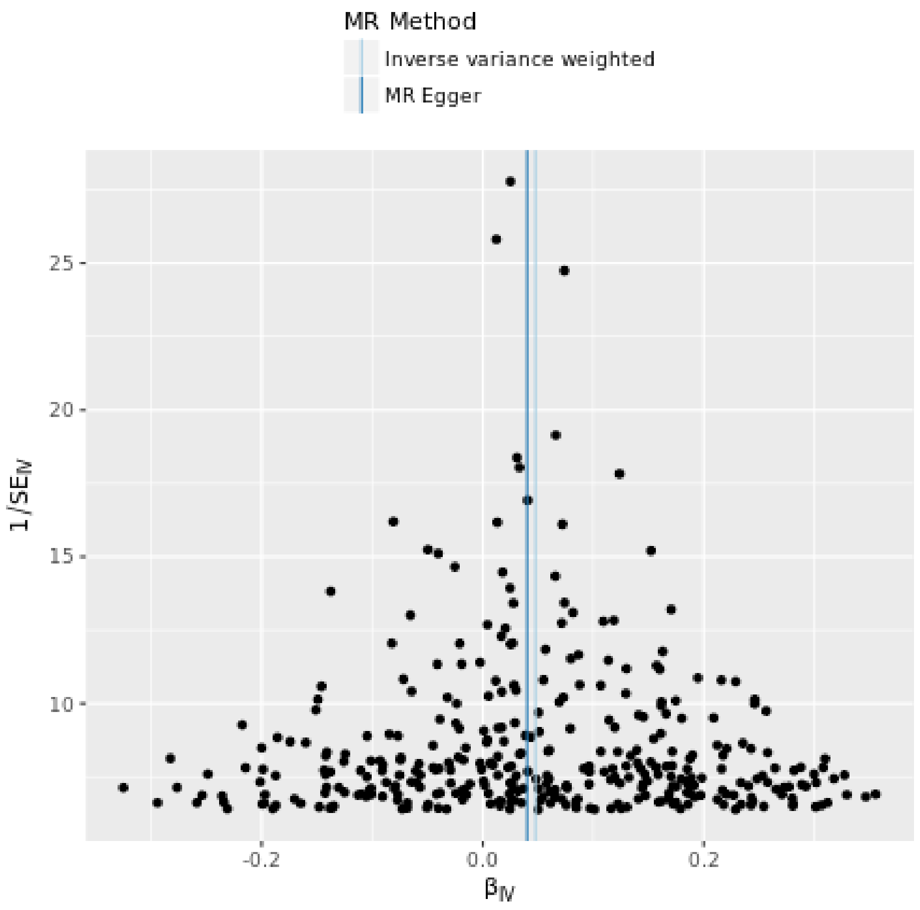

Sitting height

<table>
<tr><td>Before removing instruments</td><td>After removing instruments</td></tr>
<tr><td>Leave-one-out plot
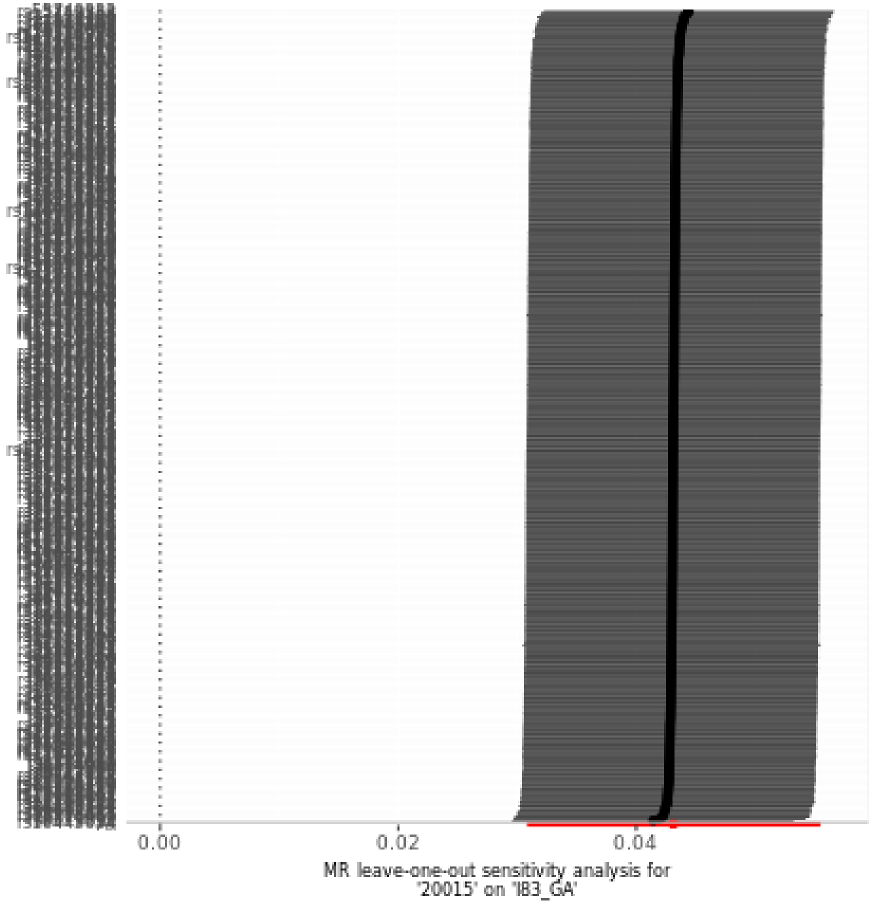
</td><td>Leave-one-out plot
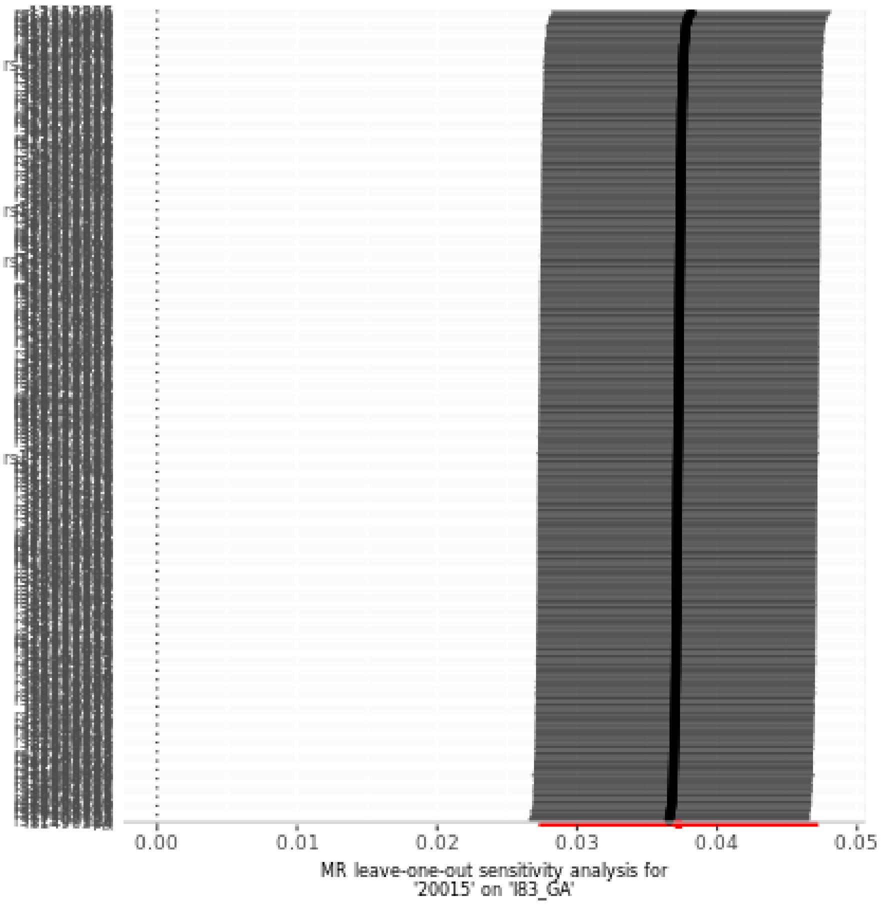
</td></tr>
</table>

Funnel plot

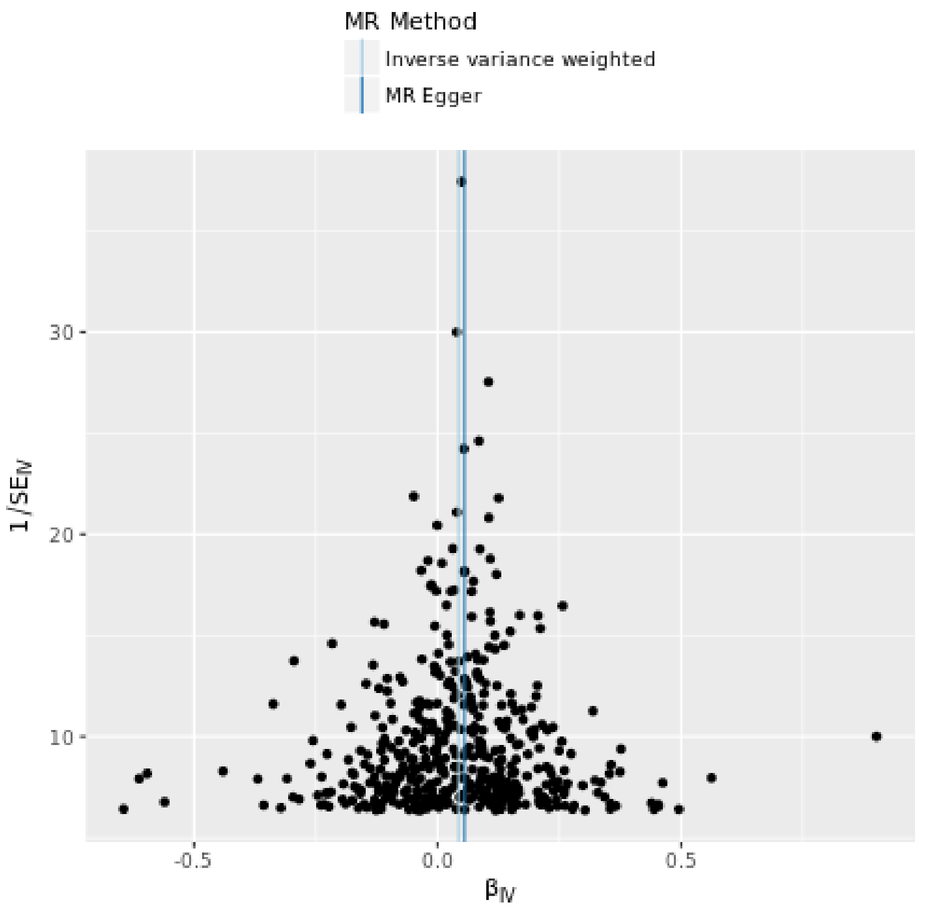

Funnel plot

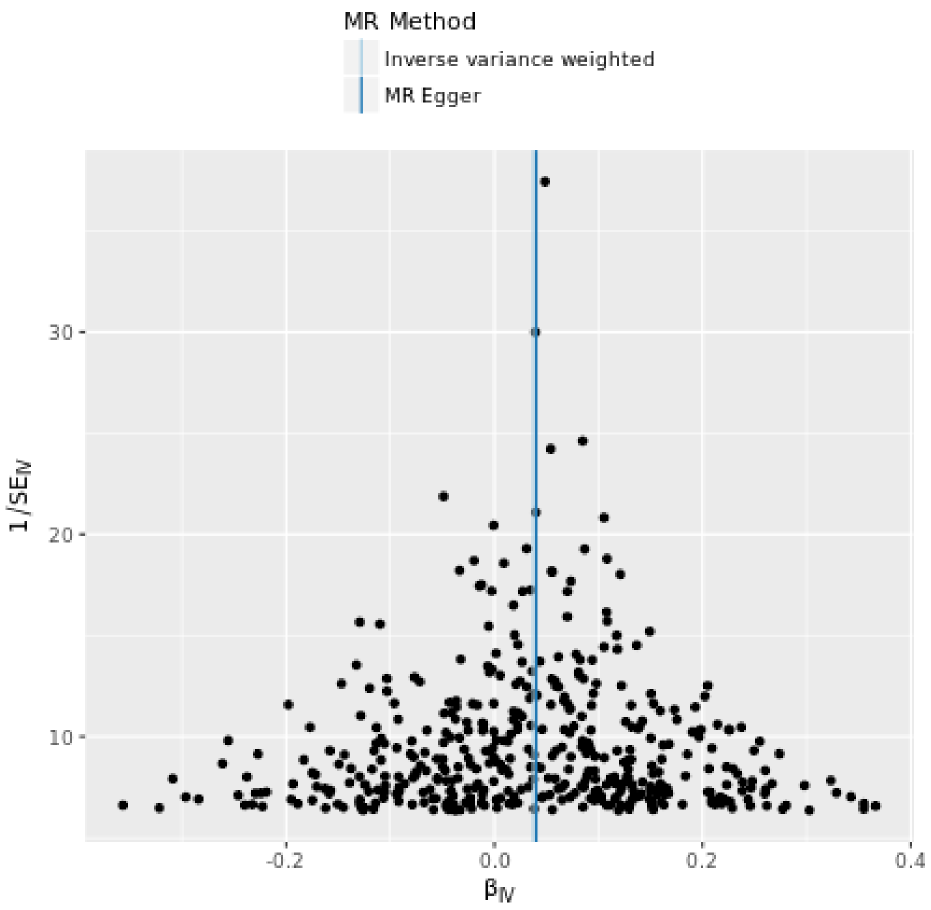

Trunk predicted mass

Before removing instruments	After removing instruments
Leave-one-out plot<br>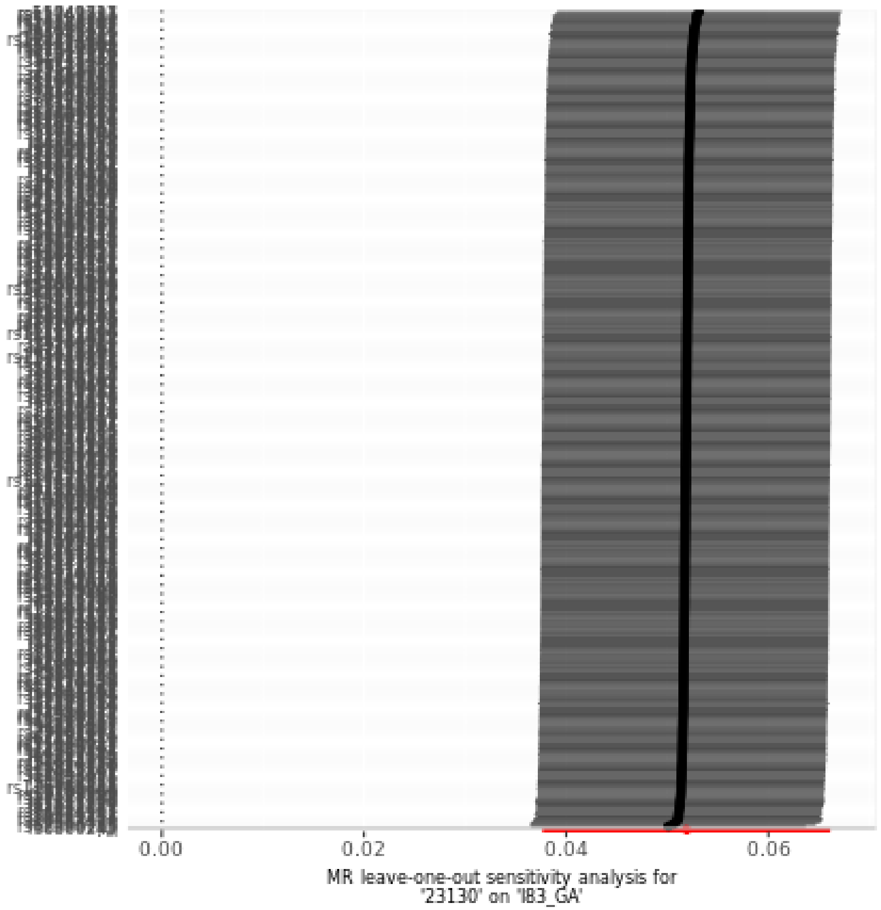 	Leave-one-out plot<br>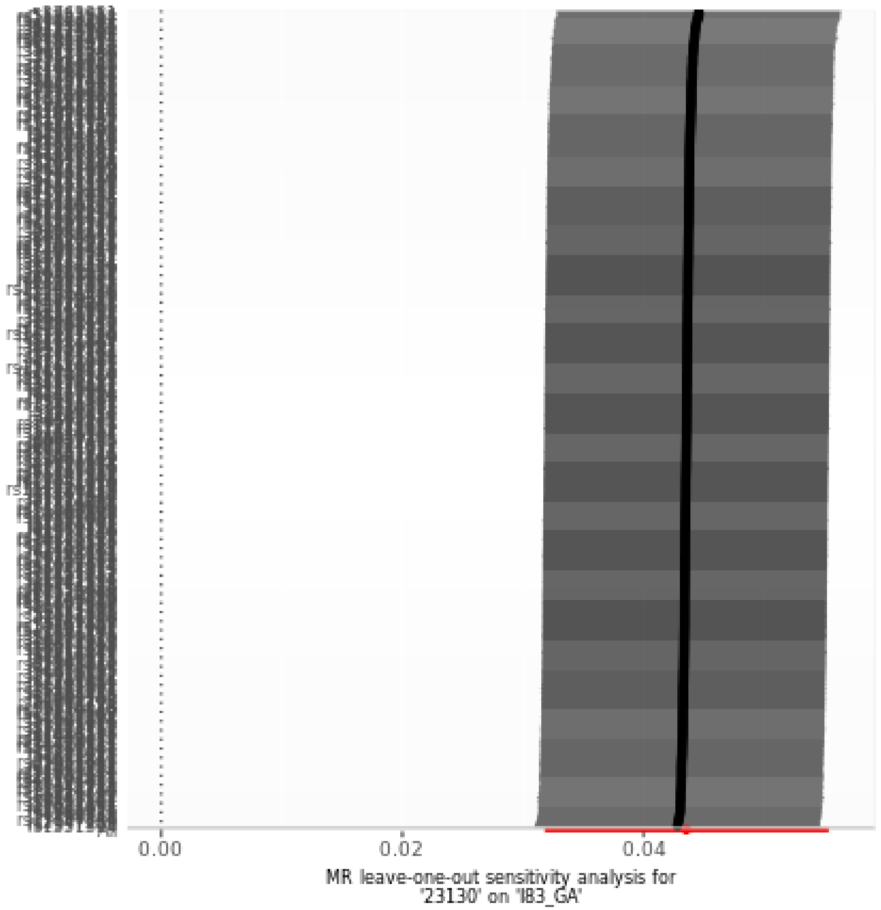

Funnel plot

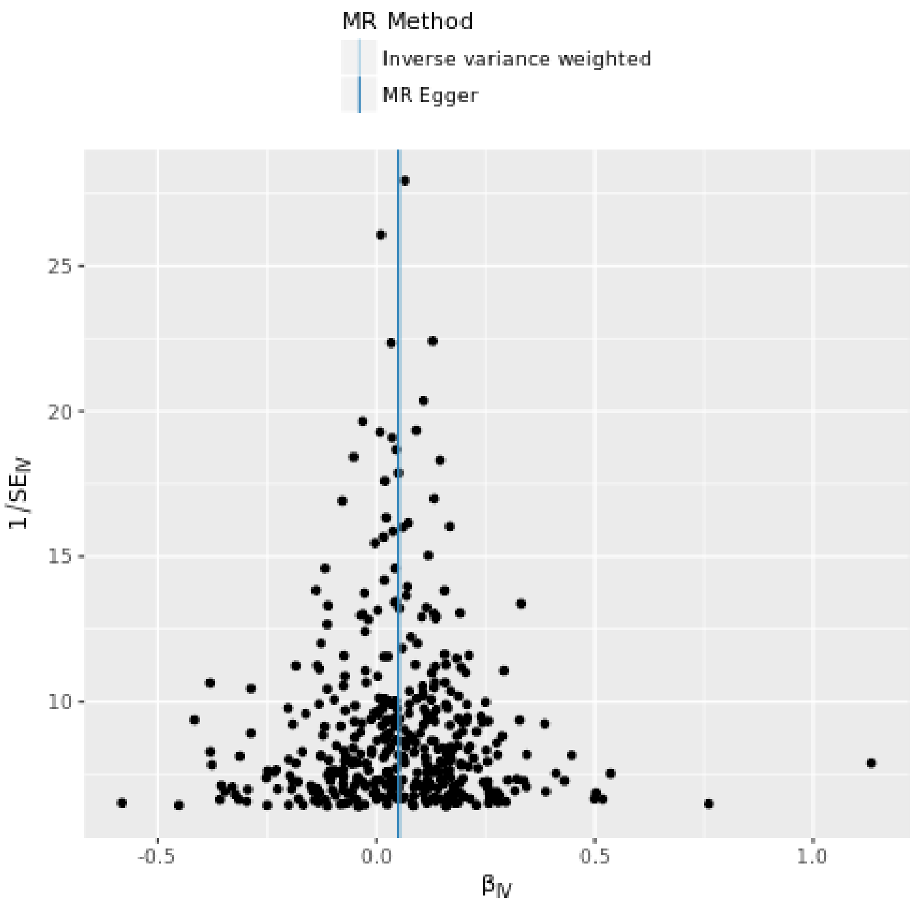

Funnel plot

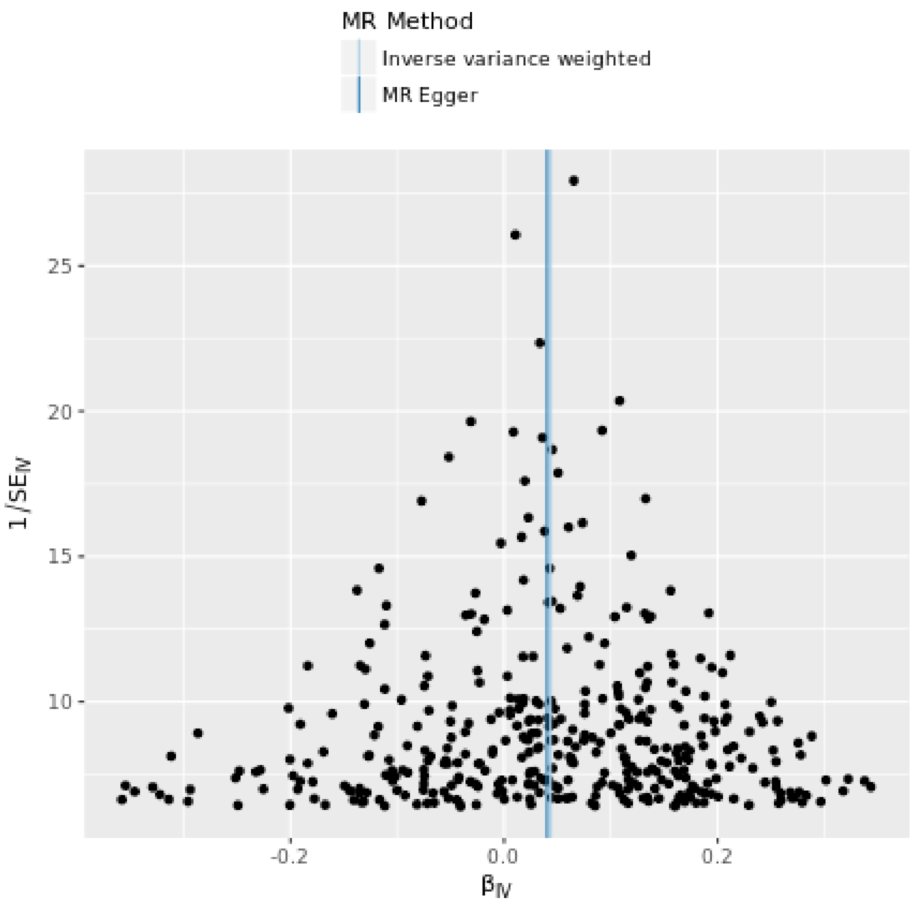

Arm fat-free mass (right)

Before removing instruments	After removing instruments
Leave-one-out plot 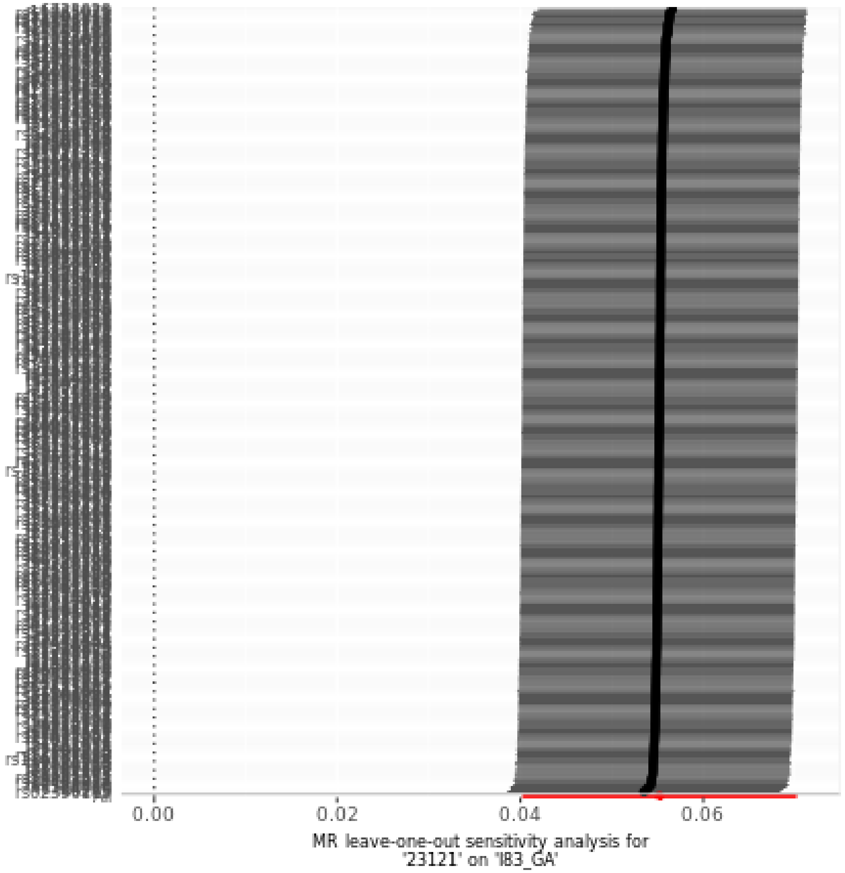	Leave-one-out plot 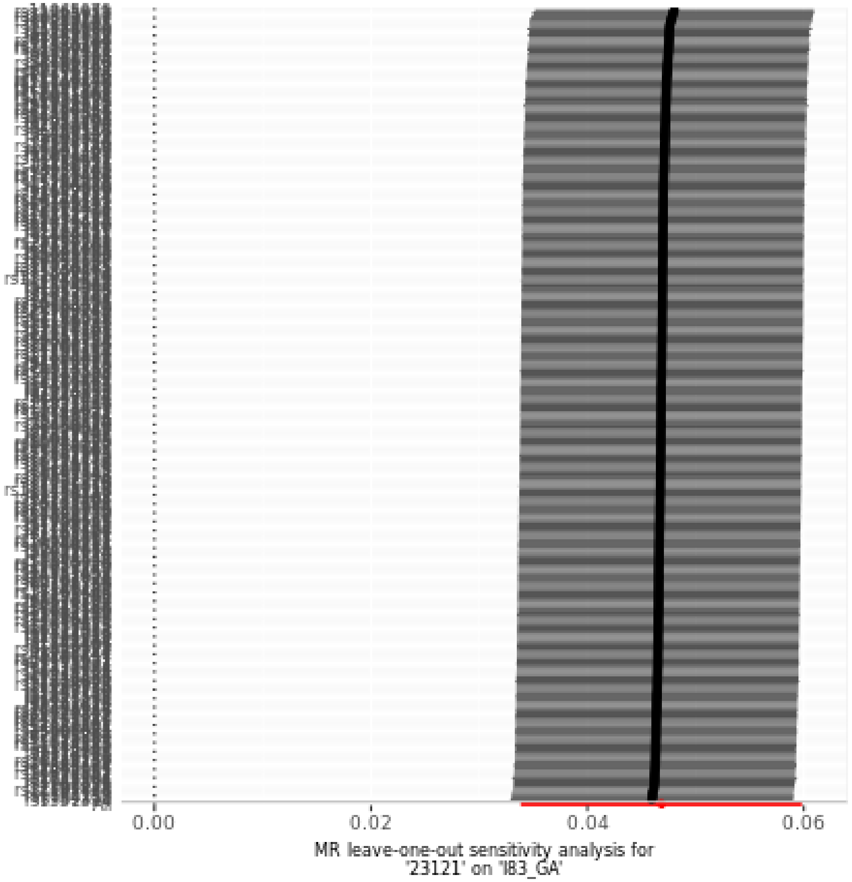

Funnel plot	Funnel plot
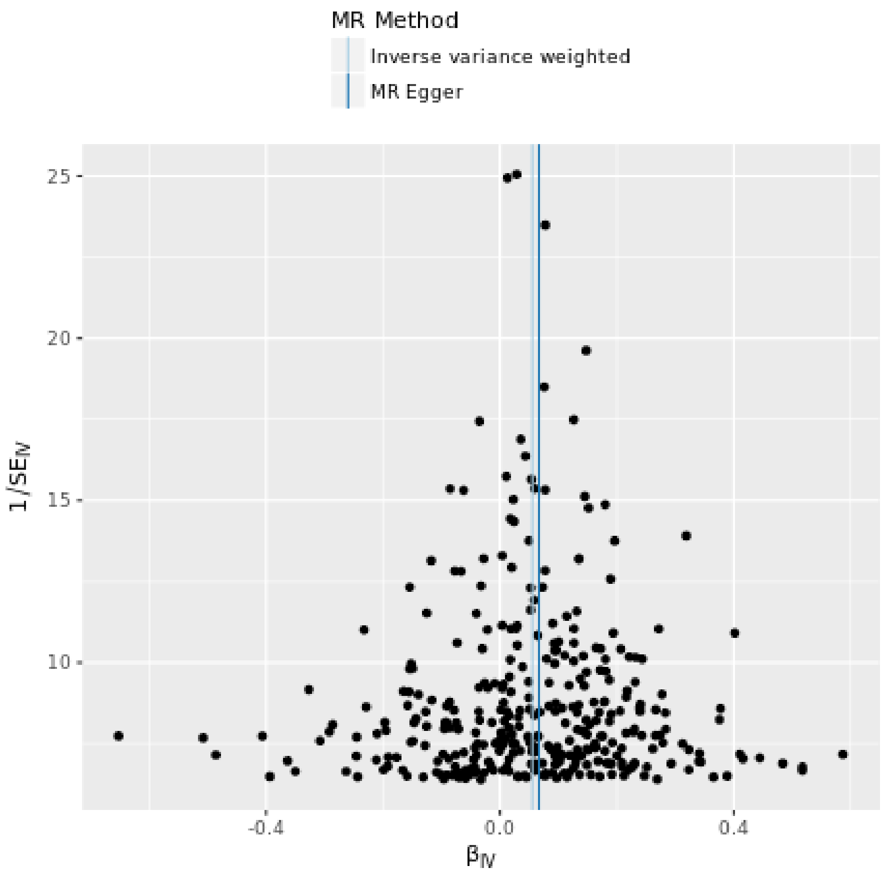	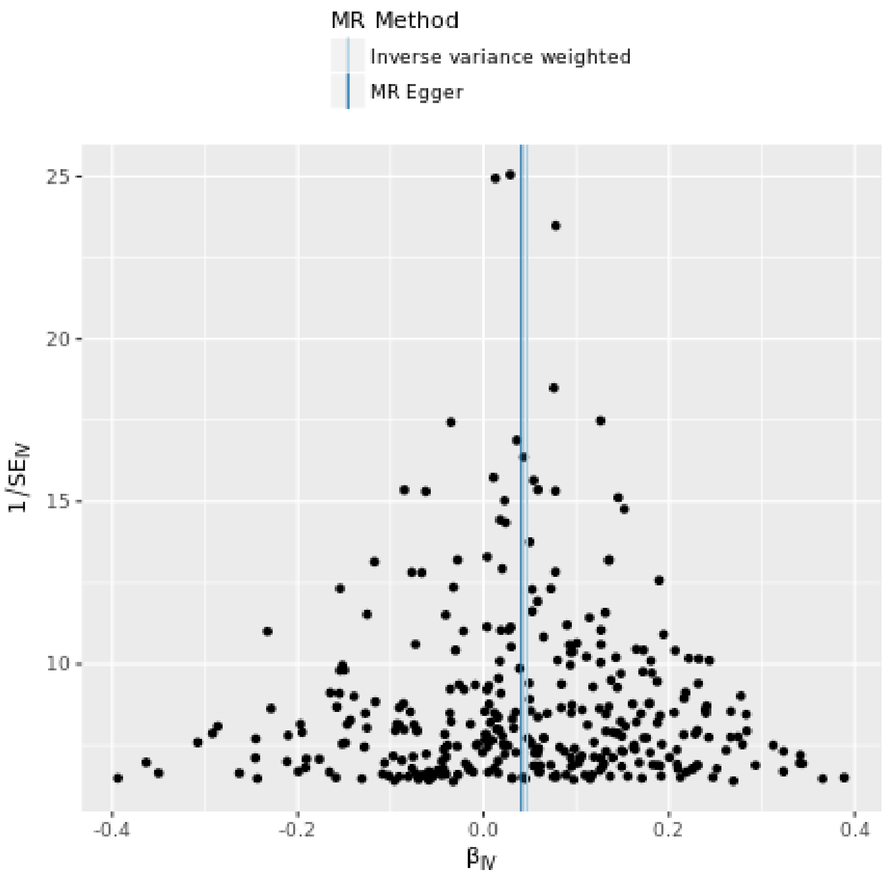

Arm predicted mass (left)

Before removing instruments	After removing instruments
Leave-one-out plot<br>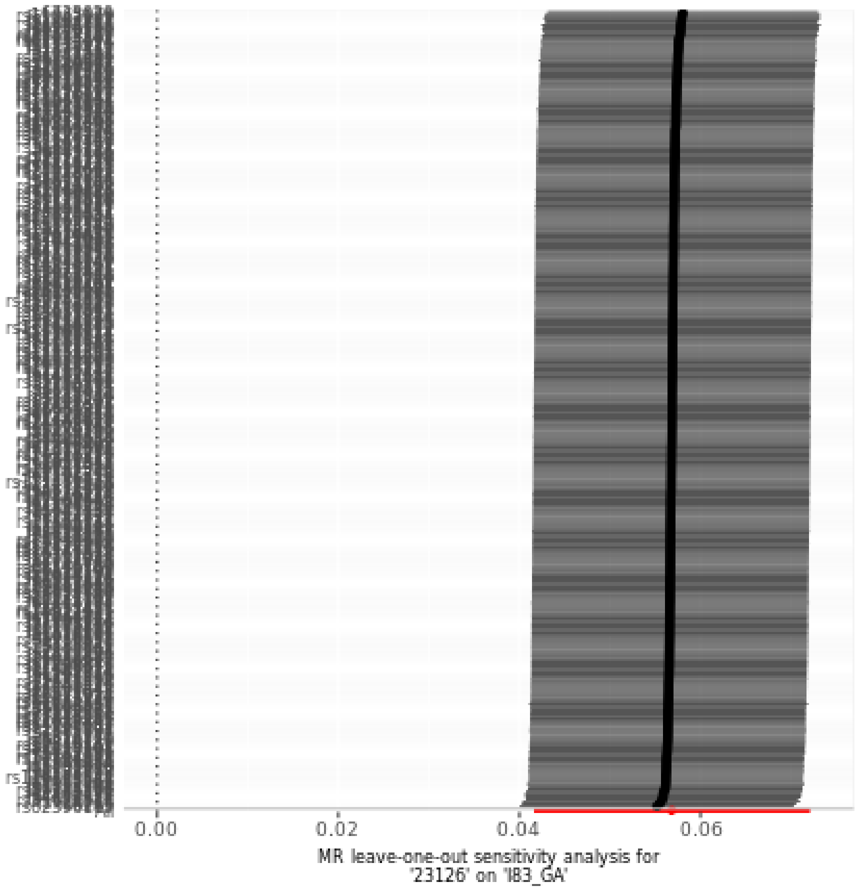	Leave-one-out plot<br>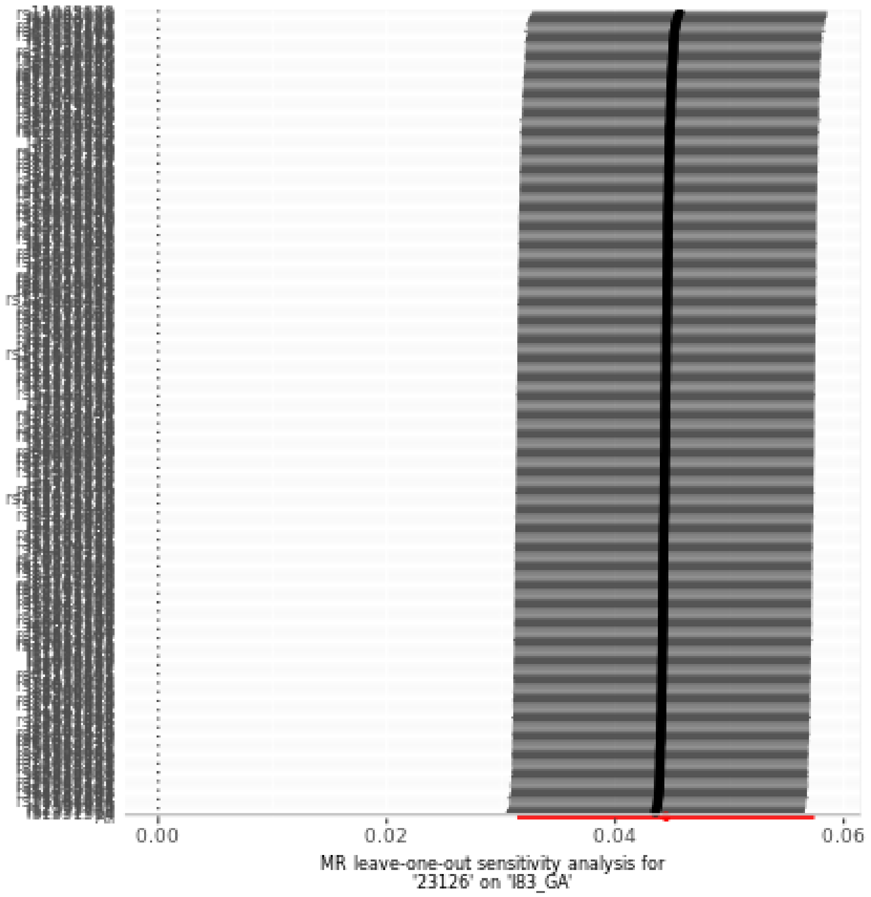

Funnel plot

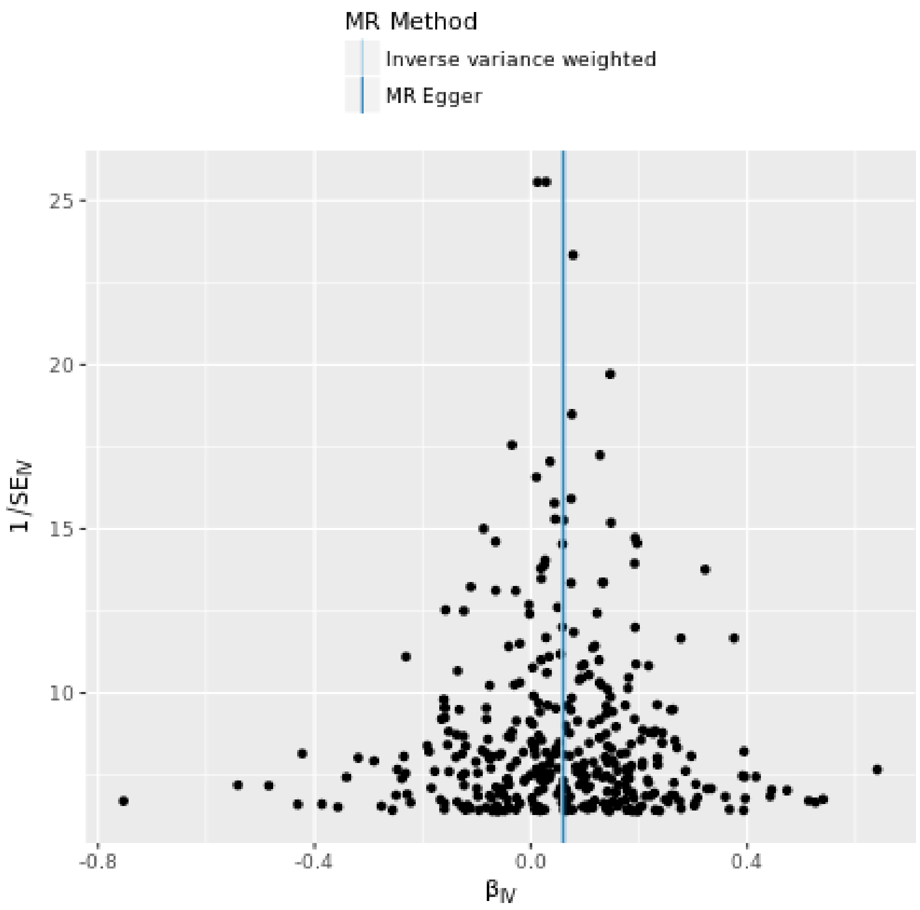

Funnel plot

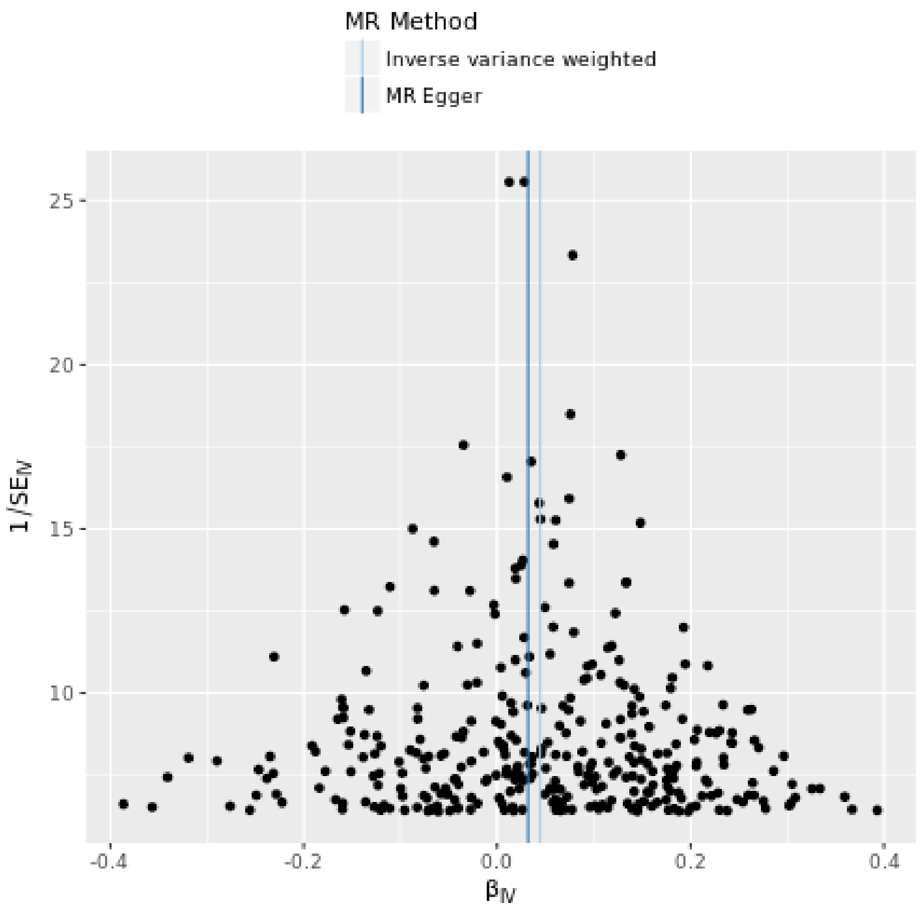

Whole body fat mass

<table>
<tr><td>Before removing instruments</td><td>After removing instruments</td></tr>
<tr><td>Leave-one-out plot
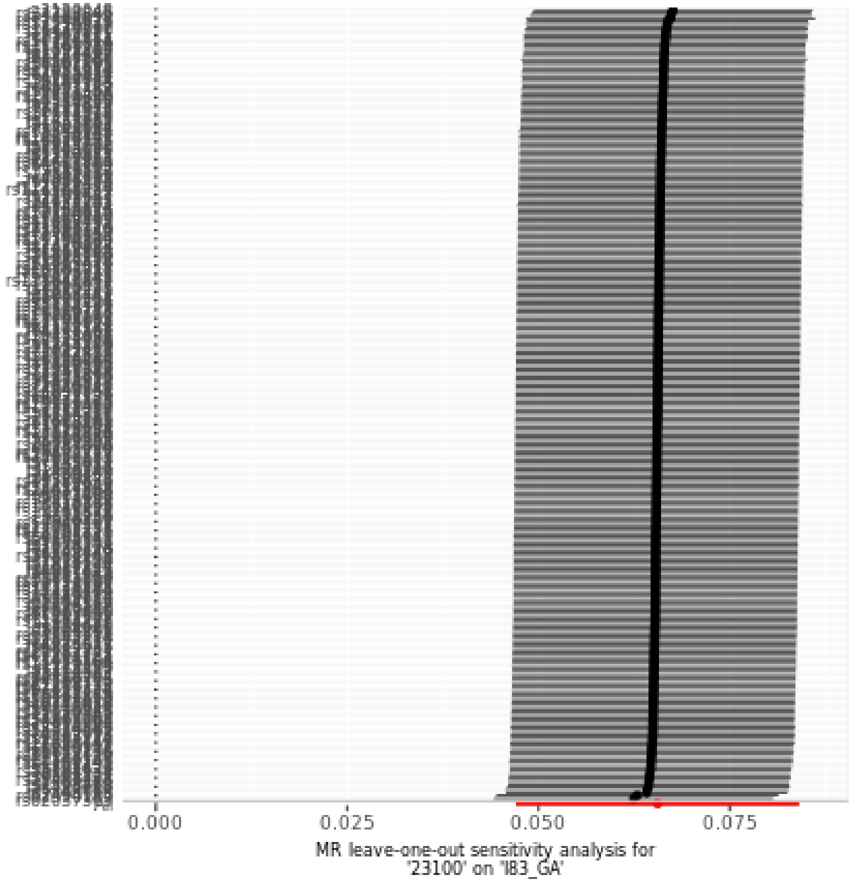
</td><td>Leave-one-out plot
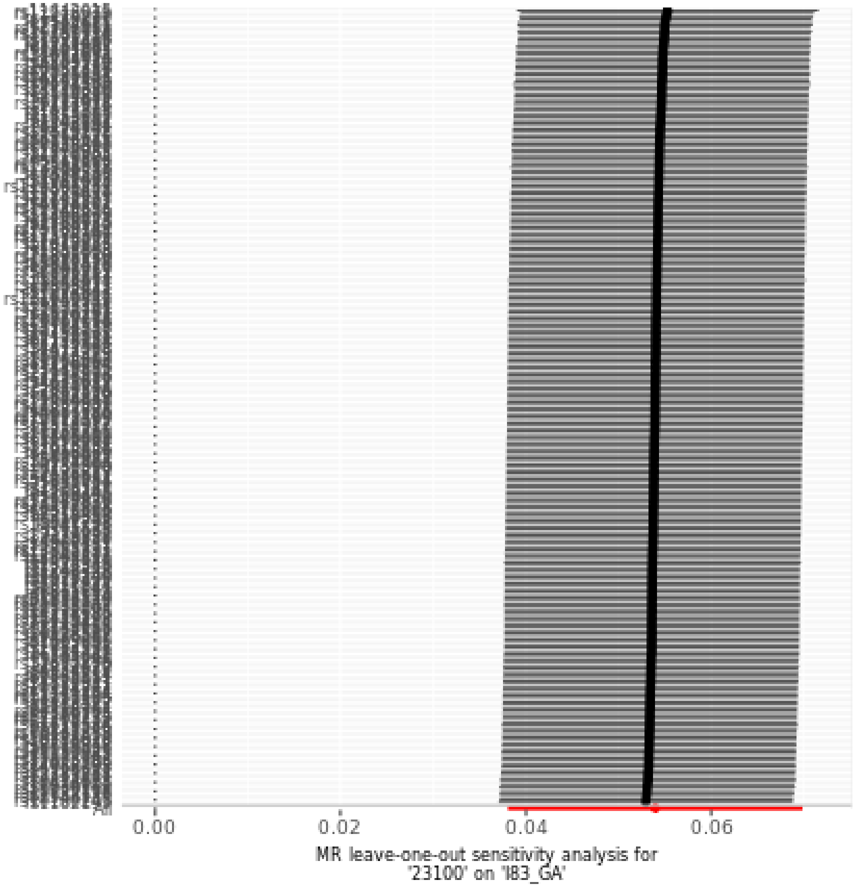
</td></tr>
</table>

Funnel plot	Funnel plot
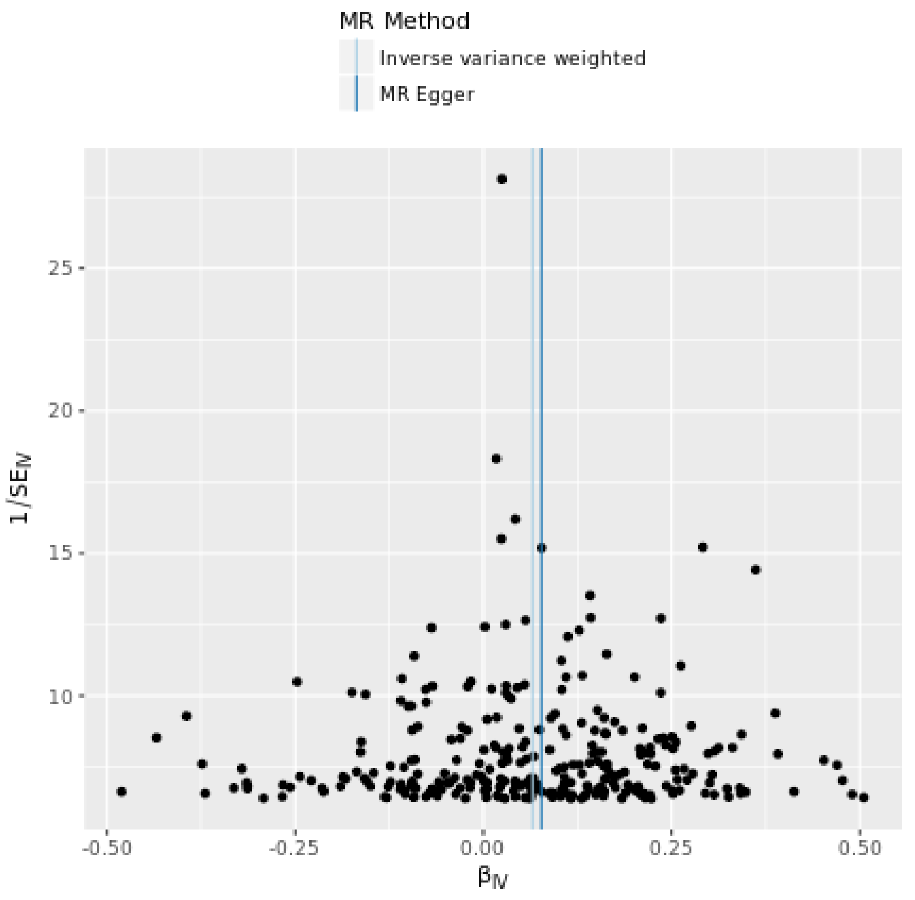	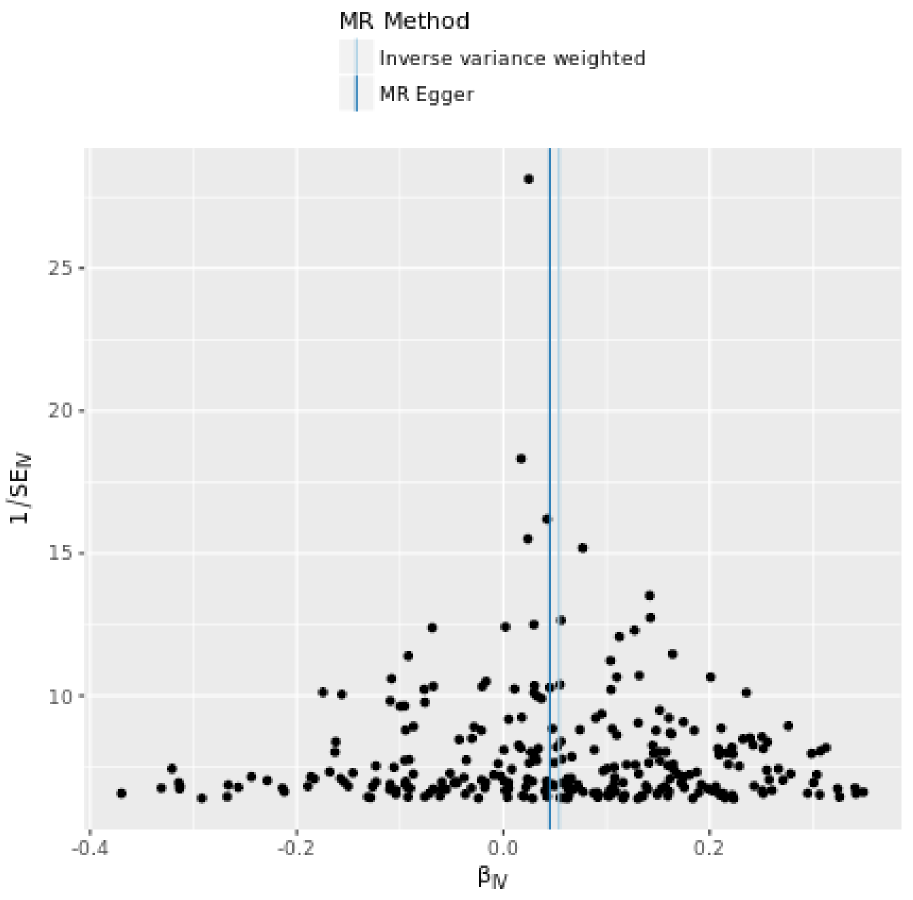

Arm fat-free mass (left)

Before removing instruments	After removing instruments
Leave-one-out plot 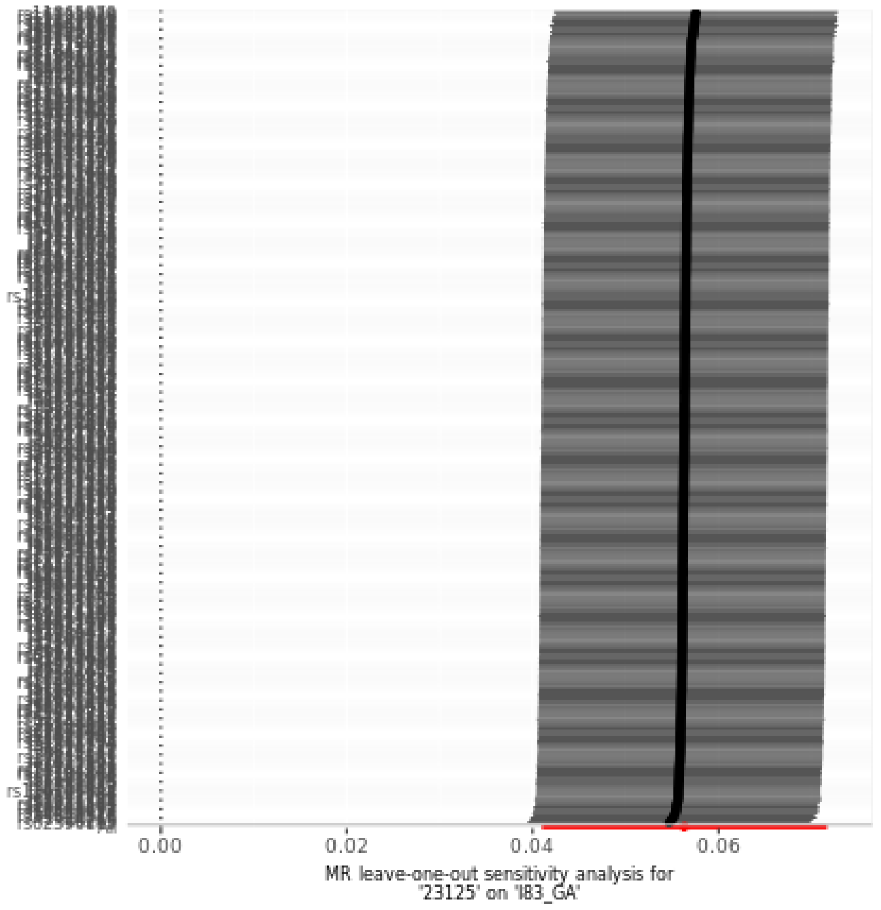	Leave-one-out plot 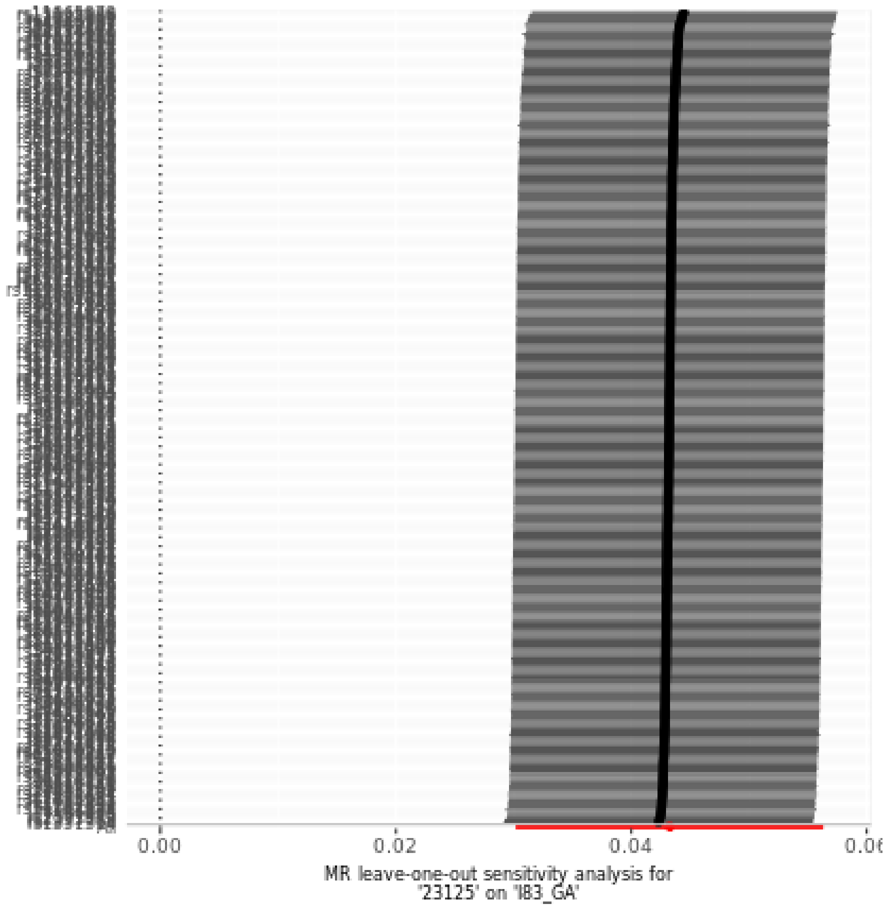

Funnel plot

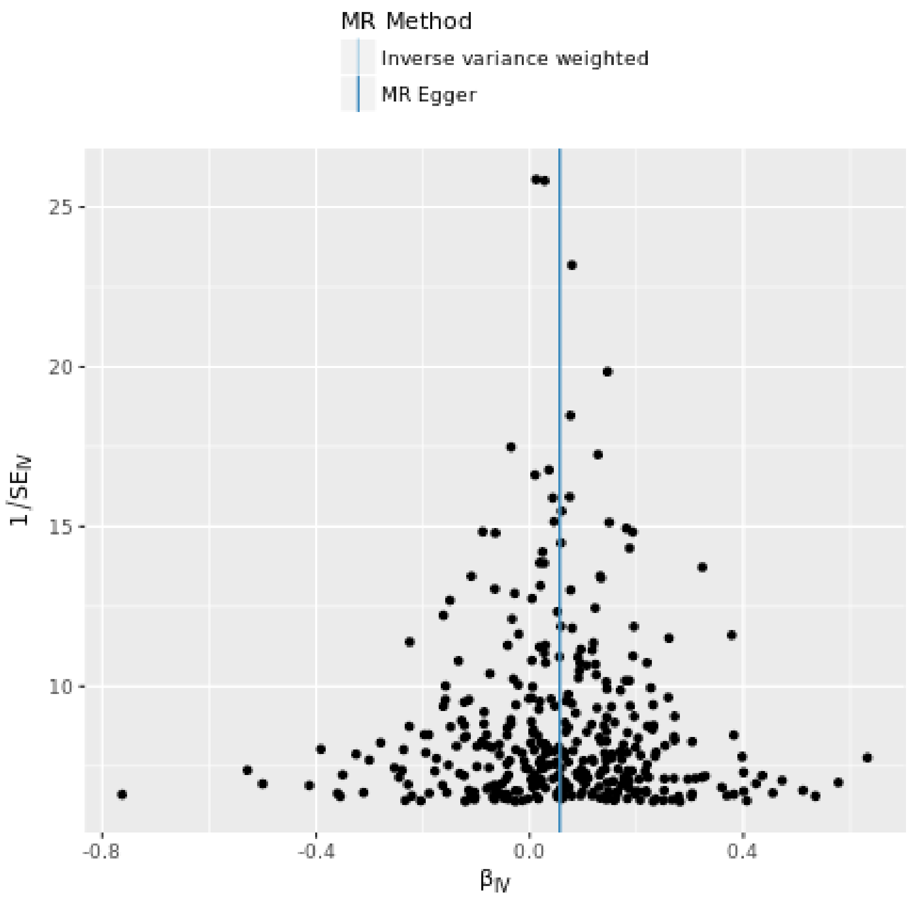

Funnel plot

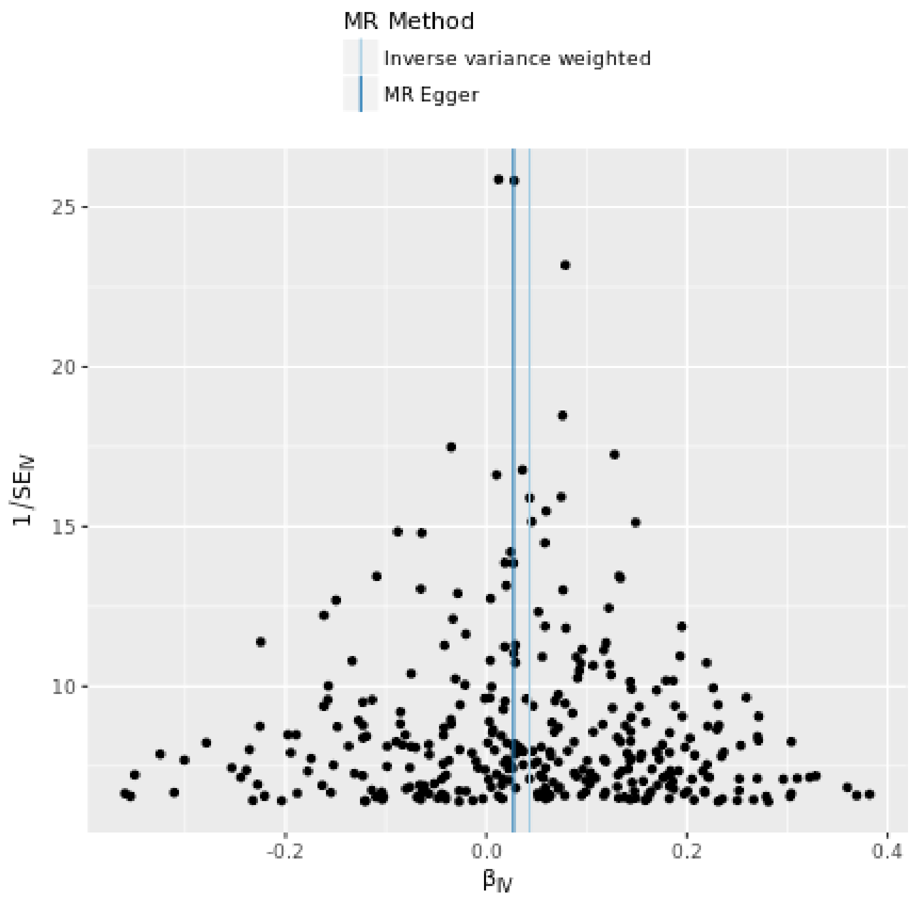

Hip circumference

Before removing instruments	After removing instruments
Leave-one-out plot 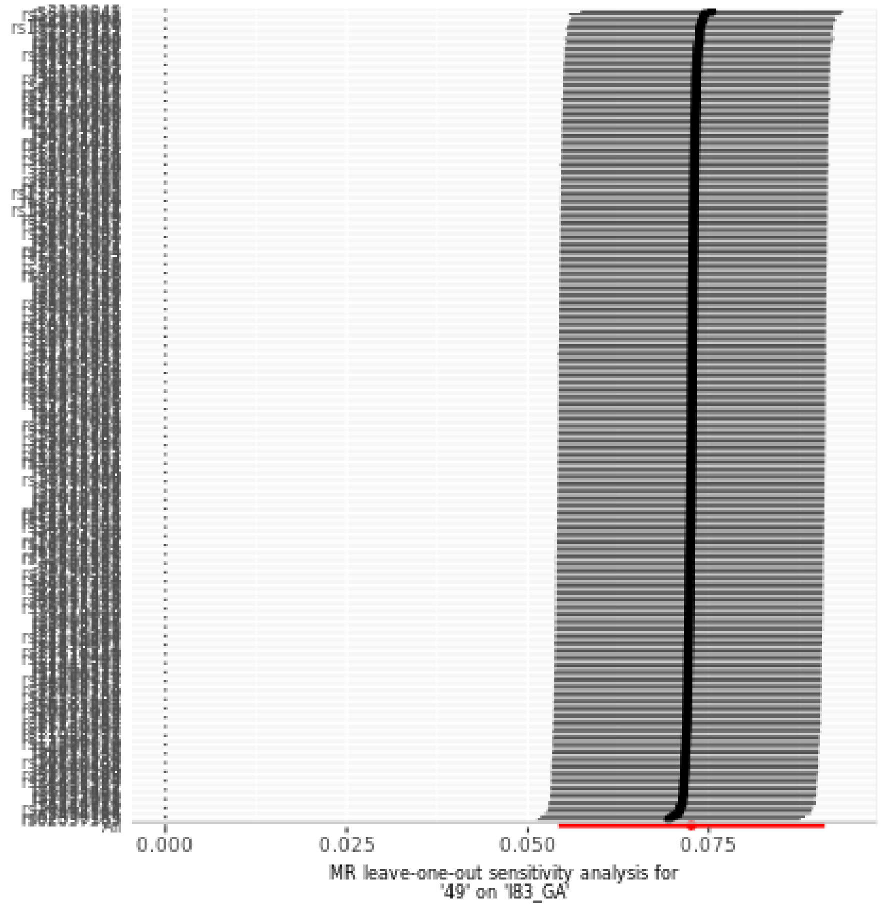	Leave-one-out plot 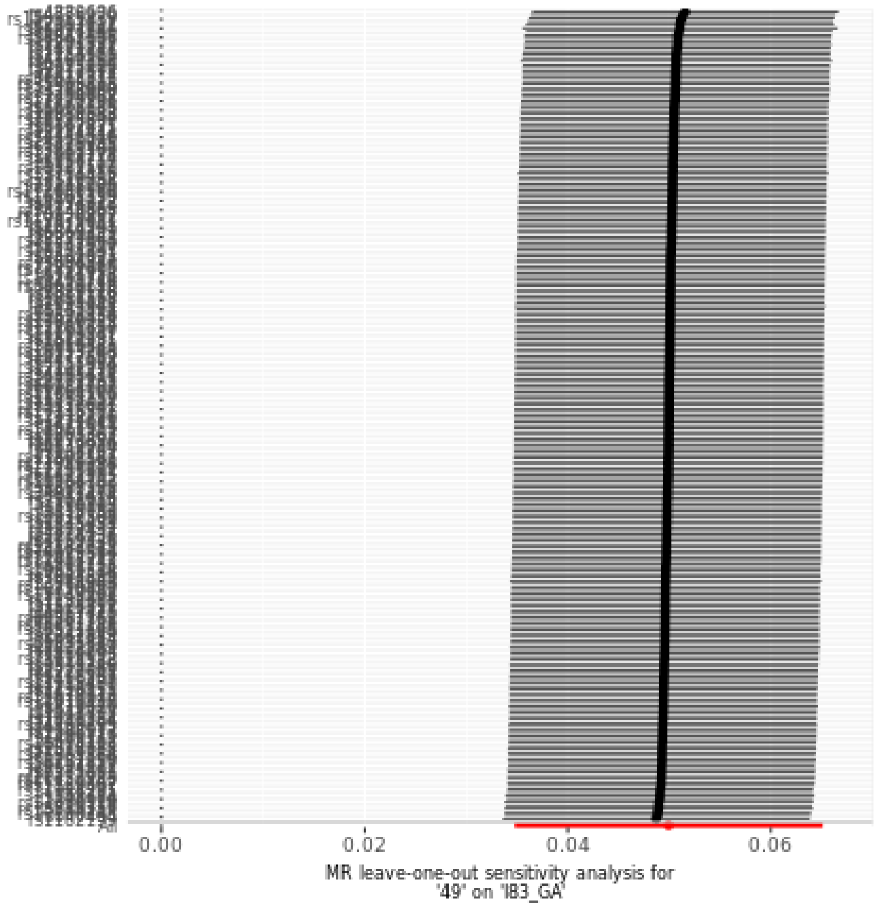

Funnel plot

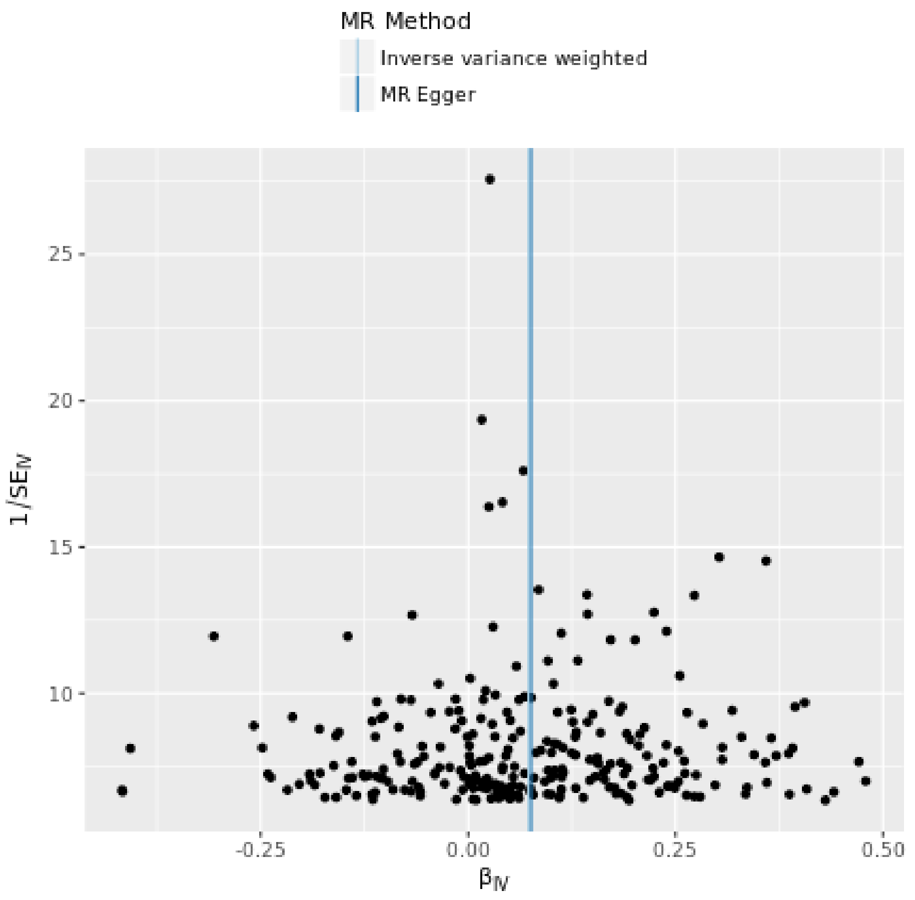

Funnel plot

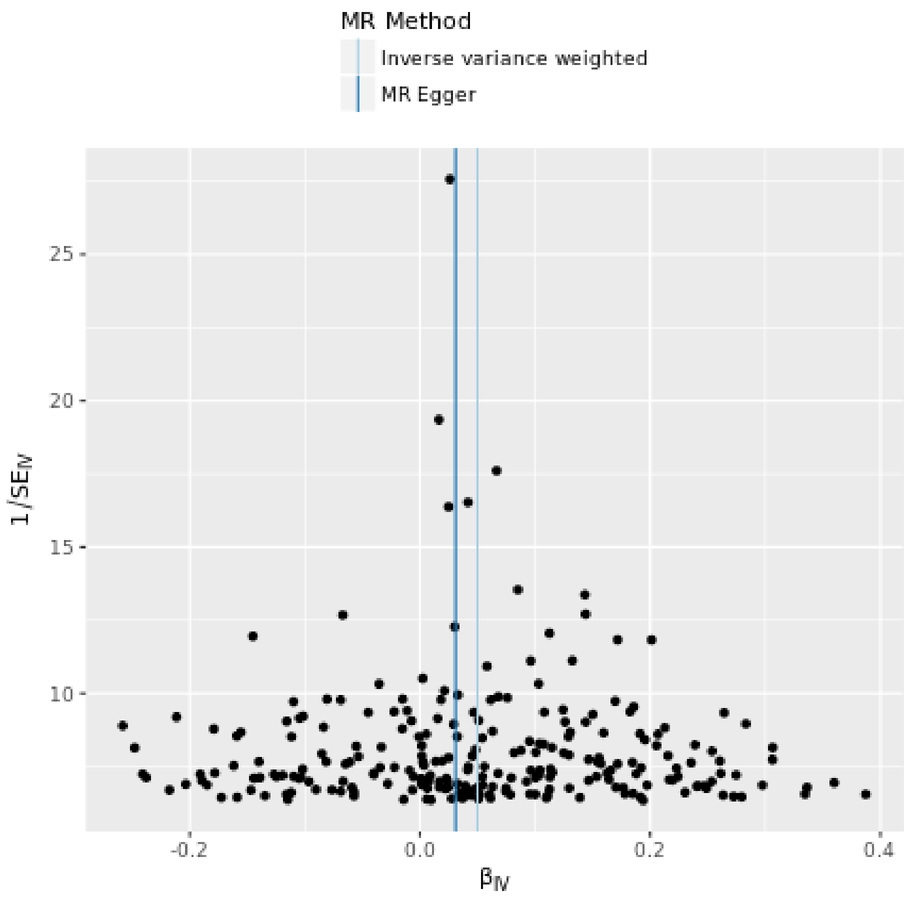

Arm predicted mass (right)

Before removing instruments	After removing instruments
Leave-one-out plot<br>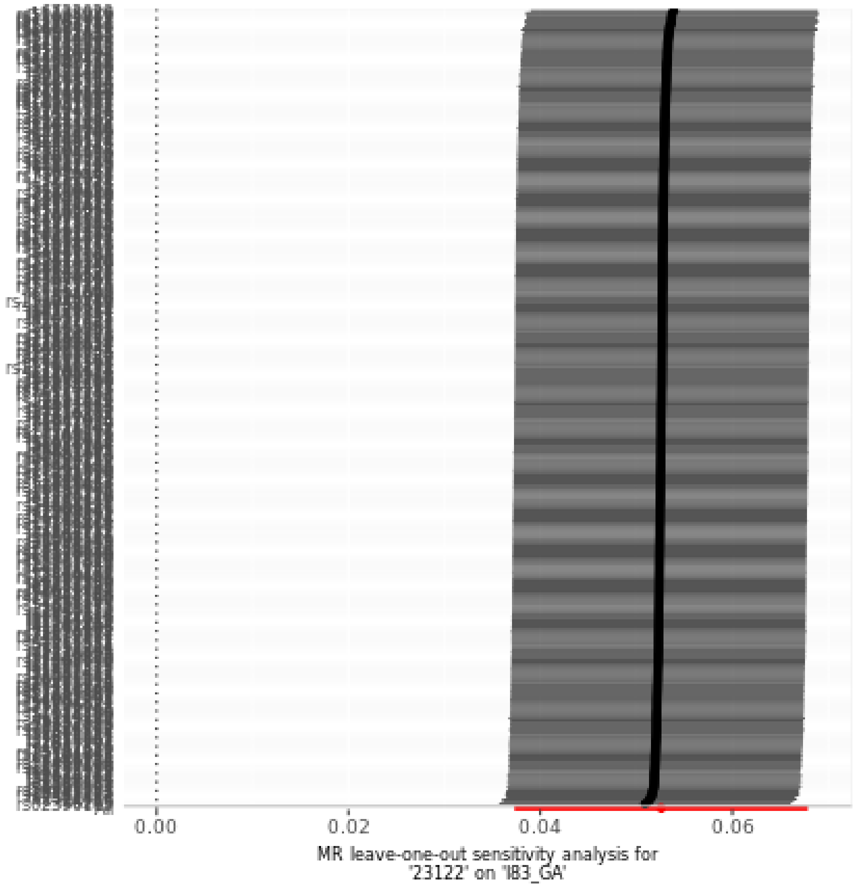 	Leave-one-out plot<br>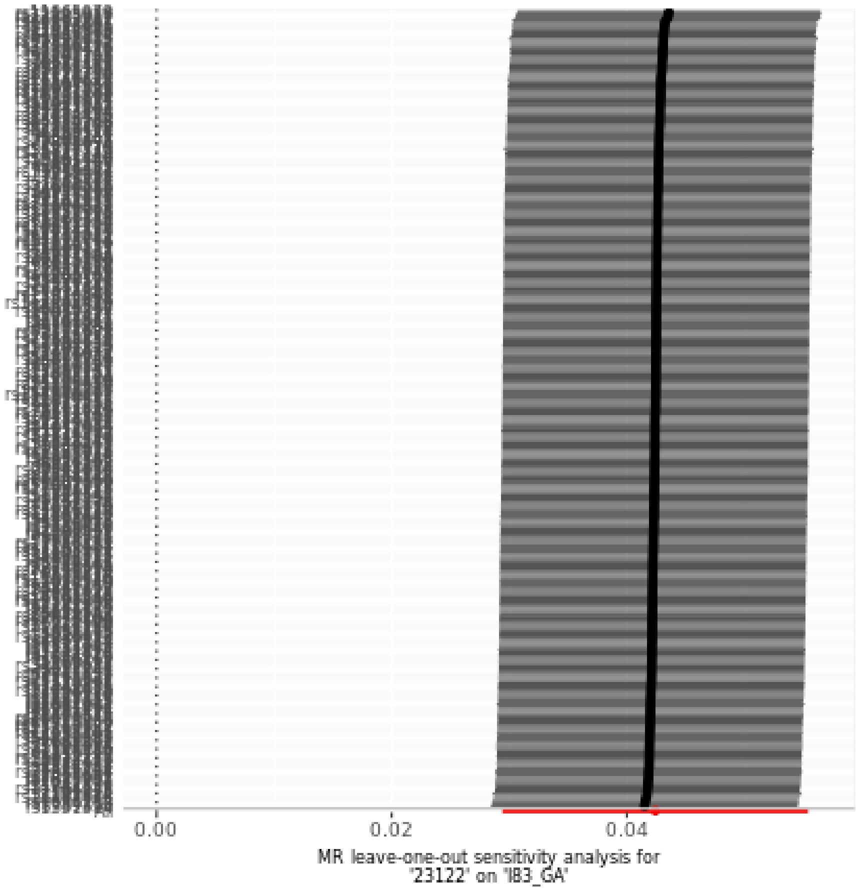

Funnel plot	Funnel plot
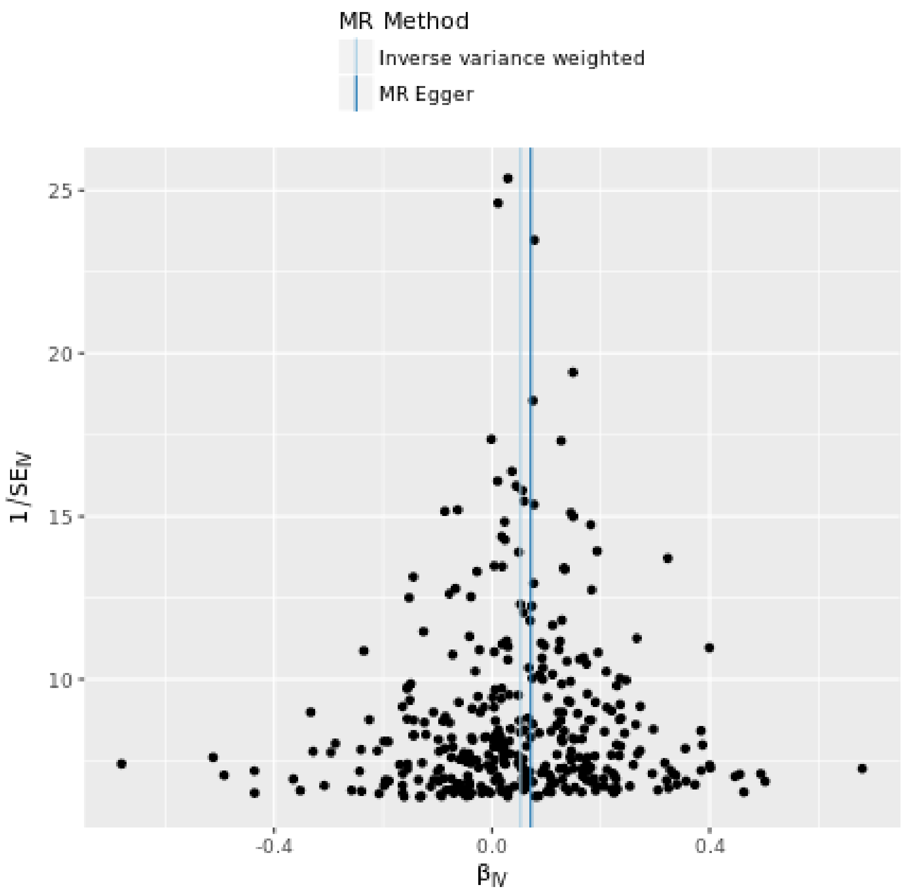	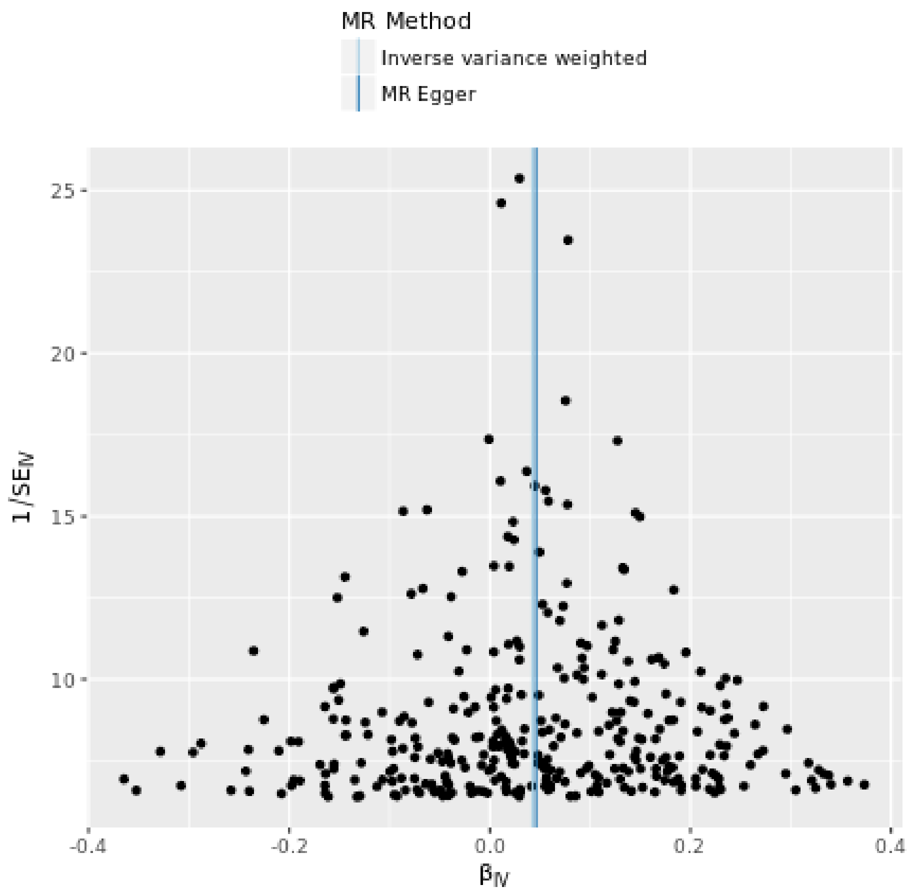

Arm fat mass (left)

Before removing instruments	After removing instruments
Leave-one-out plot<br>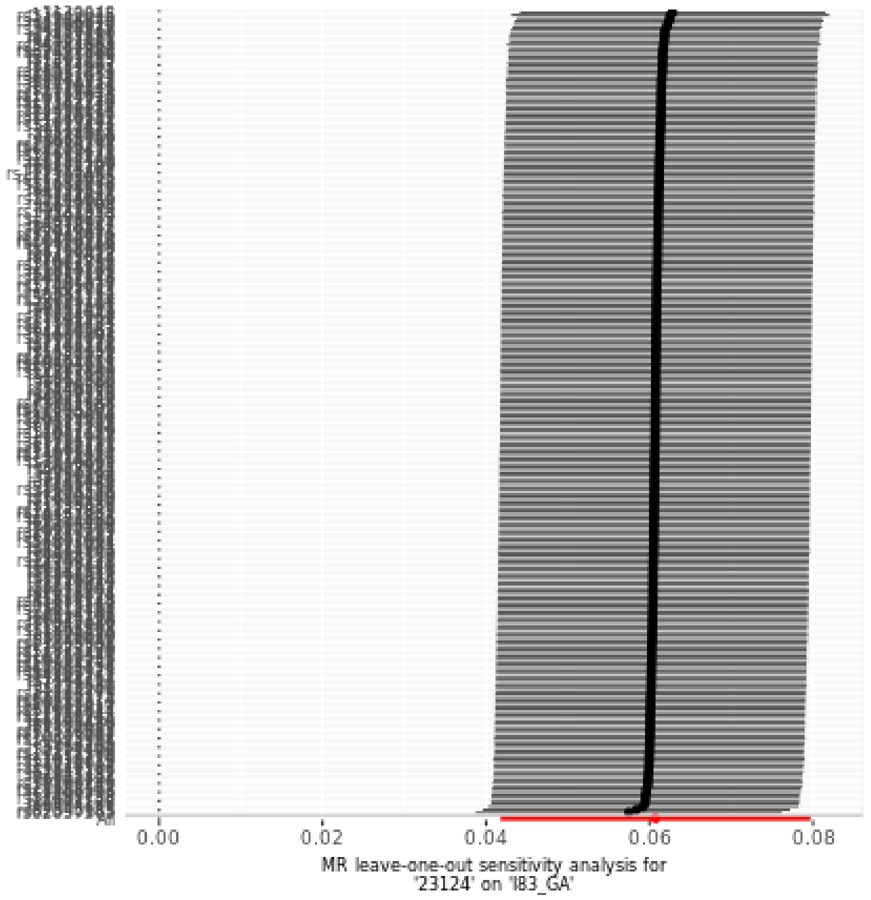 	Leave-one-out plot<br>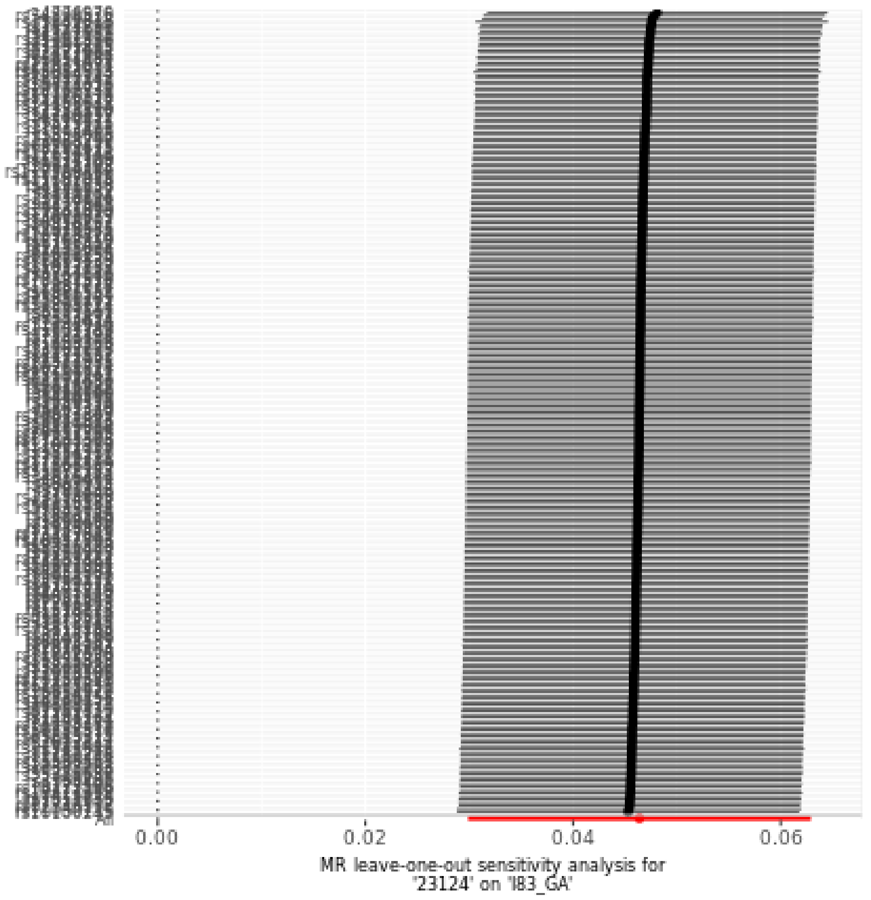

Funnel plot	Funnel plot
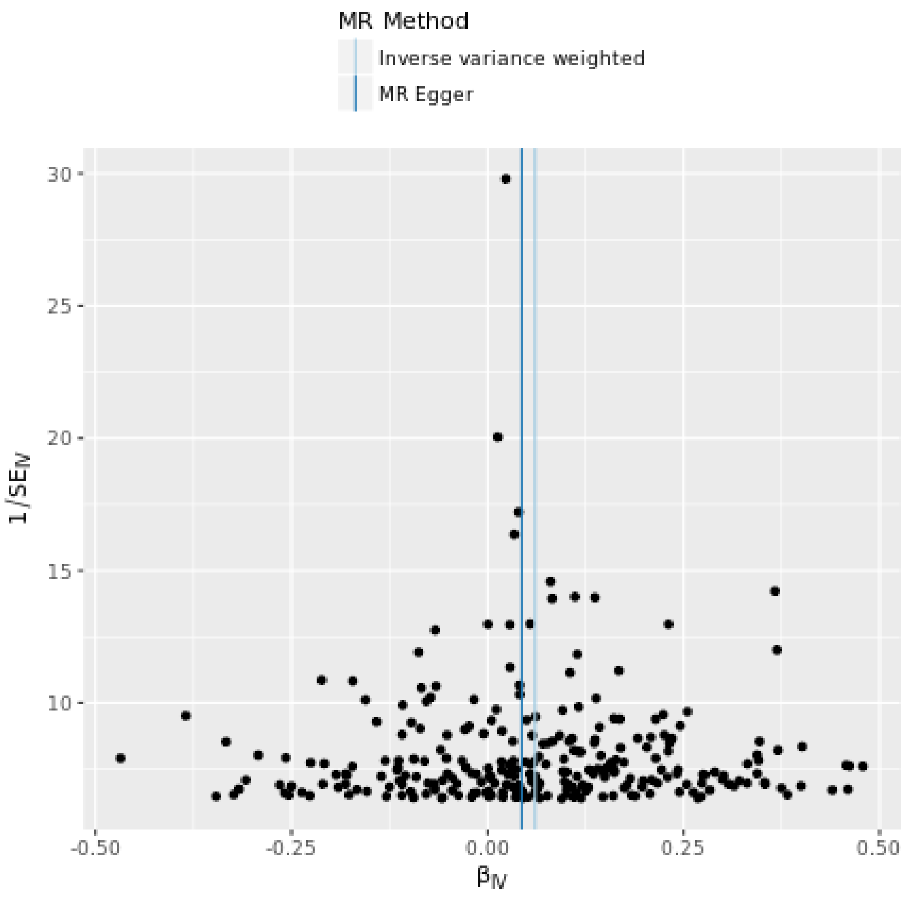	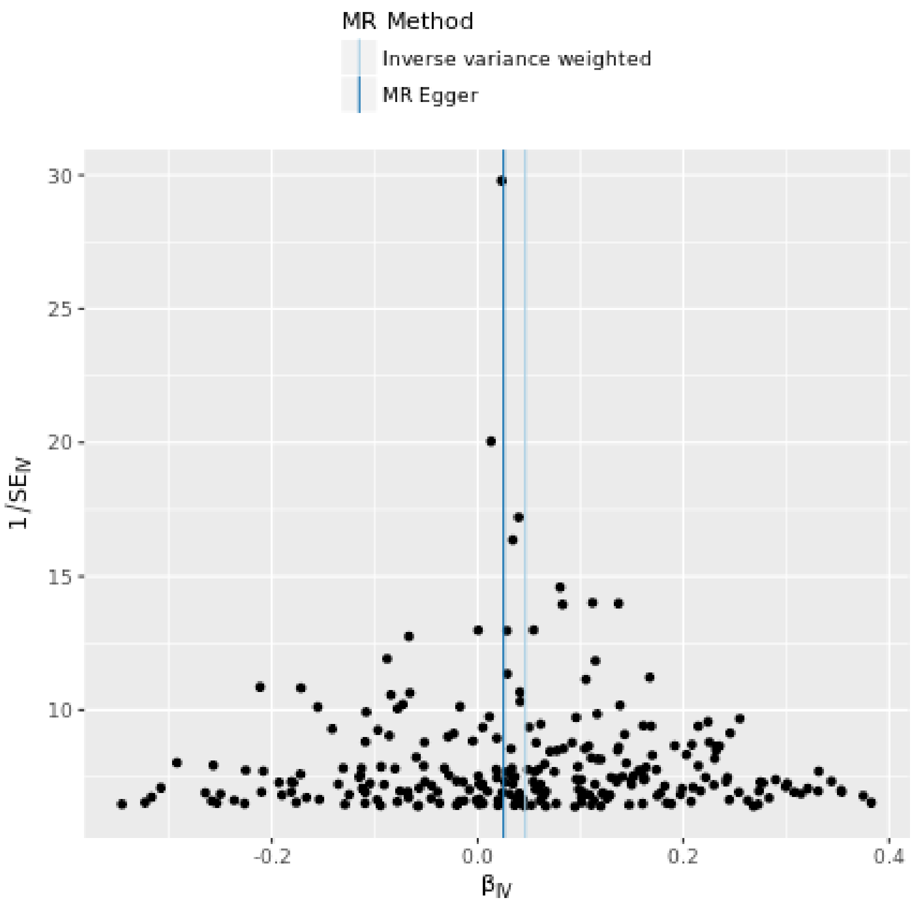

Arm fat mass (right)

<table>
<tr><td>Before removing instruments</td><td>After removing instruments</td></tr>
<tr><td>Leave-one-out plot
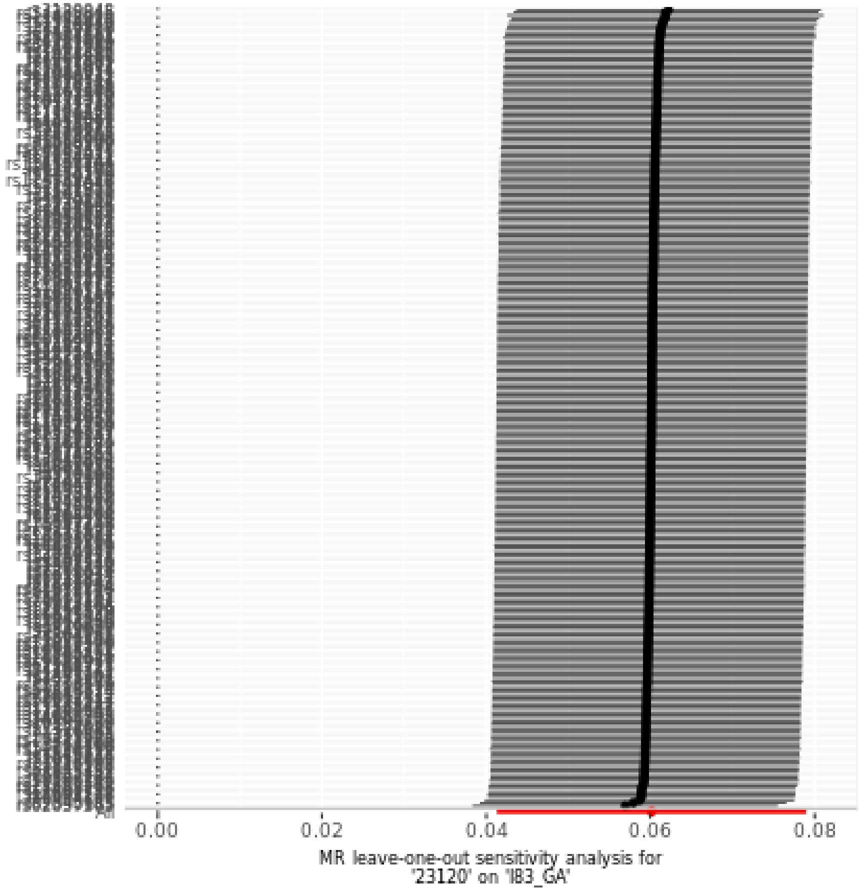
</td><td>Leave-one-out plot
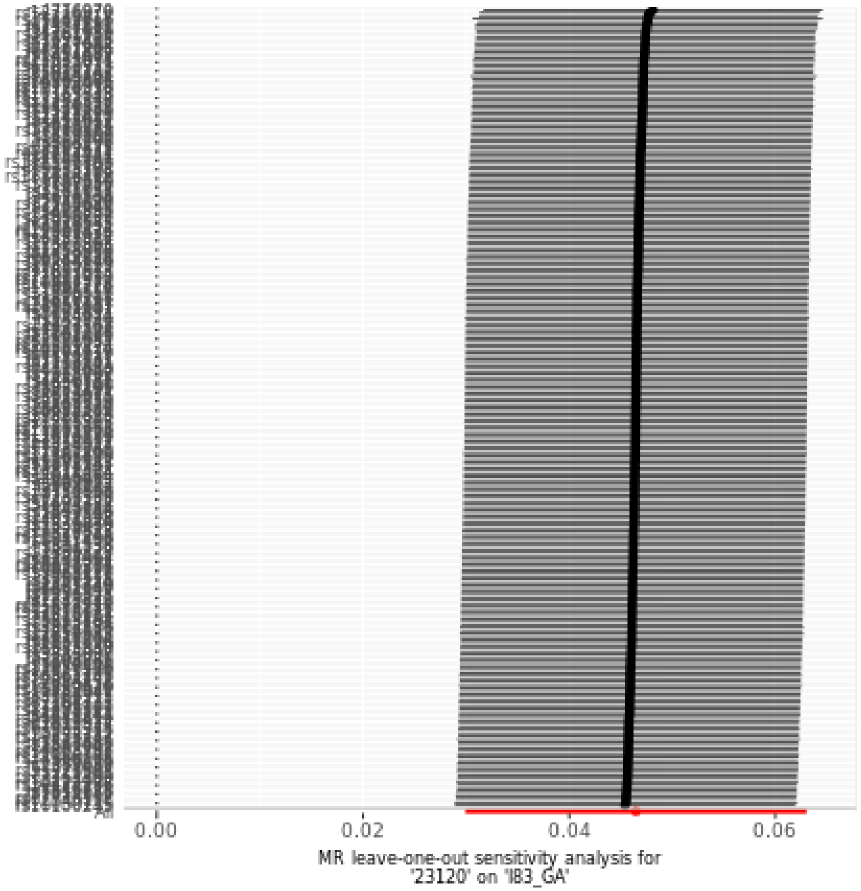
</td></tr>
</table>

Funnel plot

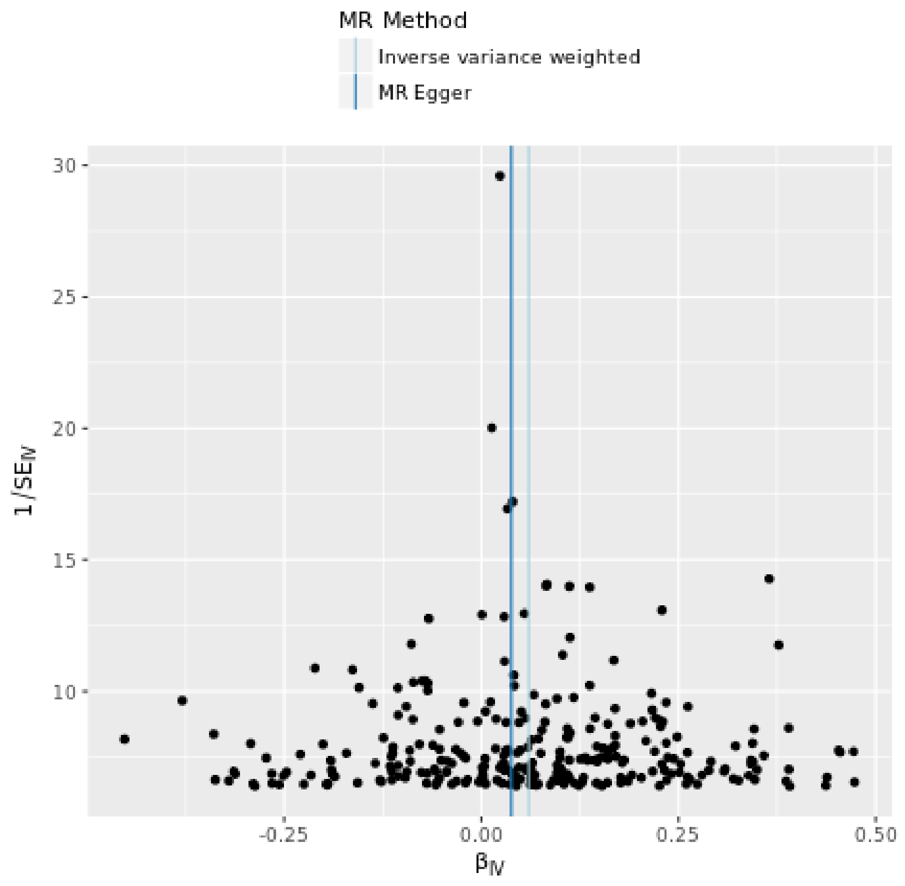

Funnel plot

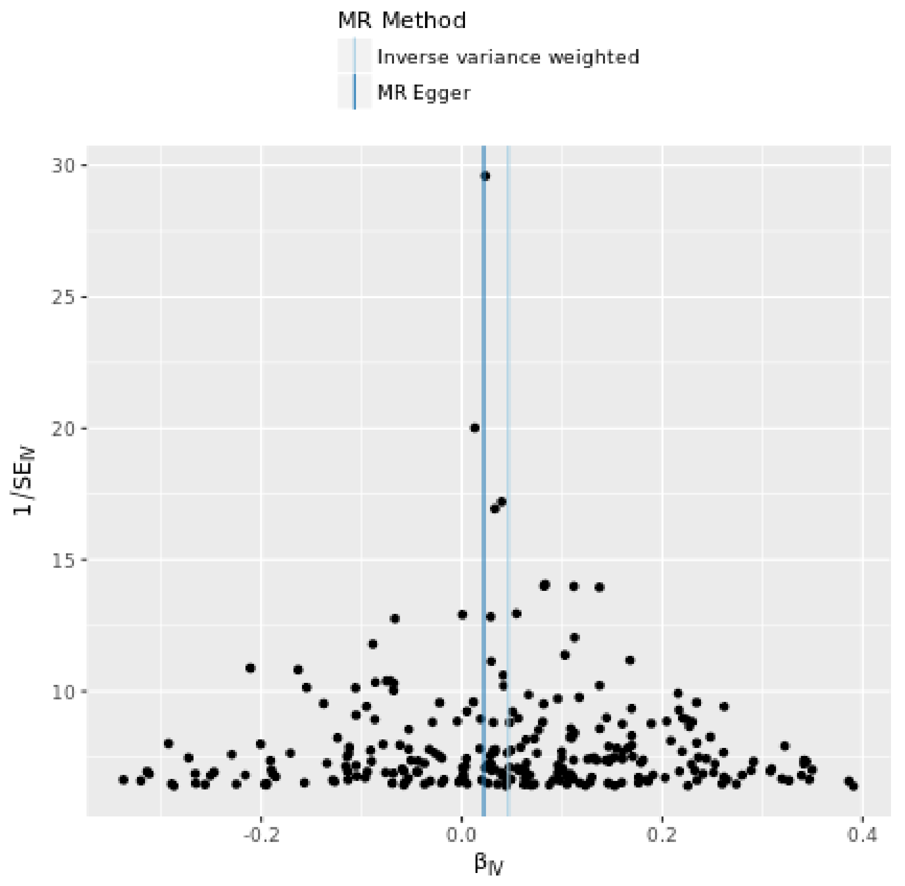

Leg fat mass (left)

Before removing instruments	After removing instruments
Leave-one-out plot 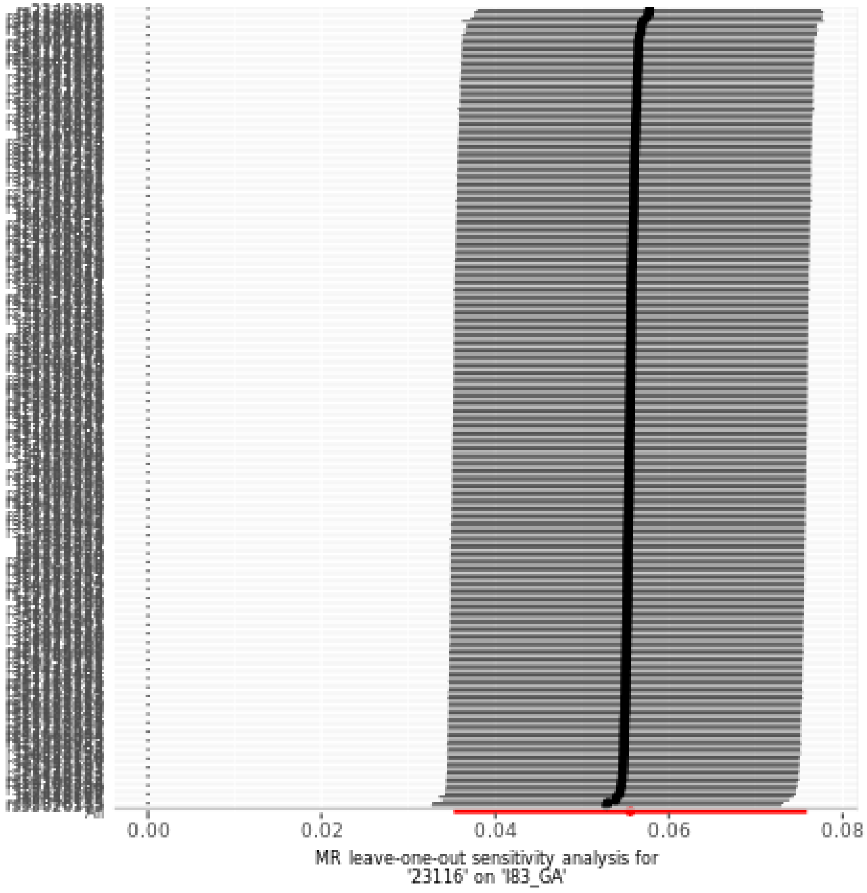	Leave-one-out plot 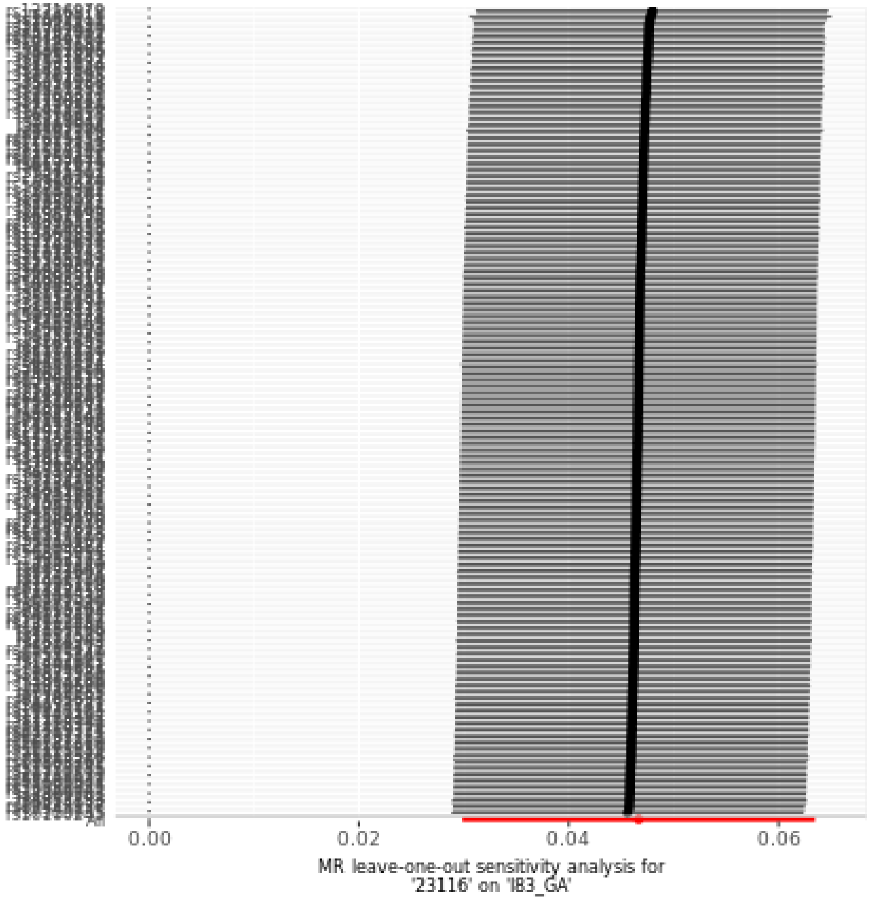

Funnel plot

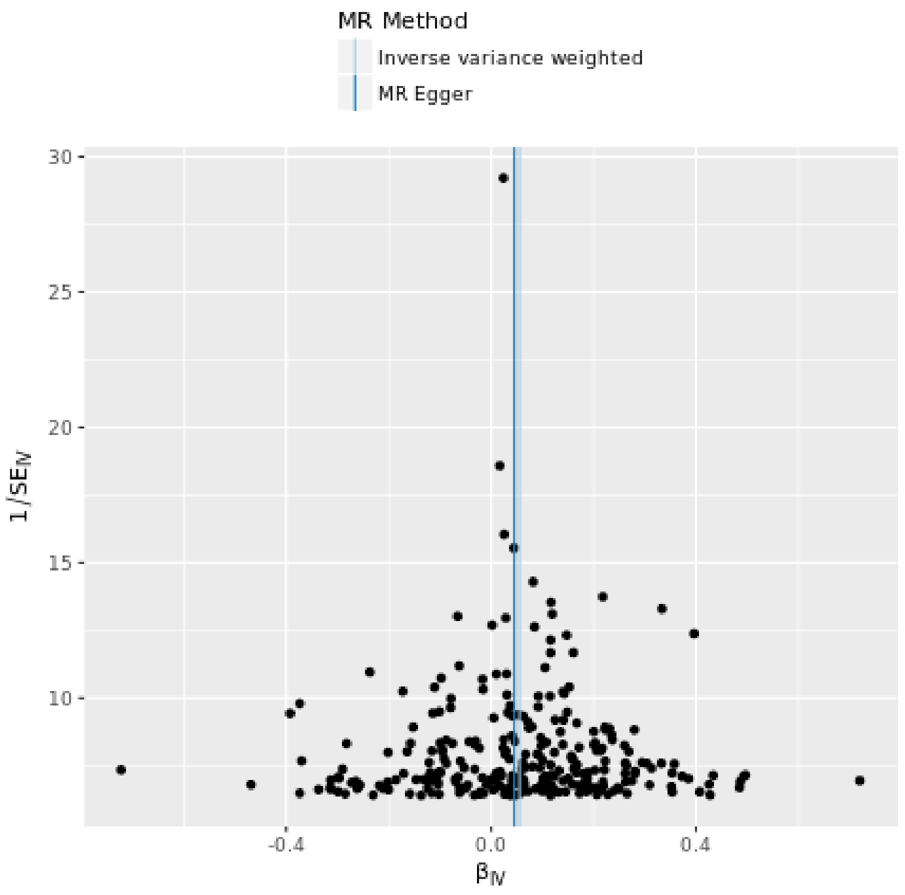

Funnel plot

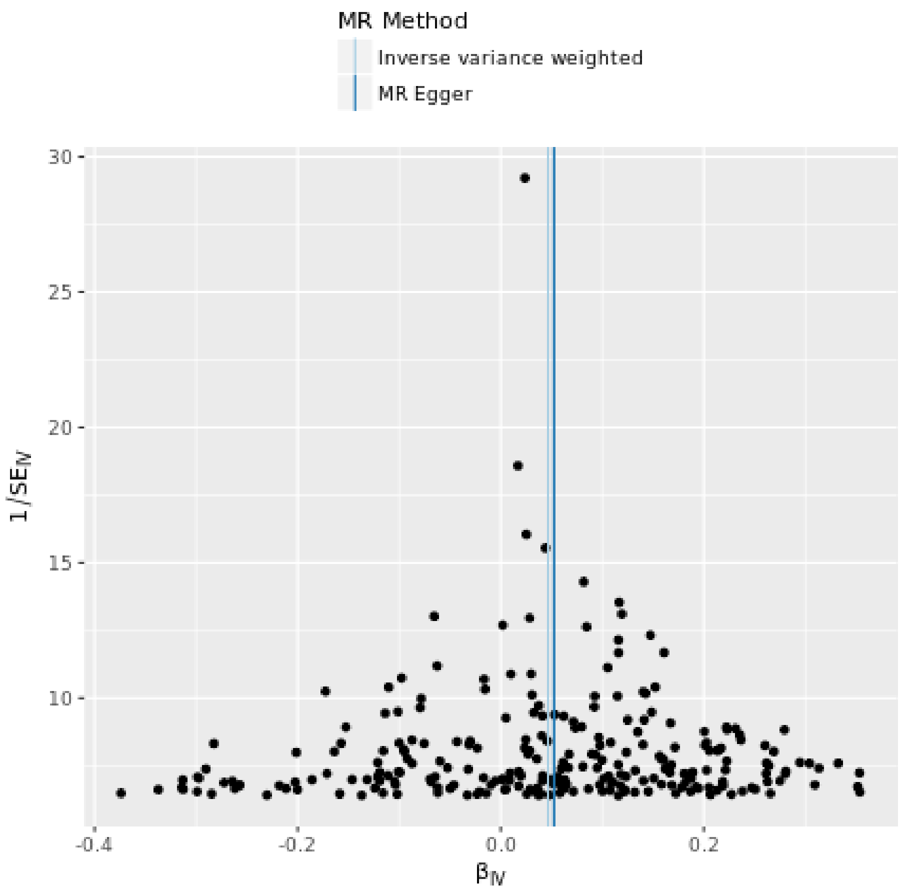

Leg fat mass (right)

Before removing instruments	After removing instruments
Leave-one-out plot<br>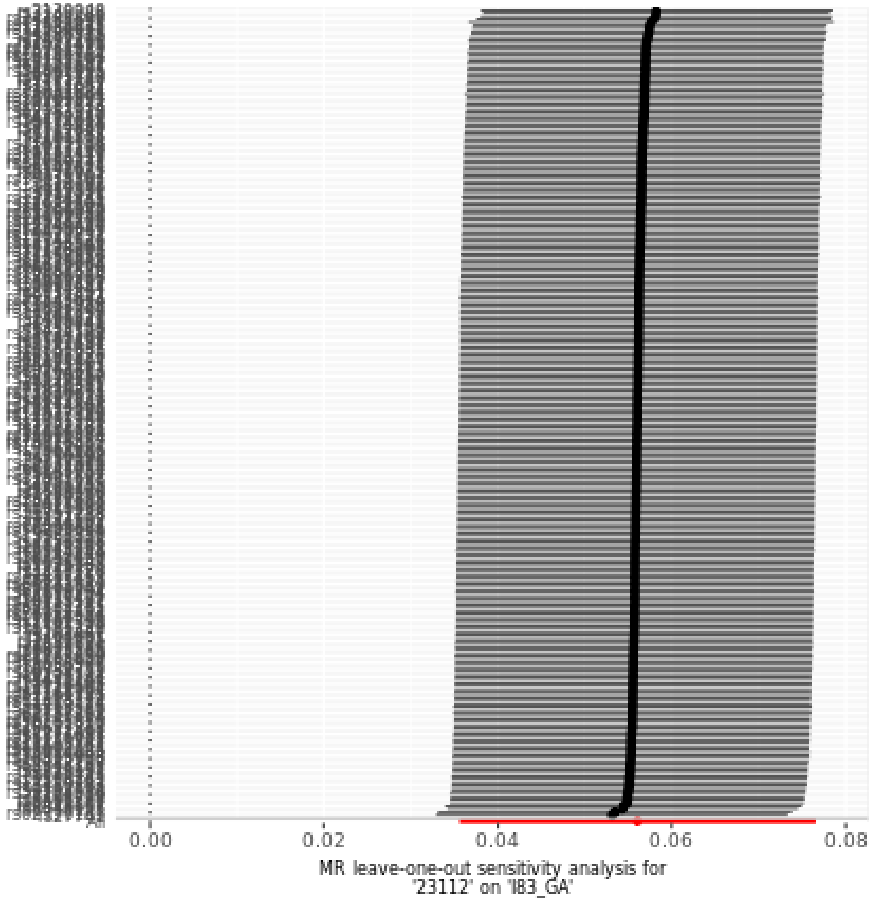 	Leave-one-out plot<br>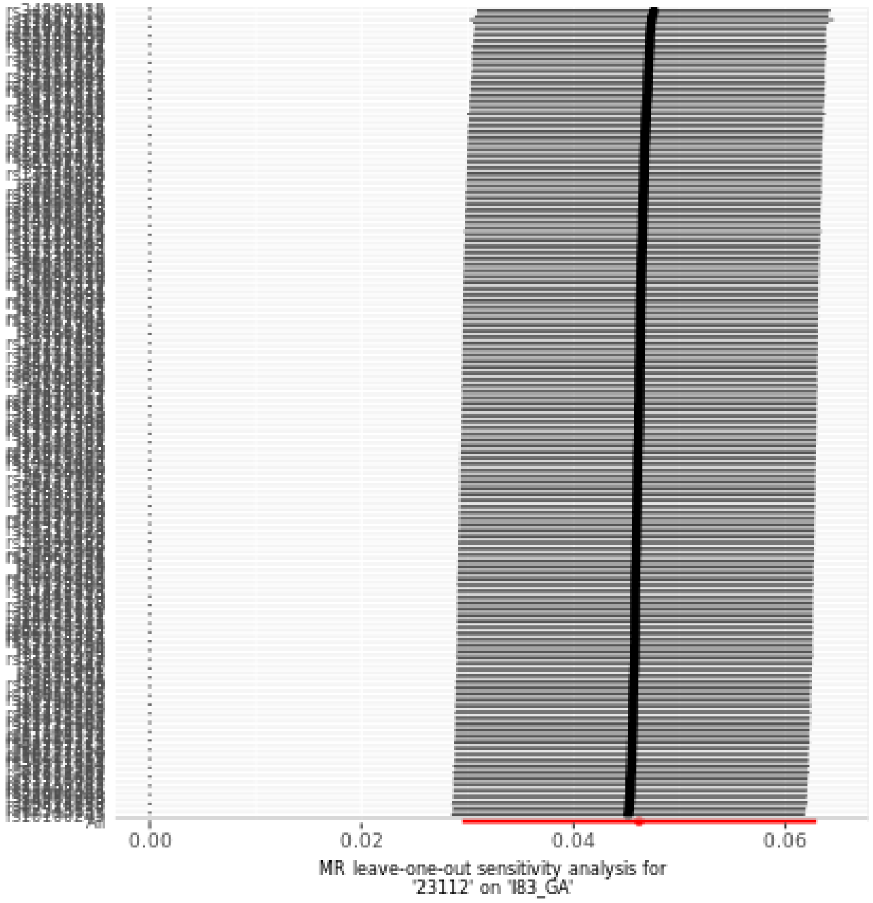

Funnel plot

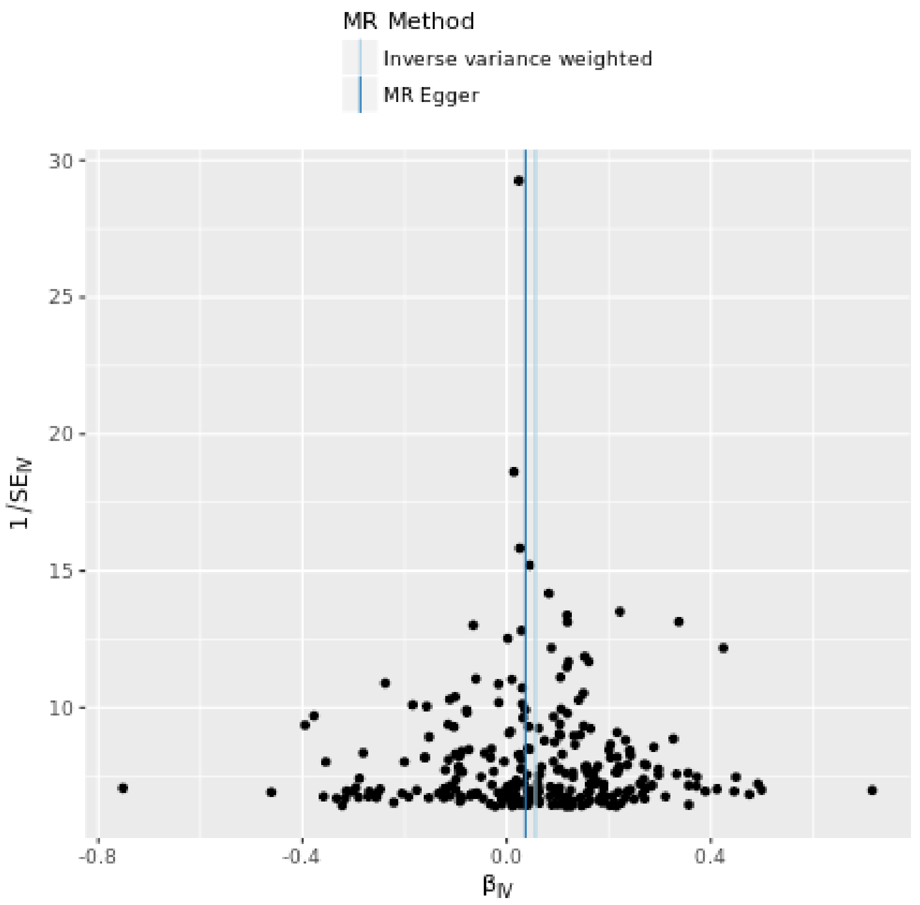

Funnel plot

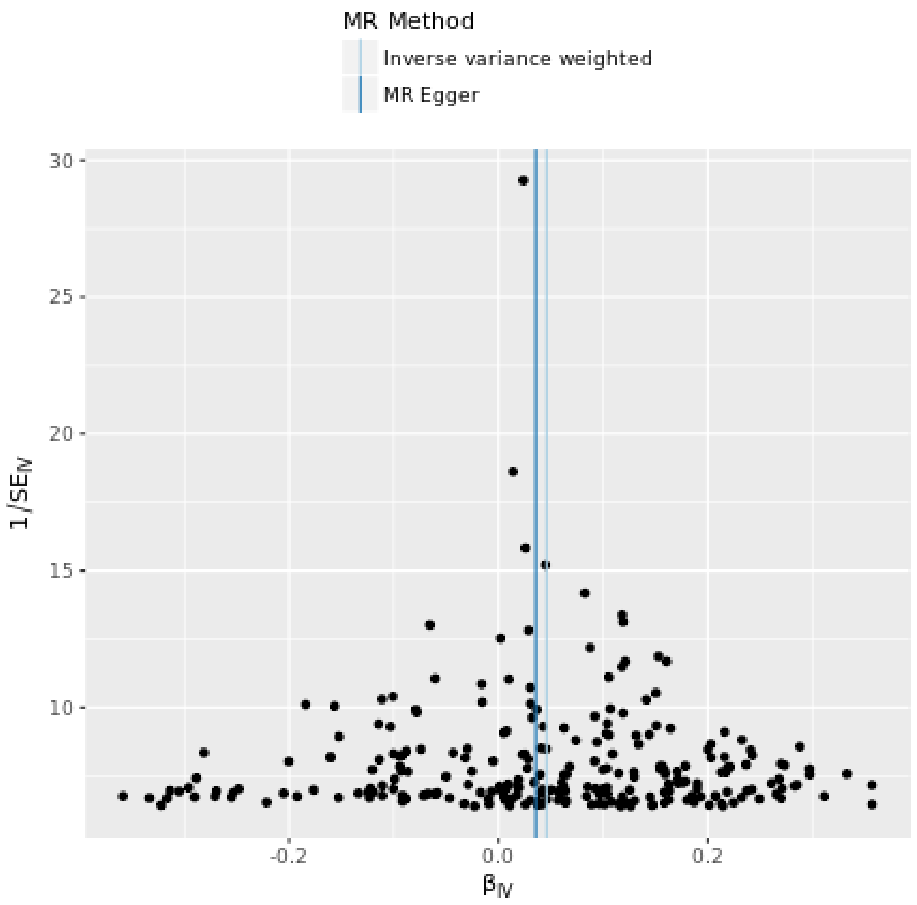

Forced vital capacity (FVC), Best measure

<table>
<tr><td>Before removing instruments</td><td>After removing instruments</td></tr>
<tr><td>Leave-one-out plot
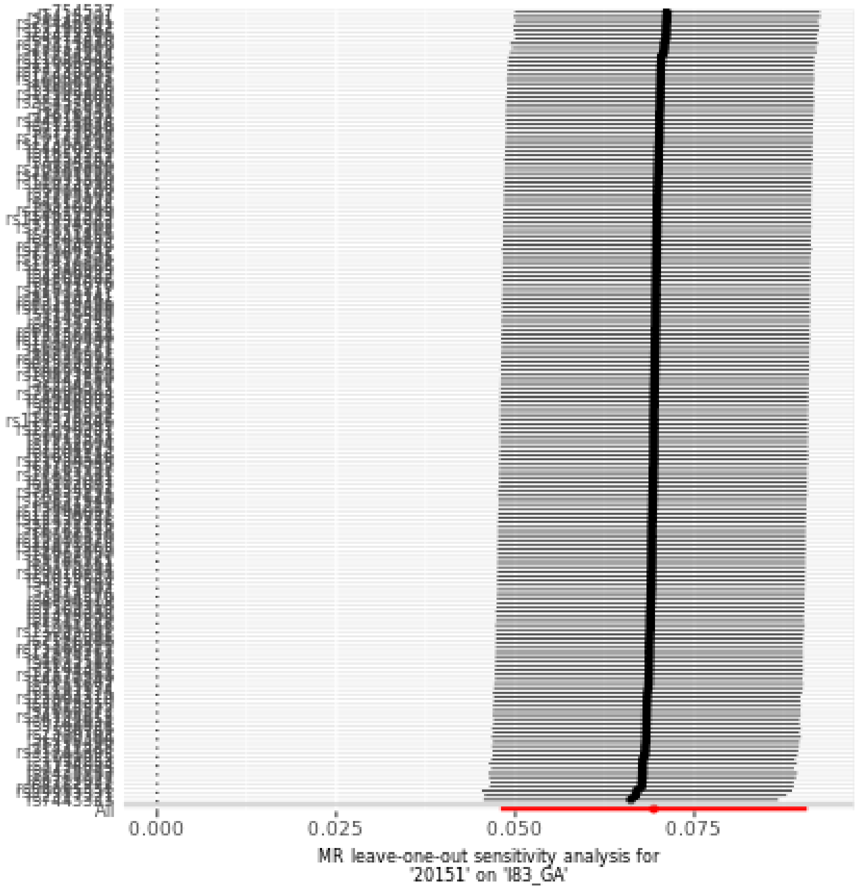

</td><td>Leave-one-out plot
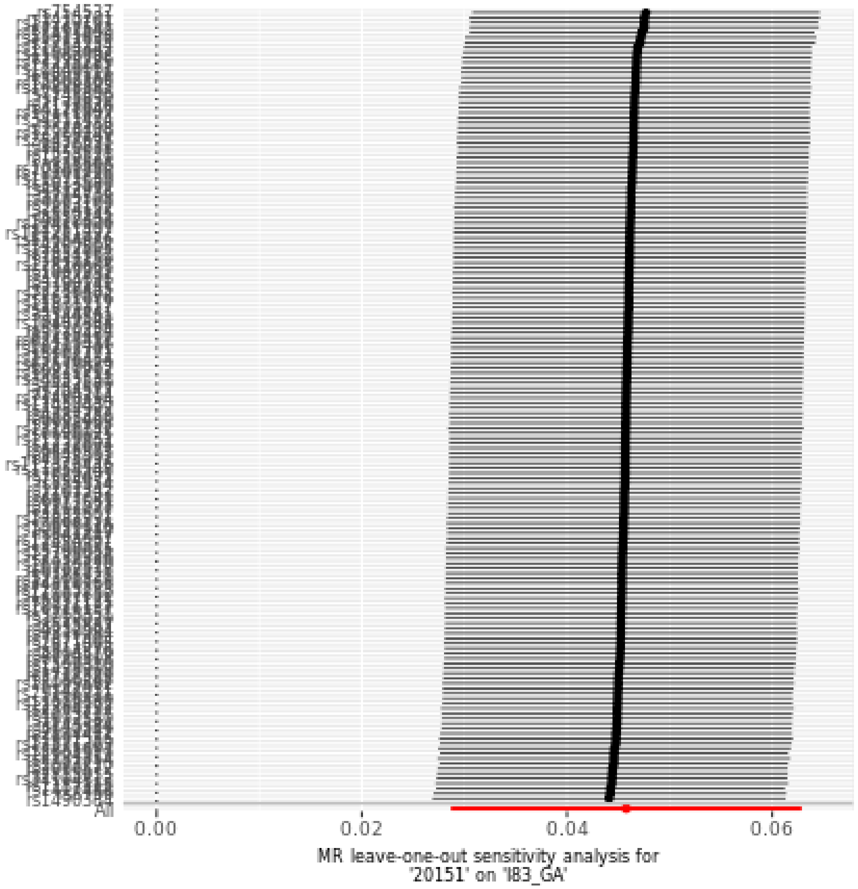

</td></tr>
</table>

Funnel plot

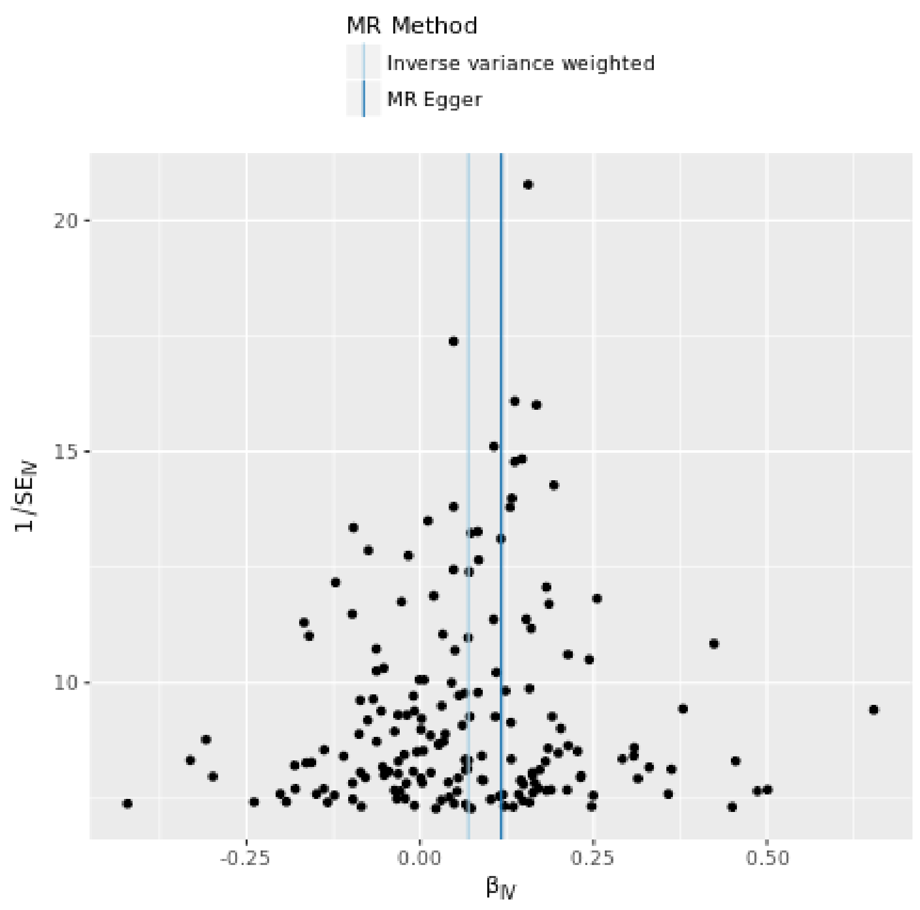

Funnel plot

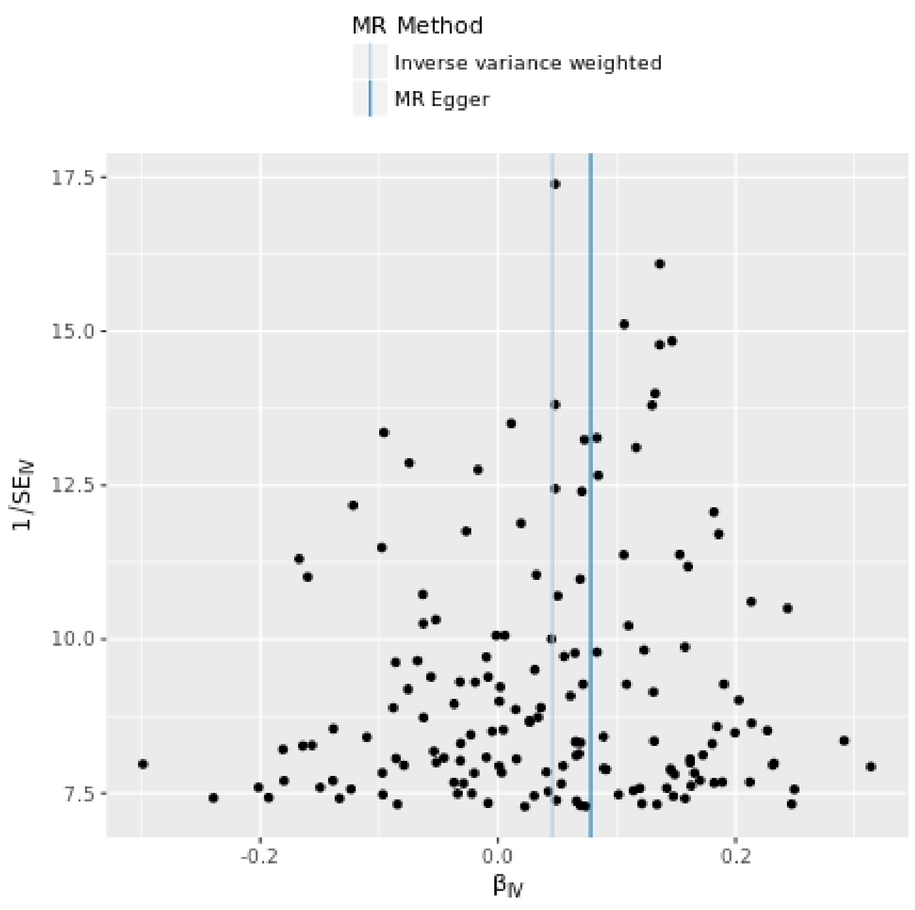

Trunk fat percentage

Before removing instruments	After removing instruments
Leave-one-out plot 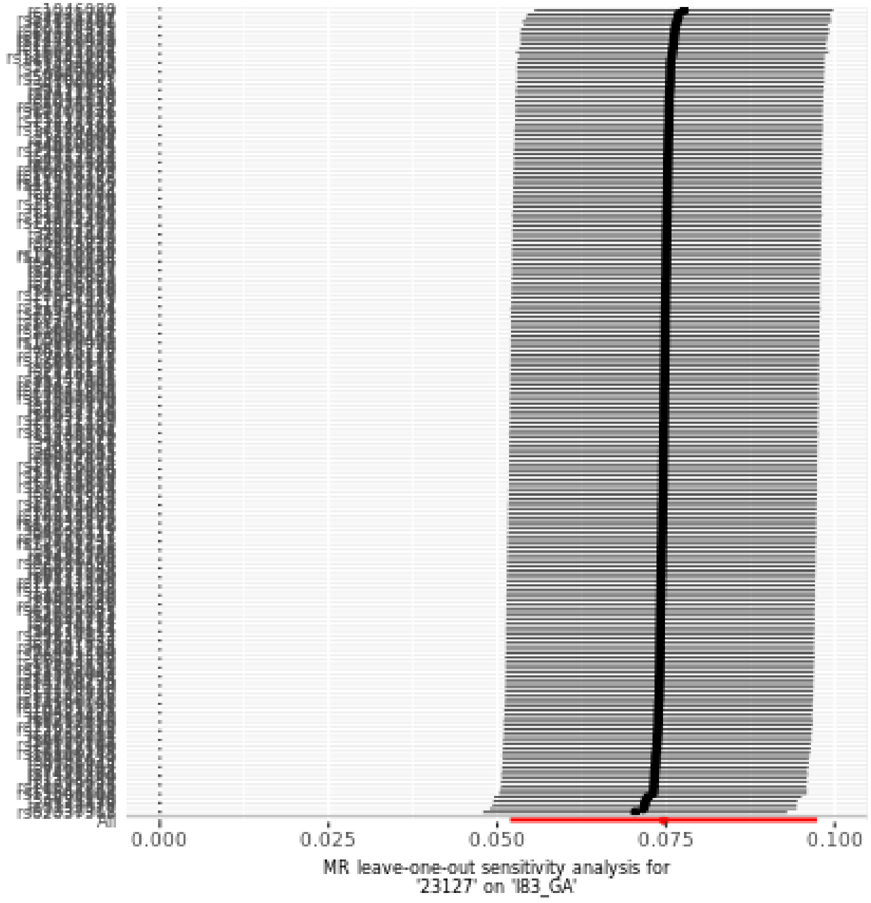	Leave-one-out plot 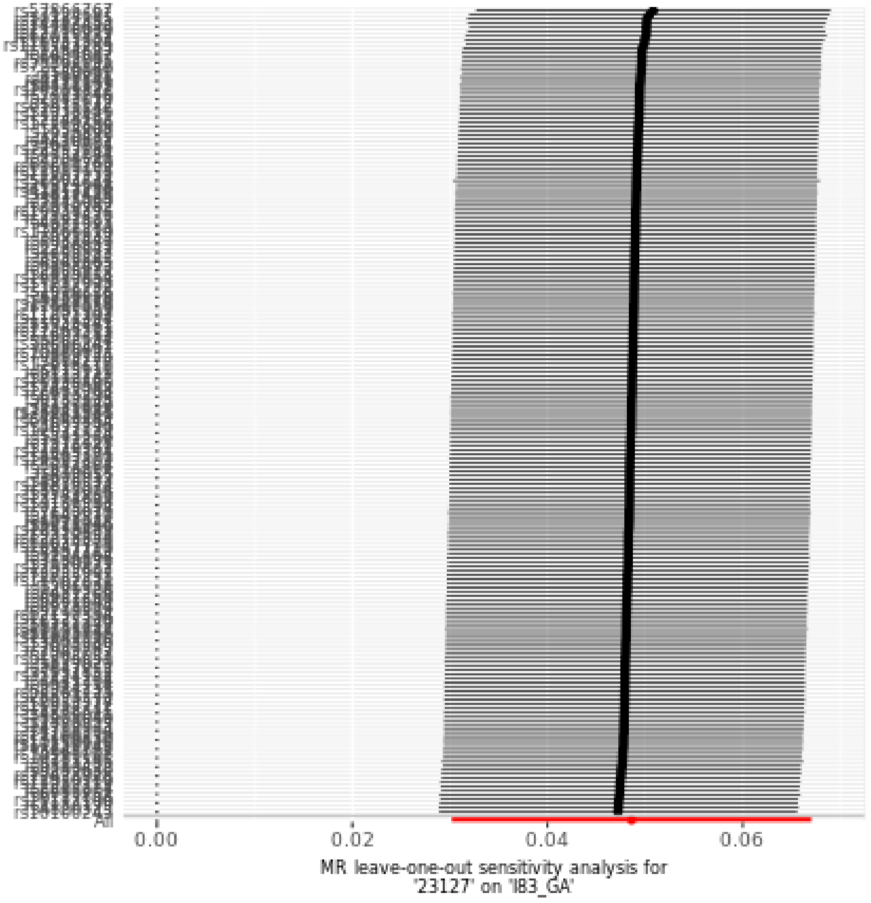

Funnel plot	Funnel plot
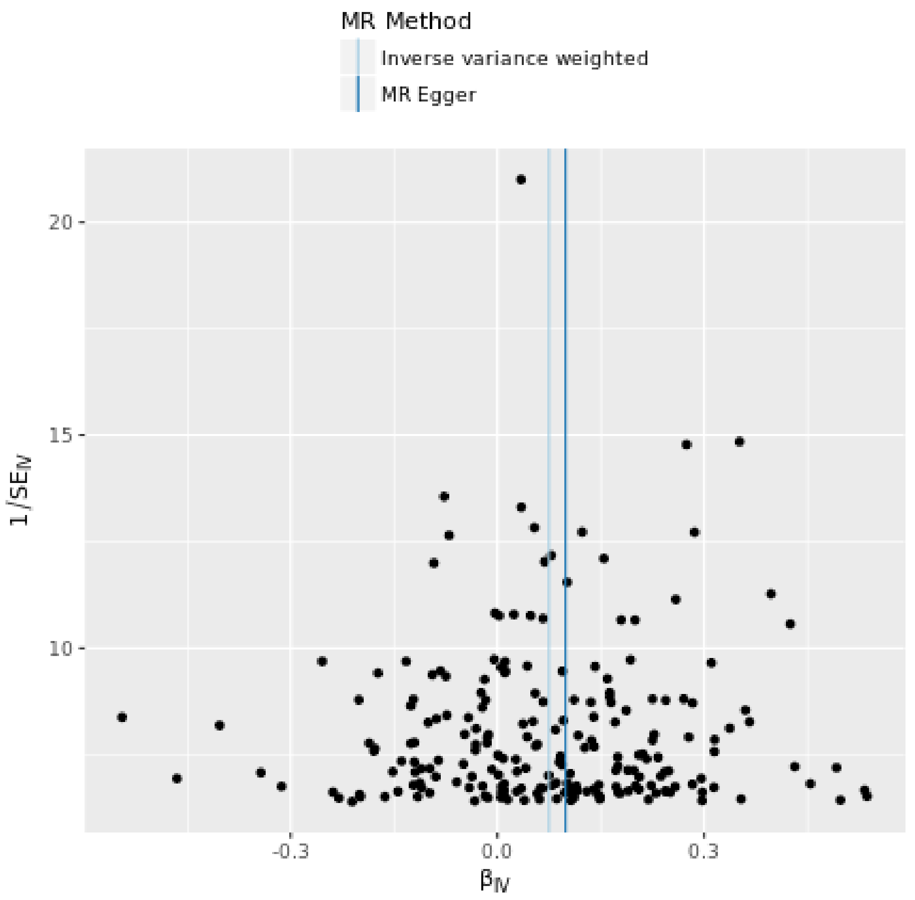	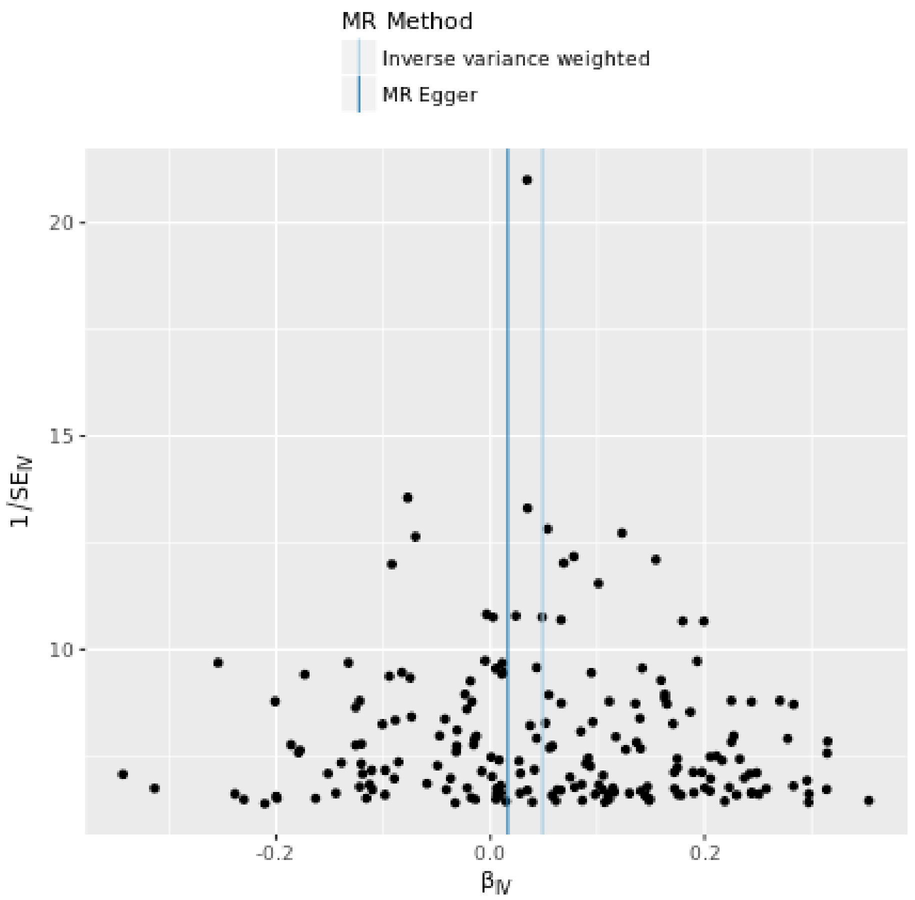

Waist circumference

Before removing instruments	After removing instruments
Leave-one-out plot<br>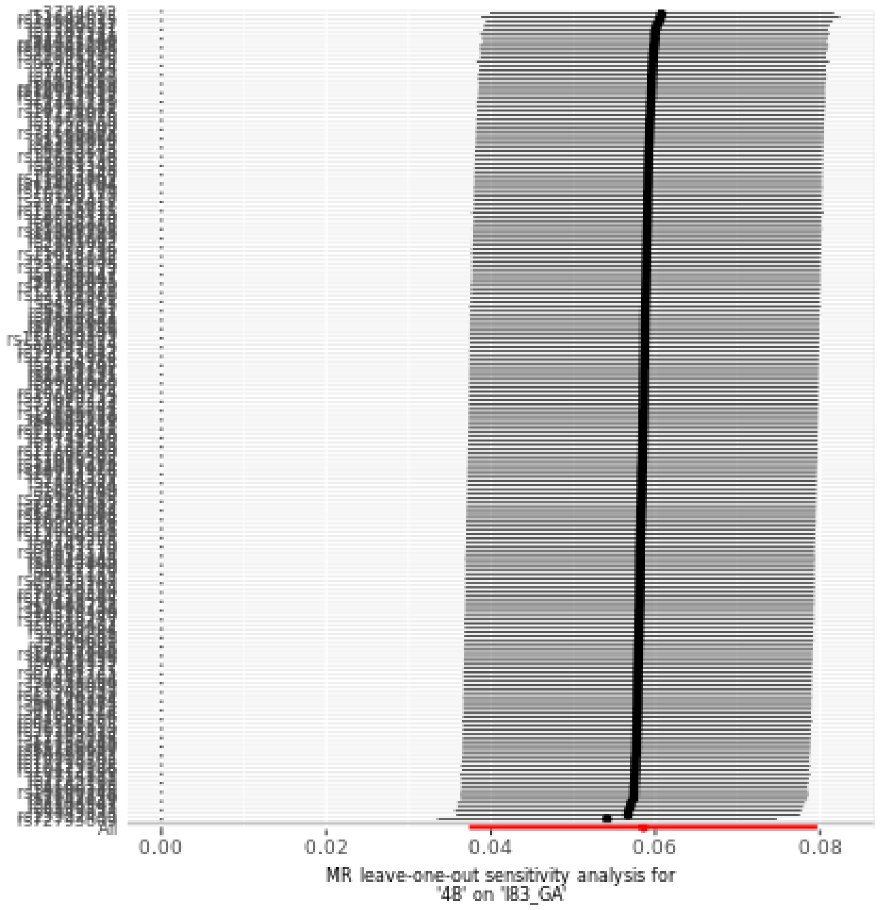 	Leave-one-out plot<br>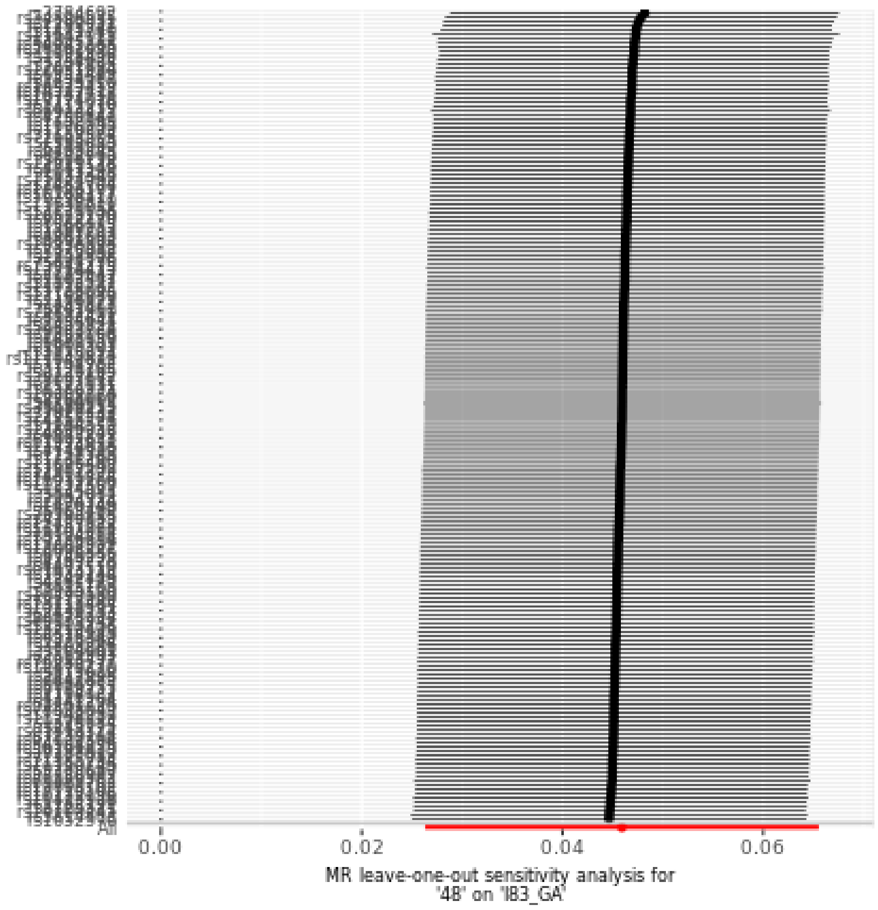

Funnel plot 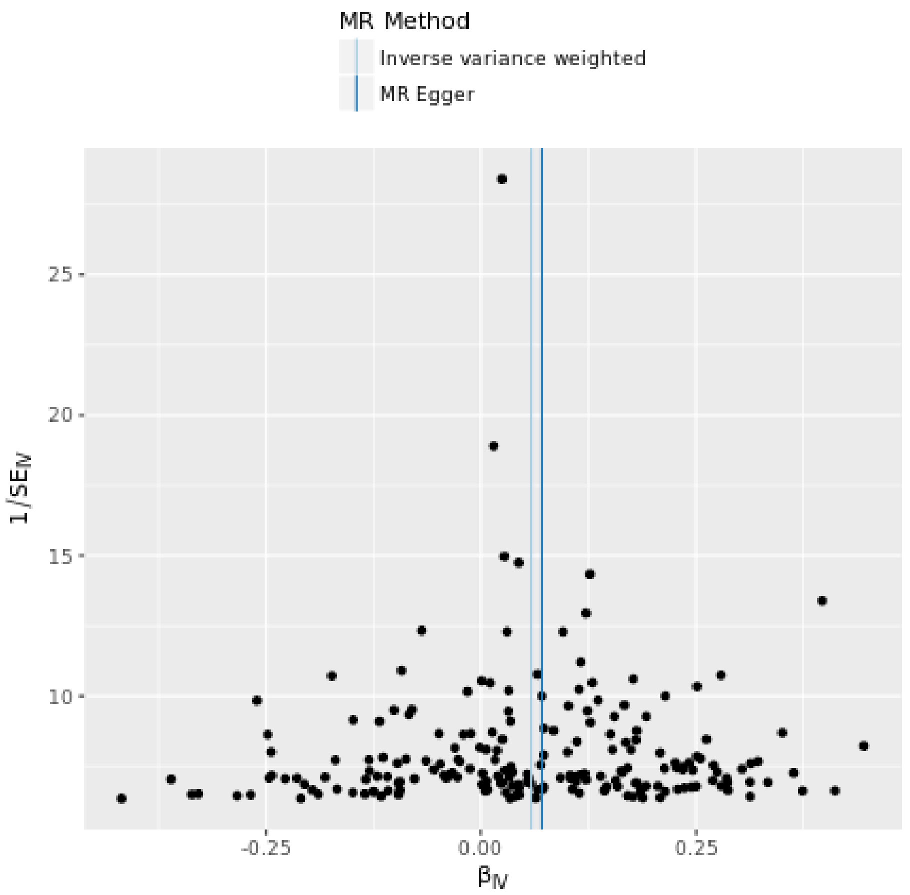	Funnel plot 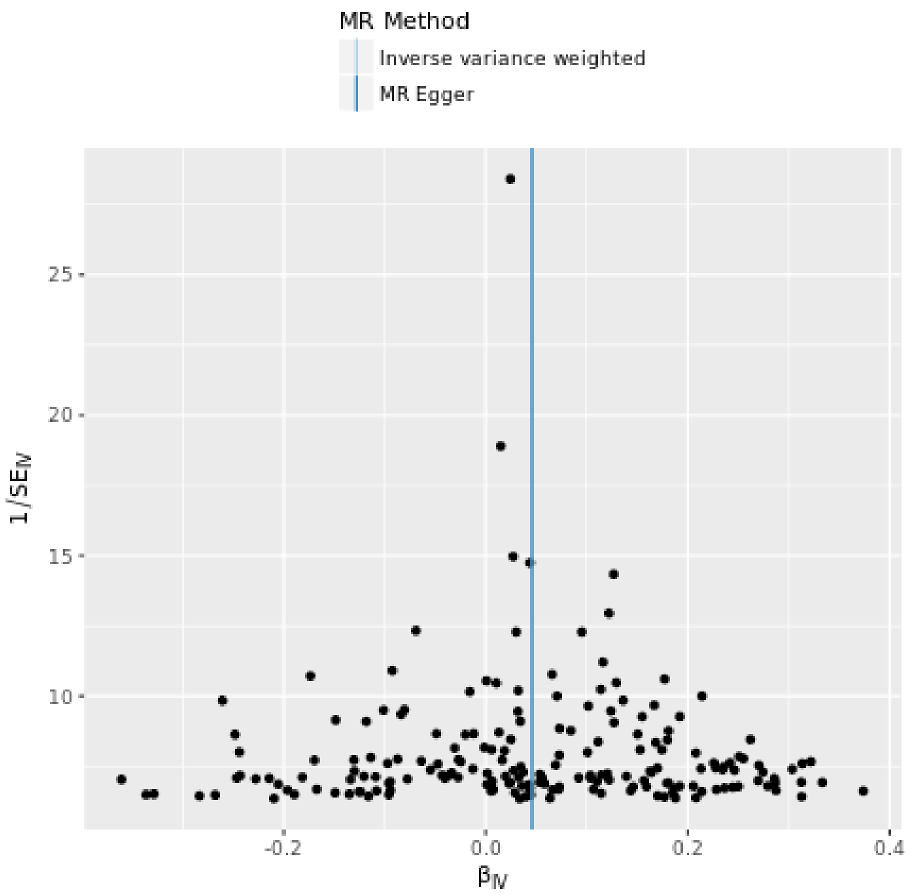

Arm fat percentage (right)

<table>
<tr><td>Before removing instruments</td><td>After removing instruments</td></tr>
<tr><td>Leave-one-out plot

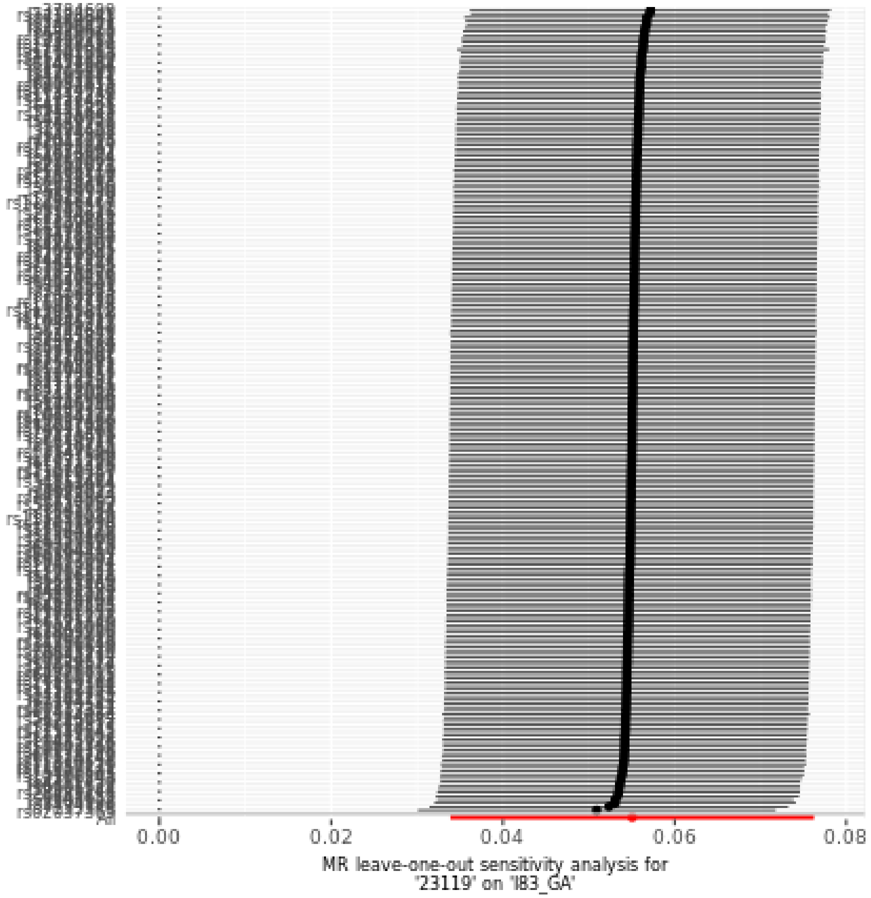

</td><td>Leave-one-out plot

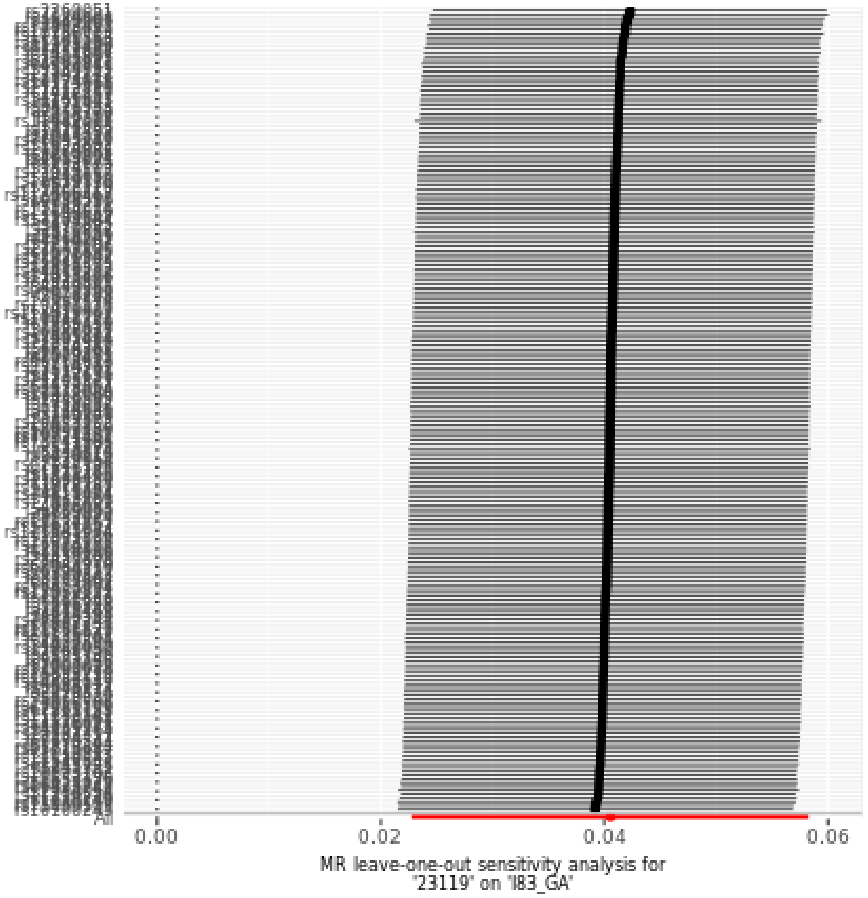

</td></tr>
</table>

Funnel plot	Funnel plot
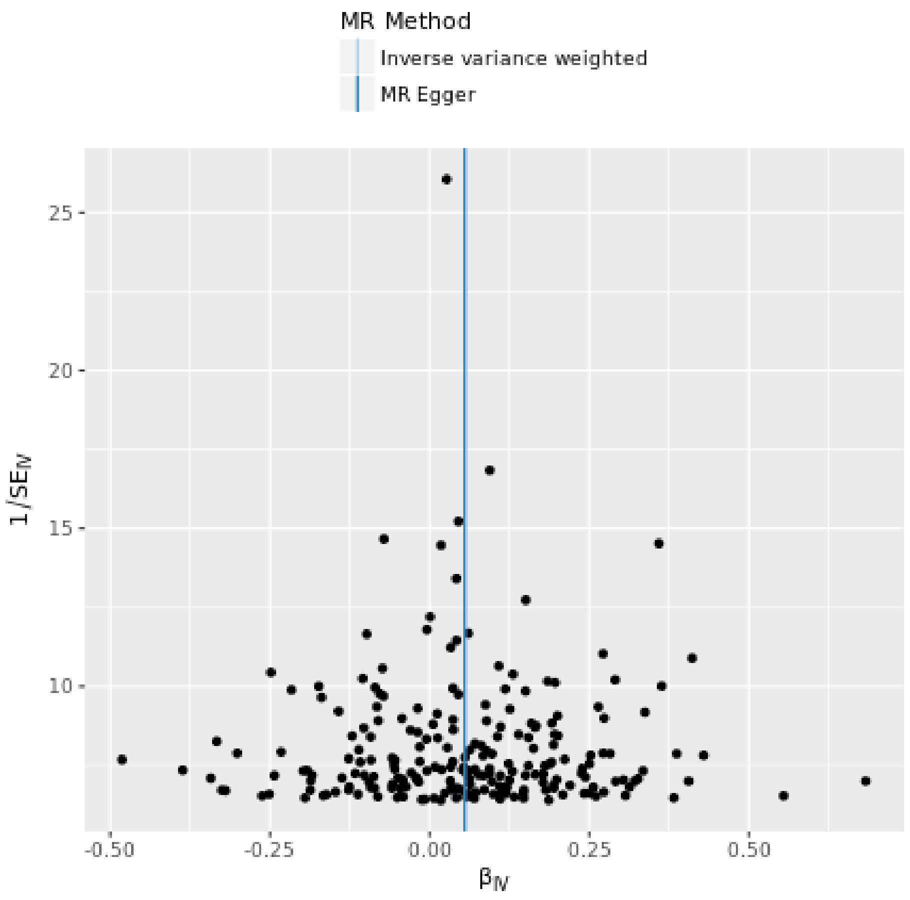	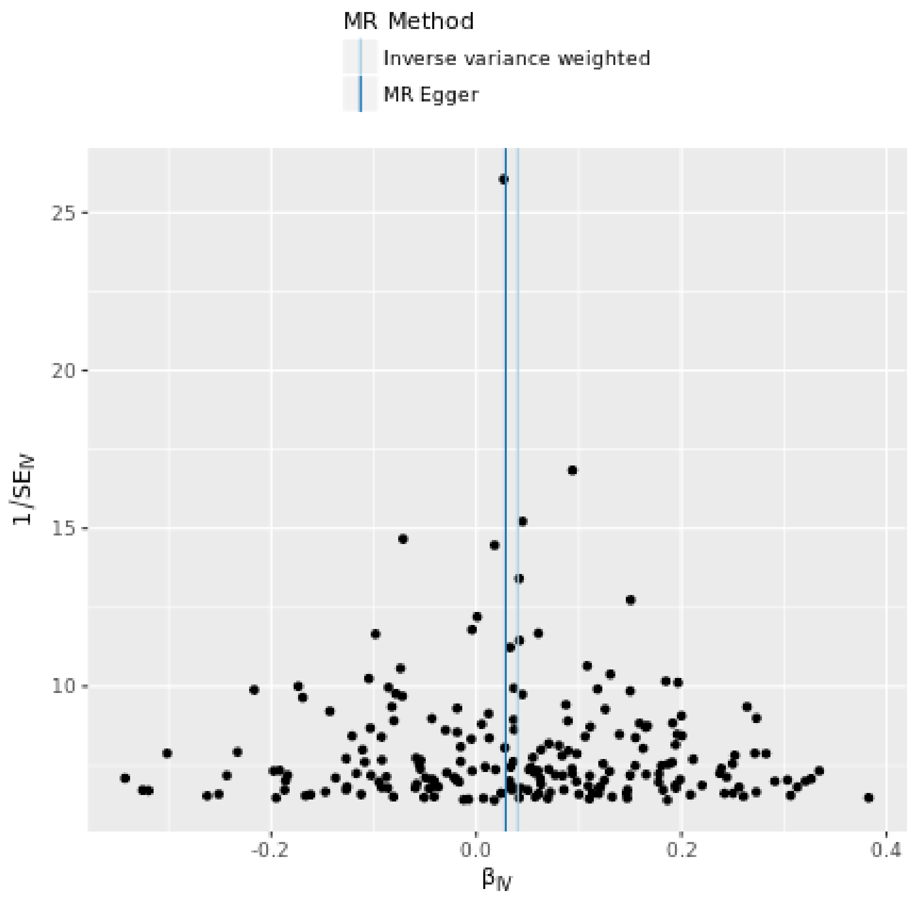

MHC class I polypeptide-related sequence B

Before removing instruments	After removing instruments
Leave-one-out plot 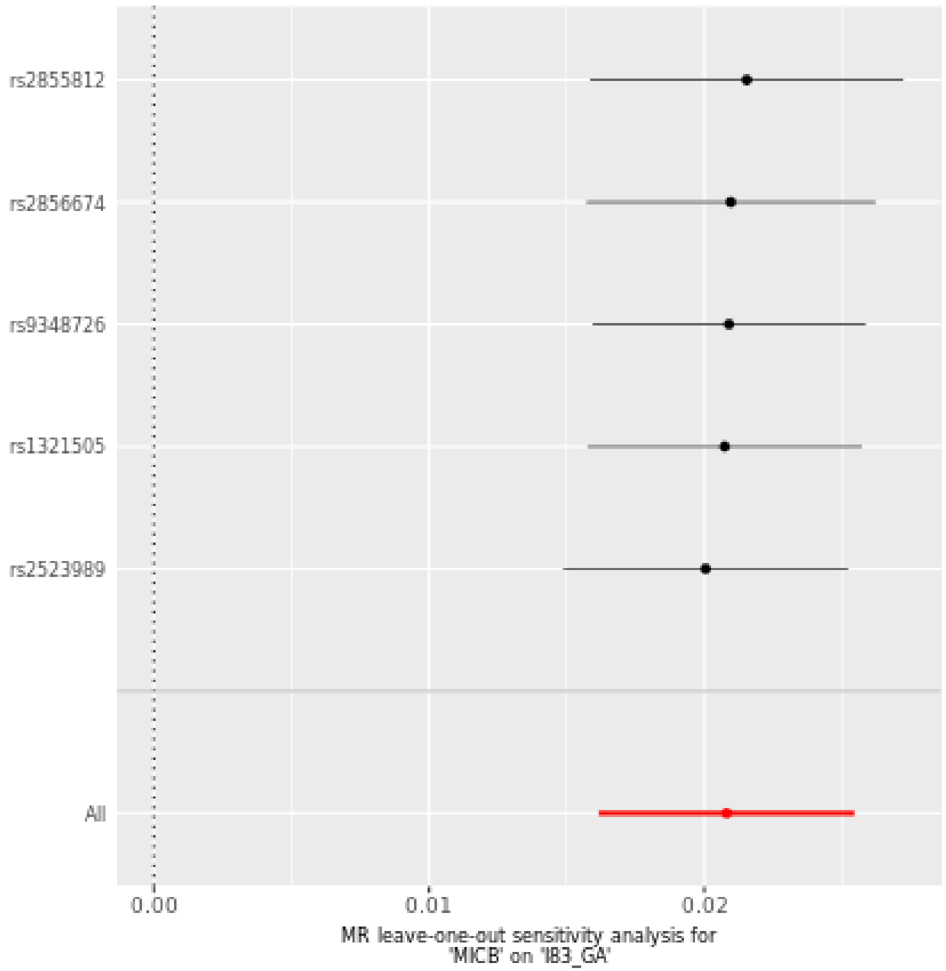	Leave-one-out plot<br>***N/A* (insufficient number of SNPs)**

Funnel plot	Funnel plot
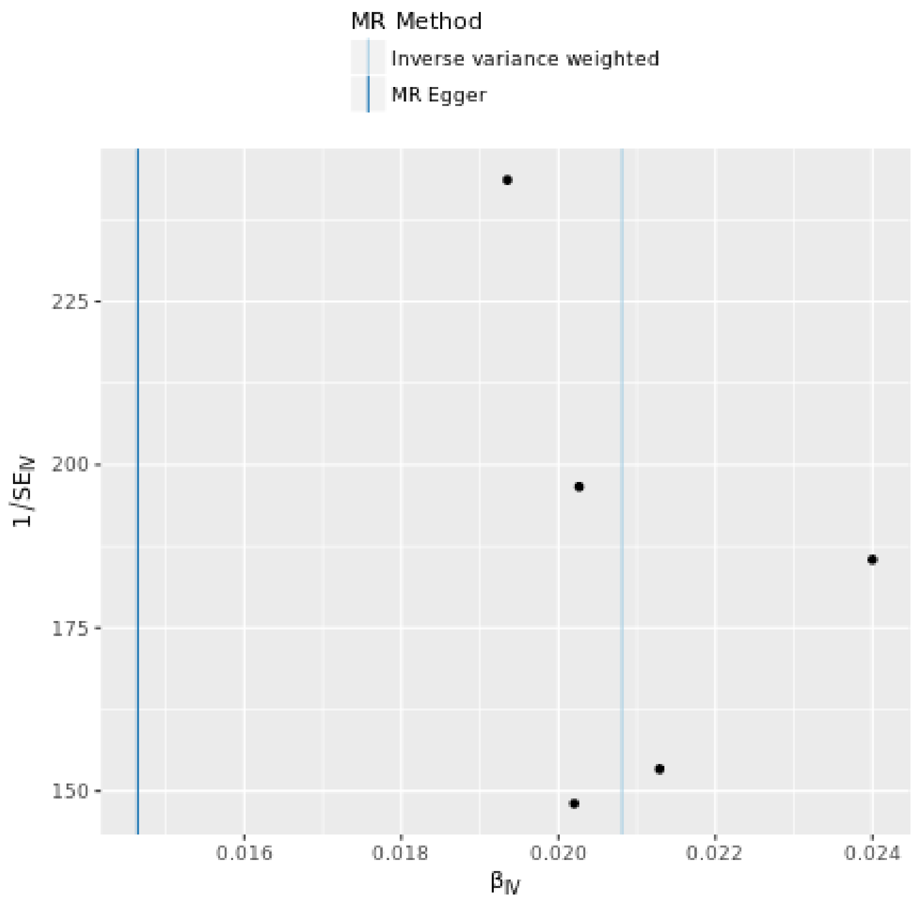	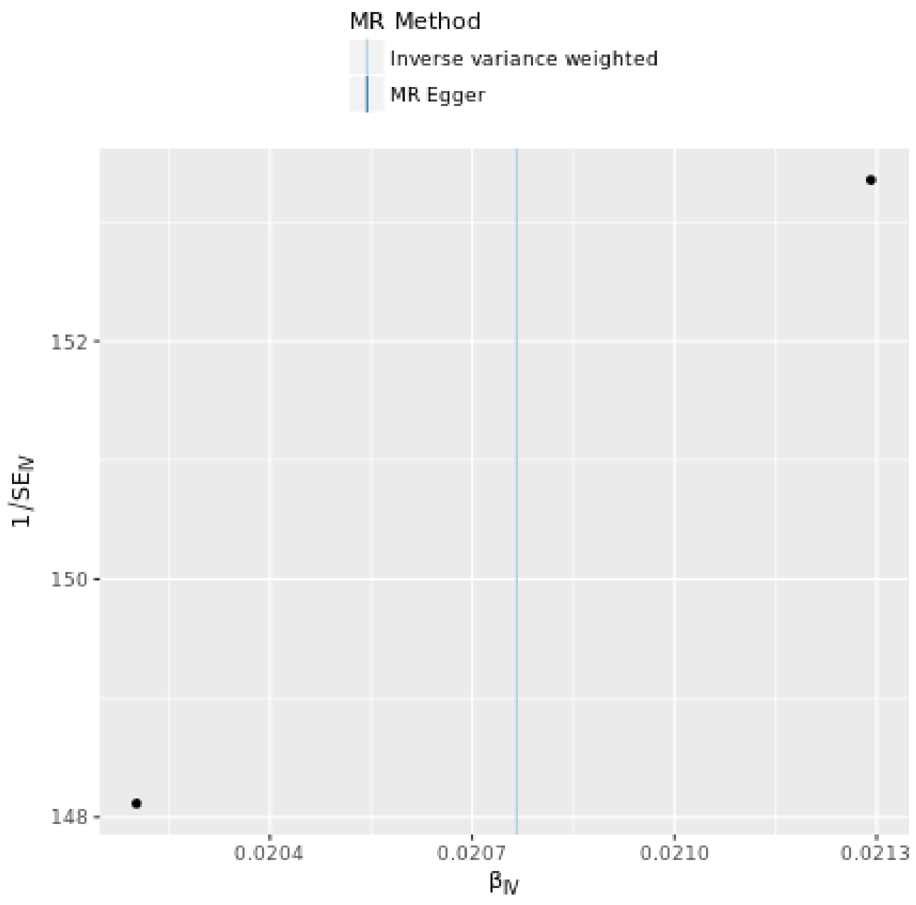

Forced vital capacity (FVC)

Before removing instruments	After removing instruments
Leave-one-out plot<br>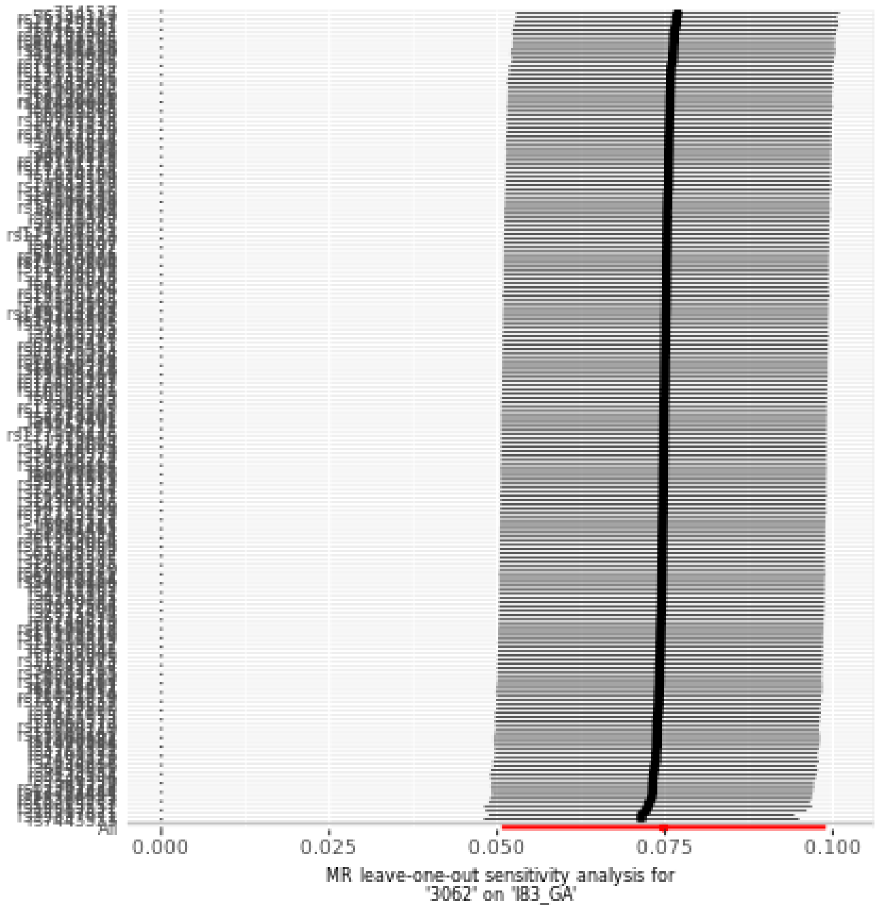	Leave-one-out plot<br>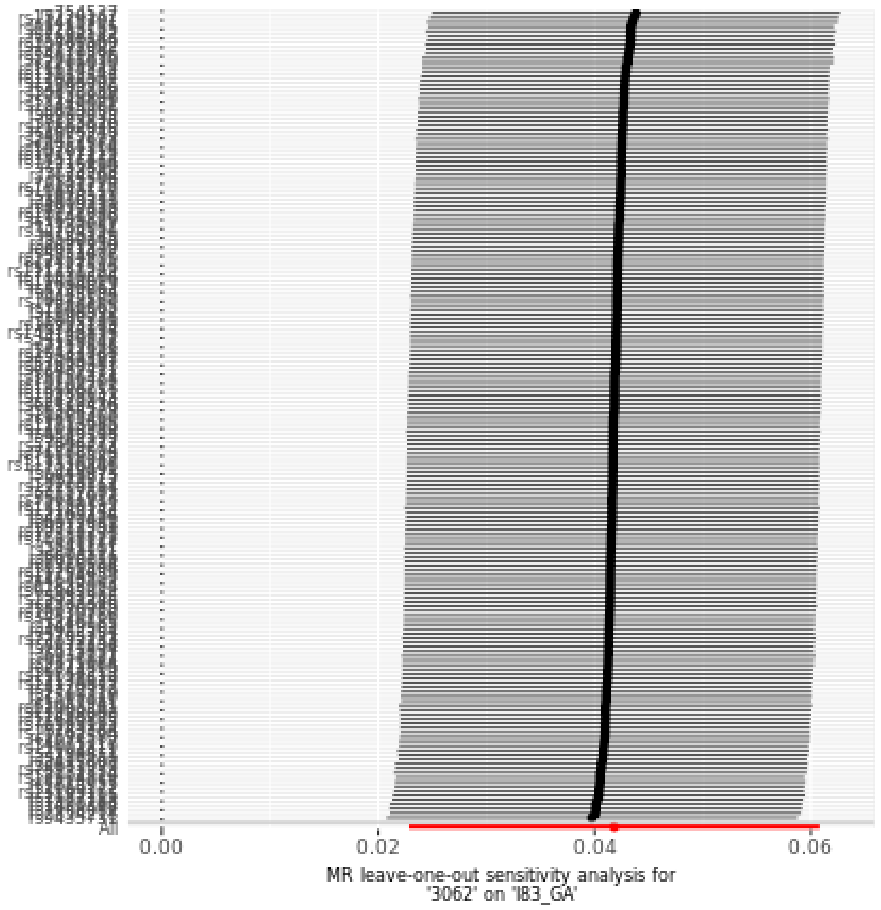

Funnel plot

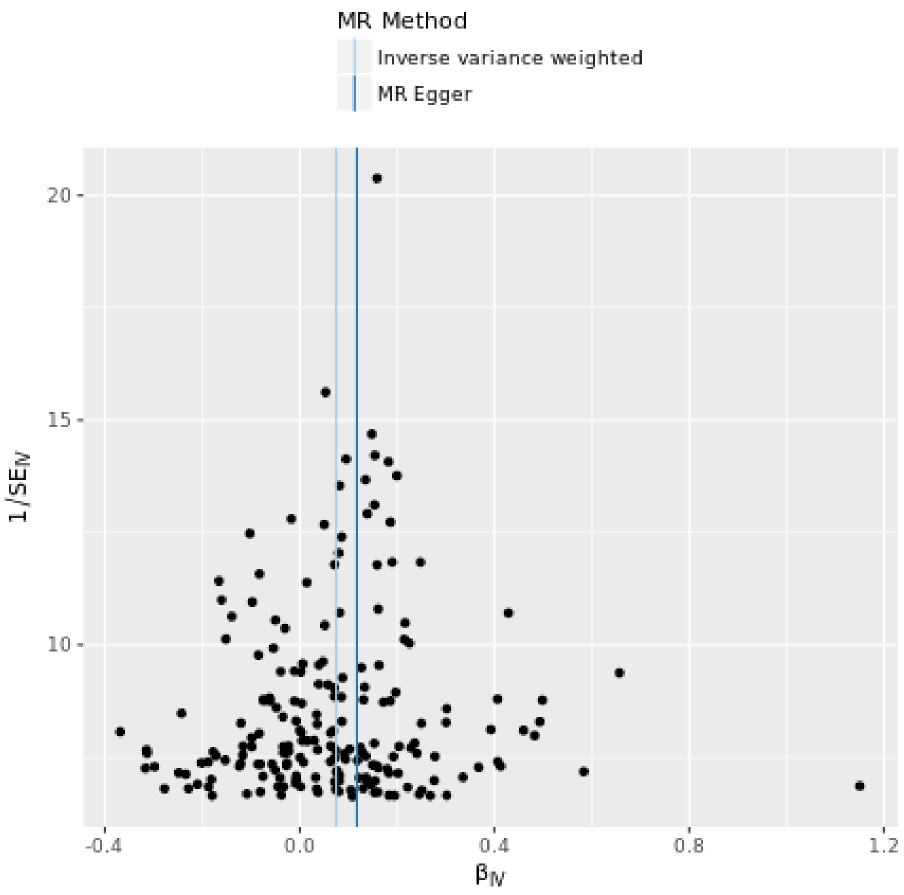

Funnel plot

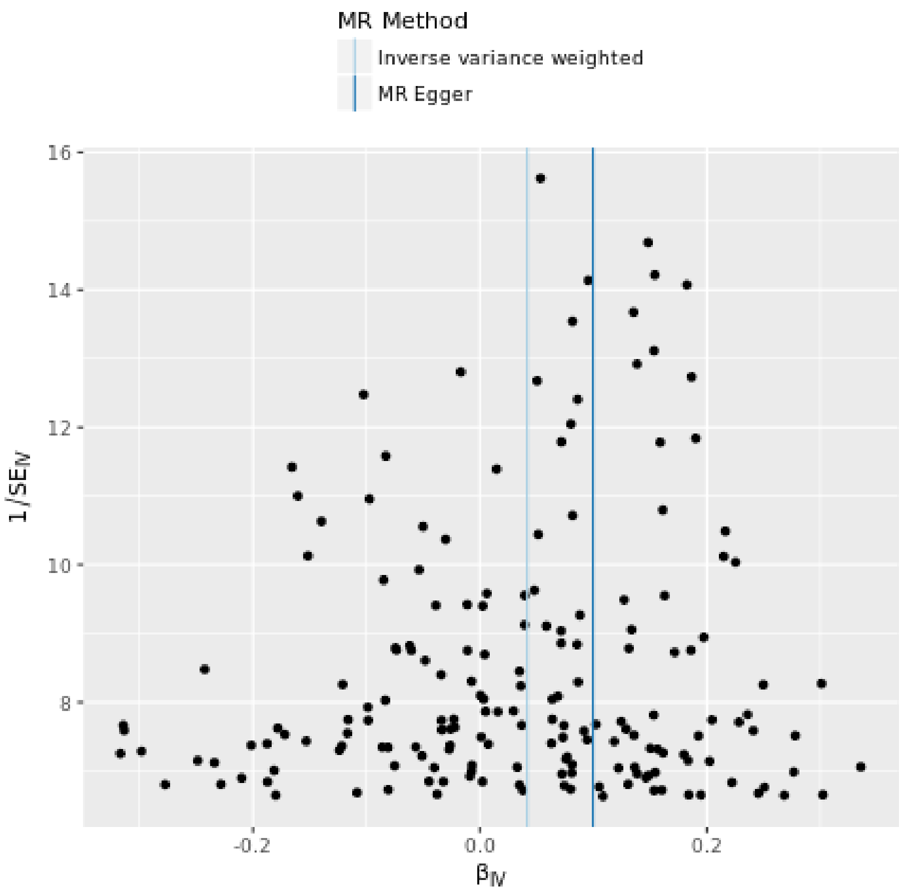

Arm fat percentage (left)

<table>
<tr><td>Before removing instruments</td><td>After removing instruments</td></tr>
<tr><td>Leave-one-out plot

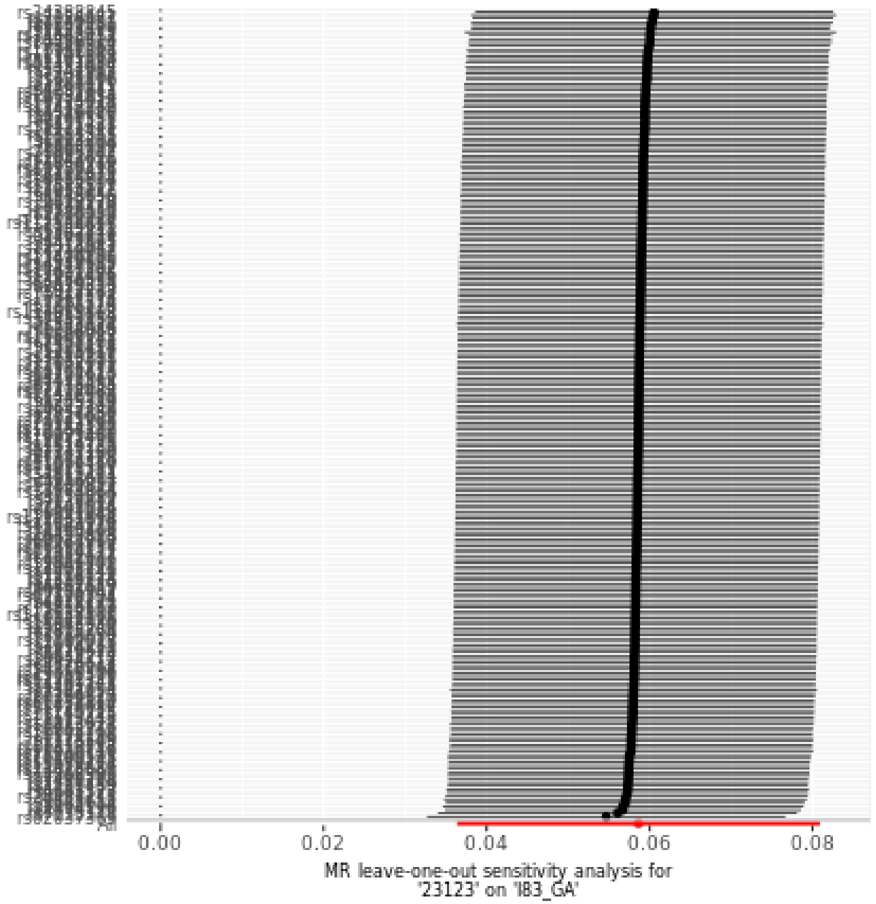

</td><td>Leave-one-out plot

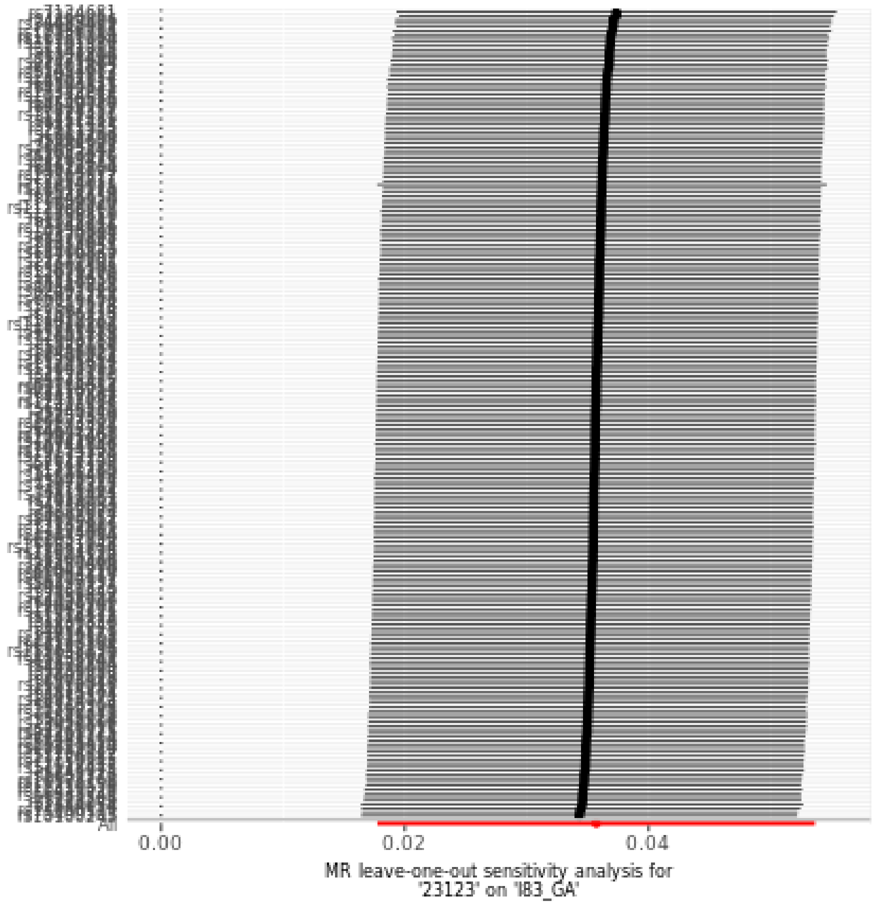

</td></tr>
</table>

Funnel plot

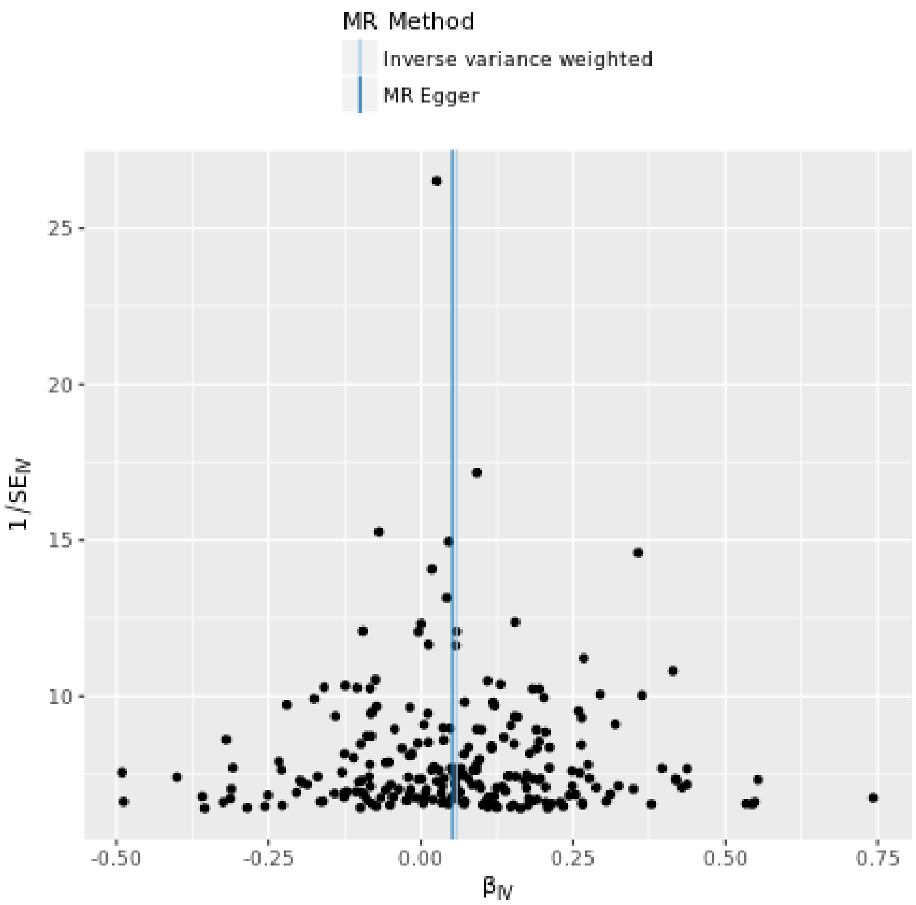

Funnel plot

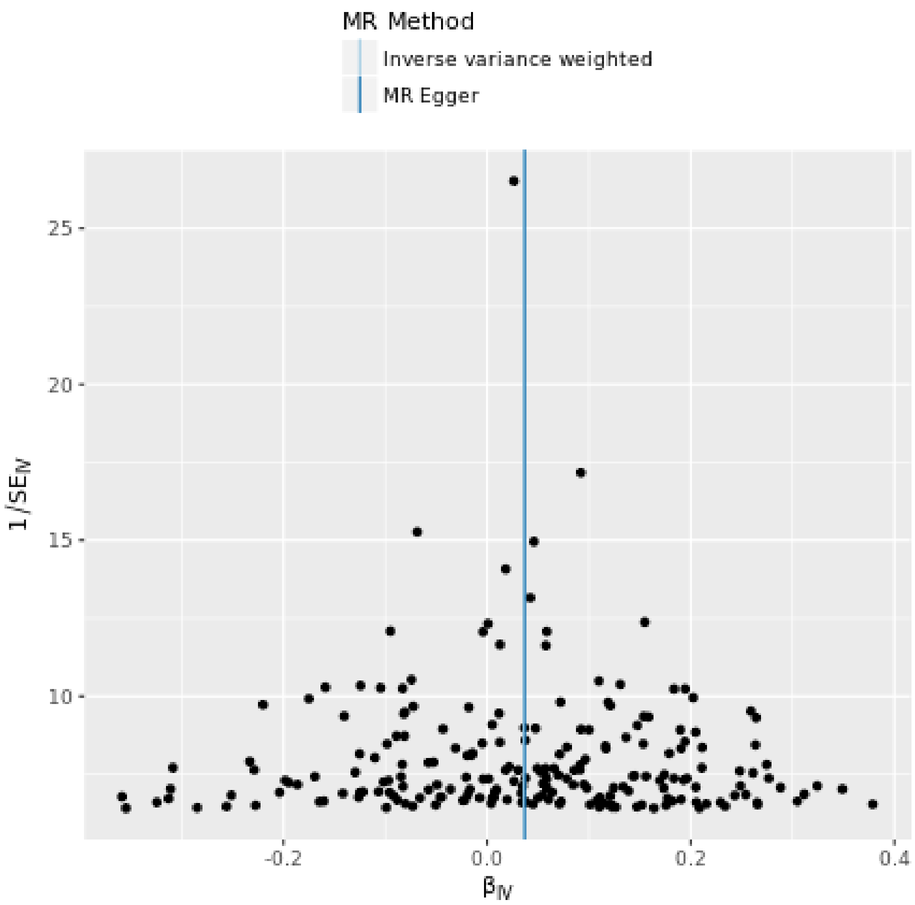

Impedance of leg (left)

Before removing instruments	After removing instruments
Leave-one-out plot<br>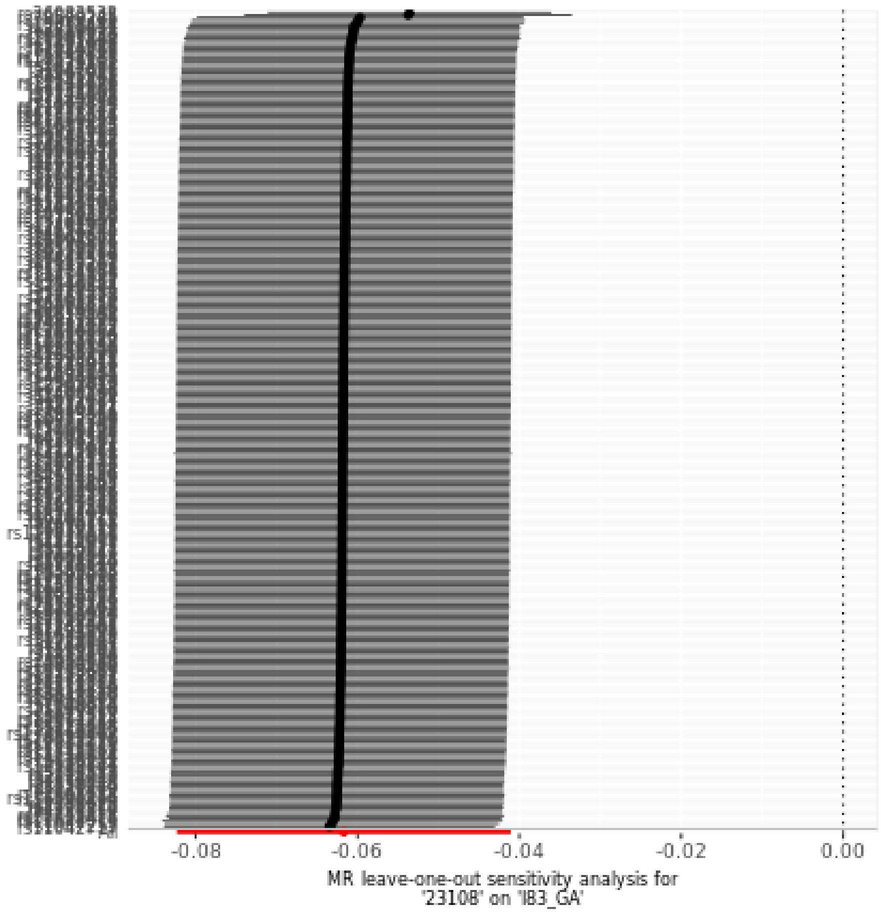 	Leave-one-out plot<br>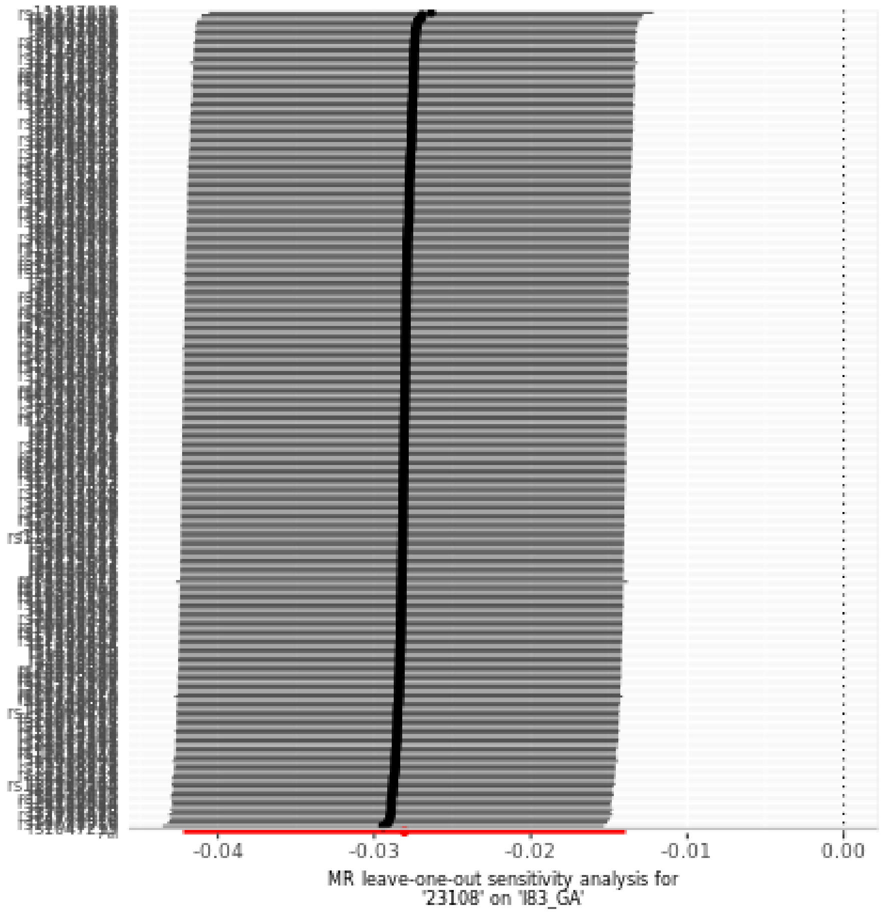

Funnel plot	Funnel plot
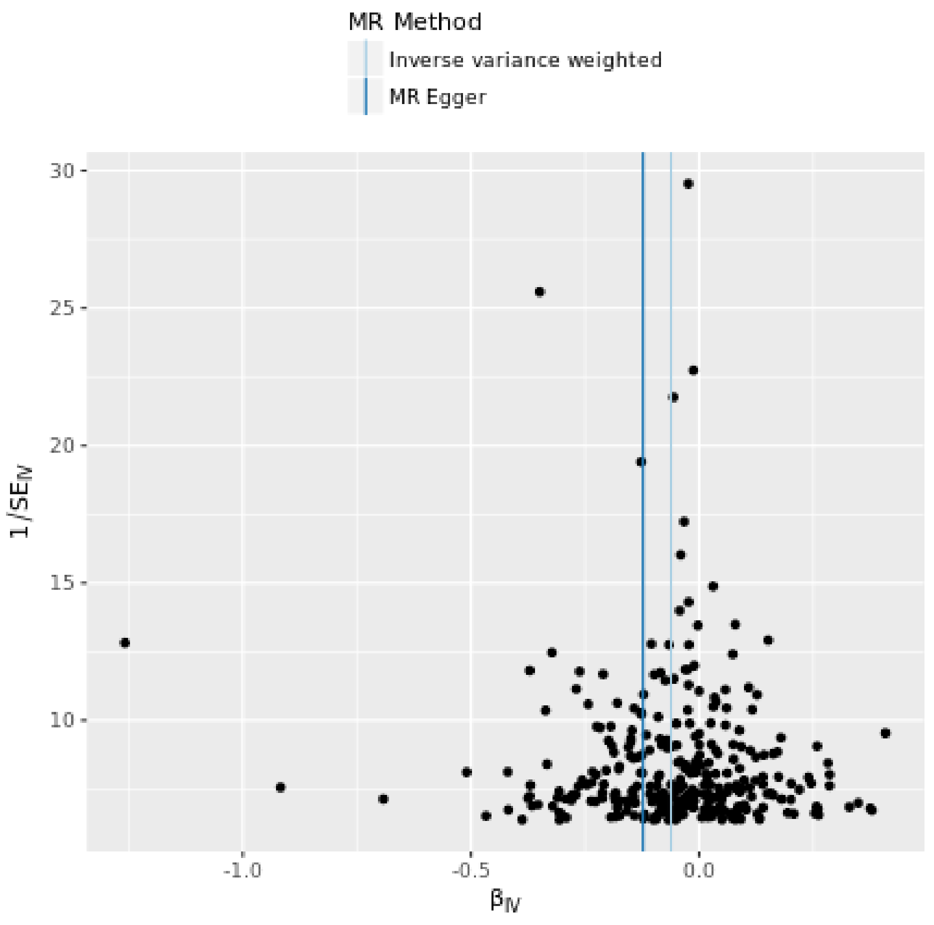	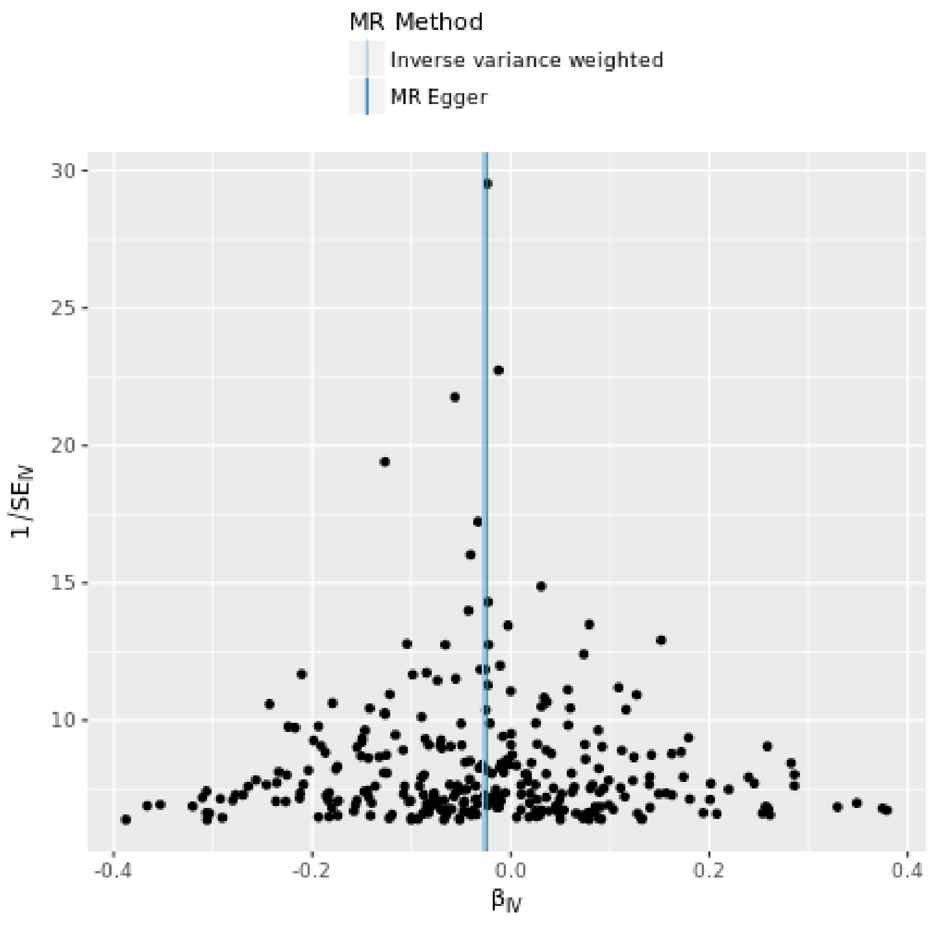

Impedance of leg (right)

Before removing instruments	After removing instruments
Leave-one-out plot<br>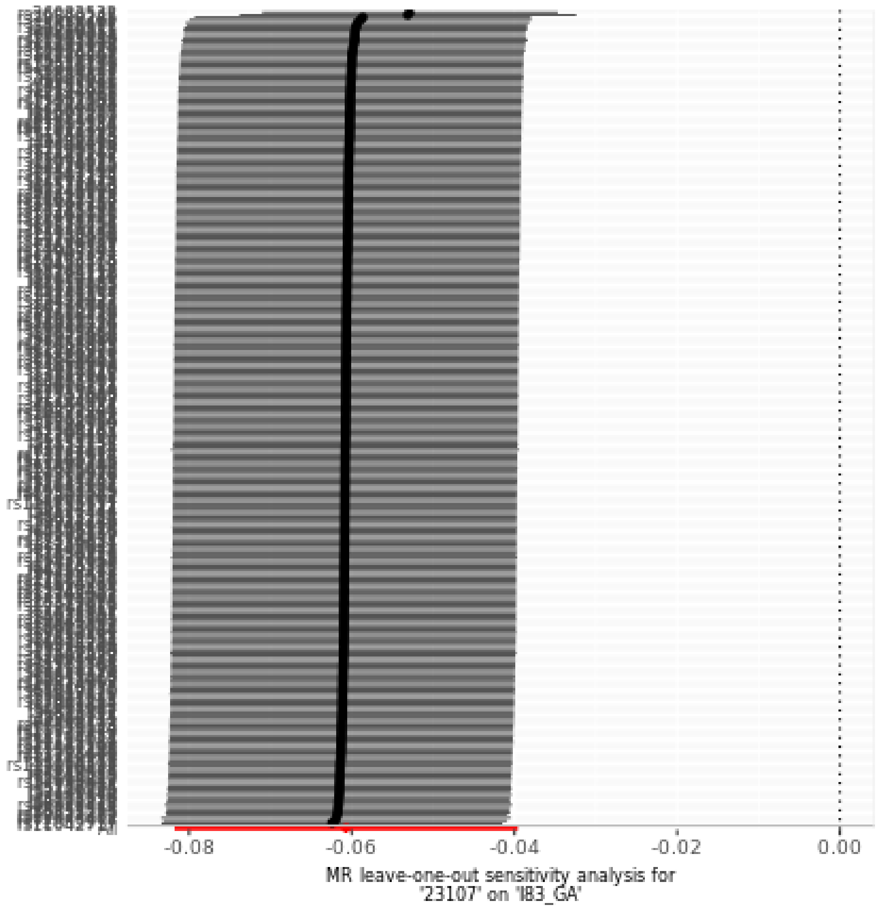	Leave-one-out plot<br>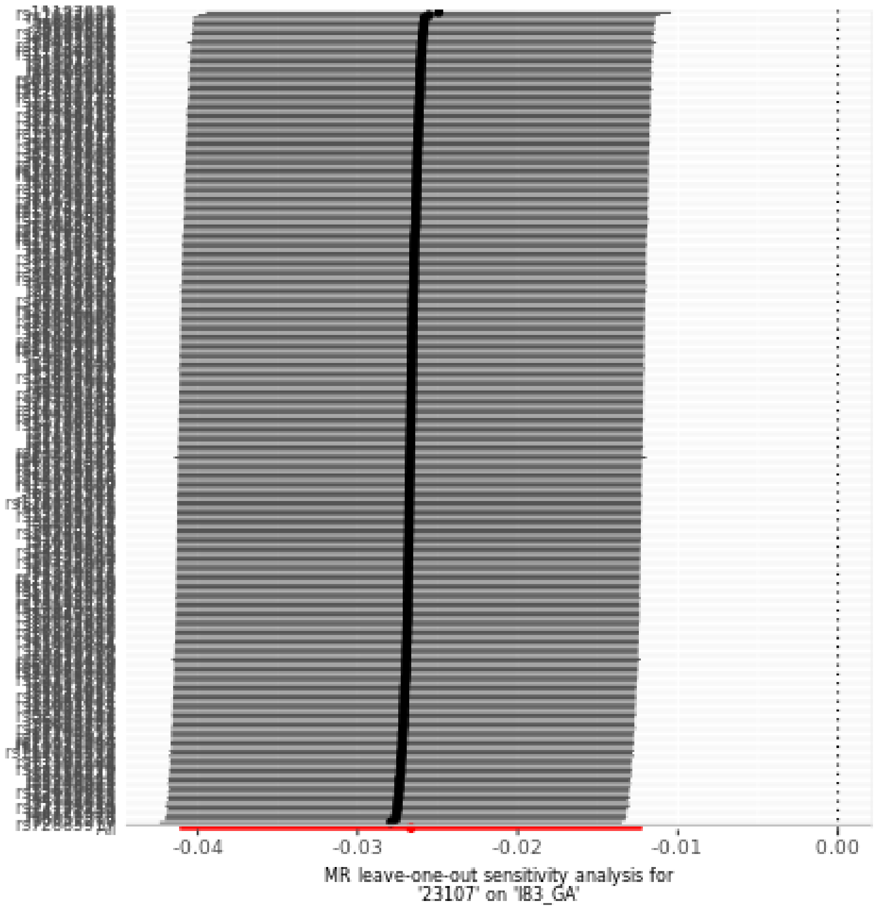

Funnel plot

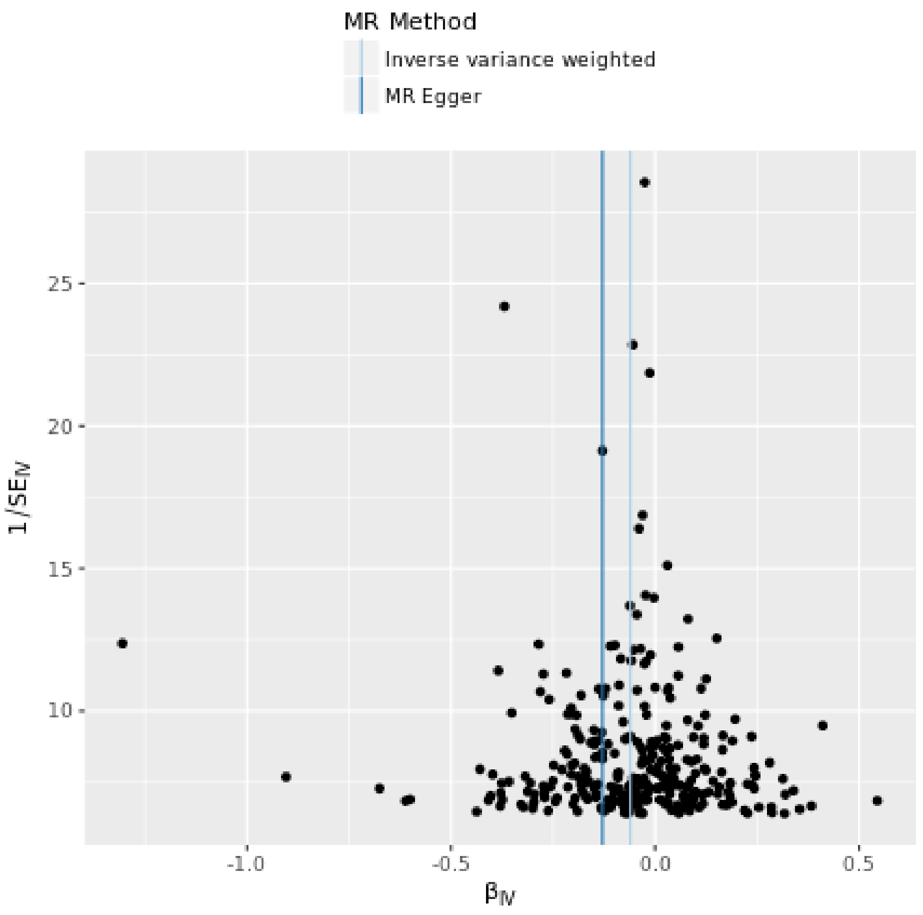

Funnel plot

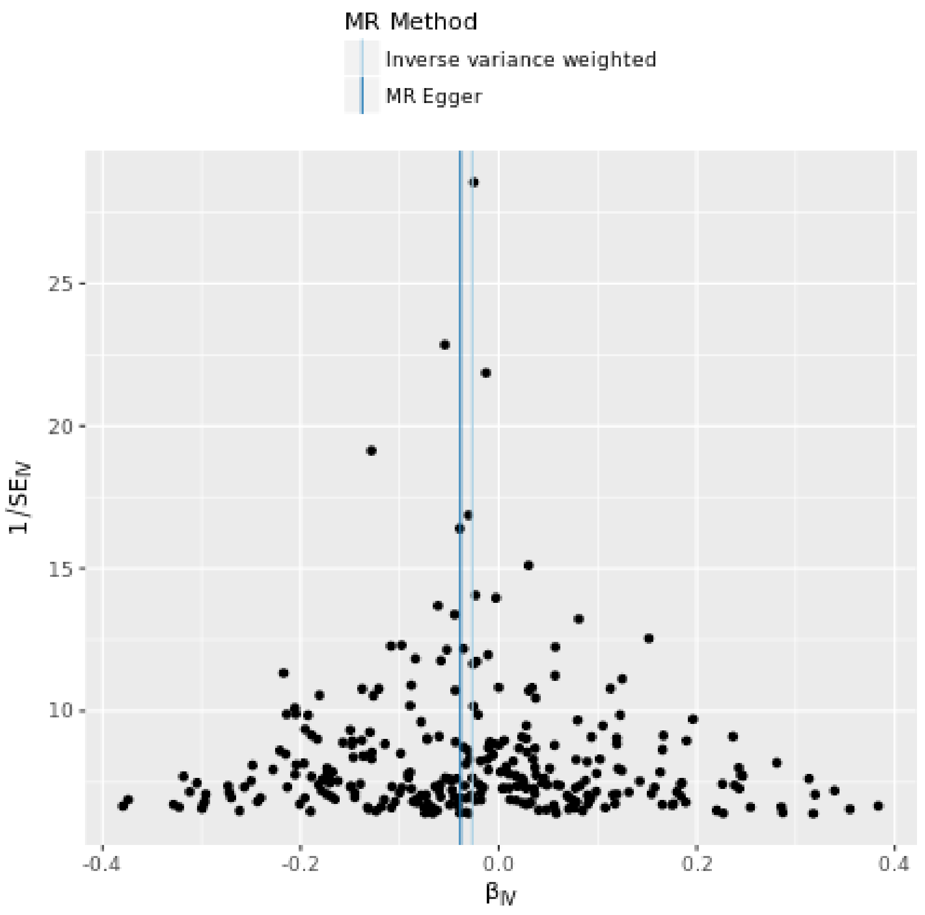

Forced expiratory volume in 1-second (FEV1), Best measure

Before removing instruments	After removing instruments
Leave-one-out plot<br>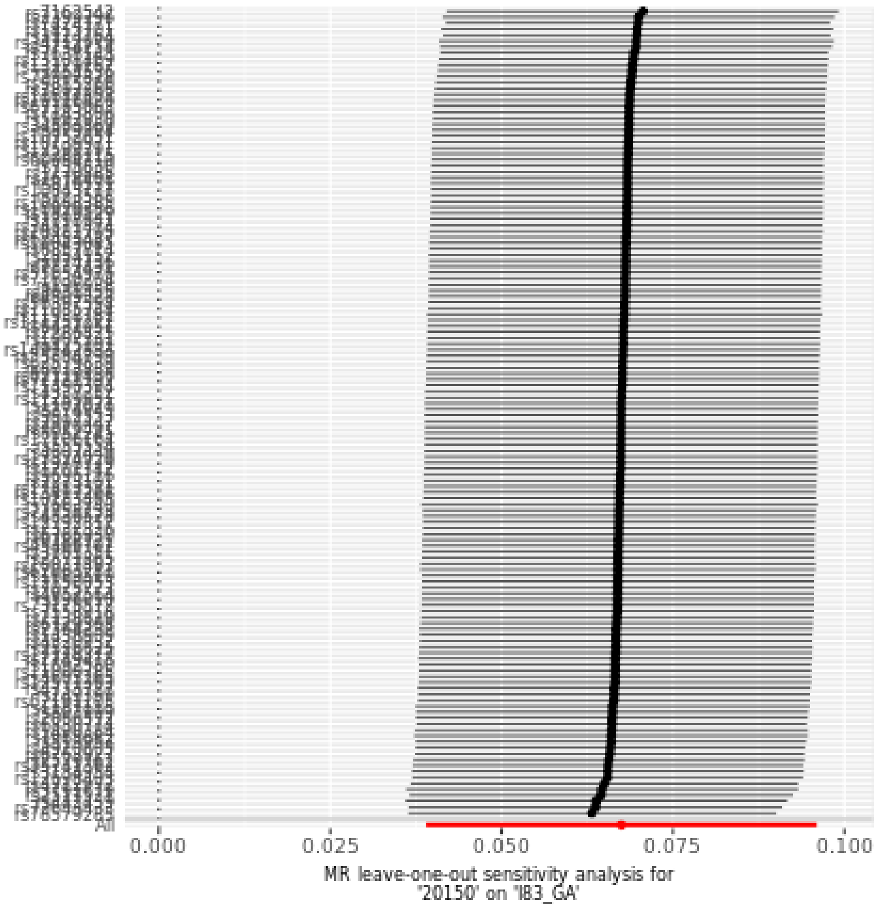	Leave-one-out plot<br>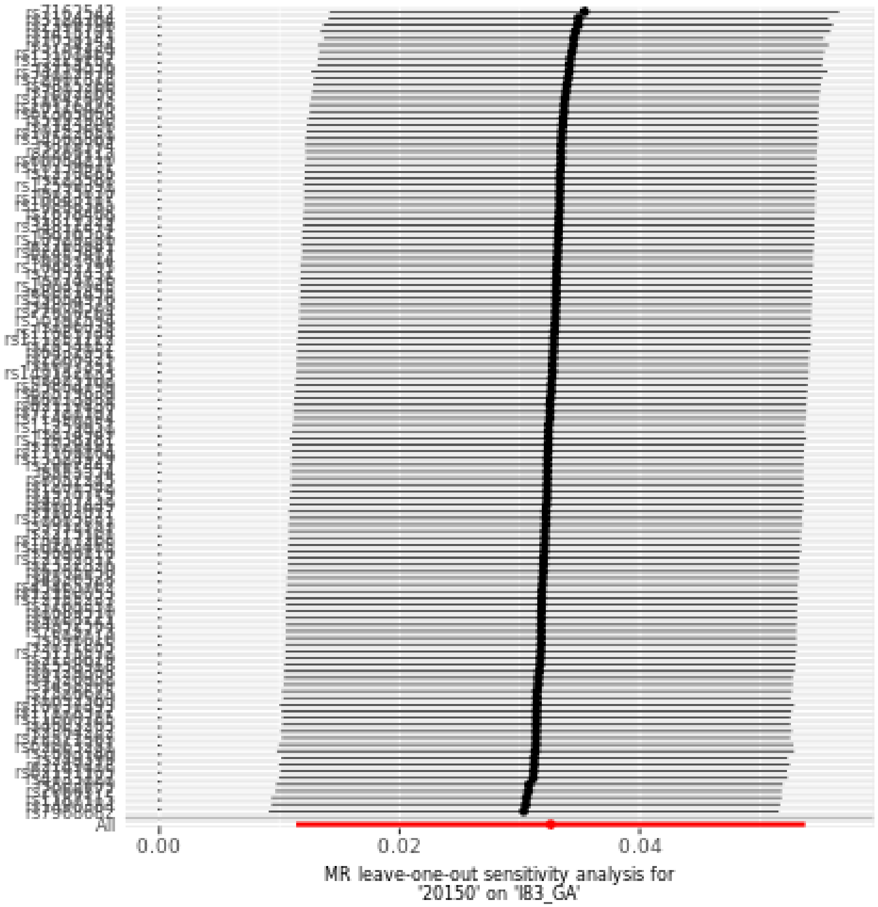

Funnel plot	Funnel plot
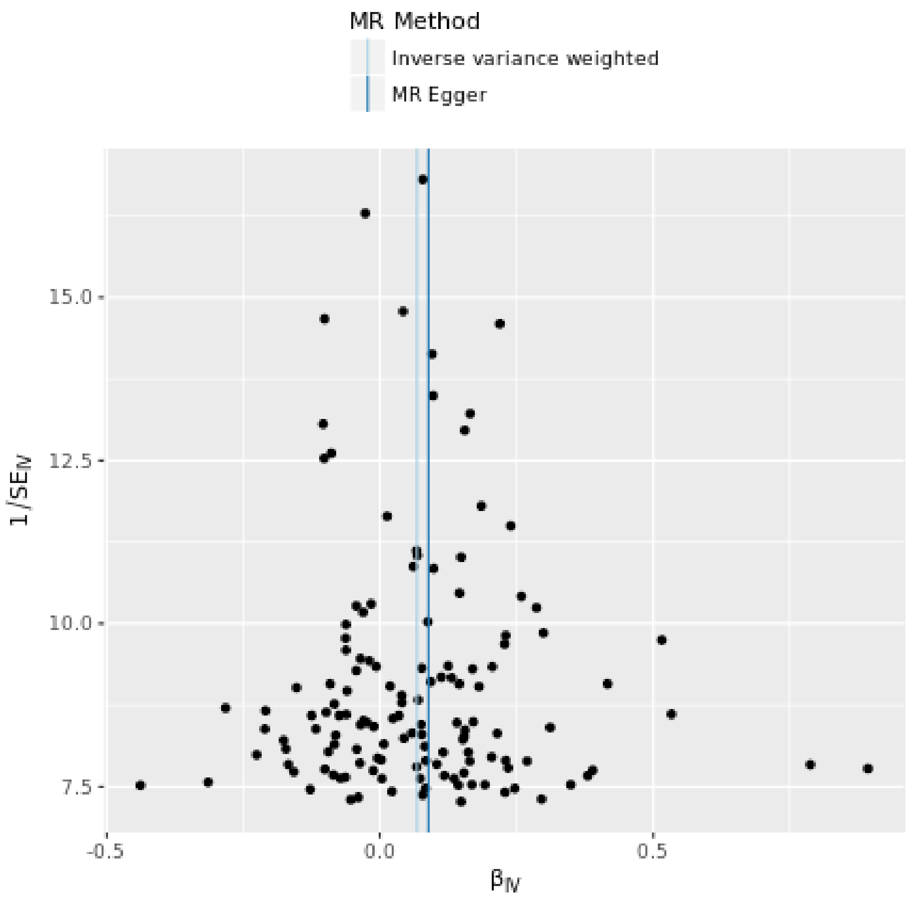	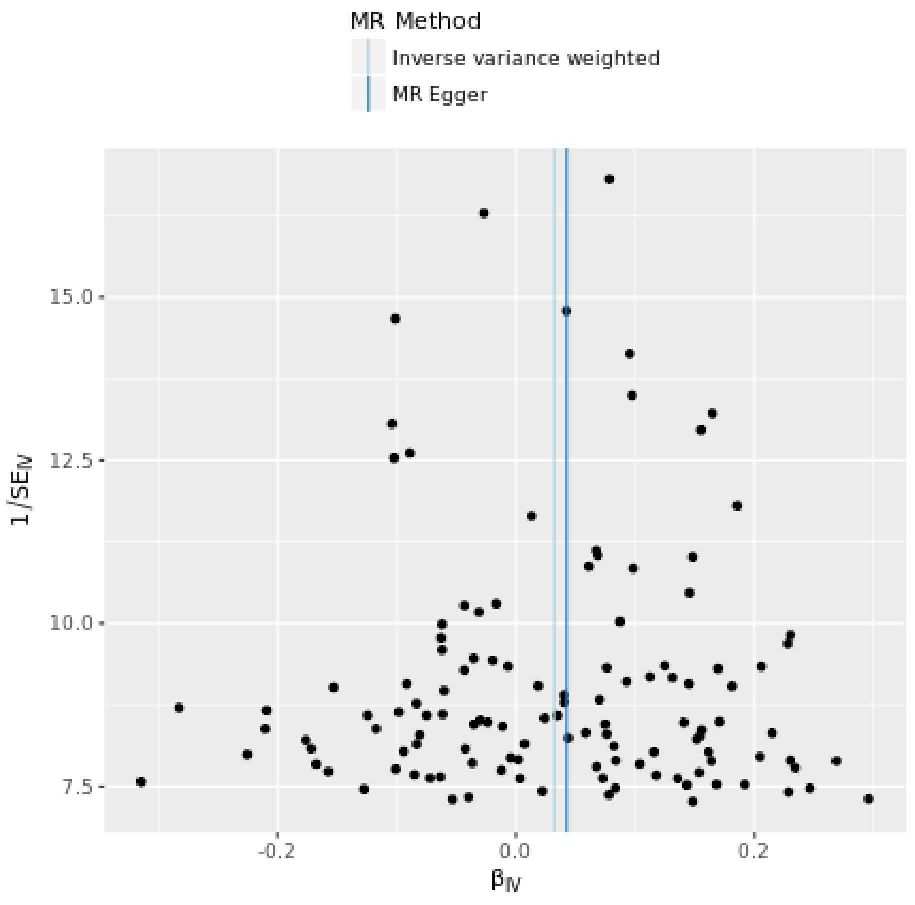

Forced expiratory volume in 1-second (FEV1)

Before removing instruments	After removing instruments
Leave-one-out plot 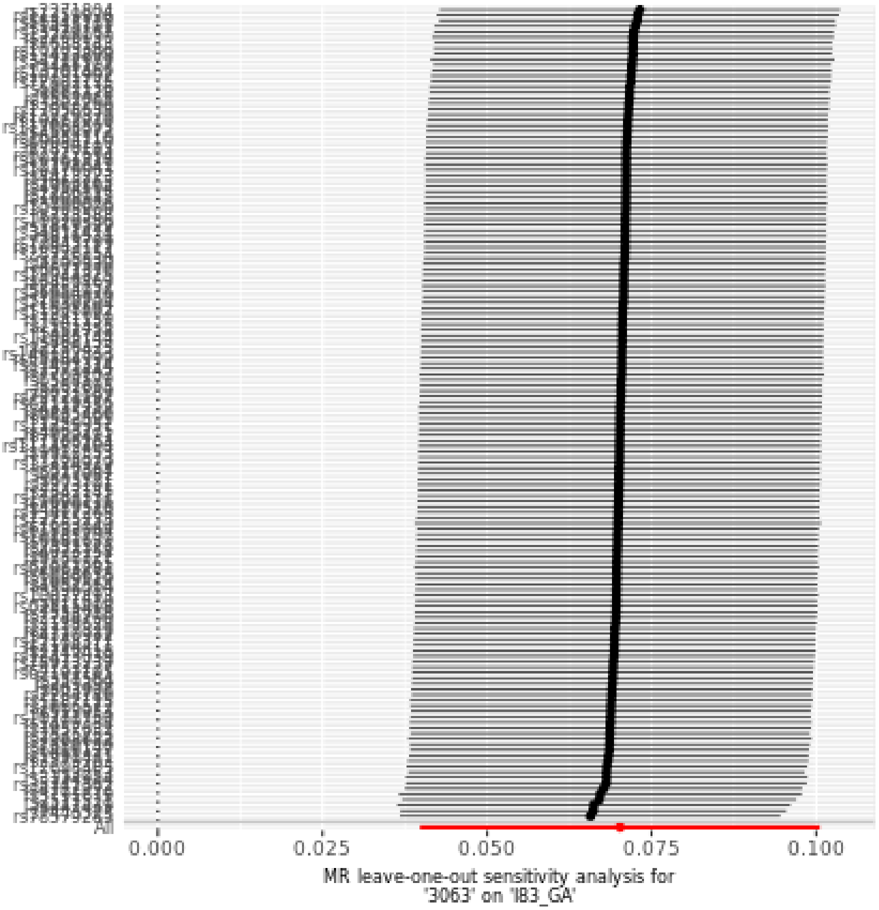	Leave-one-out plot 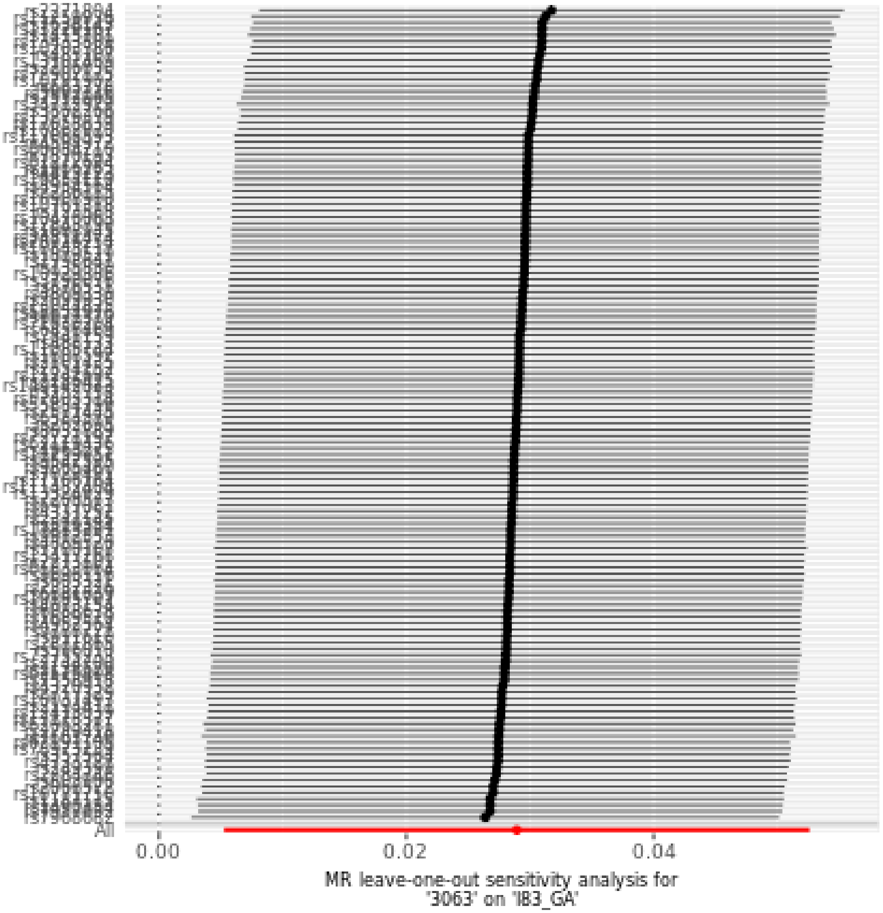

Funnel plot

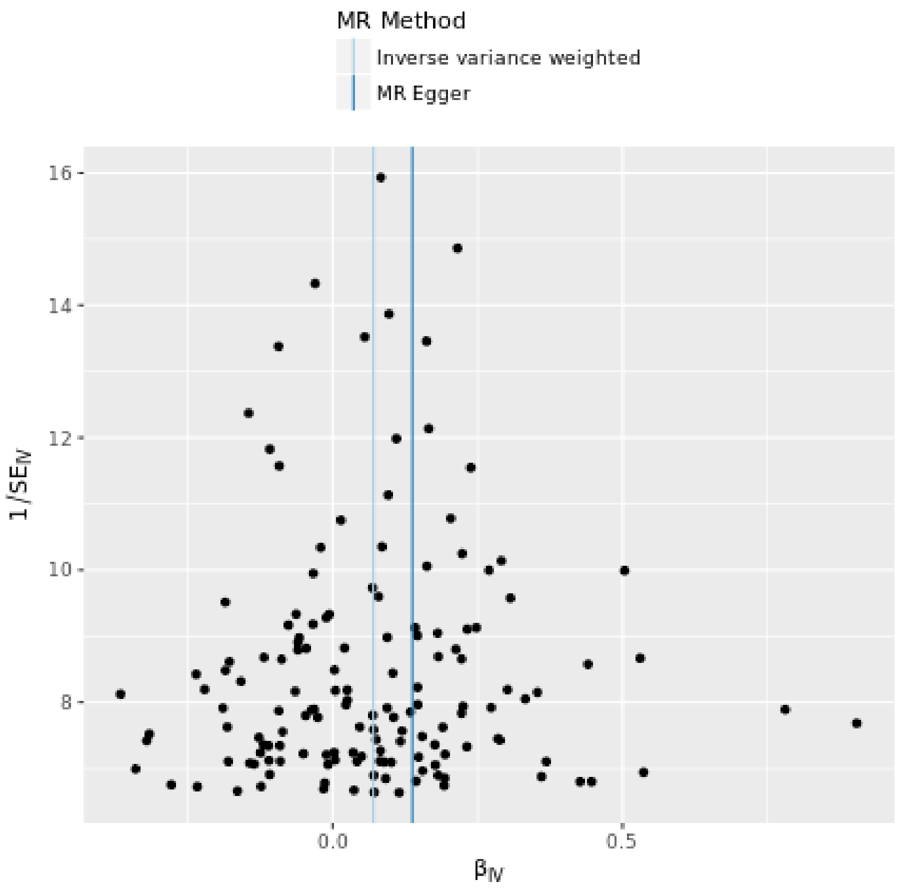

Funnel plot

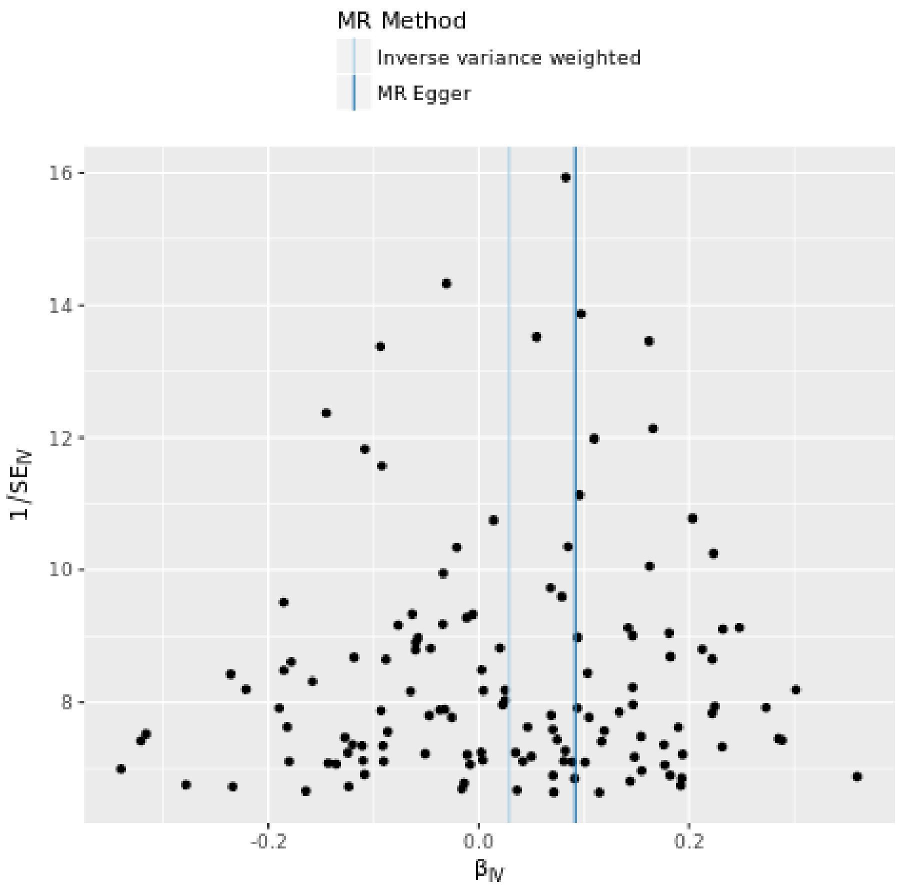

Non-cancer illness code, self-reported: malabsorption/coeliac disease

<table>
<tr><td>Before removing instruments</td><td>After removing instruments</td></tr>
<tr><td>Leave-one-out plot
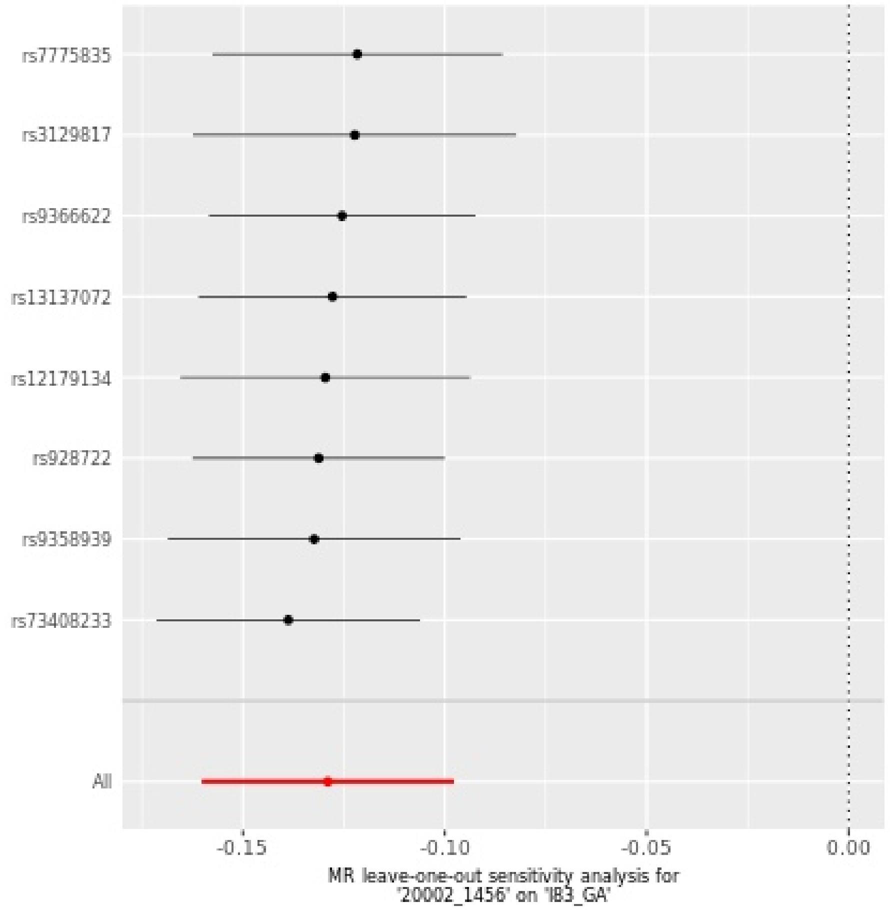
</td><td>Leave-one-out plot
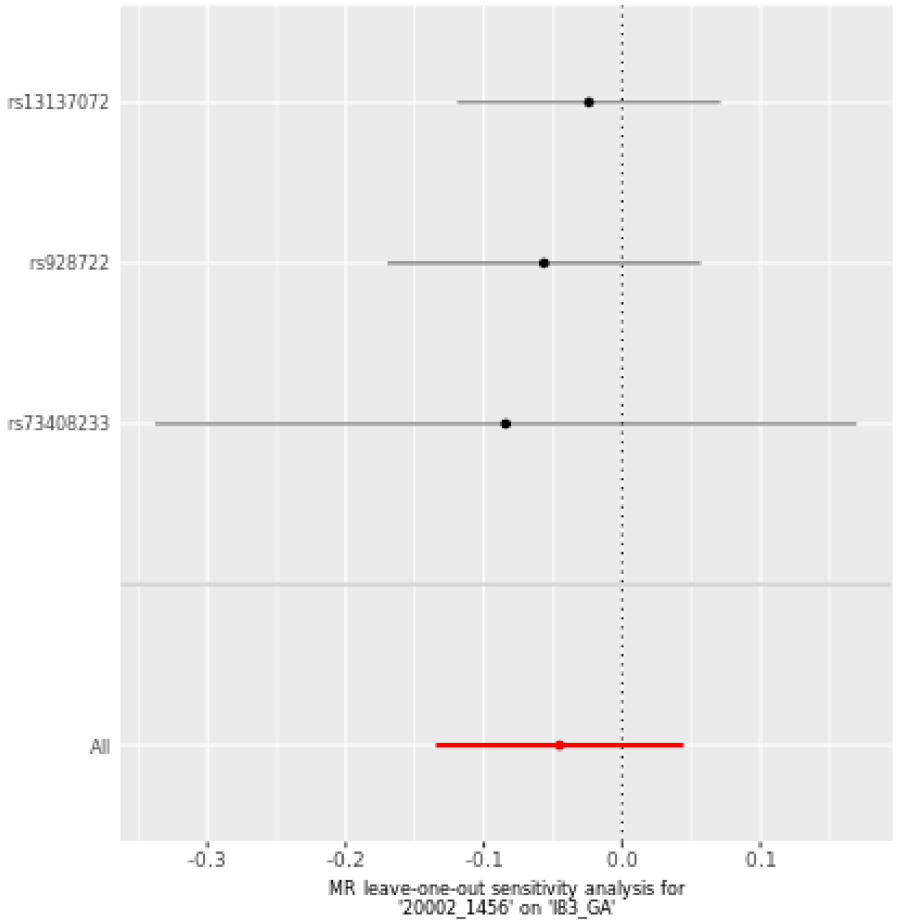
</td></tr>
</table>

Funnel plot

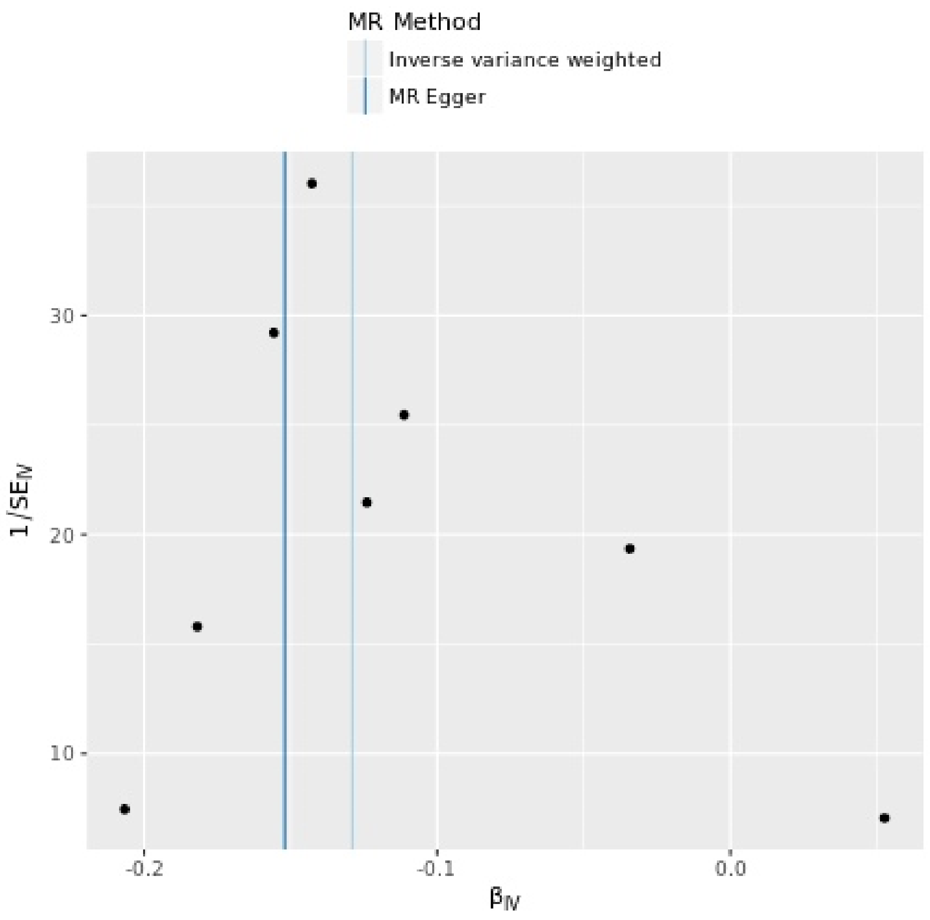

Funnel plot

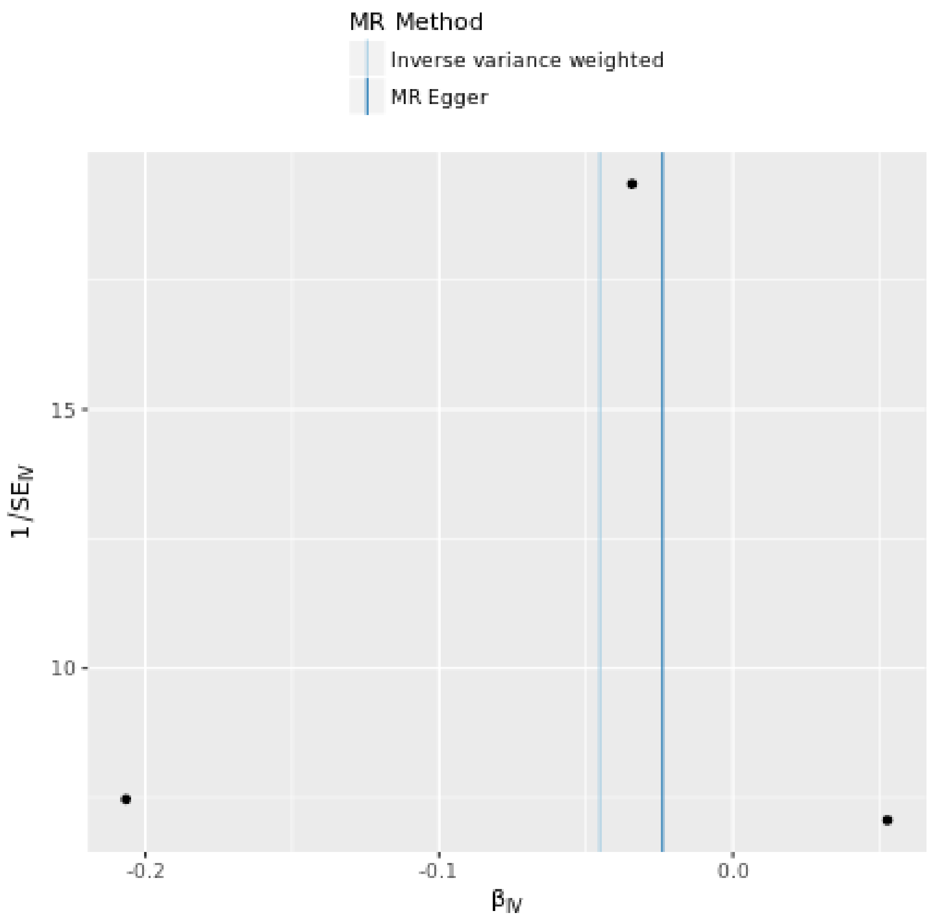

CD209 antigen

Before removing instruments	After removing instruments
Leave-one-out plot<br>***N/A* (insufficient number of SNPs)**	Leave-one-out plot<br>***N/A* (insufficient number of SNPs)**
Funnel plot<br>***N/A* (insufficient number of SNPs)**	Funnel plot<br>***N/A* (insufficient number of SNPs)**

Tissue factor

Before removing instruments	After removing instruments
Leave-one-out plot<br>***N/A* (insufficient number of SNPs)**	Leave-one-out plot<br>***N/A* (insufficient number of SNPs)**
Funnel plot<br>***N/A* (insufficient number of SNPs)**	Funnel plot<br>***N/A* (insufficient number of SNPs)**
