# Supplementary material for: Varicose veins of lower extremities: Insights from the first large-scale genetic study
Source: PLoS Genet. 2019 Apr 18;15(4):e1008110. doi: 10.1371/journal.pgen.1008110 (PMC6490943; doi:10.1371/journal.pgen.1008110)
Supplement: S1 Table — (DOCX) [file pgen.1008110.s007.docx]

**Table S1.** **Genetic correlations between VVs in different datasets included in the analysis.**

**If genetic correlation estimate (rg) was more than 1.00, it was rounded to 1.00.**

|  | **The Neale Lab data  (N =** 337,199) | **The Neale Lab data adjusted for BMI and DVT (N =** 336,107) | The Gene ATLAS data  (N = 408,455) |
| --- | --- | --- | --- |
| **The Neale Lab data**  **(N =** 337,199) | **1.00** | **1.00** | **0.99** |
| **The Neale Lab data adjusted for BMI and DVT (N =** 336,107) | **1.00** | **1.00** | **1.00** |
| The Gene ATLAS data  (N = 408,455) | **0.99** | **1.00** | **1.00** |

**BMI, body mass index; DVT, deep venous thrombosis.**
