# Supplementary material for: Varicose veins of lower extremities: Insights from the first large-scale genetic study
Source: PLoS Genet. 2019 Apr 18;15(4):e1008110. doi: 10.1371/journal.pgen.1008110 (PMC6490943; doi:10.1371/journal.pgen.1008110)
Supplement: S4 Table — (DOC) [file pgen.1008110.s010.doc]

**Table S4.** Gene prioritization based on a literature review. Protein names are indicated in italics.

| **Lead SNP** | **Candidate gene** | **OMIM^*^ code** | **Location** | **Functional effects of the encoded proteins** | **Traits^†^ associated with the revealed loci according to previous GWAS (lead SNP in that study, D’ and r^2^ for LD^‡^ with our lead SNP)** |
| --- | --- | --- | --- | --- | --- |
| **rs11121615** | *CASZ1* | 609895 | Intronic | *Castor zinc finger 1 transcription factor*  Development of heart and blood vessels: blood vessel assembly, lumen formation, and sprouting [1, 2] | **Blood pressure and arterial hypertension** [3-6]  (rs880315 in *CASZ1*, D’ = 0.12, r^2^ = 0.01)  **VVs of lower extremities** [7]  (rs11121615 in *CASZ1*, the same SNP) |
| **rs2911463^§^** | *PIEZO1* | 611184 | Intronic | *Piezo type mechanosensitive ion channel component 1*  Converts mechanical forces into biological signals: senses shear stress in vascular endothelium, smooth muscle cells, red blood cells, and other cells, and mediates stretch-activated currents  Determinant of vascular architecture in both development and adult physiology [8]  Controls epithelial cell number: triggers cell division in response to mechanical stretch and induces cell extrusion in response to crowding [9]  Mediates hypertension-dependent arterial remodeling through smooth muscle cell-related mechanisms [10]  Regulation of blood pressure, vascular tone, NO formation, and blood flow redistribution [11, 12]  Regulation of platelet function: contributes to the shear-dependent thrombus formation under arterial flow (suggestive evidence) [13]  Red blood cell volume regulation [14, 15] | **Mean corpuscular hemoglobin concentration**  [16, 17]  (rs10445033 in *PIEZO1*, D’ = 0.88,  r^2^ = 0.55 with rs2911463, and D’ = 0.84,  r^2^ = 0.12 with rs9972645;  rs9932423 in *PIEZO1*, D’ = 0.93,  r^2^ = 0.86 with rs2911463 and D’ = 0.85,  r^2^ = 0.17 with rs9972645;  rs551118 5’near *PIEZO1*, D’ = 0.96,  r^2^ = 0.557 with rs2911463 and D’ = 0.83,  r^2^ = 0.10 with rs9972645)  **VVs of lower extremities** [7]  (rs4516218 in *PIEZO1*, D’ = 0.48,  r^2^ = 0.08 with rs2911463, and D’ = 0.96,  r^2^ = 0.60 with rs9972645) |
| **rs2861819** | *PPP3R1* | 601302 | 6 kb from the 5’ end | *Protein phosphatase 3 regulatory subunit B, alpha (calcineurin subunit B, type 1)*  Pro-inflammatory effect: induction of cytokine and chemokine (MCP-1) production [18, 19] | **VVs of lower extremities** [7]  (rs6712038 5’near *PPP3R1*, D’ = 1.00,  r^2^ = 0.98) |
| **rs3101725** | *SLC12A2*  *(NKCC1)* | 600840 | 3' UTR | *Solute carrier family 12 member 2*  Membrane ion-transport protein involved in cell-volume regulation  Regulation of vascular tone [20]  Regulation of blood pressure through vascular and renal effects [20] | **Red blood cell distribution width** [17, 21]  (rs17764730 5’near *SLC12A2*, D’ = 0.95,  r^2^ = 0.84; rs10063647 5’near *SLC12A2*,  D’ = 0.93, r^2^ = 0.30; rs10089 in *SLC12A2*, D’ = 1.00, r^2^ = 0.06) |
|  | *FBN2* | 612570 | 70 kb from the 3’ end | *Fibrillin 2*  Extracellular matrix protein, component of connective tissue microfibrils involved in elastic fiber assembly [22]  Is implicated into the assembly of the aortic matrix [23] |  |
| **rs11135046** | *EBF1* | 164343 | Intronic | *Early B cell factor 1*  Transcription factor  Regulation of development and differentiation of B lymphocytes [24]  Regulator of metabolic and inflammatory signaling pathways in mature adipocytes [25] | **Blood pressure and hypertension** [26, 27]  (rs11135046 in *EBF1*, the same SNP, and rs11953630 3’near *EBF1*, D’ = 0.13,  r^2^ = 0.01)  **Cardiometabolic traits** [28]  (rs4704963 in *EBF1*, D’ = 1.00, r^2^ = 0.09) |
| **rs28558138** | *STIM2* | 610841 | 44 kb from the 5’ end | *Stromal interaction molecule 2*  Regulation of Ca^2+^ concentration in cytosol and endoplasmic reticulum [29]  Potential role in vascular remodeling due to smooth muscle cell phenotype alteration [30] | *N/A* |
| **rs7773004** | *HFE* | 613609 | 171 kb from the 5’ end | *Homeostatic iron regulator*  Regulation of iron absorption | **Mean corpuscular hemoglobin concentration and mean corpuscular volume** [16]  (rs7773004 3’near *HFE*, the same SNP)  **Height** [31]  (rs7773004 3’near *HFE,* the same SNP) |

| **rs9880192** | *GATA2* | 137295 | 86 kb from the 5’ end | *GATA binding protein 2*  Transcription factor regulating expression of genes involved in the development and proliferation of hematopoietic cell lineages (master regulator of hematopoiesis), endocrine cell lineages [32], and endothelial lineages [33]  Controls expression of genes involved in establishing endothelial  cell phenotypes and inflammation [34]  Regulates VEGF-induced angiogenesis (including mechanosignaling-dependent angiogenesis sensitive to extracellular matrix elasticity) and lymphangiogenesis via the regulation of *NRP2* and *VEGFR2* transcription [35, 36]  GATA2 is essential for vascular integrity [37] | **Traits related to** **white blood cell count/percentage** (eosinophils, granulocytes, neutrophils, monocytes, lymphocytes) [38-40]  (rs9880192*,* the same SNP) |
| --- | --- | --- | --- | --- | --- |
| **rs12625547** | *NFATC2* | 600490 | Intronic | *Nuclear factor of activated T cells 2*  DNA-binding protein which translocates to the nucleus upon T cell receptor stimulation and becomes a member of the transcription complex playing a central role in inducing gene transcription during the immune response  Modulates inflammatory activity of platelets by mediating *CD40L* expression [41]  Mediates VEGF signal transduction in the HUVEC system  [42, 43]  NFATC2 is potentially involved in the regulation of angiogenic remodeling in the developing vasculature [44] | **Allergic disease** [45]  **Eosinophil percentage** [26]  (rs12625547 in *NFATC2,* the same SNP) |
| **rs236530** | *KCNJ16* | 605722 | 86 kb from the 3’ end | *Potassium voltage-gated channel subfamily J member 16*  Inward-rectifier type potassium channel which may function in fluid and pH balance regulation | **Anthropometric traits** (sitting height, whole body fat-free mass, etc.) [26]  (rs236530*,* the same SNP) |
|  | *KCNJ2* | 600681 | 41 kb from the 3’ end | *Potassium voltage-gated channel subfamily J member 2*  Inward-rectifier type potassium channel which probably participates in establishing action potential waveform and excitability of neuronal and muscle tissues |  |
| **rs2241173** | *SOX9* | 608160 | Intronic  (*LINC01152* gene)  86 kb from the 5’ end of *SOX9* | *SRY-box 9*  Transcription factor essential for both sex and skeletal development (is required for chondrocyte differentiation and cartilage formation [46]). Directly activates the transcription of the *COL2A1* gene [47]  Ectopic *SOX9* expression was shown to mediate extracellular matrix production including that characteristic of organ fibrosis [48]  In mouse models for arterial stenosis, increase in *SOX9* expression was associated with the switch of vascular smooth muscle cells from contractile to proliferative phenotype as well as with alterations in extracellular matrix and calcification [49]  Hypoxia was demonstrated both to up-regulate *SOX9* expression and to trigger osteogenic differentiation and extracellular matrix mineralization of vascular smooth muscle cells [50]  SOX9 is required for extracellular matrix organization during mouse heart valve development [51] | *N/A* |
| **rs73107980** | *COL2A1* | 120140 | Intronic  (*HDAC7* gene)  179 kb from the 3’ end of *COL2A1*  59 kb from the 5’ end of *RAPGEF3* | *Collagen type II alpha 1 chain*  Fibrillar collagen found mainly in cartilage and the vitreous humor of the eye. *COL2A1* gene expression is under control of the *SOX9* gene*.* | **Platelet count and platelet crit** [38, 26]  **Allergic disease** [45]  **Hypertension** [26]  (rs73107980 in *HDAC7,* the same SNP) |
|  | *RAPGEF3 (EPAC1)* | 606057 |  | *Rap guanine nucleotide exchange factor 3*  Controls vascular permeability (mediates integrity of endothelial cell junctions) [52, 53], regulates inflammatory response of vascular endothelial cells (anti‐inflammatory effect) [54] and promotes vascular smooth muscle cell migration [55] |  |

LD, linkage disequilibrium; UTR, untranslated region

^*^Online Mendelian Inheritance in Man® database (<https://www.omim.org/>)

^†^The full list of traits is given in Table S5

^‡^LD were determined for European populations using LDlink online tool (<https://analysistools.nci.nih.gov/LDlink/>)

^§^The second independent signal in this region (tagged by rs9972645) was also located in the *PIEZO1* gen

REFERENCES:

1. Charpentier MS, Christine KS, Amin NM, Dorr KM, Kushner EJ, Bautch VL, et al. CASZ1 promotes vascular assembly and morphogenesis through the direct regulation of an EGFL7/RhoA-mediated pathway. Dev Cell. 2013;25: 132–143. doi:10.1016/j.devcel.2013.03.003

2. Charpentier MS, Dorr KM, Conlon FL. Transcriptional regulation of blood vessel formation. Cell Cycle. 2013;12: 2165–2166. doi:10.4161/cc.25539

3. Levy D, Ehret GB, Rice K, Verwoert GC, Launer LJ, Dehghan A, et al. Genome-wide association study of blood pressure and hypertension. Nat Genet. 2009;41: 677–687. doi:10.1038/ng.384

4. Kato N, Takeuchi F, Tabara Y, Kelly TN, Go MJ, Sim X, et al. Meta-analysis of genome-wide association studies identifies common variants associated with blood pressure variation in east Asians. Nat Genet. 2011;43: 531–538. doi:10.1038/ng.834

5. Lu X, Wang L, Lin X, Huang J, Charles Gu C, He M, et al. Genome-wide association study in Chinese identifies novel loci for blood pressure and hypertension. Hum Mol Genet. 2015;24: 865–874. doi:10.1093/hmg/ddu478

6. Takeuchi F, Isono M, Katsuya T, Yamamoto K, Yokota M, Sugiyama T, et al. Blood pressure and hypertension are associated with 7 loci in the Japanese population. Circulation. 2010;121: 2302–2309. doi:10.1161/CIRCULATIONAHA.109.904664

7. Bell RK, Durand EY, McLean CY, Eriksson N, Tung JY, Hinds D. A large scale genome wide association study of varicose veins in the 23andMe cohort. In: The 64th Annual Meeting of The American Society of Human Genetics, San Diego, California, USA, 18-22 October 2014, paper no. 2082M, p.487. San Diego: ASHG. <https://blog.23andme.com/wp-content/uploads/2014/10/Bell_ASHG2014_varicose.pdf>

8. Li J, Hou B, Tumova S, Muraki K, Bruns A, Ludlow MJ, et al. Piezo1 integration of vascular architecture with physiological force. Nature. 2014;515: 279–282. doi:10.1038/nature13701

9. Gudipaty SA, Lindblom J, Loftus PD, Redd MJ, Edes K, Davey CF, et al. Mechanical stretch triggers rapid epithelial cell division through Piezo1. Nature. 2017;543: 118–121. doi:10.1038/nature21407

10. Retailleau K, Duprat F, Arhatte M, Ranade SS, Peyronnet R, Martins JR, et al. Piezo1 in smooth muscle cells is involved in hypertension-dependent arterial remodeling. Cell Rep. 2015;13: 1161–1171. doi:10.1016/j.celrep.2015.09.072

11. Rode B, Shi J, Endesh N, Drinkhill MJ, Webster PJ, Lotteau SJ, et al. Piezo1 channels sense whole body physical activity to reset cardiovascular homeostasis and enhance performance. Nat Commun. 2017;8: 350. doi:10.1038/s41467-017-00429-3

12. Wang S, Chennupati R, Kaur H, Iring A, Wettschureck N, Offermanns S. Endothelial cation channel PIEZO1 controls blood pressure by mediating flow-induced ATP release. J Clin Invest. 2016;126: 4527–4536. doi:10.1172/JCI87343

13. Ilkan Z, Wright JR, Goodall AH, Gibbins JM, Jones CI, Mahaut-Smith MP. Evidence for shear-mediated Ca2+entry through mechanosensitive cation channels in human platelets and a megakaryocytic cell line. J Biol Chem. 2017;292: 9204–9217. doi:10.1074/jbc.M116.766196

14. Faucherre A, Kissa K, Nargeot J, Mangoni ME, Jopling C. Piezo1 plays a role in erythrocyte volume homeostasis. Haematologica. 2014;99: 70–75. doi:10.3324/haematol.2013.086090

15. Cahalan SM, Lukacs V, Ranade SS, Chien S, Bandell M, Patapoutian A. Piezo1 links mechanical forces to red blood cell volume. Elife. 2015;4. doi:10.7554/eLife.07370

16. van der Harst P, Zhang W, Mateo Leach I, Rendon A, Verweij N, Sehmi J, et al. Seventy-five genetic loci influencing the human red blood cell. Nature. 2012;492: 369–375. doi:10.1038/nature11677

17. Hodonsky CJ, Jain D, Schick UM, Morrison J V., Brown L, McHugh CP, et al. Genome-wide association study of red blood cell traits in Hispanics/Latinos: The Hispanic Community Health Study/Study of Latinos. PLOS Genet. 2017;13: e1006760. doi:10.1371/journal.pgen.1006760

18. Wu W, Chen Q, Geng F, Tong L, Yang R, Yang J, et al. Calcineurin B stimulates cytokine production through a CD14-independent Toll-like receptor 4 pathway. Immunol Cell Biol. 2016;94: 285–292. doi:10.1038/icb.2015.91

19. Satonaka H, Suzuki E, Nishimatsu H, Oba S, Takeda R, Goto A, et al. Calcineurin promotes the expression of monocyte chemoattractant protein-1 in vascular myocytes and mediates vascular inflammation. Circ Res*.* 2004;94: 693-700. doi:10.1161/01.RES.0000118250.67032.5E

20. Orlov SN, Tremblay J, Hamet P. NKCC1 and hypertension: a novel therapeutic target involved in the regulation of vascular tone and renal function. Curr Opin Nephrol Hypertens. 2010;19: 163–168. doi:10.1097/MNH.0b013e3283360a46

21. Chami N, Chen M-H, Slater AJ, Eicher JD, Evangelou E, Tajuddin SM, et al. Exome genotyping identifies pleiotropic variants associated with red blood cell traits. Am J Hum Genet. 2016;99: 8–21. doi:10.1016/j.ajhg.2016.05.007

22. Olivieri J, Smaldone S, Ramirez F. Fibrillin assemblies: extracellular determinants of tissue formation and fibrosis. Fibrogenesis Tissue Repair. 2010;3: 24. doi:10.1186/1755-1536-3-24

23. Carta L, Pereira L, Arteaga-Solis E, Lee-Arteaga SY, Lenart B, Starcher B, et al. Fibrillins 1 and 2 perform partially overlapping functions during aortic development. J Biol Chem. 2006;281: 8016–8023. doi:10.1074/jbc.M511599200

24. Gisler R, Jacobsen EW, Sigvardsson M. Cloning of human early B-cell factor and identification of target genes suggest a conserved role in B-cell development in man and mouse. Blood. 2000;96**:** 1457-1464.

25. Griffin MJ, Zhou Y, Kang S, Zhang X, Mikkelsen TS, Rosen ED. Early B-cell factor-1 (EBF1) is a key regulator of metabolic and inflammatory signaling pathways in mature adipocytes. J Biol Chem. 2013;288: 35925–35939. doi:10.1074/jbc.M113.491936

26. <http://geneatlas.roslin.ed.ac.uk/>

Canela-Xandri O, Rawlik K, Tenesa A. An atlas of genetic associations in UK Biobank. *bioRxiv* doi: 10.1101/176834 [PREPRINT]

Canela-Xandri O, Rawlik K, Tenesa A. An atlas of genetic associations in UK Biobank. Nat Genet. 2018;50: 1593–1599. doi:10.1038/s41588-018-0248-z

27. Ehret GB, Munroe PB, Rice KM, Bochud M, Johnson AD, Chasman DI, et al. Genetic variants in novel pathways influence blood pressure and cardiovascular disease risk. Nature. 2011;478: 103–109. doi:10.1038/nature10405

28. Singh A, Babyak MA, Nolan DK, Brummett BH, Jiang R, Siegler IC, et al. Gene by stress genome-wide interaction analysis and path analysis identify EBF1 as a cardiovascular and metabolic risk gene. Eur J Hum Genet. 2015;23: 854–862. doi:10.1038/ejhg.2014.189

29. Brandman O, Liou J, Park WS, Meyer T. STIM2 is a feedback regulator that stabilizes basal cytosolic and endoplasmic reticulum Ca2+ levels. Cell. 2007;131: 1327–1339. doi:10.1016/j.cell.2007.11.039

30. Fernandez RA, Wan J, Song S, Smith KA, Gu Y, Tauseef M, et al. Upregulated expression of STIM2, TRPC6, and Orai2 contributes to the transition of pulmonary arterial smooth muscle cells from a contractile to proliferative phenotype. Am J Physiol Physiol. 2015;308: C581–C593. doi:10.1152/ajpcell.00202.2014

31. Wood AR, Esko T, Yang J, Vedantam S, Pers TH, Gustafsson S, et al. Defining the role of common variation in the genomic and biological architecture of adult human height. Nat Genet. 2014;46: 1173–1186. doi:10.1038/ng.3097

32. Viger RS, Guittot SM, Anttonen M, Wilson DB, Heikinheimo M. Role of the GATA family of transcription factors in endocrine development, function, and disease. Mol Endocrinol. 2008;22: 781–798. doi:10.1210/me.2007-0513

33. Shi X, Richard J, Zirbes KM, Gong W, Lin G, Kyba M, et al. Cooperative interaction of Etv2 and Gata2 regulates the development of endothelial and hematopoietic lineages. Dev Biol. 2014;389: 208–218. doi:10.1016/J.YDBIO.2014.02.018

34. Linnemann AK, O’Geen H, Keles S, Farnham PJ, Bresnick EH. Genetic framework for GATA factor function in vascular biology. Proc Natl Acad Sci. 2011;108: 13641–13646. doi:10.1073/pnas.1108440108

35. Coma S, Allard-Ratick M, Akino T, van Meeteren LA, Mammoto A, Klagsbrun M. GATA2 and Lmo2 control angiogenesis and lymphangiogenesis via direct transcriptional regulation of neuropilin-2. Angiogenesis. 2013;16: 939–952. doi:10.1007/s10456-013-9370-9

36. Mammoto A, Connor KM, Mammoto T, Yung CW, Huh D, Aderman CM, et al. A mechanosensitive transcriptional mechanism that controls angiogenesis. Nature. 2009;457: 1103–1108. doi:10.1038/nature077655

37. Johnson KD, Hsu AP, Ryu M-J, Wang J, Gao X, Boyer ME, et al. Cis-element mutated in GATA2-dependent immunodeficiency governs hematopoiesis and vascular integrity. J Clin Invest. 2012;122: 3692–3704. doi:10.1172/JCI61623

38. Astle WJ, Elding H, Jiang T, Allen D, Ruklisa D, Mann AL, et al. The allelic landscape of human blood cell trait variation and links to common complex disease. Cell. 2016;167: 1415–1429.e19. doi:10.1016/j.cell.2016.10.042

39. Nalls MA, Couper DJ, Tanaka T, van Rooij FJA, Chen M-H, Smith A V., et al. Multiple loci are associated with white blood cell phenotypes. PLoS Genet. 2011;7: e1002113. doi:10.1371/journal.pgen.1002113

40. Keller MF, Reiner AP, Okada Y, van Rooij FJA, Johnson AD, Chen M-H, et al. Trans-ethnic meta-analysis of white blood cell phenotypes. Hum Mol Genet. 2014;23: 6944–6960. doi:10.1093/hmg/ddu401

41. Crist SA, Elzey BD, Ahmann MT, Ratliff TL. Early growth response-1 (EGR-1) and nuclear factor of activated T cells (NFAT) cooperate to mediate CD40L expression in megakaryocytes and platelets. J Biol Chem. 2013;288: 33985–33996. doi:10.1074/jbc.M113.511881

42. Armesilla AL, Lorenzo E, Gómez del Arco P, Martínez-Martínez S, Alfranca A, Redondo JM. Vascular endothelial growth factor activates nuclear factor of activated T cells in human endothelial cells: a role for tissue factor gene expression. Mol Cell Biol. 1999;19: 2032–2043.

43. Hernández GL, Volpert O V, Iñiguez MA, Lorenzo E, Martínez-Martínez S, Grau R, et al. Selective inhibition of vascular endothelial growth factor-mediated angiogenesis by cyclosporin A: roles of the nuclear factor of activated T cells and cyclooxygenase 2. J Exp Med. 2001;193: 607–620.

44. Schulz RA, Yutzey KE. Calcineurin signaling and NFAT activation in cardiovascular and skeletal muscle development. Dev Biol. 2004;266: 1–16.

45. Ferreira MA, Vonk JM, Baurecht H, Marenholz I, Tian C, Hoffman JD, et al. Shared genetic origin of asthma, hay fever and eczema elucidates allergic disease biology. Nat Genet. 2017;49: 1752–1757. doi:10.1038/ng.3985

46. Bi W, Deng JM, Zhang Z, Behringer RR, de Crombrugghe B. Sox9 is required for cartilage formation. Nat Genet. 1999;22: 85–89. doi:10.1038/8792

47. Bell DM, Leung KKH, Wheatley SC, Ng LJ, Zhou S, Wing Ling K, et al. SOX9 directly regulates the type-ll collagen gene. Nat Genet. 1997;16: 174–178. doi:10.1038/ng0697-174

48. Hanley KP, Oakley F, Sugden S, Wilson DI, Mann DA, Hanley NA. Ectopic SOX9 mediates extracellular matrix deposition characteristic of organ fibrosis. J Biol Chem. 2008;283: 14063–14071. doi:10.1074/jbc.M707390200

49. Augstein A, Mierke J, Poitz DM, Strasser RH. Sox9 is increased in arterial plaque and stenosis, associated with synthetic phenotype of vascular smooth muscle cells and causes alterations in extracellular matrix and calcification. Biochim Biophys Acta - Mol Basis Dis. 2018;1864: 2526–2537. doi:10.1016/j.bbadis.2018.05.009

50. Jeney V, Enikő B, Emese T, Andrea T. A novel player in vascular calcification: Hypoxia induces osteogenic differentiation and extracellular matrix mineralization of vascular smooth muscle cells. Atherosclerosis. 2016;252: e235. doi:10.1016/j.atherosclerosis.2016.07.230

51. Lincoln J, Kist R, Scherer G, Yutzey KE. Sox9 is required for precursor cell expansion and extracellular matrix organization during mouse heart valve development. Dev Biol. 2007;305: 120–132. doi:10.1016/J.YDBIO.2007.02.002

52. Rampersad SN, Ovens JD, Huston E, Umana MB, Wilson LS, Netherton SJ, et al. Cyclic AMP phosphodiesterase 4D (PDE4D) Tethers EPAC1 in a vascular endothelial cadherin (VE-Cad)-based signaling complex and controls cAMP-mediated vascular permeability. J Biol Chem. 2010;285: 33614–33622. doi:10.1074/jbc.M110.140004

53. Kooistra MRH, Corada M, Dejana E, Bos JL. Epac1 regulates integrity of endothelial cell junctions through VE-cadherin. FEBS Lett. 2005;579: 4966–4972. doi:10.1016/j.febslet.2005.07.080

54. Parnell E, Smith BO, Palmer TM, Terrin A, Zaccolo M, Yarwood SJ. Regulation of the inflammatory response of vascular endothelial cells by EPAC1. Br J Pharmacol. 2012;166: 434–446. doi:10.1111/j.1476-5381.2011.01808.x

55. Yokoyama U, Minamisawa S, Quan H, Akaike T, Jin M, Otsu K, et al. Epac1 is upregulated during neointima formation and promotes vascular smooth muscle cell migration. Am J Physiol Circ Physiol. 2008;295: H1547–H1555. doi:10.1152/ajpheart.01317.2007
